# Photoinduced Deoxygenative Boration of Unactivated Alcohols Involving In-Situ-Formed Alkyl Iodides

**DOI:** 10.1021/acs.orglett.5c04280

**Published:** 2025-11-11

**Authors:** Xiaojie Liu, Biping Xu, Martin Oestreich

**Affiliations:** Institut für Chemie, 26524 Technische Universität Berlin, Strasse des 17. Juni 115, 10623 Berlin, Germany

## Abstract

A method for the direct two-step/one-pot conversion of unactivated 1°, 2°, and 3° alcohols into the corresponding alkylboronate esters is reported. The hydroxy group is activated in situ by reaction with the Hendrickson “POP” reagent. The rapidly formed alkoxyphosphonium salt is then reacted with bis­(catecholato)­diboron under visible-light irradiation in the presence of stoichiometric amounts of lithium iodide. This one-pot protocol streamlines the two-step procedure, and it is operationally simple and scalable. The functional-group tolerance is broad.

## Graphical abstract

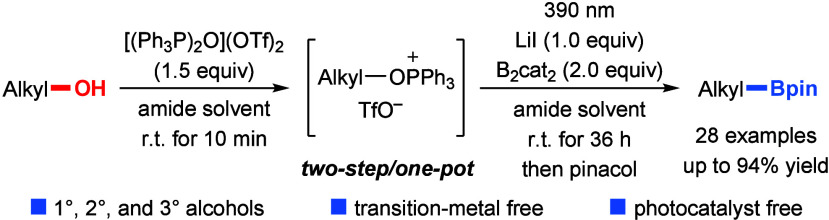

Alkylboron compounds are versatile building blocks in organic synthesis.^1^ A common way to access these key intermediates is by hydroboration of alkenes, especially α-olefins where there are no regioselectivity issues (Scheme 1, path a).^2^ An alternative approach that also overcomes that regiochemistry challenge is dehaloboration of alkyl halides under thermo-,^3^ photo-,^4^ or electrochemical^5^ conditions (path b). Although the range of commercially available alkyl halides is rather broad (with the exception of chemically labile alkyl iodides), alcohols are an even wider and naturally abundant alkyl source.^6^ Hence, the formal deoxyboration of alcohols would greatly expand the synthetic toolbox. And indeed, a number of two-step procedures that rely on prior conversion of the hydroxy group into a leaving group (LG) were developed (path c).^7^ To avoid the additional manipulation as well as the purification step, the more direct transformation of alcohols into the corresponding boronates is highly desirable, yet such a transformation has only begun to emerge very recently (path d).^8^ In continuation of our studies on deoxygenative transformations of alcohols,^9^ we herein report a robust two-step/one-pot method that converts a broad range of 1°, 2°, and 3° alcohols into alkylboronate esters (path e). As in our previous deoxysilylation,^9^ we make use of the Hendrickson “POP” reagent for rapid in situ activation of the hydroxy group followed by alkyl iodide formation with lithium iodide. The actual key step is Studer’s visible-light-promoted deiodoboration,^4f^ which is compatible with the byproducts from the halogenation step.

**Scheme 1:**
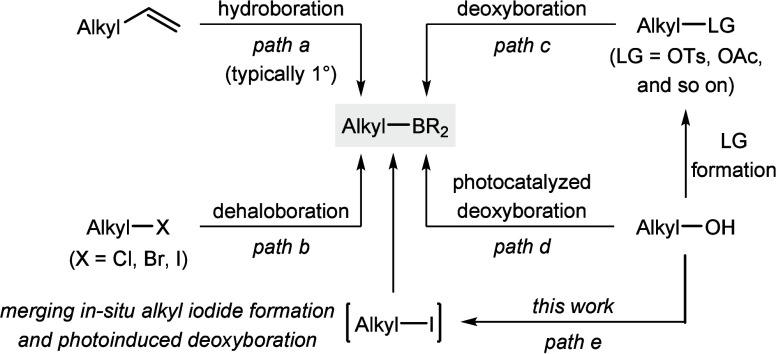
Methods for the Synthesis of Alkylboron Compounds by C­(sp^3^)–B Bond Formation

We commenced our study with 3-phenyl-1-propanol (**1a**) and bis­(catecholato)­diboron (B_2_cat_2_) as starting materials together with alcohol activation reagents that facilitate C­(sp^3^)–O bond cleavage (Table 1). After systematic optimization of the reaction conditions (see the Supporting Information for details), the desired borylated product **2a** (after transesterification with pinacol) was detected in 86% yield in the presence of the Hendrickson reagent and lithium iodide in DMF (entry 1). It made no difference to use a fan for cooling to maintain room temperature (Figure S1 in the Supporting Information). Omitting either the Hendrickson reagent, lithium iodide, or irradiation with 390 nm almost completely shut down the reaction, underscoring that all three are indispensable (entries 2–4). Changing the irradiation wavelength from 390 to 456 nm resulted in a significant decline in reaction efficiency (50% instead of 86% yield; entry 5). Attempts to thermally drive the reaction were unsuccessful, as the yield under heating was nearly identical with that at room temperature (entry 6 versus entry 4). Replacing lithium iodide with lithium bromide led to a diminished yield of only 6% (entry 7). No desired product was detected when bis­(pinacolato)­diboron (B_2_pin_2_) was used instead of B_2_cat_2_ but alkyl iodide formation proceeded as desired (entry 8). Notably, the reaction proceeded exclusively in amide solvents, giving 37% and 50% yields in DMA and NMP, respectively (entries 9 and 10). No product was observed in other solvents such as toluene or acetonitrile yet for different reasons: nearly no formation of the alkyl iodide but decomposition in toluene (entry 11) and clean formation of the alkyl iodide by no photochemical boration in acetonitrile (entry 12). Almost quantitative yield was obtained at a prolonged reaction time of 36 h (entry 13). A one-step/one-pot setup, that is, with no prestirring of the alcohol and the POP reagent, brought about lower yields.

**Table 1:**
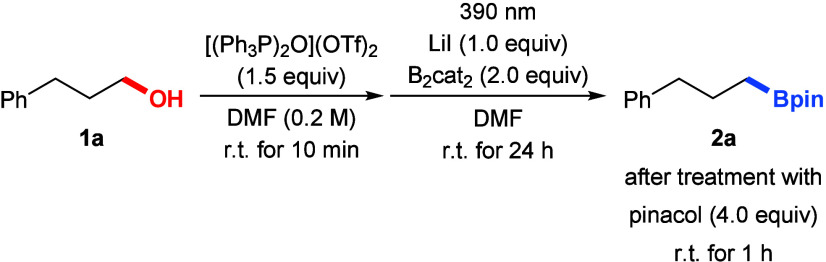
Selected Examples of the Optimization^a^

entry	variation from the standard conditions	yield of **2a** (%)^b^
1	none	86
2	w/o POP	NR
3	w/o LiI	6
4	in the dark	14
5	456 nm instead of 390 nm	50
6	100 °C in the dark	15
7	LiBr instead of Lil	6
8	B_2_pin_2_ instead of B_2_cat_2_	ND
9	DMA instead of DMF	37
10	NMP instead of DMF	50
11	toluene instead of DMF	ND
12	MeCN instead of DMF	ND
13	36 h instead of 24 h	98 (94^c^)

a Reaction conditions: Alcohol **1a** (0.20 mmol), POP (1.5 equiv), and solvent (1.0 mL) were mixed and stirred at room temperature for 10 min; B_2_cat_2_ (2.0 equiv) and LiI (1.0 equiv) were subsequently added, and then the reaction was irradiated with an LED at 390 nm at room temperature for 24 h. The reaction was terminated by the addition of pinacol (4.0 equiv) and Et_3_N (0.70 mL), and the resulting solution was maintained at room temperature for 1 h.

b The yield was determined by gas–liquid chromatography (GLC) analysis with methyl benzoate as an internal standard.

c Isolated yield. NR = no reaction. ND = not detected. DMF = *N*,*N*-dimethylformamide. DMA = *N*,*N*-dimethylacetamide. NMP = *N*-methyl-2-pyrrolidone.

With the optimized reaction conditions in hand, we next evaluated the substrate scope of the deoxygenative boration reaction across a range of alcohol substrates (Scheme 2). Consistently high yields were obtained for 1° alcohols with different aliphatic chain lengths (**1a**–**c** → **2a**–**c**) and whether a phenyl substituent was present (**2d**). Yields were slightly lower with alcohols bearing a biphenyl (**1e** → **2e**) or naphthyl group (**1f** → **2f**) at the alkyl terminus. Moreover, a double boration of 1,6-hexanediol (**1g**) proceeded smoothly with a 2-fold excess of the reagents, producing the diboronate ester **2g** in 68% isolated yield. The (*E*)-stilbene-containing alcohol **1h** reacted in 60% yield, yet **2h** was formed as a mixture of diastereomers (*E*:*Z* = 50:50). Alcohol **1i** containing a distal alkyne moiety converted chemoselectively into desired product **2i** in good yield. Ether functional groups as in **2j**–**l** and a carbazole moiety as in **2m** were compatible, affording moderate to good yields. Also, the alkyl chloride as in **2n** as well as a bromo and a trimethylsilyl substituent at an aryl group as in **2o** and **2p** were tolerated. Acyclic 2° alcohols reacted equally well (**3a**–**c** → **4a**–**c**) when DMF was replaced with DMA as the solvent under the optimized conditions; yields in DMF were approximately 25% in all three cases. In turn, a significant decrease in yield was found for cyclic 2° alcohols (**3d**–**f** → **4d**–**f**) which could be attributed to the inefficient conversion of these alcohols into the corresponding alkyl iodides; cyclohexane derivative **4f** favored the *trans* relationship with d.r. = 88:12 starting from the *trans*-configured alcohol **3f**. As an example of a 3° alcohol, 1-adamantanol (**5**) reacted efficiently after another solvent change from DMF to NMP (**5** → **6**); yields in DMF and DMA were 20% and 37%, respectively.

**Scheme 2:**
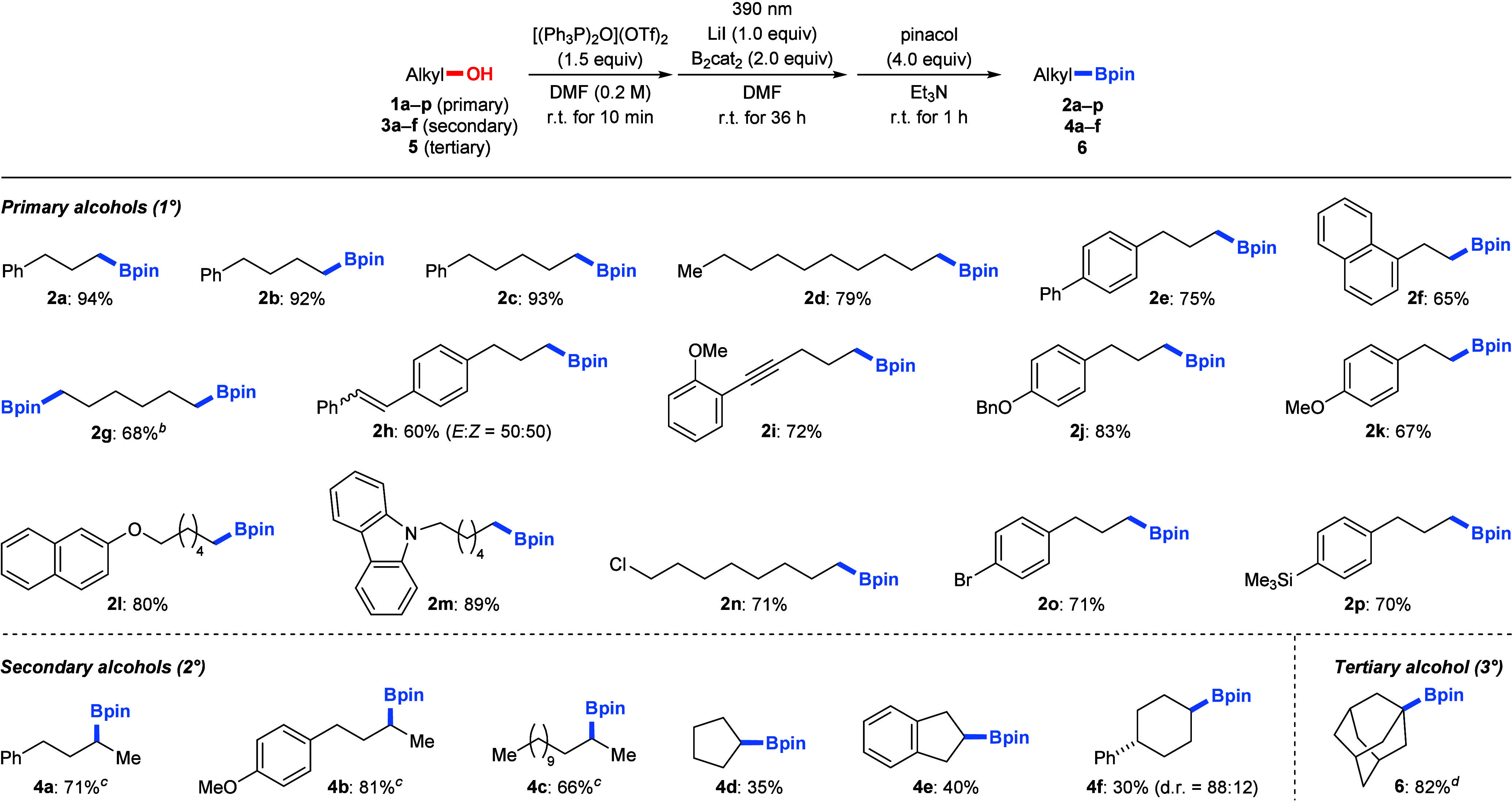
Variation of the Alcohol^a^

a Reaction conditions: The indicated alcohol (0.20 mmol), POP (1.5 equiv), and DMF (1.0 mL) were mixed and stirred at room temperature for 10 min; B_2_cat_2_ (2.0 equiv) and LiI (1.0 equiv) were subsequently added, and then the reaction was irradiated with an LED at 390 nm at room temperature for 36 h. The reaction was terminated by the addition of pinacol (4.0 equiv) and Et_3_N (0.70 mL), and the resulting solution was maintained at room temperature for 1 h. Yields are isolated after purification by flash chromatography on silica gel.
b Using a 2-fold excess of reagents.
c DMA (1.0 mL) was used.
d NMP (1.0 mL) was used.

To showcase the synthetic potential of this two-step/one-pot deoxygenative boration, we tested structurally more complex, pharmaceutically relevant alcohols (Scheme 3). (−)-β-Citronellol (**7a**) and alcohol derivatives of ibuprofen (**7b**), estrone (**7c**), naproxen (**7d**), and α-tocopherol (**7e**) reacted efficiently to deliver desired borylated products **8a**–**e** in moderate to good yields.

**Scheme 3:**
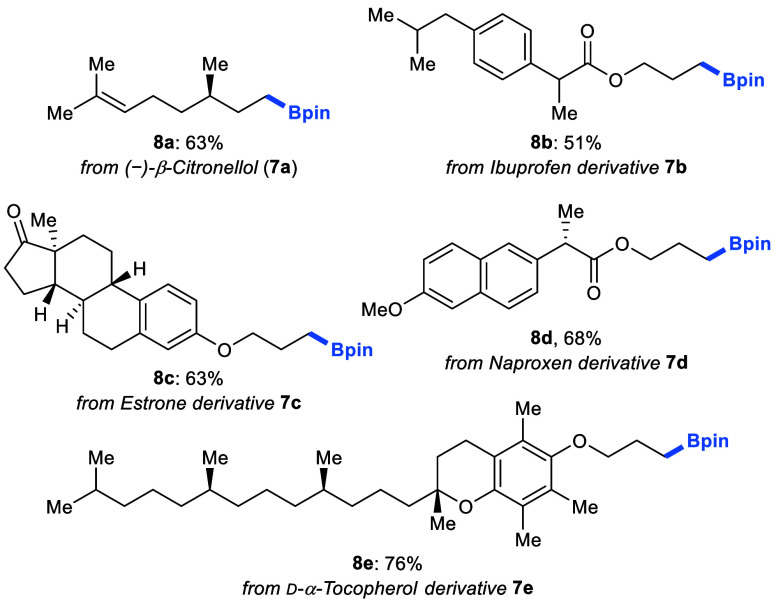
Late-Stage Boration of Structurally Complex Molecules^a^

a See Scheme 2 for the reaction conditions.

The utility of this deoxyboration was further demonstrated by a gram-scale experiment (Scheme 4, top). For the β-citronellol-derived Bpin ester **8a**, the synthetic applicability was illustrated (Scheme 4, bottom). We examined several cross-coupling reactions using a diverse set of electrophiles. The deborative arylation of **8a** with stepwise addition of thiophen-2-yllithium and *N*-bromosuccinimide yielded the product **9a** in good yield.^10^ Various palladium-catalyzed Suzuki–Miyaura cross-couplings with three different (hetero)­aryl halides afforded the arylated products **9b**–**d** in good yields.^11^

**Scheme 4:**
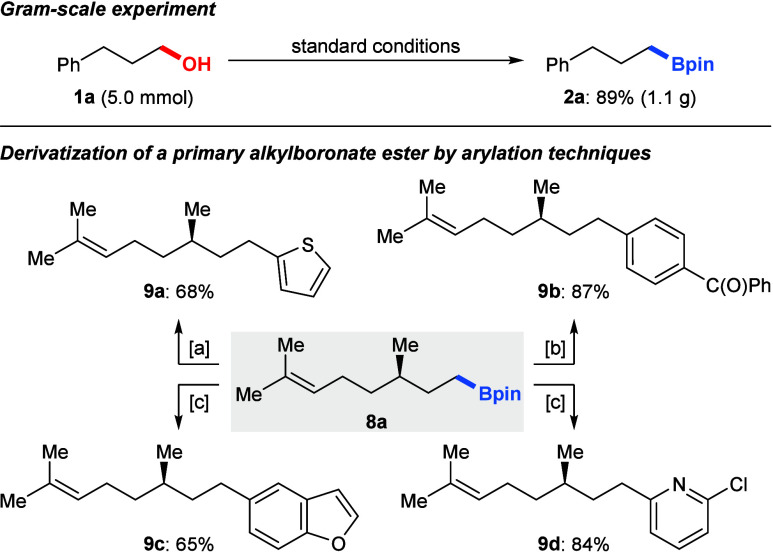
Gram-Scale Experiment and Derivatizations^a^

a Reaction conditions for the derivatizations: All reactions were performed on a 0.20 mmol scale, and isolated yields were determined after purification by flash chromatography on silica gel. [a] thiophene (4.2 equiv), *n*-BuLi (3.8 equiv), THF (3.0 mL), −78 °C to rt, 1 h then boronate **8a** (0.20 mmol), THF (3.5 mL), −78 °C, 1 h. *N*-bromosuccinimide (4.5 equiv), – 78 °C, 1 h. [b] Pd_2_(dba)_3_ (2.0 mol %), Ruphos (4.0 mol %), 4-bromobenzophenone (1.2 equiv), NaO*t*Bu (3.0 equiv), THF (0.50 mL) and H_2_O (50 μL), 80 °C, 24 h. [c] Pd_2_(dba)_3_ (1.0 mol %), *p*-MeOC_6_H_4_PPh_2_ (3.0 mol %), **8a** (2.3 equiv), 5-bromobenzofuran or 2,6-dichloropyridine (0.20 mmol), K_3_PO_4_ (6.0 equiv), 1,4-dioxane (1.0 mL), H_2_O (0.50 mL), 100 °C, 18 h.

The mechanism of this two-step/one-pot reaction sequence is understood on the basis of Studer’s mechanistic analysis.^4f^ After in-situ formation of the alkyl iodide mediated by the Hendrickson reagent (verified by an experiment in the absence of B_2_cat_2_ where 89% isolated yield were obtained), an alkyl radical is generated by purple LED irradiation at 390 nm. The subsequent chain reaction specifically requires B_2_cat_2_ and an amide solvent to stabilize the intermediate boryl radical to eventually generate the alkylboronate ester. Any byproducts introduced by converting the alcohol to the alkyl iodide do not interfere with the radical process.

In conclusion, we established a practical deoxygenative boration strategy that enables access to a wide range of alkylboronates. The key to this transformation lies in the compatibility of the Hendrickson reagent-mediated deoxygenative iodination and Studer’s photoinduced deiodinative boration process. The method exhibits excellent functional-group tolerance and is applicable to a broad range of alcohols.

## Supplementary Material



## Data Availability

The data underlying this study are available in the published article and its Supporting Information.

## Notes

Screening data, general procedures, experimental details, and spectral data for all new compounds (PDF)
