# Supplementary material for: Photoinduced Deoxygenative Boration of Unactivated Alcohols Involving In-Situ-Formed Alkyl Iodides
Source: Org Lett. 2025 Nov 11;27(46):12875–9. doi: 10.1021/acs.orglett.5c04280 (PMC12645580; doi:10.1021/acs.orglett.5c04280)
Supplement: Supplementary file 1 [file ol5c04280_si_001.pdf]

Photoinduced Deoxygenative Boration of Unactivated Alcohols Involving In-Situ-Formed Alkyl Iodides

Xiaojie Liu, Biping Xu, and Martin Oestreich*

Institut für Chemie, Technische Universität Berlin

Straße des 17. Juni 115, 10623 Berlin, Germany

martin.oestreich@tu-berlin.de

Supporting Information

Table of Contents

1 General Information S3

2 Optimization Study S5

2.1 Optimization of reaction parameters S5

2.2 General procedure for deoxygenative boration of alcohols S12

3 Syntheses of Starting Materials S13

4 Characterization Data of Products S15

4.1 Characterization data of starting materials S15

4.2 Characterization data of deoxygenative boration products S17

5 NMR Spectra of Starting Materials and Products S42

6 References S146

1 General Information

All reactions were performed in flame-dried glassware using conventional Schlenk techniques under a static pressure of nitrogen unless stated otherwise. Liquids and solutions were transferred with syringes. Toluene was distilled from metal Na under nitrogen following standard procedures; CH_2Cl_2, DMA, DMF, NMP, MeCN and 1,2-dichlorobenzene were distilled from CaH_2 under nitrogen following standard procedures; DCE was distilled from P_2O_5 under nitrogen following standard procedures. Technical grade solvents for extraction or chromatography (*n*-hexane, *n*-pentane, ethyl acetate, CH_2Cl_2) were distilled prior to use. The Kessil LED lamps (18 W, 456 nm and 40 W, 390nm) was purchased from the manufacturer. All chemicals in this reaction were purchased from Sigma Aldrich, TCI, ABCR, Fisher, Strem and BLD and used as received unless otherwise noted. Analytical thin layer chromatography (TLC) was performed on ALUGRAM® Xtra SIL G/UV254 TLC-Sheets by Macherey-Nagel. Product spots were visualized under UV light (λmax = 254 nm) and with $KMnO_4$ stain. Flash column chromatography was performed on silica gel 60 (40-63 µm, 230-400 mesh, ASTM) by Grace using the indicated solvents. ^{1}H, ^{13}C, and ^{29}Si, ^{11}B NMR spectra were recorded in $CDCl_3$ on Bruker AV400 or AV500 instruments. Chemical shifts were reported in parts per million (ppm) and were referenced to the residual solvent resonance as the internal standard ($CHCl_3$: δ = 7.26 ppm for ^{1}H NMR and $CDCl_3$: δ = 77.00 ppm for ^{13}C NMR). All other nuclei (^{11}B and ^{29}Si) were referenced in compliance with the unified scale for NMR chemical shifts as recommended by the IUPAC stating the chemical shift relative to $BF_3·Et_2O$, CCl_3F, and Me_4Si. Data were reported as follows: chemical shift, multiplicity (br = broad signal, s = singlet, d = doublet, t = triplet, q = quartet, sept = septet, m = multiplet), coupling constants (Hz), and integration. Gas liquid chromatography (GLC) was performed on an *Agilent Technologies 7820A* gas chromatograph equipped with a HP-5 capillary column (30 m × 0.32 mm, 0.25 µm film thickness) by *Agilent Technologies/CS-Chromatographie Service* using the following program: N_2 carrier gas, injection temperature 250 °C, detector temperature 300 °C, flow rate: 1.7 mL/min; temperature program: start temperature 40 °C, heating rate 10 °C/min, end temperature 280 °C for 10 min. Gas-liquid chromatography mass spectrometry (GLC–MS) measurements were conducted on an Agilent Technologies 5975C TAD–GC/MSD-System with electron impact ionization (EI) connected to a fused silica HP-5ms capillary column (length: 30 m, inner diameter: 0.25 mm, thickness of the stationary phase: 0.25 µm). Measurements were performed using the following protocol: Carrier gas: He, injector temperature: 280 °C, detector temperature: 280 °C, flow rate: 4 mL/min, temperature program: starting temperature: 40 °C, heating rate: 10 °C/min, final temperature: 280 °C for 10 min. Infrared (IR) spectra were recorded on an *Agilent Technologies Cary* 630 FT-IR spectrometer equipped with an ATR unit and the signals were reported in wave-numbers (cm^{-1}). Melting points (M.p.) were determined with a Stuart Scientific SMP20 melting point apparatus and were not corrected. High resolution

mass spectra (HRMS) were obtained from the Analytical Facility at the *Institut für Chemie, Technische Universität Berlin* on a Thermo Fisher Scientific LTQ Orbitrap XL apparatus using APCI techniques with a linear ion trap analyzer.

2 Optimization Study

2.1 Optimization of reaction parameters

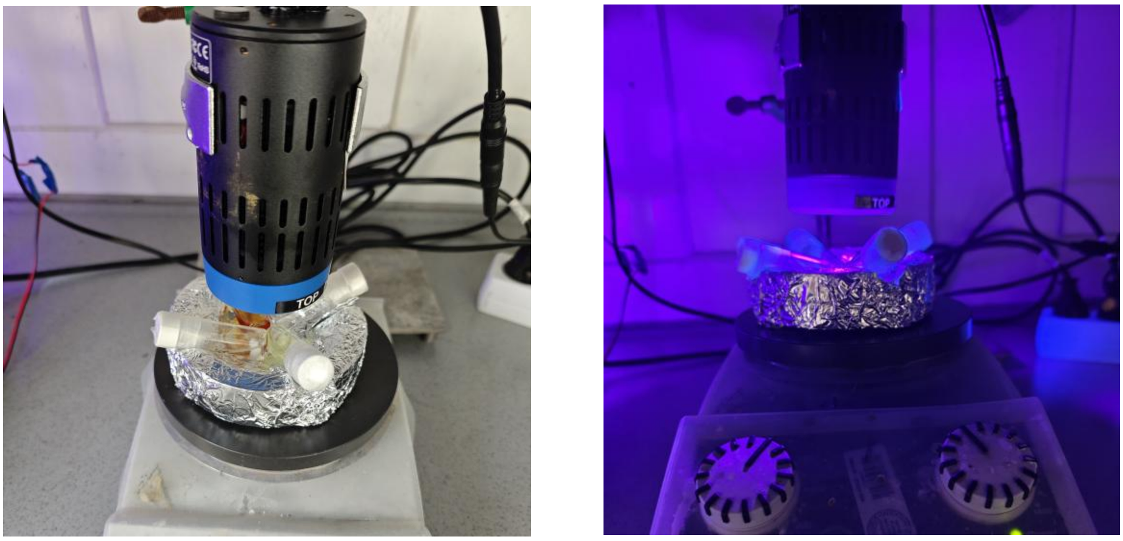

Figure S1. Typical reaction setup at room temperature.

Table S1. Screening of alcohol activation reagents[a]

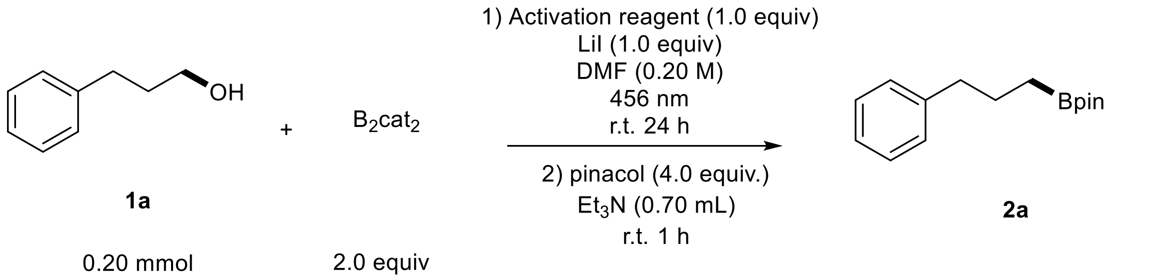

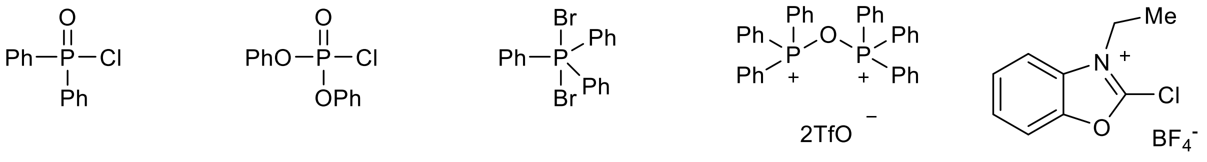

Ph₂POCl **(PhO)₂POCl** **Ph₃PBr₂** **POP** **CEBO**

entry	alcohol activation reagent	yield of **2a** (%)[b]
1	Ph_2POCl	ND
2	$(PhO)_2POCl$	ND
3	Ph_3PBr_2	ND
4	POP	48
5	CEBO	21

[a]All reactions were performed on a 0.20 mmol scale. [b]Yield was determined by GLC analysis with methyl benzoate as an internal standard. ND = Not detected.

Table S2. Screening of the wavelength of the light source[a]

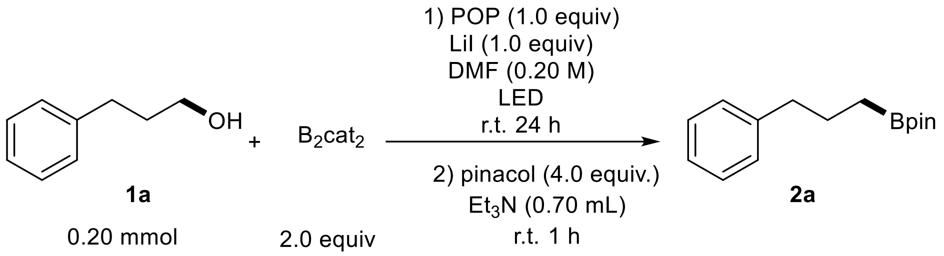

entry	Light wave length	yield of **2a** (%)[b]
1	456 nm	48
2	390 nm	68

[a]All reactions were performed on a 0.20 mmol scale. [b]Yield was determined by GLC analysis with methyl benzoate as an internal standard. N.D. = Not detected.

Table S3. Screening of solvents[a]

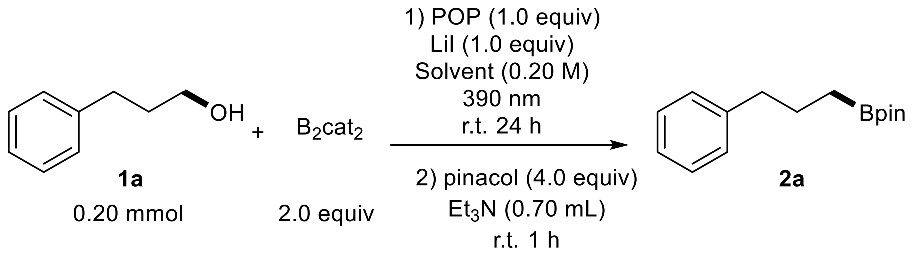

entry	solvent	yield of **2a** (%)[b]
1	DMF	68
2	DMA	37
3	NMP	50
4	1,2-Dichlorobenzene	ND
5	CH_2Cl_2	ND
6	DCE	ND
7	MeCN	ND

[a]All reactions were performed on a 0.20 mmol scale. [b]Yield was determined by GLC analysis with methyl benzoate as an internal standard. DMF = *N*,*N*-dimethylformamide, DMA = *N*, *N*-dimethylacetamide, NMP = *N*-methyl-2-pyrrolidone, DCE = 1,2-dichloroethane.

Table S4. Screening of additives[a]

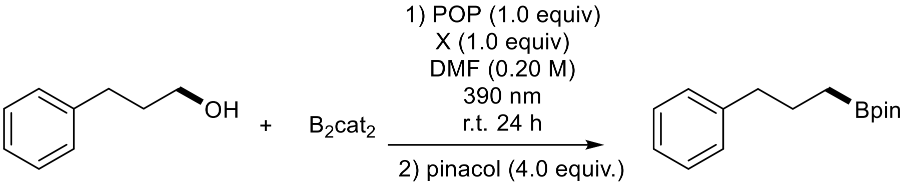

1a 0.20 mmol 2.0 equiv **2a**

entry	Additives	yield of **2a** (%)[b]
1	LiI	68
2	NaI	55
3	KI	41
4	CsI	61
5	TBAI	32
6	LiBr	6
7	NaBr	4
8	KBr	ND
9	TBAB	ND
10[c]	LiI	65
11[d]	LiI	60

[a]All reactions were performed on a 0.20 mmol scale. [b]Yield was determined by GLC analysis with methyl benzoate as an internal standard. TBAI = Tetrabutylammonium iodide, TBAB = Tetrabutylammonium bromide. [c]DMAP (1.0 equiv) was added in standard condition, DMAP = 4-dimethylaminopyridine. [d]Barton's base (1.0 equiv) was added in standard condition.

Table S5. Screening of the equivalent of reactant[a]

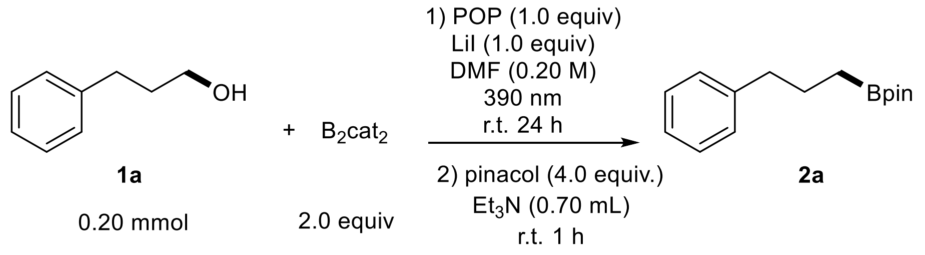

entry	Equivalent of reactant	yield of **2a** (%)[b]
1	None	68
2	POP (1.5 equiv)	86
3	LiI (2.0 equiv)	31
4	LiI (0.5 equiv)	37
5	B_2cat_2 (4.0 equiv)	45
6	DMF (0.10M)	65
7	DMF (0.40 M)	64
8	No pre-stir	42
9[c]	36 h instead of 24 h	98
10[c]	48 h instead of 24 h	96

[a]All reactions were performed on a 0.20 mmol scale. [b]Yield was determined by GLC analysis with methyl benzoate as an internal standard. [c]POP (1.5 equiv) was used.

Table S6. Control experiments[a]

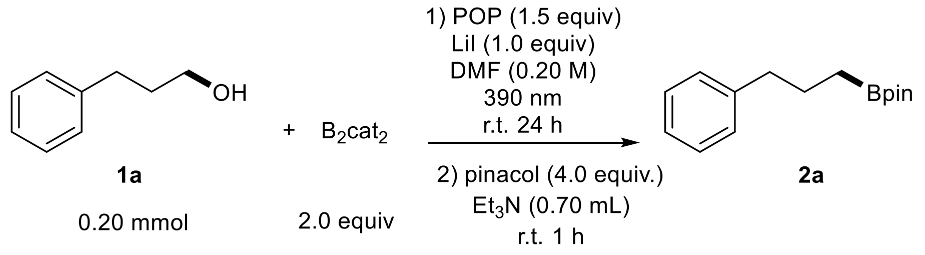

entry	Deviation from standard conditions	yield of **2a** (%)[b]
1	None	98
2	No POP	ND
3	No LiI	6
4	In the dark	14
5	100 °C in the dark	15
6	B_2pin_2 instead of B_2cat_2	N.D.

[a]All reactions were performed on a 0.20 mmol scale. [b]Yield was determined by GLC analysis with methyl benzoate as an internal standard, B_2pin_2 = Bis (pinacolato) diboron.

2.2 General procedure (GP) for deoxygenative boration of alcohols

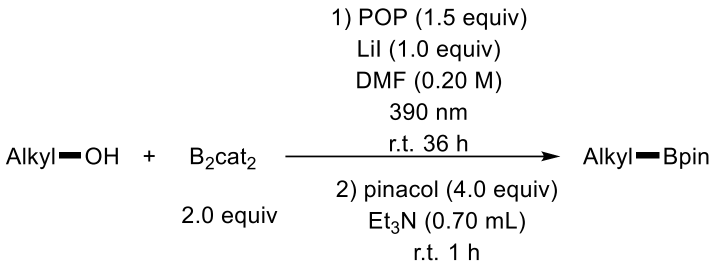

Inside the glove box, to an 8-mL scintillation vial equipped with a magnetic stirring bar are added the alcohols (0.20 mmol), POP (252 mg, 0.30 mmol, 1.5 equiv), followed by the addition of DMF (1.0 mL, 0.20 M) for 10 min at room temperature. Then B_2cat_2 (95 mg, 0.40 mmol, 2.0 equiv), LiI (27 mg, 0.20 mmol, 1.0 equiv) were added to the scintillation vial. After that the vial is then sealed and placed at a distance (app. 5.0 cm) from purple LED Kessil lamp (40 W, 390 nm) outside of the glove box (Figure S1), and the mixture is stirred at room temperature for 36 hours. After the indicated reaction time, add pinacol (95 mg, 0.80 mmol, 4.0 equiv), triethylamine (0.70 mL) to the scintillation vial for another 1h at room temperature. Then the mixture was diluted with ethyl acetate (10 mL) and washed with water (5.0 mL) twice. The organic phase was then dried over anhydrous Na_2SO_4, filtered, and concentrated *in vacuo*. The residue was dissolved with 1.0 mL ethyl acetate and subjected to GLC analysis with methyl benzoate as the internal standard.

3 Syntheses of Starting Materials

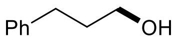

1a

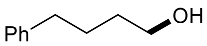

1b

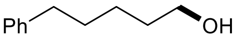

1c

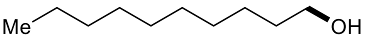

1d

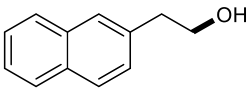

1f

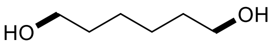

1g

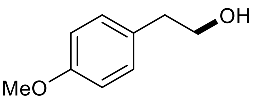

1k

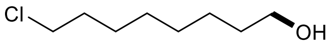

1n

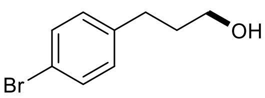

1o

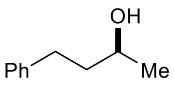

3a

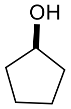

3d

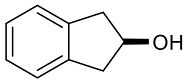

3e

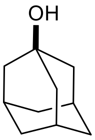

5

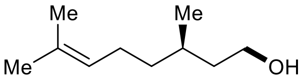

7a

All starting materials shown in the figure above are commercially available and used directly without further purification.

For POP and starting materials **1e, 1h, 1i, 1l, 1m, 1p, 7b, 7c, 7d** and **7e**, the preparation details has been provided in our previous paper[S1].

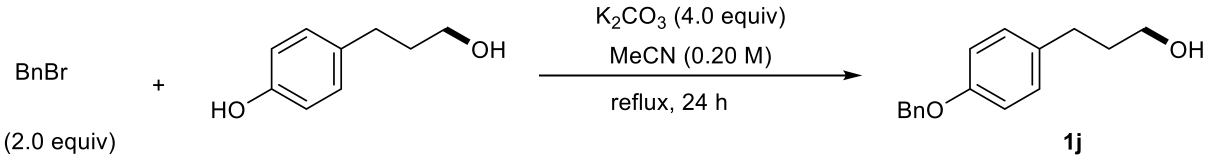

1j

To a 100 mL nitrogen filled round-bottom flask equipped with a Teflon-coated magnetic stir-bar was added 4-(3-hydroxypropyl) phenol (1.5 g, 10 mmol), benzyl bromide (3.4 g, 20 mmol, 2.0 equiv) and K_2CO_3 (5.5 g, 40 mmol, 4.0 equiv), then MeCN (50 mL) was added *via* syringe before the reaction mixture was allowed to heat to reflux in an oil bath for 24 hours. After the indicated reaction time, the reaction mixture was cooled to room temperature, filtered through a pad of silica gel and washed with ethyl acetate. The filtrate was concentrated under reduced pressure and the residue was purified by flash chromatography on silica gel (eluent = *n*-pentane:ethyl acetate = 5:1) to obtain the product **1j**.

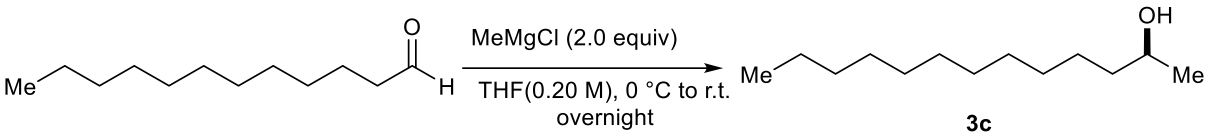

3c

In a nitrogen-filled Schlenk tube with a rubber septum was charged with tridecan-2-one (2.0 g, 10 mmol) and THF 50 mL. After cooling to 0 °C with ice bath, 20 mL of methylmagnesium chloride (1.0 M) was dropwise added to the solution via syringe through a rubber septum over a period of 2 hours, then the reaction mixture was stirred at room temperature for 3 hours. The reaction mixture was quenched saturated NH_4Cl solution (10 mL) and extracted with ethyl acetate (50 mL) twice. Then the combined organic layer was dried with anhydrous Na_2SO_4, followed by evaporation under reduced pressure to remove the solvent. The residue was purified by flash chromatography on silica gel (eluent = *n*-pentane:ethyl acetate = 20:1) to obtain **3c** as colorless oil.

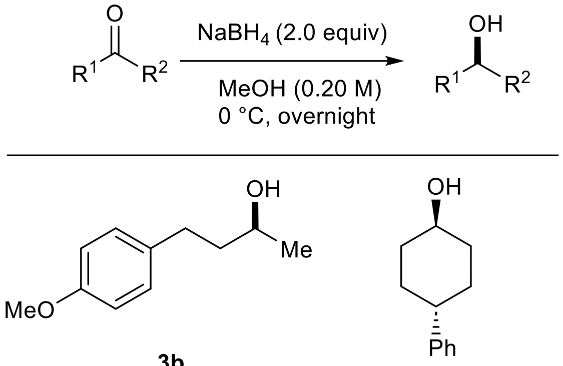

3b **3f**

To a 100 mL flame-dried round-bottom Schlenk flask charged with a Teflon-coated magnetic stir-bar and ketone (10 mmol) was added dry MeOH 50 mL. When the solution was cooled to 0 °C with ice bath, sodium borohydride (0.76 g, 20 mmol, 2.0 equiv) was portionwise added over 15 minutes under a nitrogen flow. Upon finishing addition of sodium borohydride, the reaction was stired overnight. After the reaction was cooled to room temperature, it was quenched with saturated NH_4Cl solution (10 mL) and extracted with CH_2Cl_2 (50 mL) twice. Then the combined organic layer was dried with anhydrous Na_2SO_4, followed by evaporation under reduced pressure. The residue was purified by flash chromatography on silica gel (eluent = *n*-pentane:ethyl acetate = 5:1) to obtain the corresponding alcohols.

4 Characterization Data of Products

4.1 Characterization data of starting materials

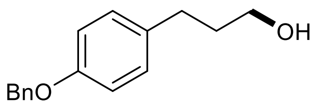

1j
$C_{16}H_{18}O_2$
M = 242.32 g/mol

3-(4-(Benzyloxy) phenyl) propan-1-ol (1j): Prepared from 4-(3-hydroxypropyl) phenol (1.5 g, 10 mmol) and benzyl bromide (3.2 g, 20 mmol, 2.0 equiv) according to the method mentioned in **Section 3**. Purification by flash column chromatography on silica gel using *n*-pentane:ethyl acetate = 5:1 afforded **1j** as a white solid (2.2 g, 90% yield).

$\mathbf{R_f}$ = 0.40 (*n*-pentane: ethyl acetate = 2:1)
M.p.: 66–68 °C.
^{1}H NMR (400 MHz, $CDCl_3$) δ 7.44 (d, *J* = 7.6 Hz, 2H), 7.40–7.37 (m, 2H), 7.34–7.31 (m, 1H), 7.12 (d, *J* = 6.8 Hz, 2H), 6.91 (d, *J* = 6.8 Hz, 2H), 5.05 (s, 2H), 3.68 (t, *J* = 6.5 Hz, 2H), 2.66 (t, *J* = 7.9 Hz, 2H), 1.89–1.84 (m, 2H), 1.42 (s, 1H) ppm.
^{13}C NMR (101 MHz, $CDCl_3$) δ 157.1, 137.2, 134.1, 129.3, 128.5, 127.9, 127.4, 114.8, 70.1, 62.3, 34.4, 31.1 ppm.

IR (ATR): ṽ/cm^{-1} = 3301, 3277, 3030, 2920, 2859, 2342, 2120, 2090, 1881, 1750, 1606, 1506, 1451, 1378, 1296, 1228, 1173, 1045, 1001, 908, 808, 735, 693.

HRMS (APCI) for $C_{16}H_{19}O_2^+$ $[M+H]^+$: calculated 243.1380, found 243.1377.

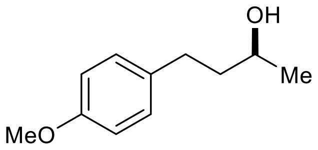

3b
$C_{11}H_{16}O_2$
M = 180.25 g/mol

4-(4-Methoxyphenyl) butan-2-ol (3b): Prepared from 4-(4-methoxyphenyl) butan-2-one (1.8 g, 10 mmol) and sodium borohydride (0.76 g, 20 mmol, 2.0 equiv) according to the method mentioned in **Section 3**. Purification by flash column chromatography on silica gel using *n*-pentane:ethyl acetate = 5:1 afforded **3b** as a colorless oil (1.6 g, 90% yield).

$\mathbf{R_f}$ = 0.40 (*n*-pentane: ethyl acetate = 2:1)

^{1}H NMR (500 MHz, $CDCl_3$) δ 7.12 (d, *J* = 8.3 Hz, 2H), 6.84 (d, *J* = 8.0 Hz, 2H), 3.85–3.79 (m, 4H), 2.73–2.59 (m, 2H), 1.79–1.71 (m, 3H), 1.22 (d, *J* = 6.3 Hz, 3H) ppm.

^{13}C NMR (101 MHz, $CDCl_3$) δ 157.7, 134.0, 129.2, 113.7, 67.3, 55.2, 41.0, 31.1, 23.5 ppm.

IR (ATR): ṽ/cm^{-1} = 3352, 2927, 2340, 2107, 1876, 1611, 1510, 1458, 1372, 1298, 1240, 1176, 1125, 1063, 1033, 953, 823, 743, 701.

HRMS (APCI) for $C_{11}H_{15}O^+$ [M–OH]$^+$: calculated 163.1117, found 163.1119.

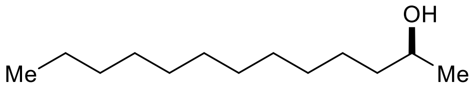

3c
$C_{13}H_{28}O$
M = 200.37 g/mol

Tridecan-2-ol (3c): Prepared from tridecan-2-one (2.0 g, 10 mmol) and methylmagnesium chloride (1.0 M) (20 mL, 20 mmol, 2.0 equiv) according to the Method mentioned in **Section 3**. Purification by flash column chromatography on silica gel using *n*-pentane:ethyl acetate = 5:1 afforded **3c** as a colorless oil (1.7 g, 84% yield).

R_f = 0.70 (*n*-pentane: ethyl acetate = 4:1)

^{1}H NMR (400 MHz, $CDCl_3$) δ 3.80–3.73 (m, 1H), 1.59 (s, 1H), 1.44–1.25 (m, 20H), 1.16 (d, *J* = 6.2 Hz, 3H), 0.88–0.85 (m, 3H) ppm.

^{13}C NMR (101 MHz, $CDCl_3$) δ 68.1, 39.3, 31.9, 29.6, 29.6, 29.6, 29.3, 25.8, 23.4, 22.6, 14.1 ppm.

IR (ATR): ṽ/cm^{-1} = 3339, 2920, 2852, 2676, 2110, 1651, 1462, 1374, 1304, 1116, 1042, 939, 842, 720.

HRMS (APCI) for $C_{13}H_{27}$ [M–OH]$^+$: calculated 183.2107, found 183.2104.

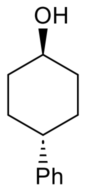

3f
$C_{12}H_{16}O$
M = 176.26 g/mol

4-Phenylcyclohexan-1-ol (3f): Prepared from 4-phenylcyclohexanone (1.7 g, 10 mmol) and sodium borohydride (0.76 g, 20 mmol, 2.0 equiv) according to the method mentioned in **Section 3**. Purification by flash column chromatography on silica gel using *n*-pentane:ethyl acetate = 5:1 afforded **3f** as a white solid (1.5 g, 85% yield).

R_f = 0.40 (*n*-pentane: ethyl acetate = 2:1)

M.p.: 120.5–122.5 °C.

^{1}H NMR (400 MHz, $CDCl_3$) δ 7.32–7.28 (m, 2H), 7.22–7.17 (m, 3H), 3.73–3.66 (m, 1H), 2.54–2.47 (m, 1H), 2.12–2.09 (m, 2H), 1.96–1.92 (m, 2H), 1.60–1.39 (m, 5H) ppm.

^{13}C NMR (101 MHz, $CDCl_3$) δ 146.5, 128.3, 126.8, 126.0, 70.6, 43.4, 35.9, 32.4 ppm.

IR (ATR): ṽ/cm^{-1} = 3419, 3369, 3027, 2919, 2851, 2341, 2079, 1797, 1598, 1491, 1449, 1343, 1311, 1197, 1148, 1115, 1058, 965, 887, 840, 753, 694.

HRMS (APCI) for $C_{12}H_{15}^+$ $[M–OH]^+$: calculated 159.1168, found 159.1165.

4.2 Characterization data of deoxygenative boration products

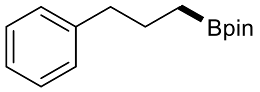

2a
$C_{15}H_{23}BO_2$
M = 246.16 g/mol

4,4,5,5-Tetramethyl-2-(3-phenylpropyl)-1,3,2-dioxaborolane (2a): Prepared from 3-phenylpropan-1-ol (27 mg, 0.20 mmol) and B_2cat_2 (95 mg, 0.40 mmol) according to **GP**. Purification by flash column chromatography on silica gel using *n*-pentane:ethyl acetate = 99:1 afforded **2a** as a colorless oil (46 mg, 94% yield).

R_f = 0.75 (*n*-pentane: ethyl acetate = 90:10).

^{1}**H NMR** (400 MHz, $CDCl_3$) δ 7.30 (t, *J* = 7.4 Hz, 2H), 7.22–7.18(m, 3H), 2.65 (t, *J* = 7.8 Hz, 2H), 1.81–1.70 (m, 2H), 1.28 (s, 12H), 0.87 (t, *J* = 7.9 Hz, 2H) ppm.

^{13}**C NMR** (101 MHz, $CDCl_3$) δ 142.6, 128.5, 128.1, 125.5, 82.9, 38.5, 26.1, 24.8 ppm. (The carbon atom attached to the boron atom was not observed due to quadrupolar relaxation.)

^{11}**B NMR** (128 MHz, $CDCl_3$) δ 34.0(s) ppm.

IR (ATR): ṽ/cm^{-1} = 2977, 2929, 2089, 1602, 1453, 1369, 1315, 1268, 1225, 1142, 967, 846, 743, 697.

HRMS (APCI) for $C_{15}H_{24}BO_2^+$ [M+H]$^+$: calculated 247.1864, found 247.1863.

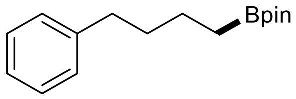

2b
$C_{16}H_{25}BO_2$
M = 260.18 g/mol

4,4,5,5-Tetramethyl-2-(4-phenylbutyl)-1,3,2-dioxaborolane (2b): Prepared from 4-phenylbutan-1-ol (30 mg, 0.20 mmol) and B_2cat_2 (95 mg, 0.40 mmol) according to **GP**. Purification by flash column chromatography on silica gel using *n*-pentane:ethyl acetate = 99:1 afforded **2b** as a colorless oil (48 mg, 92% yield).

R_f = 0.75 (*n*-pentane: ethyl acetate = 90:10).

^{1}**H NMR** (400 MHz, $CDCl_3$) δ 7.17 (t, *J* = 7.5 Hz, 2H), 7.09–7.05 (m, 3H), 2.51 (t, *J* = 7.8 Hz, 2H), 1.58–1.50 (m, 2H), 1.42–1.35 (m, 2H), 1.15 (s, 12H), 0.73 (t, *J* = 7.8 Hz, 2H) ppm.

^{13}**C NMR** (101 MHz, $CDCl_3$) δ 142.9, 128.3, 128.1, 125.5, 82.8, 35.7, 34.2, 24.8, 23.7 ppm. (The carbon atom attached to the boron atom was not observed due to quadrupolar relaxation.)

^{11}**B NMR** (128 MHz, $CDCl_3$) δ 34.0(s) ppm.

IR (ATR): ṽ/cm^{-1} = 2977,2927,1988,1728,1603,1453,1371,1315,1214,1142, 1006, 966, 845, 744, 697.

HRMS (APCI) for $C_{16}H_{26}BO_2^+$ [M+H]$^+$: calculated 261.2020, found 261.2020.

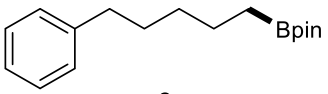

2c
$C_{17}H_{27}BO_2$
M = 274.21 g/mol

4,4,5,5-Tetramethyl-2-(5-phenylpentyl)-1,3,2-dioxaborolane (2c): Prepared from 5-phenylpentan-1-ol (33 mg, 0.20 mmol) and B_2cat_2 (95 mg, 0.40 mmol) according to **GP**. Purification by flash column chromatography on silica gel using *n*-pentane:ethyl acetate = 99:1 afforded **2c** as a colorless oil (51 mg, 93% yield).

R_f = 0.75 (*n*-pentane: ethyl acetate = 90:10).

^{1}H NMR (400 MHz, $CDCl_3$) δ 7.18 (t, *J* = 7.5 Hz, 2H), 7.08 (d, *J* = 7.5 Hz, 3H), 2.52 (t, *J* = 7.8 Hz, 2H), 1.58–1.50 (m, 2H), 1.41–1.33 (m, 2H), 1.31–1.22(m, 2H), 1.15 (s, 12H), 0.70 (t, *J* = 7.7 Hz, 2H) ppm.

^{13}C NMR (101 MHz, $CDCl_3$) δ 142.9, 128.4, 128.1, 125.5, 82.8, 35.8, 32.0, 31.2, 24.8, 23.8 ppm. (The carbon atom attached to the boron atom was not observed due to quadrupolar relaxation.)

^{11}B NMR (128 MHz, $CDCl_3$) δ 34.0(s) ppm.

IR (ATR): ṽ/cm^{-1} = 2977, 2926, 2855, 2114, 1603, 1453, 1371, 1316, 1234, 1143, 1029, 967, 846, 744, 697.

HRMS (APCI) for $C_{17}H_{28}BO_2^+$ $[M+H]^+$: calculated 275.2177, found 275.2178.

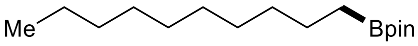

2d
$C_{16}H_{33}BO_2$
M = 268.25 g/mol

2-Decyl-4,4,5,5-tetramethyl-1,3,2-dioxaborolane (2d): Prepared from decan-1-ol (32 mg, 0.20 mmol) and B_2cat_2 (95 mg, 0.40 mmol) according to **GP**. Purification by flash column chromatography on silica gel using *n*-pentane:ethyl acetate = 99:1 afforded **2d** as a colorless oil (42 mg, 79% yield).

R_f = 0.80 (*n*-pentane: ethyl acetate = 90:10).

^{1}H NMR (400 MHz, $CDCl_3$) δ 1.42–1.35 (m, 2H), 1.30–1.23 (m, 26H), 0.86 (t, *J* = 6.9 Hz, 3H), 0.75 (t, *J* = 7.8 Hz, 2H) ppm.

^{13}C NMR (101 MHz, $CDCl_3$) δ 82.8, 32.4, 31.9, 29.6, 29.6, 29.4, 29.3, 24.8, 24.0, 22.7, 14.1 ppm. (The carbon atom attached to the boron atom was not observed due to quadrupolar relaxation.)

^{11}B NMR (128 MHz, $CDCl_3$) δ 34.2(s) ppm.

IR (ATR): ṽ/cm^{-1} = 2922, 2853, 1731, 1464, 1372, 1315, 1214, 1144, 968, 846, 720, 672.

HRMS (APCI) for $C_{16}H_{32}BO_2^+$ [M–H]$^+$: calculated 267.2490, found 267.2492.

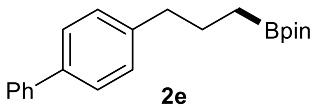

2e

$C_{21}H_{27}BO_2$
M = 322.26 g/mol

2-(3-([1,1'-Biphenyl]-4-yl) propyl)-4,4,5,5-tetramethyl-1,3,2-dioxaborolane (2e): Prepared from **1e** (42 mg, 0.20 mmol) and B_2cat_2 (95 mg, 0.40 mmol) according to **GP**. Purification by flash column chromatography on silica gel using *n*-pentane:ethyl acetate = 99:1 afforded **2e** as a white solid (48 mg, 75% yield).

R_f = 0.75 (*n*-pentane: ethyl acetate = 90:10).

M.p.: 70–72 °C.

^{1}H NMR (500 MHz, $CDCl_3$) δ 7.49 (d, *J* = 6.9 Hz, 2H), 7.42 (d, *J* = 8.1 Hz, 2H), 7.34 (t, J = 7.7 Hz, 2H), 7.23 (t, *J* = 7.3 Hz, 1H), 7.17 (d, J = 7.8 Hz, 2H), 2.59–2.56 (m, 2H), 1.73–1.67(m, 2H), 1.17 (s, 12H), 0.78 (t, *J* = 7.9 Hz, 2H) ppm.

^{13}C NMR (126 MHz, $CDCl_3$) δ 141.9, 141.3, 138.6, 129.0, 128.7, 127.0, 127.0, 126.9, 83.0, 38.2, 26.1, 24.9 ppm. (The carbon atom attached to the boron atom was not observed due to quadrupolar relaxation.)

^{11}B NMR (128 MHz, $CDCl_3$) δ 34.1(s) ppm.

IR (ATR): ṽ/cm^{-1} = 2977, 2927, 2860, 2634, 2110, 2075, 1987, 1905, 1598, 1518, 1484, 1367, 1317, 1264, 1207, 1138, 1007, 969, 887, 844, 767, 733, 701.

HRMS (APCI) for $C_{21}H_{27}BO_2^+$ [M]$^+$: calculated 322.2099, found 322.2097.

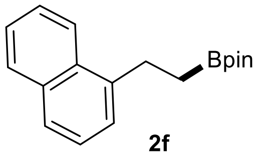

2f
$C_{18}H_{23}BO_2$
M = 282.19 g/mol

4,4,5,5-Tetramethyl-2-(2-(naphthalen-1-yl) ethyl)-1,3,2-dioxaborolane (2f): Prepared from 2-(naphthalen-1-yl) ethan-1-ol (34 mg, 0.20 mmol) and B_2cat_2 (95 mg, 0.40 mmol) according to **GP**. Purification by flash column chromatography on silica gel using *n*-pentane:ethyl acetate = 99:1 afforded **2f** as a colorless oil (37 mg, 65% yield).

R_f = 0.65 (*n*-pentane: ethyl acetate = 90:10).

^{1}H NMR (400 MHz, $CDCl_3$) δ 8.11 (d, *J* = 8.6 Hz, 1H), 7.85 (d, *J* = 6.9 Hz, 1H), 7.72–7.68 (m, 1H), 7.53–7.45 (m, 2H), 7.42–7.39 (m, 2H), 3.25–3.21 (m, 2H), 1.33–1.29 (m, 2H), 1.26 (s, 12H) ppm.

^{13}C NMR (101 MHz, $CDCl_3$) δ 140.4, 133.8, 131.7, 128.6, 126.3, 125.5, 125.5, 125.3, 125.0, 123.9, 83.1, 26.9, 24.8 ppm. (The carbon atom attached to the boron atom was not observed due to quadrupolar relaxation.)

^{11}B NMR (128 MHz, $CDCl_3$) δ 33.9(s) ppm.

IR (ATR): $\tilde{v}$/cm^{-1} =3043, 2976, 2930, 2320, 2105, 1918, 1724, 1596, 1509, 1463, 1366, 1308, 1269, 1246, 1140, 1014, 965, 884, 845, 774, 672.

HRMS (APCI) for $C_{18}H_{23}BO_2^+$ $[M]^+$: calculated 282.1786, found 282.1788.

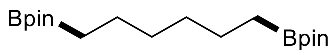

2g
$C_{18}H_{36}B_2O_4$
M = 338.10 g/mol

1,6-Bis(4,4,5,5-tetramethyl-1,3,2-dioxaborolan-2-yl)hexane (2g): Prepared from hexane-1,6-diol (24 mg, 0.20 mmol) and B_2cat_2 (190 mg, 0.80 mmol) according to **GP**. Purification by flash column chromatography on silica gel using *n*-pentane:ethyl acetate = 98:2 afforded **2g** as a white solid (46 mg, 68% yield).

R_f = 0.50 (*n*-pentane: ethyl acetate = 90:10).

M.p.: 45–47 °C.

^{1}H NMR (400 MHz, $CDCl_3$) δ 1.41–1.34 (m, 4H), 1.28–1.24 (m, 4H), 1.22 (s, 24H), 0.74 (t, *J* = 7.7 Hz, 4H) ppm.

^{13}C NMR (101 MHz, $CDCl_3$) δ 82.8, 32.2, 24.8, 23.9 ppm. (The carbon atom attached to the boron atom was not observed due to quadrupolar relaxation.)

^{11}B NMR (128 MHz, $CDCl_3$) δ 34.0(s) ppm.

IR (ATR): ṽ/cm^{-1} = 3731, 2973, 2924, 1963, 1729, 1464, 1371, 1309, 1238, 1141, 967, 918, 882, 845, 761, 714, 674.

HRMS (APCI) for $C_{18}H_{37}B_2O_4^+$ $[M+H]^+$: calculated 339.2872, found 339.2870.

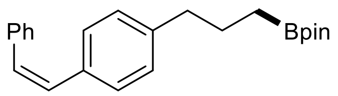

2h (*Z*)
$C_{23}H_{29}BO_2$
M = 348.29 g/mol

(*Z*)-4,4,5,5-Tetramethyl-2-(3-(4-styrylphenyl) propyl)-1,3,2-dioxaborolane (2h (*Z*)): Prepared from **1h** (48 mg, 0.20 mmol) and B_2cat_2 (95 mg, 0.40 mmol) according to **GP**. Purification by flash column chromatography on silica gel using *n*-pentane:ethyl acetate = 99:1 afforded **2h** (*Z*) as a colorless oil (22 mg, 31% yield).

R_f = 0.75 (*n*-pentane: ethyl acetate = 90:10).

^{1}H NMR (400 MHz, $CDCl_3$) δ 7.21–7.11 (m, 5H), 7.08 (d, *J* = 8.1 Hz, 2H), 6.95 (d, *J* = 8.2 Hz, 2H), 6.47 (s, 2H), 2.51–2.47 (m, 2H), 1.67–1.59 (m, 2H), 1.17 (s, 12H), 0.76–0.73 (m, 2H) ppm.

^{13}C NMR (101 MHz, $CDCl_3$) δ 141.7, 137.5, 134.4, 130.2, 129.4, 128.8, 128.7, 128.3, 128.2, 126.9, 82.9, 38.3, 25.9, 24.8 ppm. (The carbon atom attached to the boron atom was not observed due to quadrupolar relaxation.)

^{11}B NMR (128 MHz, $CDCl_3$) δ 33.9(s) ppm.

IR (ATR): ṽ/cm^{-1} = 2976, 2927, 1907, 1733, 1599, 1491, 1459, 1369, 1316, 1267, 1223, 1142, 966, 845, 771, 695.

HRMS (APCI) for $C_{23}H_{29}BO_2^+$ $[M]^+$: calculated 348.2255, found 348.2260.

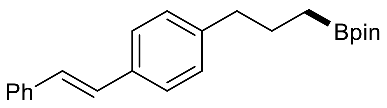

2h (*E*)
$C_{23}H_{29}BO_2$
M = 348.29 g/mol

(*E*)-4,4,5,5-Tetramethyl-2-(3-(4-styrylphenyl) propyl)-1,3,2-dioxaborolane (2h (*E*)): Prepared from **1h** (48 mg, 0.20 mmol) and B_2cat_2 (95 mg, 0.40 mmol) according to **GP**. Purification by flash column chromatography on silica gel using *n*-pentane:ethyl acetate = 99:1 afforded **2h** (*E*) as a white solid (20 mg, 29% yield).

R_f = 0.72 (*n*-pentane: ethyl acetate = 90:10).

M.p.: 96–98 °C.

^{1}H NMR (400 MHz, $CDCl_3$) 7.43 (d, *J* = 7.0 Hz, 2H), 7.35 (d, J = 8.2 Hz, 2H), 7.27 (t, *J* = 7.7 Hz, 2H), 7.18–7.17(m, 1H), 7.09 (d, *J* = 8.0 Hz, 2H), 7.00 (d, *J* = 3.5 Hz, 2H), 2.56–2.52 (m, 2H), 1.70–1.62 (m, 2H), 1.17 (s, 12H), 0.76 (t, *J* = 7.9 Hz, 2H) ppm.

^{13}C NMR (101 MHz, $CDCl_3$) δ 142.4, 137.5, 134.7, 128.9, 128.7, 128.6, 127.6, 127.3, 126.4, 82.9, 38.3, 26.0, 24.8 ppm. (The carbon atom attached to the boron atom was not observed due to quadrupolar relaxation.)

^{11}B NMR (128 MHz, $CDCl_3$) δ 33.7(s) ppm.

IR (ATR): ṽ/cm^{-1} = 2976, 2940, 2620, 2321, 2109, 1908, 1734, 1595, 1511, 1449, 1360, 1321, 1263, 1210, 1139, 1026, 965, 887, 819, 752, 726, 692.

HRMS (APCI) for $C_{23}H_{29}BO_2^+$ [M]$^+$: calculated 348.2255, found 348.2257.

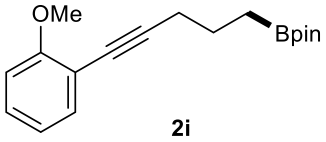

2i
$C_{18}H_{25}BO_3$
M = 300.21 g/mol

2-(5-(2-Methoxyphenyl) pent-4-yn-1-yl)-4,4,5,5-tetramethyl-1,3,2-dioxaborolane (2i): Prepared from **1i** (38 mg, 0.20 mmol) and B_2cat_2 (95 mg, 0.40 mmol) according to **GP**. Purification by flash column chromatography on silica gel using *n*-pentane:ethyl acetate = 98:2 afforded **2i** as a colorless oil (44 mg, 72% yield).

R_f = 0.40 (*n*-pentane: ethyl acetate = 90:10).

^{1}H NMR (400 MHz, $CDCl_3$) δ 7.37 (d, *J* = 7.5 Hz, 1H), 7.22 (t, *J* = 7.0 Hz, 1H), 6.88–6.83 (m, 2H), 3.86 (s, 3H), 2.48 (t, *J* = 7.2 Hz, 2H), 1.79–1.71 (m, 2H), 1.24 (s, 12H), 0.98–0.94 (m, 2H) ppm.

^{13}C NMR (101 MHz, $CDCl_3$) δ 159.7, 133.7, 128.7, 120.3, 113.2, 110.5, 94.6, 83.0, 55.7, 24.8, 23.6, 22.2 ppm. (The carbon atom attached to the boron atom was not observed due to quadrupolar relaxation.)

^{11}B NMR (128 MHz, $CDCl_3$) δ 33.8(s) ppm.

IR (ATR): ṽ/cm^{-1} = 2975, 2932, 2262, 2127, 2087, 1894, 1725, 1595, 1491, 1460, 1370, 1316, 1258, 1141, 1025, 966, 881, 847, 793, 749, 673.

HRMS (APCI) for $C_{18}H_{26}BO_3^+$ [M+H]$^+$: calculated 301.1970, found 301.1969.

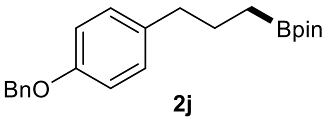

2j
$C_{22}H_{29}BO_3$
M = 352.28 g/mol

2-(3-(4-(Benzyloxy) phenyl) propyl)-4,4,5,5-tetramethyl-1,3,2-dioxaborolane (2j): Prepared from **1j** (48 mg, 0.20 mmol) and B_2cat_2 (95 mg, 0.40 mmol) according to **GP**. Purification by flash column chromatography on silica gel using *n*-pentane:ethyl acetate = 99:1 afforded **2j** as a white solid (58 mg, 83% yield).

R_f = 0.70 (*n*-pentane: ethyl acetate = 90:10).

M.p.: 72–74 °C.

^{1}H NMR (500 MHz, $CDCl_3$) δ 7.44 (d, *J* = 8.1 Hz, 2H), 7.40–7.37 (m, 2H), 7.33–7.31 (m, 1H), 7.10 (d, *J* = 7.9 Hz, 2H), 6.89 (d, *J* = 8.7 Hz, 2H), 5.04 (s, 2H), 2.56 (t, *J* = 7.7 Hz, 2H), 1.73–1.67 (m, 2H), 1.25 (s, 12H), 0.82 (t, *J* = 7.9 Hz, 2H) ppm.

^{13}C NMR (126 MHz, $CDCl_3$) δ 156.9, 137.3, 135.1, 129.4, 128.5, 127.8, 127.4, 114.6, 82.9, 70.1, 37.7, 26.2, 24.8 ppm. (The carbon atom attached to the boron atom was not observed due to quadrupolar relaxation.)

^{11}B NMR (128 MHz, $CDCl_3$) δ 33.9(s) ppm.

IR (ATR): ṽ/cm^{-1} = 2985, 2934, 2876, 616, 2102, 1993, 1733, 1607, 1511, 1455, 1367, 1313, 1234, 1165, 1140, 1009, 966, 916, 885, 847, 808, 743, 696, 717, 696.

HRMS (APCI) for $C_{22}H_{29}BO_3^+$ [M]$^+$: calculated 352.2204, found 352.2206.

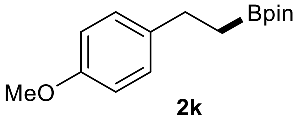

2k
$C_{15}H_{23}BO_3$
M = 262.16 g/mol

2-(4-Methoxyphenethyl)-4,4,5,5-tetramethyl-1,3,2-dioxaborolane (2k): Prepared from 2-(4-methoxyphenyl) ethan-1-ol (30 mg, 0.20 mmol) and B_2cat_2 (95 mg, 0.40 mmol) according to **GP**. Purification by flash column chromatography on silica gel using *n*-pentane:ethyl acetate = 98:2 afforded **2k** as a colorless oil (35 mg, 67% yield).

R$_f$ = 0.60 (*n*-pentane: ethyl acetate = 90:10).

^{1}H NMR (400 MHz, $CDCl_3$) δ 7.13 (d, *J* = 8.7 Hz, 2H), 6.81 (d, *J* = 8.6 Hz, 2H), 3.78 (s, 3H), 2.71–2.67 (m, 2H), 1.22 (s, 12H), 1.13–1.09 (m, 2H) ppm.

^{13}C NMR (101 MHz, $CDCl_3$) δ 157.5, 136.5, 128.8, 113.6, 83.0, 55.2, 29.0, 24.8 ppm. (The carbon atom attached to the boron atom was not observed due to quadrupolar relaxation.)

^{11}B NMR (128 MHz, $CDCl_3$) δ 33.8(s) ppm.

IR (ATR): ṽ/cm^{-1} = 2977, 2932, 2324, 2120, 2059, 1996, 1876, 1726, 1611, 1510, 1464, 1368, 1317, 1241, 1176, 1141, 1036, 966, 879, 828, 757, 700, 672.

HRMS (APCI) for $C_{15}H_{23}BO_3^+$ [M]$^+$: calculated 262.1735, found 262.1736.

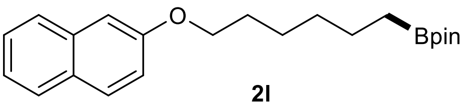

2l
$C_{22}H_{31}BO_3$
M = 354.30 g/mol

4,4,5,5-Tetramethyl-2-(6-(naphthalen-2-yloxy) hexyl)-1,3,2-dioxaborolane (2l): Prepared from **1l** (49 mg, 0.20 mmol) and B_2cat_2 (95 mg, 0.40 mmol) according to **GP**. Purification by flash column chromatography on silica gel using *n*-pentane:ethyl acetate = 99:1 afforded **2l** as a colorless oil (57 mg, 80% yield).

R$_f$ = 0.70 (*n*-pentane: ethyl acetate = 90:10).

^{1}H NMR (400 MHz, $CDCl_3$) δ 7.77–7.71 (m, 3H), 7.43 (t, *J* = 7.5 Hz, 1H), 7.32 (t, *J* = 6.9 Hz, 1H), 7.17–7.12 (m, 2H), 4.07 (t, *J* = 6.6 Hz, 2H), 1.89–1.82 (m, 2H), 1.55–1.38 (m, 6H), 1.25 (s, 12H), 0.82 (t, *J* = 7.6 Hz, 2H) ppm.

^{13}C NMR (101 MHz, $CDCl_3$) δ 157.1, 134.6, 129.2, 128.8, 127.6, 126.6, 126.2, 123.4, 119.0, 106.5, 82.8, 67.9, 32.1, 29.1, 25.8, 24.8, 23.9 ppm. (The carbon atom attached to the boron atom was not observed due to quadrupolar relaxation.)

^{11}B NMR (128 MHz, $CDCl_3$) δ 34.2(s) ppm.

IR (ATR): ṽ/cm^{-1} = 2924, 2855, 1902, 1728, 1628, 1510, 1463, 1371, 1316, 1256, 1214, 1142, 1018, 967, 834, 744, 672.

HRMS (APCI) for $C_{22}H_{32}BO_3^+$ [M+H]$^+$: calculated 355.2439, found 355.2440.

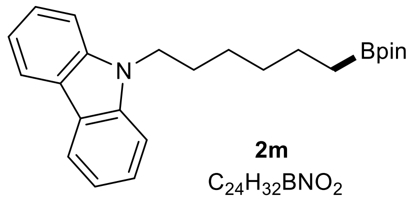

2m
$C_{24}H_{32}BNO_2$
M = 377.34 g/mol

9-(6-(4,4,5,5-Tetramethyl-1,3,2-dioxaborolan-2-yl) hexyl)-9H-carbazole (2m): Prepared from **1m** (53 mg, 0.20 mmol) and B_2cat_2 (95 mg, 0.40 mmol) according to **GP**. Purification by flash column chromatography on silica gel using *n*-pentane:ethyl acetate = 99:1 afforded **2m** as a colorless oil (67 mg, 89% yield).

R_f = 0.70 (*n*-pentane: ethyl acetate = 90:10).

^{1}H NMR (400 MHz, $CDCl_3$) δ 8.12 (d, *J* = 7.7 Hz, 2H), 7.50–7.46 (m, 2H), 7.42 (d, *J* = 8.1 Hz, 2H), 7.24 (t, *J* = 7.4 Hz, 2H), 4.30 (t, *J* = 7.3 Hz, 2H), 1.89–1.84 (m, 2H), 1.43–1.39 (m, 6H), 1.25 (s, 12H), 0.78 (t, *J* = 7.1 Hz, 2H) ppm.

^{13}C NMR (101 MHz, $CDCl_3$) δ 140.4, 125.5, 122.7, 120.3, 118.6, 108.6, 82.9, 43.0, 32.1, 28.8, 27.1, 24.8, 23.8 ppm. (The carbon atom attached to the boron atom was not observed due to quadrupolar relaxation.)

^{11}B NMR (128 MHz, $CDCl_3$) δ 34.2(s) ppm.

IR (ATR): ṽ/cm^{-1} = 3050, 2975, 2925, 2854, 2319, 2102, 1881, 1596, 1452, 1370, 1322, 1233, 1142, 966, 845, 747.

HRMS (APCI) for $C_{24}H_{33}BNO_2^+$ [M+H]$^+$: calculated 378.2599, found 378.2599.

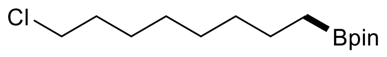

2n
$C_{14}H_{28}BClO_2$
M = 274.64 g/mol

2-(8-Chlorooctyl)-4,4,5,5-tetramethyl-1,3,2-dioxaborolane (2n): Prepared from 8-chlorooctan-1-ol (33 mg, 0.20 mmol) and B_2cat_2 (95 mg, 0.40 mmol) according to **GP**. Purification by flash column chromatography on silica gel using *n*-pentane:ethyl acetate = 99:1 afforded **2n** as a colorless oil (39 mg, 71% yield).

R_f = 0.75 (*n*-pentane: ethyl acetate = 90:10).

^{1}H NMR (400 MHz, $CDCl_3$) δ 3.51 (t, *J* = 6.8 Hz, 2H), 1.78–1.71 (m, 2H), 1.43–1.35 (m, 4H), 1.30–1.26 (m, 6H), 1.23 (s, 12H), 0.75 (t, *J* = 7.7 Hz, 2H) ppm.

^{13}C NMR (101 MHz, $CDCl_3$) δ 82.8, 45.1, 32.6, 32.2, 29.2, 28.7, 26.8, 24.8, 23.9 ppm. (The carbon atom attached to the boron atom was not observed due to quadrupolar relaxation.)

^{11}B NMR (128 MHz, $CDCl_3$) δ 34.2(s) ppm.

IR (ATR): ṽ/cm^{-1} = 2977, 2925, 2855, 2125, 1728, 1463, 1371, 1315, 1231, 1143, 967, 881, 846, 723.

HRMS (APCI) for $C_{14}H_{29}BClO_2^+$ [M+H]$^+$: calculated 275.1944, found 275.1943.

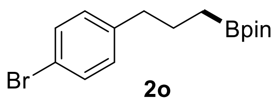

2o
$C_{15}H_{22}BBrO_2$
M = 324.09 g/mol

2-(3-(4-Bromophenyl) propyl)-4,4,5,5-tetramethyl-1,3,2-dioxaborolane (2o): Prepared from 3-(4-bromophenyl) propan-1-ol (43 mg, 0.20 mmol) and B_2cat_2 (95 mg, 0.40 mmol) according to **GP**. Purification by flash column chromatography on silica gel using *n*-pentane:ethyl acetate = 99:1 afforded **2o** as a colorless oil (46 mg, 71% yield).

R_f = 0.60 (*n*-pentane: ethyl acetate = 90:10).

^{1}H NMR (400 MHz, $CDCl_3$) δ 7.37 (d, *J* = 8.3 Hz, 2H), 7.04 (d, *J* = 8.3 Hz, 2H), 2.57–2.53 (m, 2H), 1.74–1.66 (m, 2H), 1.24 (s, 12H), 0.80 (t, *J* = 7.9 Hz, 2H) ppm.

^{13}C NMR (101 MHz, $CDCl_3$) δ 141.6, 131.2, 130.3, 119.2, 83.0, 37.8, 25.8, 24.8 ppm. (The carbon atom attached to the boron atom was not observed due to quadrupolar relaxation.)

^{11}B NMR (128 MHz, $CDCl_3$) δ 34.0(s) ppm.

IR (ATR): ṽ/cm^{-1} = 2976 ,2929, 2177, 2063, 1891, 1732, 1486, 1369, 1316, 1267, 1225, 1142, 1071, 1010, 967, 887, 845, 812, 672.

HRMS (APCI) for $C_{15}H_{23}BBrO_2^+$ [M+H]$^+$: calculated 325.0969, found 325.0971.

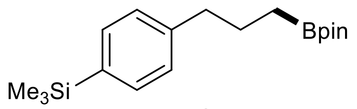

2p
$C_{18}H_{31}BO_2Si$
M = 318.34 g/mol

Trimethyl(4-(3-(4,4,5,5-tetramethyl-1,3,2-dioxaborolan-2-yl) propyl) phenyl) silane (2p): Prepared from **1p** (42 mg, 0.20 mmol) and B_2cat_2 (95 mg, 0.40 mmol) according to **GP**. Purification by flash column chromatography on silica gel using *n*-pentane:ethyl acetate = 99:1 afforded **2p** as a colorless oil (45 mg, 70% yield).

R_f = 0.75 (*n*-pentane: ethyl acetate = 90:10).

^{1}H NMR (400 MHz, CDCl3) δ 7.45 (d, *J* = 7.8 Hz, 2H), 7.20 (d, *J* = 7.8 Hz, 2H), 2.64– 2.60 (m, 2H), 1.79–1.72 (m, 2H), 1.25 (s, 12H), 0.85 (t, J = 7.9 Hz, 2H), 0.27 (s, 9H) ppm.

^{13}C NMR (101 MHz, $CDCl_3$) δ 143.3, 137.0, 133.2, 128.0, 82.9, 38.5, 26.0, 24.8, -1.1 ppm. (The carbon atom attached to the boron atom was not observed due to quadrupolar relaxation.)

^{11}B NMR (128 MHz, $CDCl_3$) δ 33.9(s) ppm.

^{29}Si NMR (79 MHz, $CDCl_3$) δ –4.5(s) ppm.

IR (ATR): ṽ/cm^{-1} = 2931, 2252, 1907, 1600, 1458, 1370, 1316, 1246, 1143, 1107, 968, 834, 753.

HRMS (APCI) for $C_{18}H_{32}BO_2Si^+$ [M+H]$^+$: calculated 319.2259, found 319.2264.

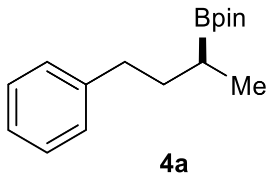

4a
$C_{16}H_{25}BO_2$
M = 260.18 g/mol

4,4,5,5-Tetramethyl-2-(4-phenylbutan-2-yl)-1,3,2-dioxaborolane (4a): Prepared from 4-phenylbutan-2-ol (30 mg, 0.20 mmol) and B_2cat_2 (95 mg, 0.40 mmol) according to **GP**. Purification by flash column chromatography on silica gel using *n*-pentane:ethyl acetate = 99:1 afforded **4a** as a colorless oil (37 mg, 71% yield).

R_f = 0.75 (*n*-pentane: ethyl acetate = 90:10).

^{1}H NMR (400 MHz, $CDCl_3$) δ 7.23 (d, *J* = 7.2 Hz, 2H), 7.17–7.12 (m, 3H), 2.60 (t, *J* = 7.6 Hz, 2H), 1.81–1.72 (m, 1H), 1.61–1.52 (m, 2H), 1.23 (s, 12H), 1.09–1.02 (m, 1H), 1.00 (d, *J* = 7.0 Hz, 3H) ppm.

^{13}C NMR (101 MHz, $CDCl_3$) δ 143.1, 128.4, 128.2, 125.5, 82.9, 35.3, 35.3, 24.8, 24.7, 15.4. ppm. (The carbon atom attached to the boron atom was not observed due to quadrupolar relaxation.)

^{11}B NMR (128 MHz, $CDCl_3$) δ 34.5(s) ppm.

IR (ATR): ṽ/cm^{-1} = 2926, 2119, 1874, 1603, 1458, 1368, 1313, 1213, 1141, 1008, 966, 847, 745, 697.

HRMS (APCI) for $C_{16}H_{26}BO_2^+$ $[M+H]^+$: calculated 261.2020, found 261.2022.

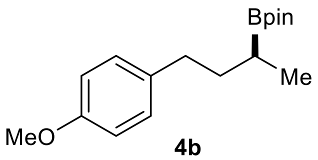

4b
$C_{17}H_{27}BO_3$
M = 290.21 g/mol

2-(4-(4-Methoxyphenyl) butan-2-yl)-4,4,5,5-tetramethyl-1,3,2-dioxaborolane (4b): Prepared from **3b** (36 mg, 0.20 mmol) and B_2cat_2 (95 mg, 0.40 mmol) according to **GP**. Purification by flash column chromatography on silica gel using *n*-pentane:ethyl acetate = 99:1 afforded **4b** as a colorless oil (47 mg, 81% yield).

R_f = 0.70 (*n*-pentane: ethyl acetate = 90:10).

^{1}H NMR (400 MHz, $CDCl_3$) δ 7.10 (d, *J* = 8.8 Hz, 2H), 6.81 (d, *J* = 8.6 Hz, 2H), 3.78 (s, 3H), 2.56 (t, *J* = 7.5 Hz, 2H), 1.79–1.70 (m, 1H), 1.57–1.50 (m, 1H), 1.25 (s, 12H), 1.09–1.04 (m, 1H), 1.01 (d, *J* = 5.8 Hz, 3H) ppm.

^{13}C NMR (101 MHz, $CDCl_3$) δ 157.5, 135.2, 129.3, 113.6, 82.8, 55.2, 35.5, 34.3, 24.8, 24.7, 15.4 ppm. (The carbon atom attached to the boron atom was not observed due to quadrupolar relaxation.)

^{11}B NMR (128 MHz, $CDCl_3$) δ 34.3(s) ppm.

IR (ATR): ṽ/cm^{-1} = 2927, 1510, 1461, 1368, 1313, 1241, 1141, 1036, 966, 821, 686.

HRMS (APCI) for $C_{17}H_{27}BO_3^+$ [M]$^+$: calculated 290.2048, found 290.2048.

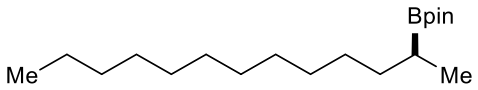

4c
$C_{19}H_{39}BO_2$
M = 310.33 g/mol

4,4,5,5-Tetramethyl-2-(tridecan-2-yl)-1,3,2-dioxaborolane (4c): Prepared from tridecan-2-ol (40 mg, 0.20 mmol) and B_2cat_2 (95 mg, 0.40 mmol) according to **GP**. Purification by flash column chromatography on silica gel using *n*-pentane:ethyl acetate = 99:1 afforded **4c** as a colorless oil (41 mg, 66% yield), and the spectroscopic data match the literature report [S2].

R_f = 0.75 (*n*-pentane: ethyl acetate = 90:10).

^{1}H NMR (400 MHz, $CDCl_3$) δ 1.46–1.39 (m, 1H), 1.31–1.23 (m, 32H), 0.95 (d, *J* = 5.1 Hz, 3H), 0.87 (t, *J* = 6.8 Hz, 3H) ppm.

^{13}C NMR (101 MHz, $CDCl_3$) δ 82.7, 33.2, 31.9, 29.8, 29.7, 29.6, 29.3, 29.0, 24.7, 24.7, 22.7, 15.5, 14.1 ppm. (The carbon atom attached to the boron atom was not observed due to quadrupolar relaxation.)

^{11}B NMR (128 MHz, $CDCl_3$) δ 34.4(s) ppm.

IR (ATR): ṽ/cm^{-1} = 2921, 2852, 2610, 2125, 2085, 1462, 1381, 1312, 1271, 1214, 1143, 1006, 967, 859, 720, 687.

GLC-MS (EI) for $C_{19}H_{39}BO_2$ [M$^+$]: calculated 310.33, found 310.3.

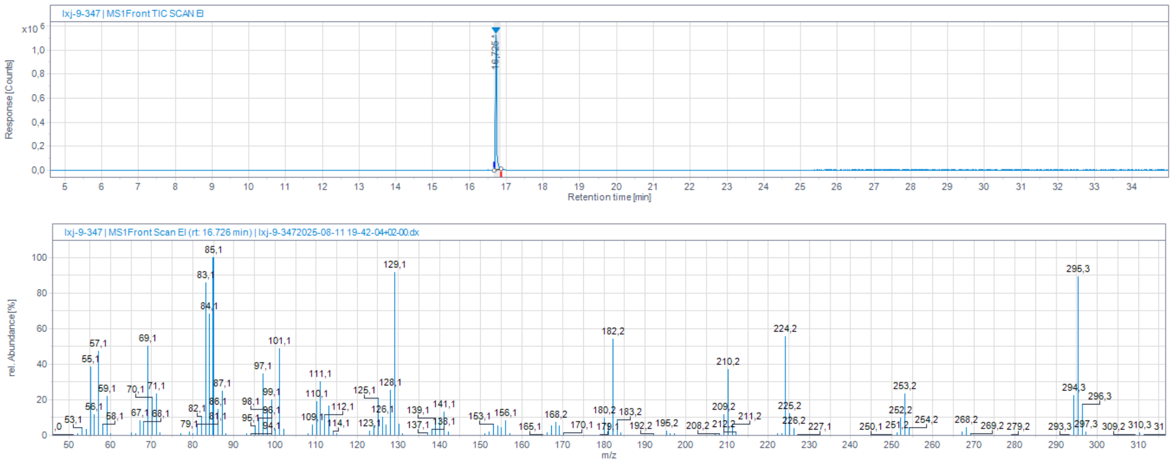

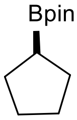

4d

$C_{11}H_{21}BO_2$

M = 196.10 g/mol

2-Cyclopentyl-4,4,5,5-tetramethyl-1,3,2-dioxaborolane (4d): Prepared from cyclopentanol (17 mg, 0.20 mmol) and B_2cat_2 (95 mg, 0.40 mmol) according to **GP**. Purification by flash column chromatography on silica gel using *n*-pentane:ethyl acetate = 99:1 afforded **4d** as a colorless oil (14 mg, 35% yield).

R$_f$ = 0.75 (*n*-pentane:ethyl acetate = 90:10).

^{1}H NMR (400 MHz, $CDCl_3$) δ 1.79–1.71 (m, 2H), 1.62–1.56 (m, 2H), 1.54–1.39 (m, 4H), 1.23 (s, 12H), 1.17 (t, *J* = 8.7 Hz, 1H) ppm.

^{13}C NMR (101 MHz, $CDCl_3$) δ 82.7, 28.5, 26.8, 24.7 ppm. (The carbon atom attached to the boron atom was not observed due to quadrupolar relaxation.)

^{11}B NMR (128 MHz, $CDCl_3$) δ 34.7(s) ppm.

IR (ATR): ṽ/cm^{-1} = 2947, 2865, 2608, 2325, 2130, 1996, 1921, 1737, 1452, 1415, 1378, 1308, 1272, 1214, 1142, 1030, 1004, 971, 927, 889, 857, 670.

HRMS (APCI) for $C_{11}H_{22}BO_2^+$ [M+H]$^+$: calculated 197.1707, found 197.1707.

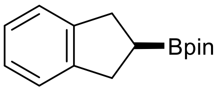

4e
$C_{15}H_{21}BO_2$
M = 244.14 g/mol

2-(2,3-Dihydro-1*H*-inden-2-yl)-4,4,5,5-tetramethyl-1,3,2-dioxaborolane (4e): Prepared from 2,3-dihydro-1H-inden-2-ol (27 mg, 0.20 mmol) and B_2cat_2 (95 mg, 0.40 mmol) according to **GP**. Purification by flash column chromatography on silica gel using *n*-pentane:ethyl acetate = 99:1 afforded **4e** as a colorless oil (20 mg, 40% yield).

R_f = 0.75 (*n*-pentane: ethyl acetate = 90:10).

^{1}H NMR (500 MHz, $CDCl_3$) δ 7.20 (d, *J* = 8.6 Hz, 2H), 7.14–7.10 (m, 2H), 3.06 (dd, *J* = 15.6, 9.4 Hz, 2H), 2.97 (dd, *J* = 15.6, 10.3 Hz, 2H), 1.94–1.84 (m, 1H), 1.27 (s, 12H) ppm.

^{13}C NMR (101 MHz, $CDCl_3$) δ 144.4, 125.9, 124.2, 83.2, 35.1, 24.7 ppm. (The carbon atom attached to the boron atom was not observed due to quadrupolar relaxation.)

^{11}B NMR (128 MHz, $CDCl_3$) δ 35.0 ppm.

IR (ATR): ṽ/cm^{-1} = 3067, 2976, 2930, 2845, 2325, 2113, 2068, 1995, 1894, 1827, 1723, 1601, 1459, 1414, 1373, 1313, 1260, 1214, 1140, 1006, 970, 856, 783, 740, 669.

HRMS (APCI) for $C_{15}H_{22}BO_2^+$ [M+H]$^+$: calculated 245.1707, found 245.1703.

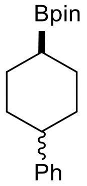

4f
$C_{18}H_{27}BO_2$
M = 286.22 g/mol

4,4,5,5-Tetramethyl-2-(4-phenylcyclohexyl)-1,3,2-dioxaborolane (4f): Prepared from **3f** (35 mg, 0.20 mmol) and B_2cat_2 (95 mg, 0.40 mmol) according to **GP**. Purification by flash column chromatography on silica gel using *n*-pentane:ethyl acetate = 99:1 afforded **4f** as a white solid (mixture of *trans*:*cis*= 88:12, 17 mg in total, 30% yield).

R_f = 0.75 (*n*-pentane: ethyl acetate = 90:10)

M.p.: 67–69 °C.

^{1}H NMR (400 MHz, $CDCl_3$) δ 7.21 (d, *J* = 8.3 Hz, 2H), 7.14–7.07 (m, 3H), 2.44–2.40 (*trans*, m, 1H), 2.10–2.06 (*cis*, m, 0.16H), 1.96–1.94 (*cis*, m, 0.28H), 1.86–1.76 (*trans*, m, 4H), 1.49–1.44 (*cis*, m, 0.53H), 1.41–1.40 (*cis*, m, 0.22H),1.38–1.30 (*trans*, m, 4H), 1.22 (*cis*, s, 1.69H) 1.18 (*trans*, s, 12H), 0.90–0.86(*trans*, m, 1H) ppm.

^{13}C NMR (101 MHz, $CDCl_3$) δ 148.2(*cis*), 148.1(*trans*), 128.4(*cis*), 128.2(*trans*), 126.8(*trans*), 126.7(*cis*), 125.7(*trans*), 125.7(*cis*), 83.0(*cis*), 82.8(*trans*), 44.5(*cis*), 44.4(*trans*), 35.0(*trans*), 33.3(*cis*), 28.2(*trans*), 27.9(*cis*), 24.9(*cis*), 24.7(*trans*) ppm. (The carbon atom attached to the boron atom was not observed due to quadrupolar relaxation.)

^{11}B NMR (128 MHz, $CDCl_3$) δ 33.9(s) ppm.

IR (ATR): ṽ/cm^{-1} = 3059, 3025, 2975, 2919, 2849, 2340, 2204, 2211, 2103, 2002, 1868, 1723, 1600, 1491, 1447, 1411, 1381, 1311, 1261, 1217, 1140, 1082, 992, 966, 849, 754, 697, 671.

HRMS (APCI) for $C_{18}H_{28}BO_2^+$ [M+H]$^+$: calculated 287.2177, found 287.2170.

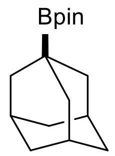

6
$C_{16}H_{27}BO_2$
M = 262.20 g/mol

2-((3*r*,5*r*,7*r*)-Adamantan-1-yl)-4,4,5,5-tetramethyl-1,3,2-dioxaborolane (6): Prepared from adamantan-1-ol (30 mg, 0.20 mmol) and B_2cat_2 (95 mg, 0.40 mmol) according to **GP**. Purification by flash column chromatography on silica gel using *n*-pentane:ethyl acetate = 99:1 afforded **6** as a colorless oil (43 mg, 82% yield).

R_f = 0.80 (*n*-pentane: ethyl acetate = 90:10).

^{1}H NMR (400 MHz, $CDCl_3$) δ 1.86–1.81 (m, 3H), 1.76–1.74 (m, 12H), 1.20 (s, 12H) ppm.

^{13}C NMR (101 MHz, $CDCl_3$) δ 82.6, 38.0, 37.5, 27.6, 24.6 ppm. (The carbon atom attached to the boron atom was not observed due to quadrupolar relaxation.)

^{11}B NMR (128 MHz, $CDCl_3$) δ 33.5(s) ppm.

IR (ATR): ṽ/cm^{-1} = 2977, 2898, 2844, 2676, 2243, 2185, 1738, 1449, 1379, 1346, 1293, 1213, 1144, 1101, 1066, 961, 908, 851, 730, 676.

HRMS (APCI) for $C_{16}H_{26}BO_2^+$ [M–H]$^+$: calculated 261.2020, found 261.2018.

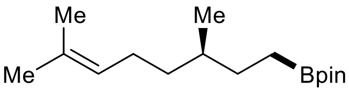

8a
$C_{16}H_{31}BO_2$
M = 266.23 g/mol

(*S*)-2-(3,7-Dimethyloct-6-en-1-yl)-4,4,5,5-tetramethyl-1,3,2-dioxaborolane (8a): Prepared from (*R*)-3,7-dimethyloct-6-en-1-ol (31 mg, 0.20 mmol) and B_2cat_2 (95 mg, 0.40 mmol) according to **GP**. Purification by flash column chromatography on silica gel using *n*-pentane:ethyl acetate = 99:1 afforded **8a** as a colorless oil (34 mg, 63% yield).

R_f = 0.75 (*n*-pentane: ethyl acetate = 90:10).

^{1}H NMR (400 MHz, $CDCl_3$) δ 5.10–5.06 (m, 1H), 2.02–1.87 (m, 2H), 1.66 (s, 3H), 1.59 (s, 3H), 1.47–1.26 (m, 4H), 1.23 (s, 12H), 1.14–1.06 (m, 1H), 0.84 (d, *J* = 6.3 Hz, 3H), 0.80–0.67 (m, 2H) ppm.

^{13}C NMR (101 MHz, $CDCl_3$) δ 130.8, 125.1, 82.8, 36.7, 34.5, 30.9, 25.7, 25.6, 24.8, 24.8, 19.1, 17.6 ppm. (The carbon atom attached to the boron atom was not observed due to quadrupolar relaxation.)

^{11}B NMR (128 MHz, $CDCl_3$) δ 34.2(s) ppm.

IR (ATR): $\tilde{v}$/cm^{-1} = 2921, 2092, 1732, 1451, 1370, 1315, 1144, 967, 846, 741, 673.

HRMS (APCI) for $C_{16}H_{32}BO_2^+$ [M+H]$^+$: calculated 267.2490, found 267.2491.

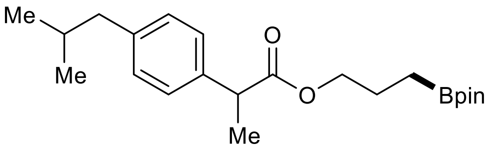

8b
$C_{22}H_{35}BO_4$
M = 374.33 g/mol

3-(4,4,5,5-Tetramethyl-1,3,2-dioxaborolan-2-yl) propyl 2-(4-isobutylphenyl) propanoate (8b): Prepared from **7b** (53 mg, 0.20 mmol) and B_2cat_2 (95 mg, 0.40 mmol) according to **GP**. Purification by flash column chromatography on silica gel using *n*-pentane:ethyl acetate = 98:2 afforded **8b** as a colorless oil (38 mg, 51% yield).

R_f = 0.50 (*n*-pentane: ethyl acetate = 90:10).

^{1}H NMR (400 MHz, $CDCl_3$) δ 7.19 (d, *J* = 8.1 Hz, 2H), 7.08 (d, *J* = 8.1 Hz, 2H), 4.08–3.96 (m, 2H), 3,70–3.65 (m, 1H), 2.43 (d, *J* = 7.2 Hz, 2H), 1.89–1.79 (m, 1H), 1.73–1.65 (m, 2H), 1.47 (d, *J* = 7.2 Hz, 3H), 1.23 (s, 12H), 0.89 (d, *J* = 6.6 Hz, 6H), 0.77–0.73 (m, 2H) ppm.

^{13}C NMR (101 MHz, $CDCl_3$) δ 174.8, 140.3, 137.9, 129.2, 127.1, 83.1, 66.4, 45.1, 45.0, 30.1, 24.8, 23.0, 22.4, 18.5 ppm. (The carbon atom attached to the boron atom was not observed due to quadrupolar relaxation.)

^{11}B NMR (128 MHz, $CDCl_3$) δ 33.8(s) ppm.

IR (ATR): ṽ/cm^{-1} = 2953, 1731, 1463, 1370, 1318, 1142, 1092, 966, 845, 798, 728, 672.

HRMS (APCI) for $C_{22}H_{36}BO_4^+$ [M+H]$^+$: calculated 375.2701, found 375.2704.

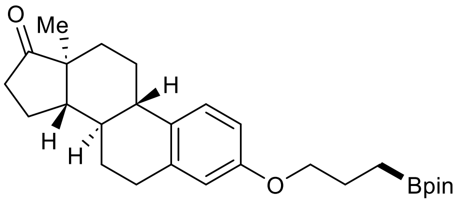

8c
$C_{27}H_{39}BO_4$
M = 438.42 g/mol

(8*R*,9*S*,13*S*,14*S*)-13-Methyl-3-(3-(4,4,5,5-tetramethyl-1,3,2-dioxaborolan-2-yl)propoxy)-6,7,8,9,11,12,13,14,15,16-decahydro-17H-cyclopenta[a]phenanthren-17-one (8c): Prepared from **7c** (66 mg, 0.20 mmol) and B_2cat_2 (95 mg, 0.40 mmol) according to **GP**. Purification by flash column chromatography on silica gel using *n*-pentane:ethyl acetate = 98:2 afforded **8c** as a white solid (55 mg, 63% yield).

R_f = 0.50 (*n*-pentane: ethyl acetate = 80:20).

M.p.: 147–149 °C.

^{1}H NMR (400 MHz, $CDCl_3$) δ 7.18 (d, *J* = 8.7 Hz, 1H), 6.71 (d, *J* = 8.6 Hz, 1H), 6.65 (s, 1H), 3.91 (t, *J* = 6.7 Hz, 2H), 2.91–2.85 (m, 2H), 2.50 (dd, *J* = 18.8, 8.5 Hz, 1H), 2.41–2.38 (m, 1H), 2.27–2.23 (m, 1H), 2.19–1.93 (m, 4H), 1.91–1.84 (m, 2H), 1.67–1.59 (m, 2H), 1.57–1.39 (m, 5H), 1.25 (s, 13H), 0.93–0.89 (m, 5H) ppm.

^{13}C NMR (101 MHz, $CDCl_3$) δ 221.1, 157.1, 137.6, 131.7, 126.2, 114.6, 112.2, 83.1, 69.5, 50.4, 48.0, 44.0, 38.4, 35.9, 31.6, 29.6, 26.6, 25.9, 24.8, 23.8, 21.6, 13.8 ppm. (The carbon atom attached to the boron atom was not observed due to quadrupolar relaxation.)

^{11}B NMR (128 MHz, $CDCl_3$) δ 33.7(s) ppm.

IR (ATR): ṽ/cm^{-1} = 3447, 2931, 2628, 2321, 2083, 1847, 1732, 1609, 1571, 1495, 1473, 1369, 1328, 1253, 1140, 1058, 1004, 965, 946, 868, 845, 807, 741, 671.

HRMS (APCI) for $C_{27}H_{40}BO_4^+$ [M+H]$^+$: calculated 439.3014, found 439.3016.

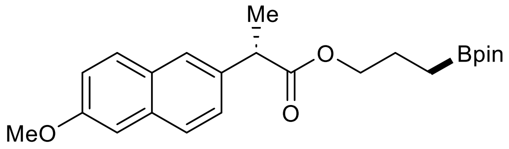

8d

$C_{23}H_{31}BO_5$

M = 398.31 g/mol

3-(4,4,5,5-Tetramethyl-1,3,2-dioxaborolan-2-yl) propyl (*S*)-2-(6-methoxynaphthalen-2-yl) propanoate (8d): Prepared from **7d** (58 mg, 0.20 mmol) and B_2cat_2 (95 mg, 0.40 mmol) according to **GP**. Purification by flash column chromatography on silica gel using *n*-pentane:ethyl acetate = 98:2 afforded **8d** as a colorless oil (54 mg, 68% yield).

R$_f$ = 0.45 (*n*-pentane: ethyl acetate = 90:10).

^{1}H NMR (400 MHz, $CDCl_3$) δ 7.71–7.66 (m, 3H), 7.41 (dd, *J* = 8.5, 1.9 Hz, 1H), 7.15–7.10 (m, 2H), 4.11–3.99 (m, 2H), 3.90 (s, 3H), 3.87–3.82 (m, 1H), 1.74–1.66 (m, 2H), 1.57 (d, *J* = 7.1 Hz, 3H), 1.22 (s, 12H), 0.76 (t, *J* = 8.0 Hz, 2H) ppm.

^{13}C NMR (101 MHz, $CDCl_3$) δ 174.7, 157.5, 135.8, 133.6, 129.2, 128.9, 127.0, 126.3, 125.8, 118.8, 105.5, 83.0, 66.5, 55.2, 45.5, 24.7, 23.0, 18.5 ppm. (The carbon atom attached to the boron atom was not observed due to quadrupolar relaxation.)

^{11}B NMR (128 MHz, $CDCl_3$) δ 33.6(s) ppm.

IR (ATR): ṽ/cm^{-1} = 2928, 2317, 2127, 1901, 1730, 1606, 1371, 1321, 1264, 1143, 1032, 967, 849, 810, 745, 671.

HRMS (APCI) for $C_{23}H_{32}BO_5^+$ [M+H]$^+$: calculated 399.2337, found 399.2338.

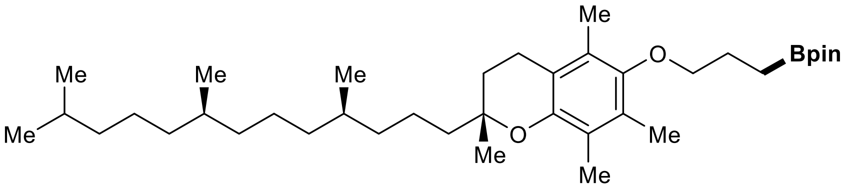

8e
$C_{38}H_{67}BO_4$
M = 598.76 g/mol

4,4,5,5-Tetramethyl-2-(3-(((*S*)-2,5,7,8-tetramethyl-2-((4*S*,8*S*)-4,8,12-trimethyltridecyl) chroman-6-yl) oxy) propyl)-1,3,2-dioxaborolane (8e): Prepared from **7e** (98 mg, 0.20 mmol) and B_2cat_2 (95 mg, 0.40 mmol) according to **GP**. Purification by flash column chromatography on silica gel using *n*-pentane:ethyl acetate = 98:2 afforded **8e** as a colorless oil (91 mg, 76% yield).

$\mathbf{R_f}$ = 0.60 (*n*-pentane: ethyl acetate = 90:10).

^{1}H NMR (400 MHz, $CDCl_3$) δ 3.60 (t, *J* = 6.6 Hz, 2H), 2.56 (t, *J* = 6.8 Hz, 2H), 2.16 (s, 3H), 2.12 (s, 3H), 2.07 (s, 3H), 1.95–1.88 (m, 2H), 1.84–1.71 (m, 2H), 1.56–1.31(m, 8H), 1.28–1.23 (m, 21H), 1.17–1.03 (m, 7H), 0.96 (t, *J* = 7.8 Hz, 2H), 0.88–0.84 (m, 12H) ppm.

^{13}C NMR (101 MHz, $CDCl_3$) δ 148.3, 147.5, 127.9, 125.9, 122.6, 117.4, 83.0, 74.7, 74.6, 40.1, 39.4, 37.5, 37.4, 37.4, 37.3, 32.8, 32.7, 31.3, 28.0, 24.9, 24.8, 24.8, 24.8, 24.4, 23.9, 22.7, 22.6, 21.0, 20.6, 19.7, 19.6, 12.7, 11.8, 11.8 ppm. (The carbon atom attached to the boron atom was not observed due to quadrupolar relaxation.)

^{11}B NMR (128 MHz, $CDCl_3$) δ 33.5(s) ppm.

IR (ATR): $\tilde{\nu}$/cm^{-1} = 2923, 2331, 2080, 1730, 1458, 1368, 1316, 1254, 1144, 1087, 1008, 921, 845, 737, 671.

HRMS (APCI) for $C_{38}H_{68}BO_4^+$ $[M+H]^+$: calculated 599.5205, found 599.5214.

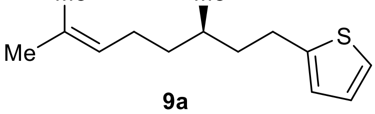

9a
$C_{14}H_{22}S$
M = 222.39 g/mol

(*R*)-2-(3,7-Dimethyloct-6-en-1-yl) thiophene (9a): According to the literature[S3], a solution of thiophene (71 mg, 0.85 mmol, 4.3 equiv) in THF (3.0 mL) was cooled to –78 °C and treated with *n*-BuLi (0.30 mL, 0.75 mmol, 2.5 M in hexanes). The cooling bath was removed and the

mixture was stirred at room temperature for 1 hour. The mixture was cooled to –78 °C and **8a** (53 mg, 0.20 mmol) was added dropwise as a solution in THF (3.5 mL). The mixture was stirred at –78 °C for 1 hour. A solution of the *N*-bromosuccinimide (NBS) (160 mg, 0.90 mmol, 4.5 equiv) in THF (3.5 mL) was added dropwise. After 1 hour at –78 °C, $Na_2S_2O_3$ sat. (10 mL) was added and the reaction mixture was allowed to warm to room temperature. The reaction mixture was diluted with Et_2O and water. The layers were separated and the aqueous layer was extracted with Et_2O. The combined organic layers were dried ($MgSO_4$), filtered and concentrated under vacuum. The crude material was adsorbed on silica and purified by flash column chromatography on silica gel using *n*-pentane:ethyl acetate = 99:1 afforded **9a** as a colorless oil (30 mg, 68% yield).
R_f = 0.85 (*n*-pentane: ethyl acetate = 90:10).

^{1}H NMR (500 MHz, $CDCl_3$) δ 7.10 (d, *J* = 5.1 Hz, 1H), 6.92 (t, *J* = 4.3 Hz, 1H), 6.78 (d, *J* = 4.0 Hz, 1H), 5.12 (t, *J* = 7.3 Hz, 1H), 2.91–2.78(m, 2H), 2.07–1.93 (m, 2H), 1.75–1.70 (m, 4H), 1.62 (s, 3H), 1.57–1.49 (m, 3H), 1.43–1.38 (m, 1H), 1.25–1.19 (m, 1H), 0.95 (d, *J* = 5.9 Hz, 3H) ppm.

^{13}C NMR (126 MHz, $CDCl_3$) δ 146.0, 131.2, 126.6, 124.8, 123.8, 122.6, 39.0, 36.9, 32.0, 27.5, 25.7, 25.5, 19.4, 17.6 ppm.

IR (ATR): $\tilde{v}$/cm^{-1} = 2957, 2912, 2851, 2726, 2661, 2122, 2002, 1897, 1775, 1578, 1534, 1440, 1376, 1240, 1108, 1081, 1035, 983, 932, 848, 818, 740, 687.

HRMS (APCI) for $C_{14}H_{23}S^+$ $[M+H]^+$: calculated 223.1515, found 223.1515.

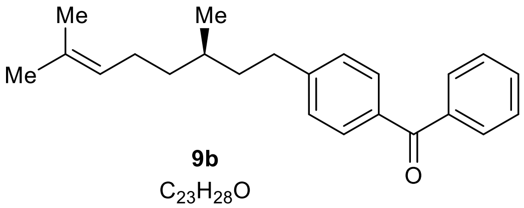

9b
$C_{23}H_{28}O$
M = 320.48 g/mol

(*R*)-(4-(3,7-Dimethyloct-6-en-1-yl) phenyl) (phenyl) methanone (9b): According to the literature[S4], to a Schlenck tube, $Pd_2(dba)_3$ (3.7 mg, 4.0 *µ*mol, 2 mol %), Ruphos (3.8 mg, 8.0 *µ*mol, 4 mol %), **8a** (53 mg, 0.20 mmol), 4-bromobenzophenone (62 mg, 0.24 mmol, 1.2 equiv) and NaO*t*Bu (58 mg, 0.60 mmol, 3.0 equiv) were added. The mixture was diluted with THF (0.5 mL) and H_2O (50 *µ*L) under an atmosphere of nitrogen. The mixture was stirred for 24 hours at 80 °C, and monitored by TLC. Upon completion of the reaction, the reaction mixture was diluted with diethyl ether (10 mL). After the aqueous layer was extracted with diethyl ether

(10 mL) twice and the combined organic layers were dried over Na_2SO_4, and concentrated in vacuo. The product **9b** was purified by flash column chromatography on silica gel using *n*-pentane:ethyl acetate = 99:1 afforded **9b** as a colorless oil (56 mg, 87% yield).

R_f = 0.85 (*n*-pentane: ethyl acetate = 90:10).

^{1}H NMR (400 MHz, $CDCl_3$) δ 7.79 (d, *J* = 7.0 Hz, 2H), 7.74 (d, *J* = 7.9 Hz, 2H), 7.58 (t, *J* = 7.4 Hz, 1H), 7.47 (t, *J* = 7.5 Hz, 2H), 7.29 (d, *J* = 7.9 Hz, 2H), 5.11 (t, *J* = 7.3 Hz, 1H), 2.77–2.62 (m, 2H), 2.06–1.93 (m, 2H), 1.69–1.64 (m, 4H), 1.61 (s, 3H), 1.54–1.45 (m, 2H), 1.43–1.36 (m, 1H), 1.26–1.19 (m, 1H), 0.96 (d, *J* = 6.0 Hz, 3H) ppm.

^{13}C NMR (101 MHz, $CDCl_3$) δ 196.4, 148.4, 137.9, 135.0, 132.1, 131.2, 130.3, 129.9, 128.3, 128.2, 124.7, 38.5, 36.9, 33.5, 32.1, 25.7, 25.4, 19.5, 17.6 ppm.

IR (ATR): ṽ/cm^{-1} = 3315, 3208, 3056, 3027, 2958, 2912, 2853, 2093, 1996, 1922, 1809, 1656, 1604, 1446, 1412, 1377, 1311, 1276, 1177, 1148, 1116, 1074, 1026, 956, 922, 843, 789, 740, 700.

HRMS (APCI) for $C_{23}H_{29}O^+$ [M+H]$^+$: calculated 321.2213, found 321.2209.

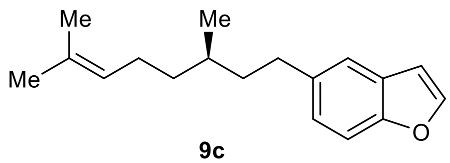

9c
$C_{18}H_{24}O$
M = 256.39 g/mol

(*R*)-5-(3,7-Dimethyloct-6-en-1-yl) benzofuran (9c): According to the literature[S5], inside a nitrogen-filled glove box, a 10 mL Schlenck tube equipped with a stir bar was charged with $Pd_2(dba)_3$ (1.8 mg, 2.0 *μ*mol, 1 mol %), *p*-$MeOC_6H_4PPh_2$ (1.7 mg, 6.0 *μ*mol, 3 mol %), 5-bromobenzofuran (39 mg, 0.20 mmol), and K_3PO_4 (256 mg, 1.2 mmol, 6.0 equiv). The Schlenck tube was sealed with a rubber septum and then removed from the glove box and placed in a heating block. 1,4-dioxane (1.0 mL) was added to the vial *via* syringe and the resulting mixture was stirred at room temperature for 5 minutes. Then **8a** (122 mg, 0.46 mmol, 2.3 equiv) and degassed DI water (0.50 mL) were added sequentially to the reaction *via* syringe. The heating block was heated to 100 °C and held at that temperature for 18 hours. Upon completion of the reaction, the reaction mixture was diluted with diethyl ether (10 mL). After the aqueous layer was extracted with diethyl ether and the combined organic layers were dried over Na_2SO_4, and concentrated in vacuo. The product **9c** was purified by flash column chromatography on silica gel using *n*-pentane:ethyl acetate = 99:1 afforded **9c** as a colorless

oil (33 mg, 65% yield).

R_f = 0.85 (*n*-pentane: ethyl acetate = 90:10).

^{1}H NMR (500 MHz, $CDCl_3$) δ 7.59 (s, 1H), 7.41 (d, *J* = 9.1 Hz, 2H), 7.12 (d, *J* = 8.4 Hz, 1H), 6.71 (d, *J* = 2.2 Hz, 1H), 5.12 (t, *J* = 7.9 Hz, 1H), 2.78–2.73(m, 1H), 2.70–2.64 (m, 1H), 2.07–1.93 (m, 2H), 1.70–1.66(m, 4H), 1.62 (s, 3H), 1.53–1.47 (m, 2H), 1.45–1.38 (m, 1H), 1.25–1.20 (m, 1H), 0.97 (d, *J* = 6.0 Hz, 3H) ppm.

^{13}C NMR (126 MHz, $CDCl_3$) δ 153.5, 145.0, 137.6, 131.1, 127.4, 124.9, 124.9, 120.3, 110.9, 106.3, 39.5, 37.0, 33.3, 32.1, 25.7, 25.5, 19.6, 17.6 ppm.

IR (ATR): ṽ/cm^{-1} = 3022, 2958, 2912, 2853, 2727, 2465, 2374, 2339, 2244, 2195, 2056, 2019, 1920, 1867, 1700, 1585, 1537, 1465, 1376, 1329, 1260, 1195, 1126, 1031, 983, 933, 878, 805, 762, 732.

HRMS (APCI) for $C_{18}H_{25}O^+$ [M+H]$^+$: calculated 257.1900, found 257.1900.

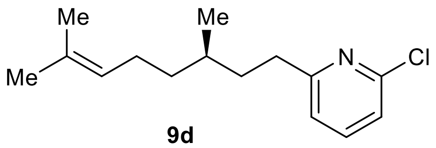

9d

$C_{15}H_{22}ClN$

M = 251.80 g/mol

(*R*)-2-Chloro-6-(3,7-dimethyloct-6-en-1-yl) pyridine (9d): According to the literature[S4], inside a nitrogen-filled glove box, a 10 mL Schlenck tube equipped with a stir bar was charged with $Pd_2(dba)_3$ (1.8 mg, 2.0 *µ*mol, 1 mol %), *p*$MeOC_6H_4PPh_2$ (1.7 mg, 6.0 *µ*mol, 3 mol %), 2,6-dichloropyridine (29 mg, 0.20 mmol), and K_3PO_4 (256 mg, 1.2 mmol, 6.0 equiv). The Schlenck tube was sealed with a rubber septum and then removed from the glove box and placed in a heating block. 1,4-dioxane (1.0 mL) was added to the vial *via* syringe and the resulting mixture was stirred at room temperature for 5 minutes. Then **8a** (122 mg, 0.46 mmol, 2.3 equiv) and degassed DI water (0.50 mL) were added sequentially to the reaction *via* syringe. The heating block was heated to 100 °C and held at that temperature for 18 hours. Upon completion of the reaction, the reaction mixture was diluted with diethyl ether (10 mL). After the aqueous layer was extracted with diethyl ether and the combined organic layers were dried over Na_2SO_4, and concentrated in vacuo. The product **9d** was purified by flash column chromatography on silica gel using *n*-pentane:ethyl acetate = 99:1 afforded **9d** as a colorless oil (42 mg, 84% yield).

R_f = 0.85 (*n*-pentane: ethyl acetate = 90:10).

^{1}H NMR (400 MHz, $CDCl_3$) δ 7.53 (t, *J* = 7.7 Hz, 1H), 7.12 (d, *J* = 7.9 Hz, 1H), 7.05 (d, *J* = 7.5 Hz, 1H), 5.08 (t, *J* = 6.9 Hz, 1H), 2.83–2.67 (m, 2H), 2.05–1.89 (m, 2H), 1.76–1.67 (m, 4H), 1.59 (s, 3H), 1.54–1.45 (m, 2H), 1.43–1.34(m, 1H), 1.21–1.16 (m, 1H), 0.93 (d, *J* = 6.2 Hz, 3H) ppm.

^{13}C NMR (101 MHz, $CDCl_3$) δ 164.0, 150.7, 138.8, 131.2, 124.7, 121.3, 121.0, 36.9, 35.7, 32.3, 25.7, 25.4, 19.4, 17.6 ppm.

IR (ATR): $\tilde{\nu}$/cm^{-1} = 3167, 3055,2958, 2913, 2854, 2727, 2306, 2223, 2159, 2021, 1969,1730, 1670,1584,1559, 1437, 1408, 1376, 1320, 1230, 1161,1138,987, 881, 827, 789, 736, 689.

HRMS (APCI) for $C_{15}H_{23}ClN^+$ $[M+H]^+$: calculated 252.1514, found 252.1511.

5 NMR Spectra of Products

Figure S2. ^{1}H NMR (400 MHz, $CDCl_3$, 298K) of **1j**.

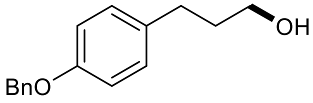

1j

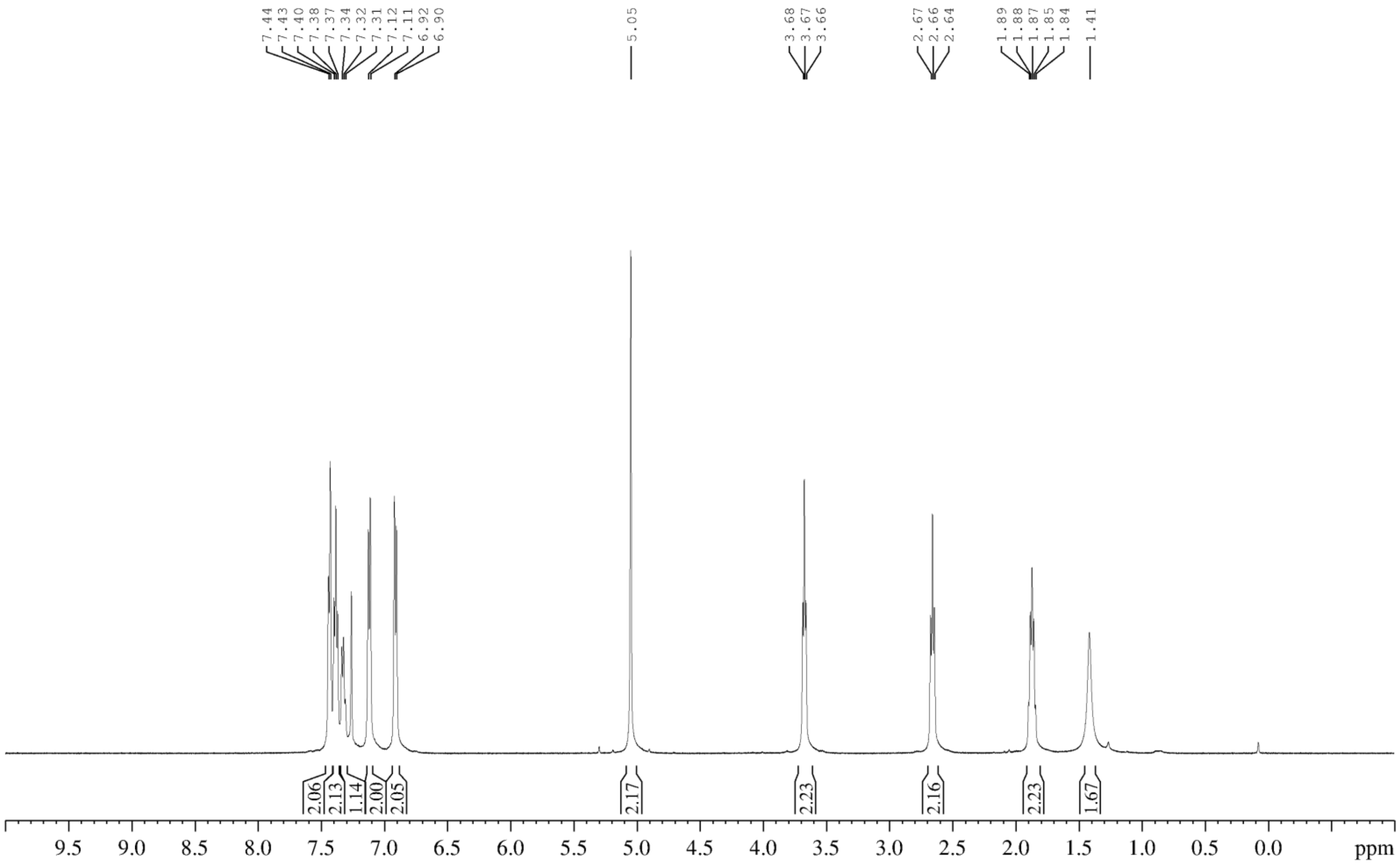

Figure S3. ^{13}C NMR (101 MHz, $CDCl_3$, 298K) of **1j**.

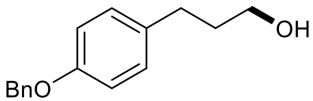

1j

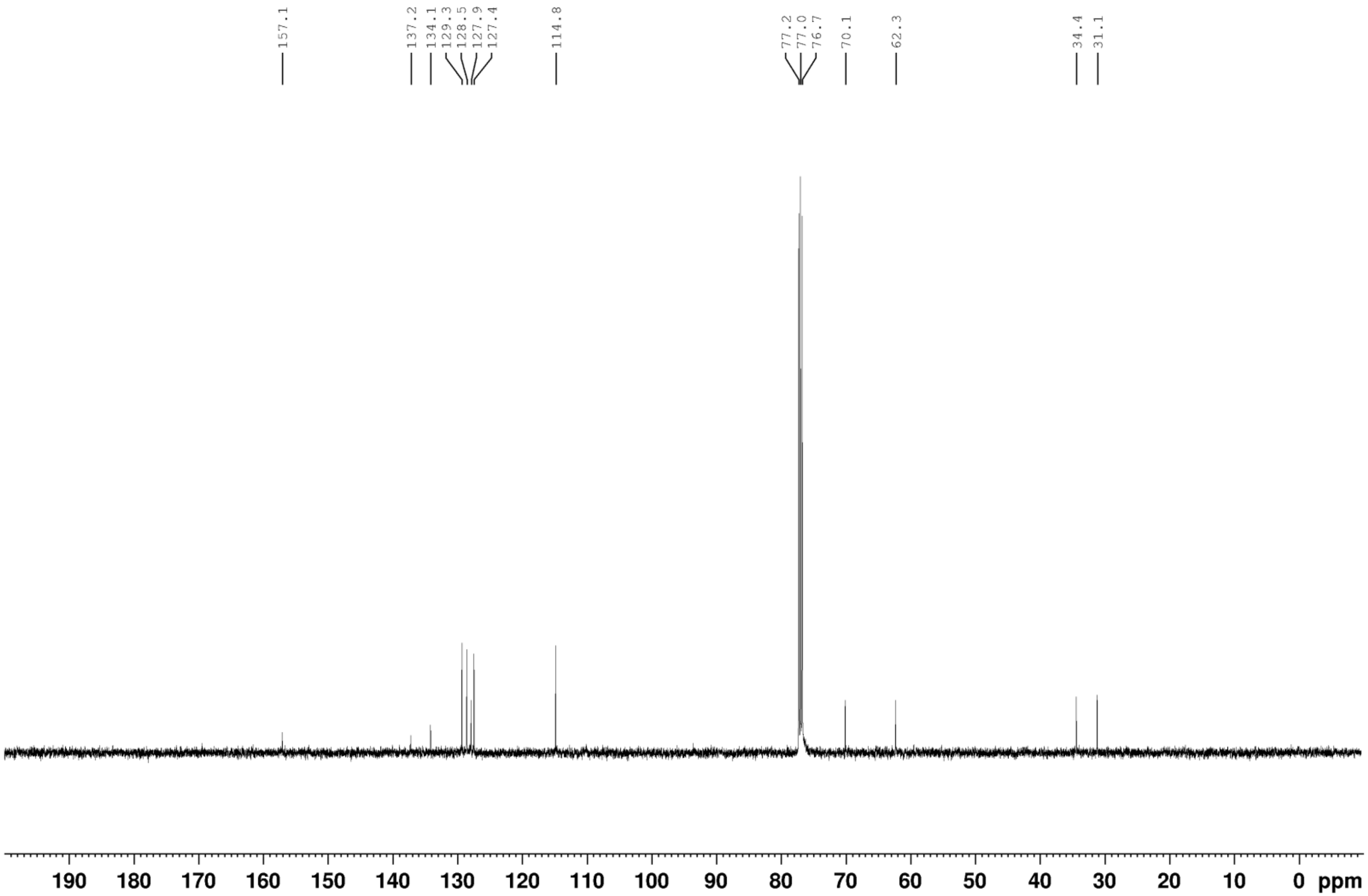

Figure S4. ^{1}H NMR (400 MHz, $CDCl_3$, 298K) of **3b**.

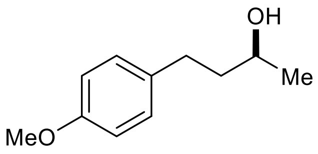

3b

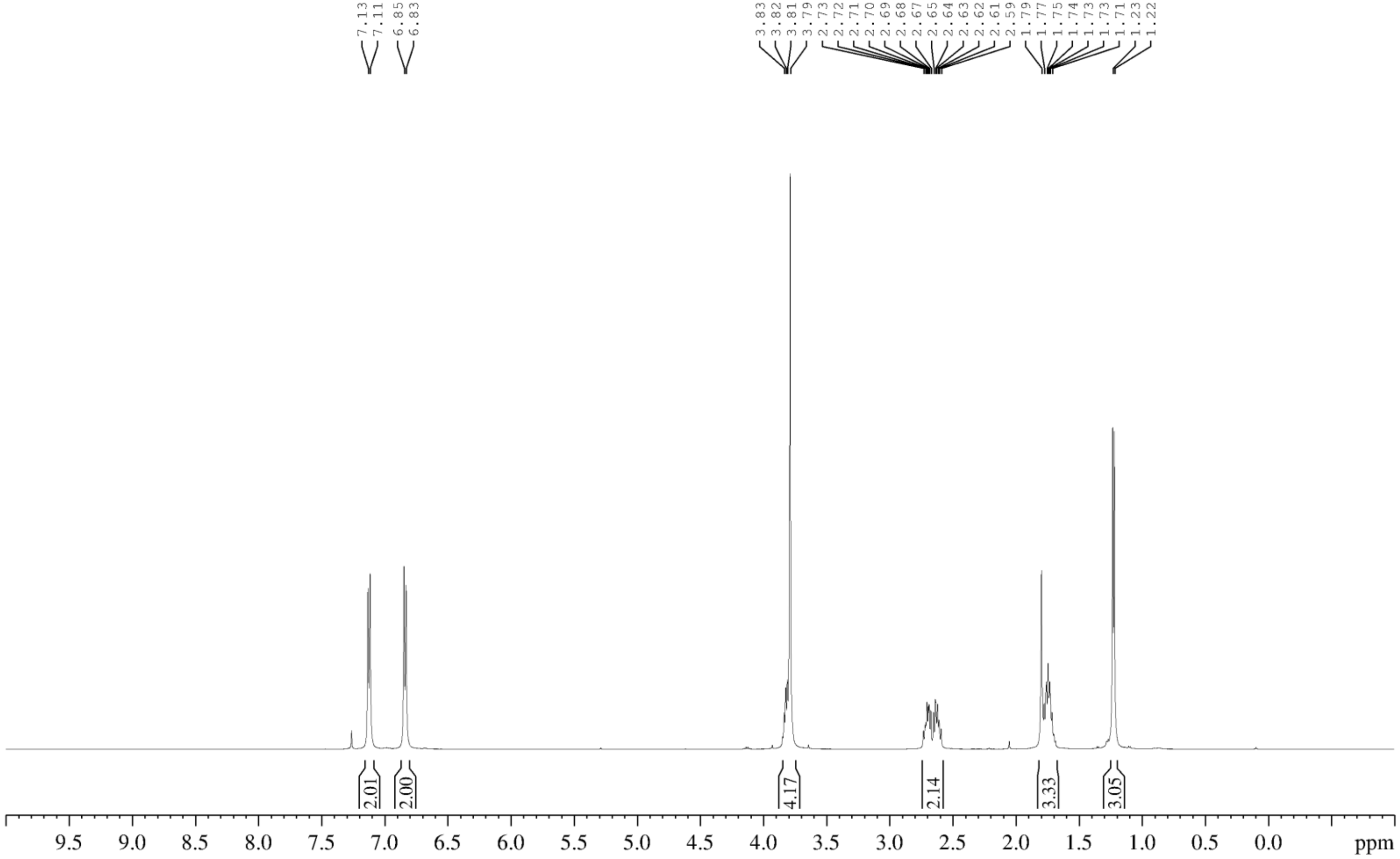

Figure S5. ^{13}C NMR (101 MHz, $CDCl_3$, 298K) of **3b**.

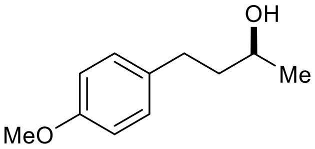

3b

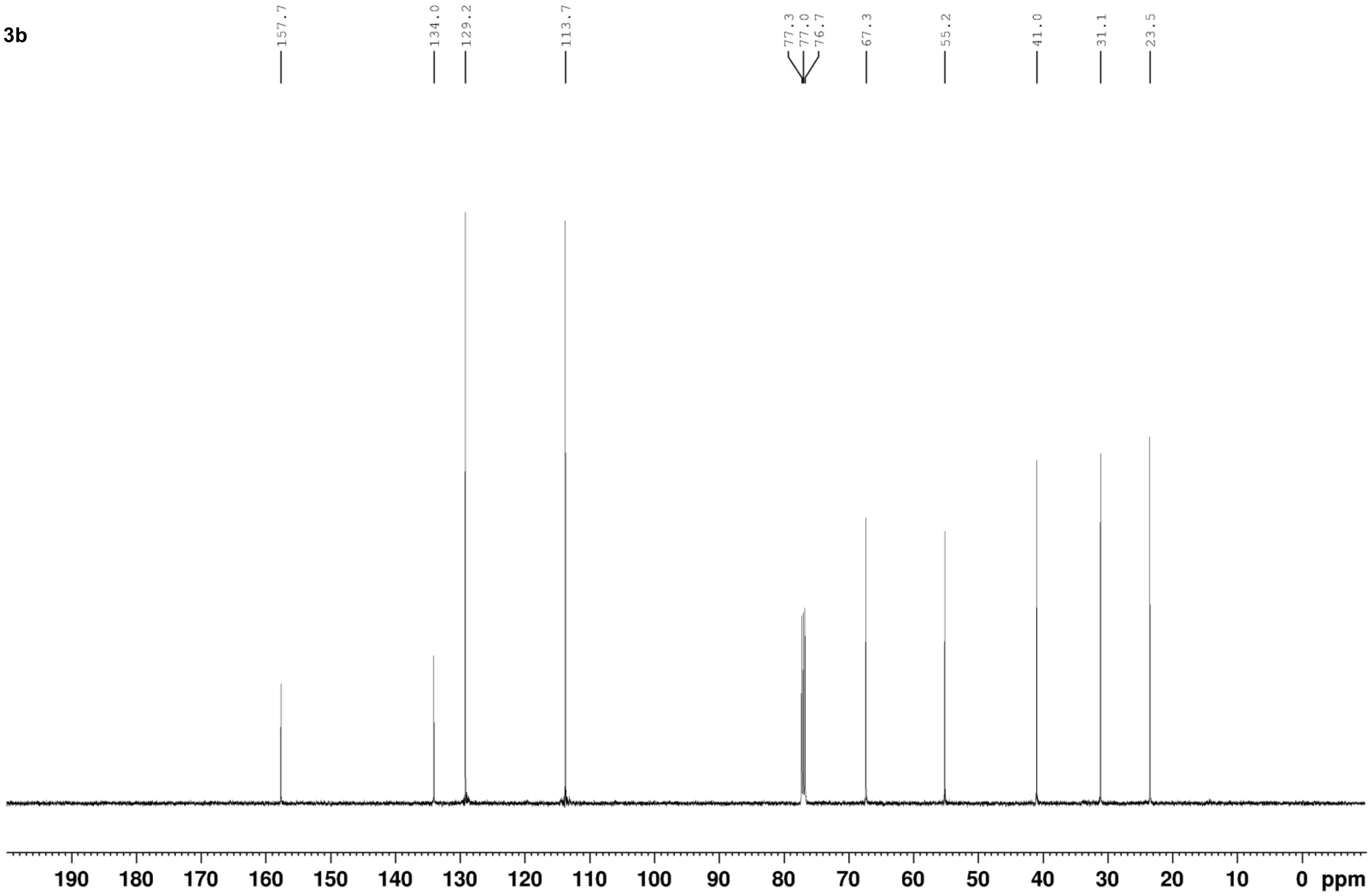

Figure S6. ^{1}H NMR (400 MHz, $CDCl_3$, 298K) of **3c**.

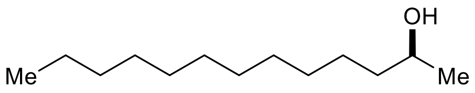

3c

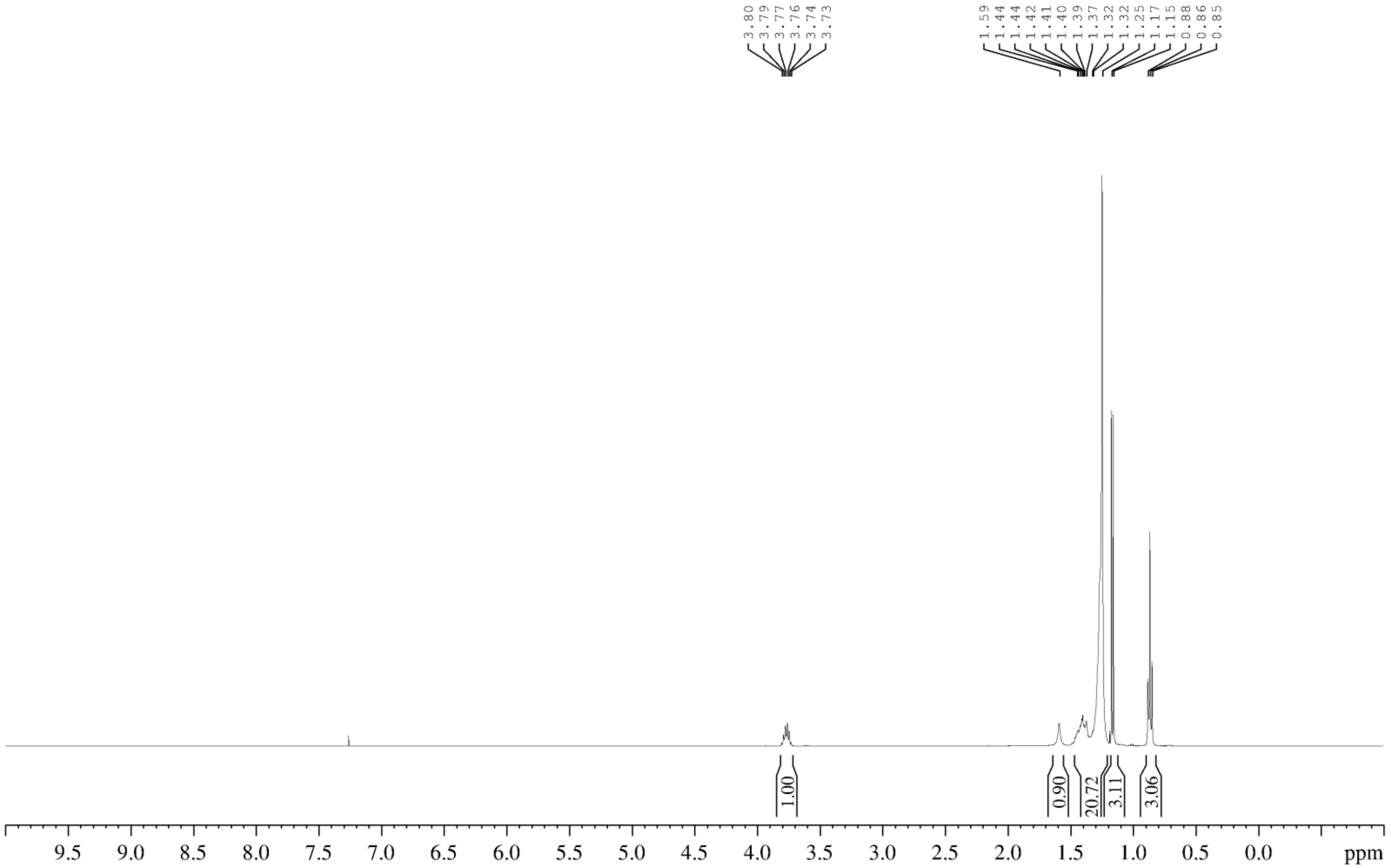

Figure S7. ^{13}C NMR (101 MHz, $CDCl_3$, 298K) of **3c**.

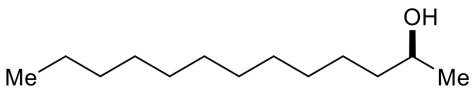

3c

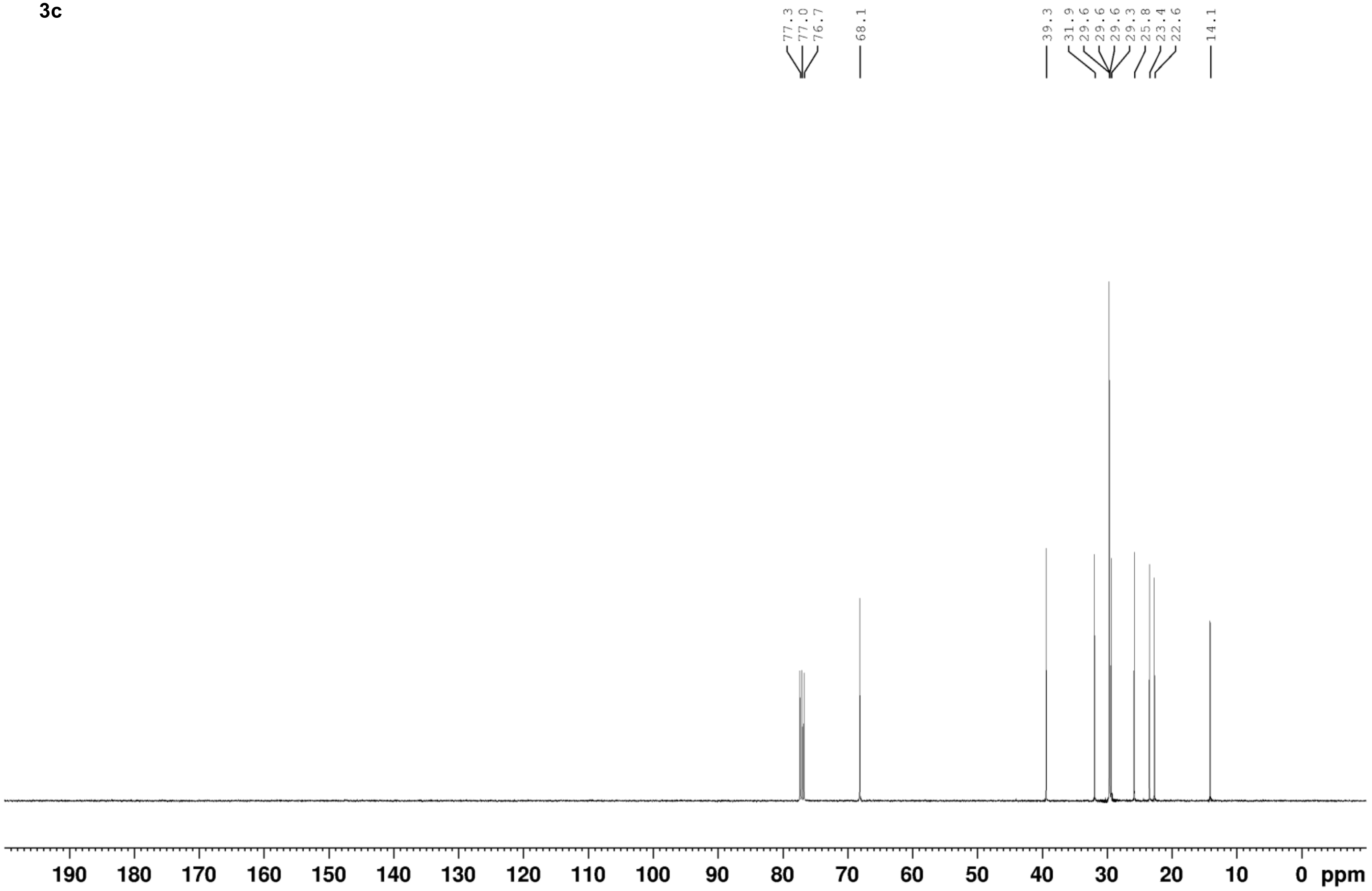

Figure S8. ^{1}H NMR (400 MHz, $CDCl_3$, 298K) of **3f**.

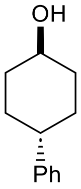

3f

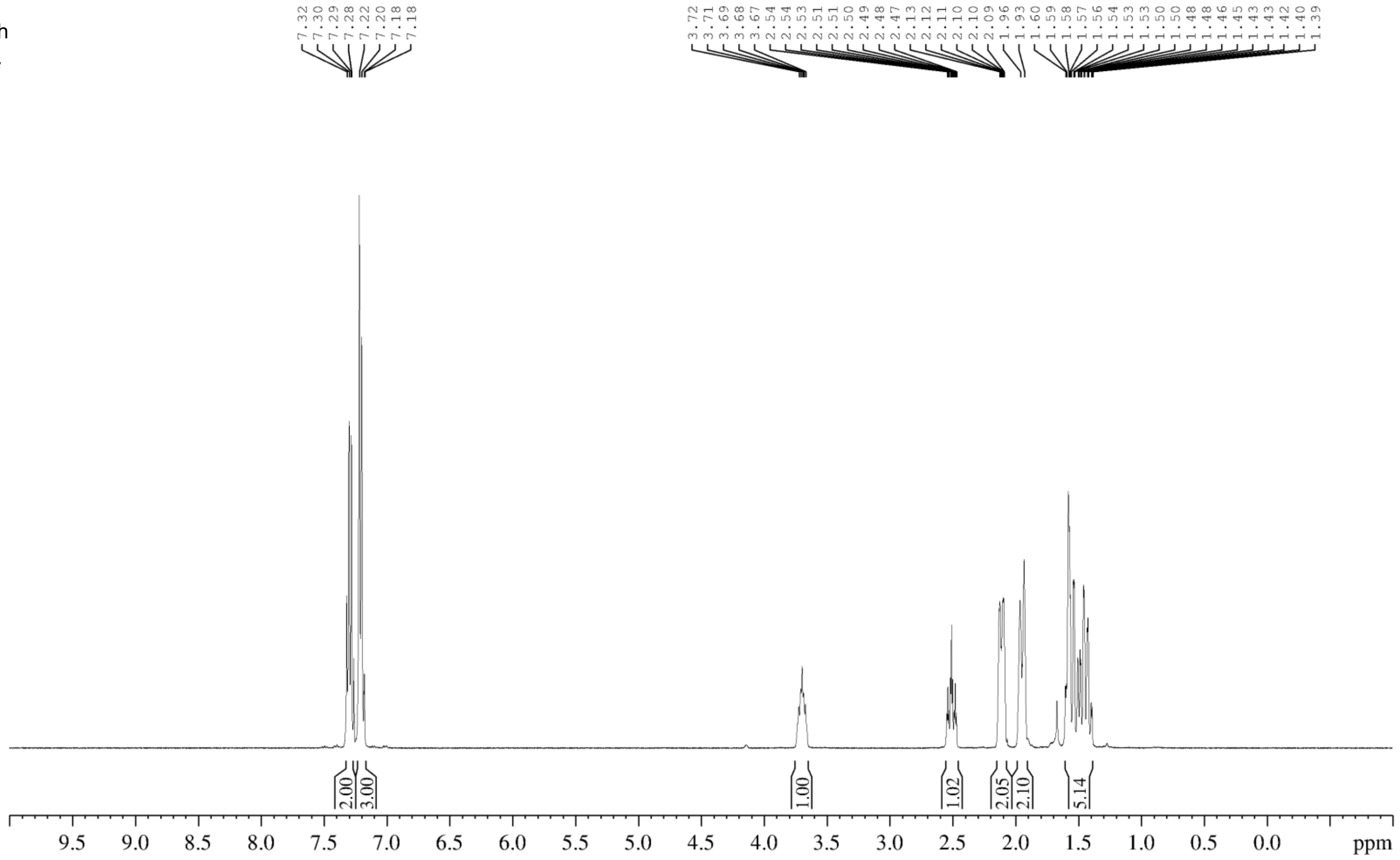

Figure S9. ^{13}C NMR (101 MHz, $CDCl_3$, 298K) of **3f**.

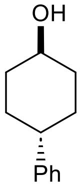

3f

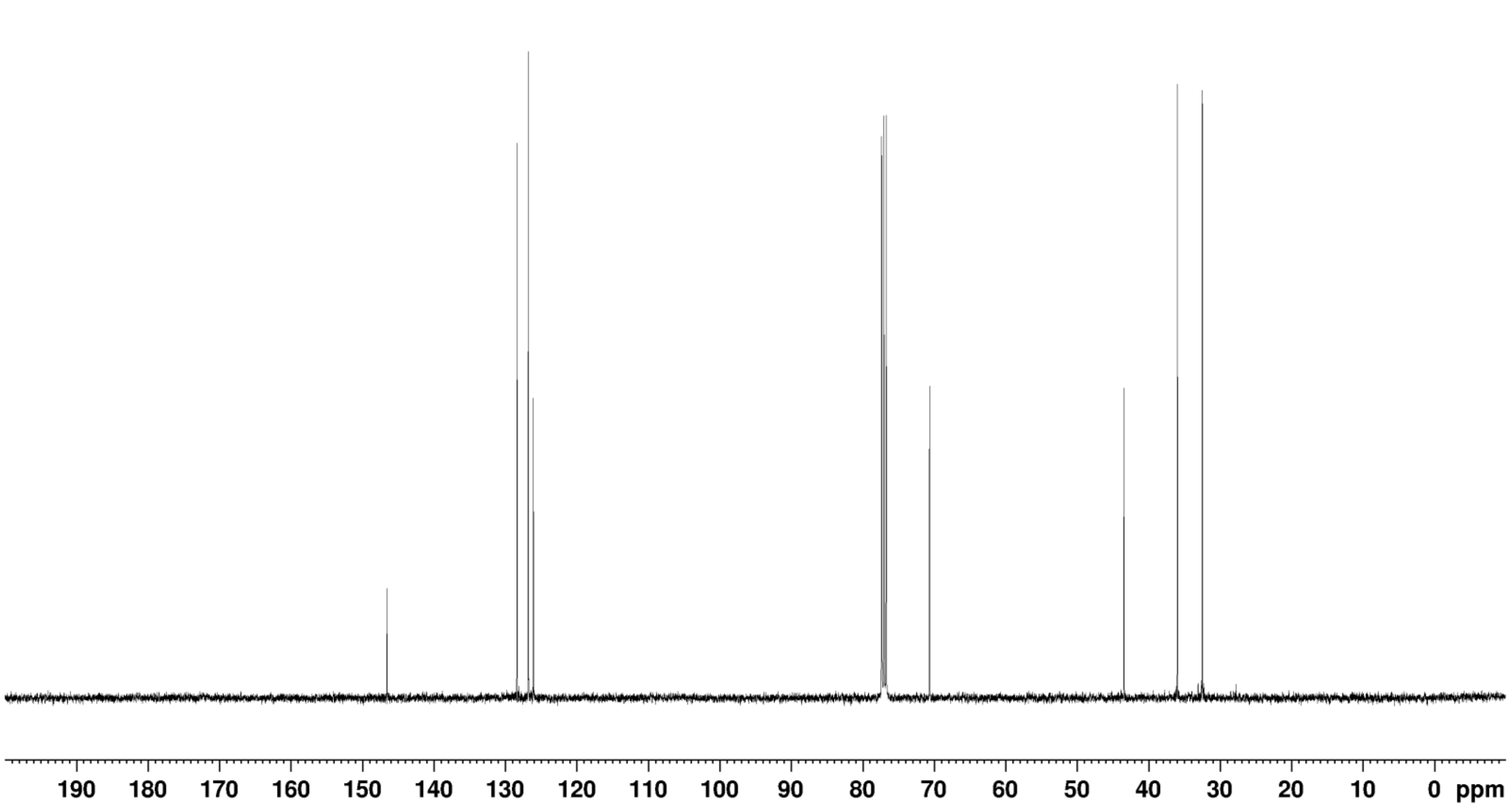

Figure S10. ^1H NMR (400 MHz, $CDCl_3$, 298 K) of **2a**.

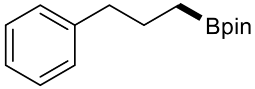

2a

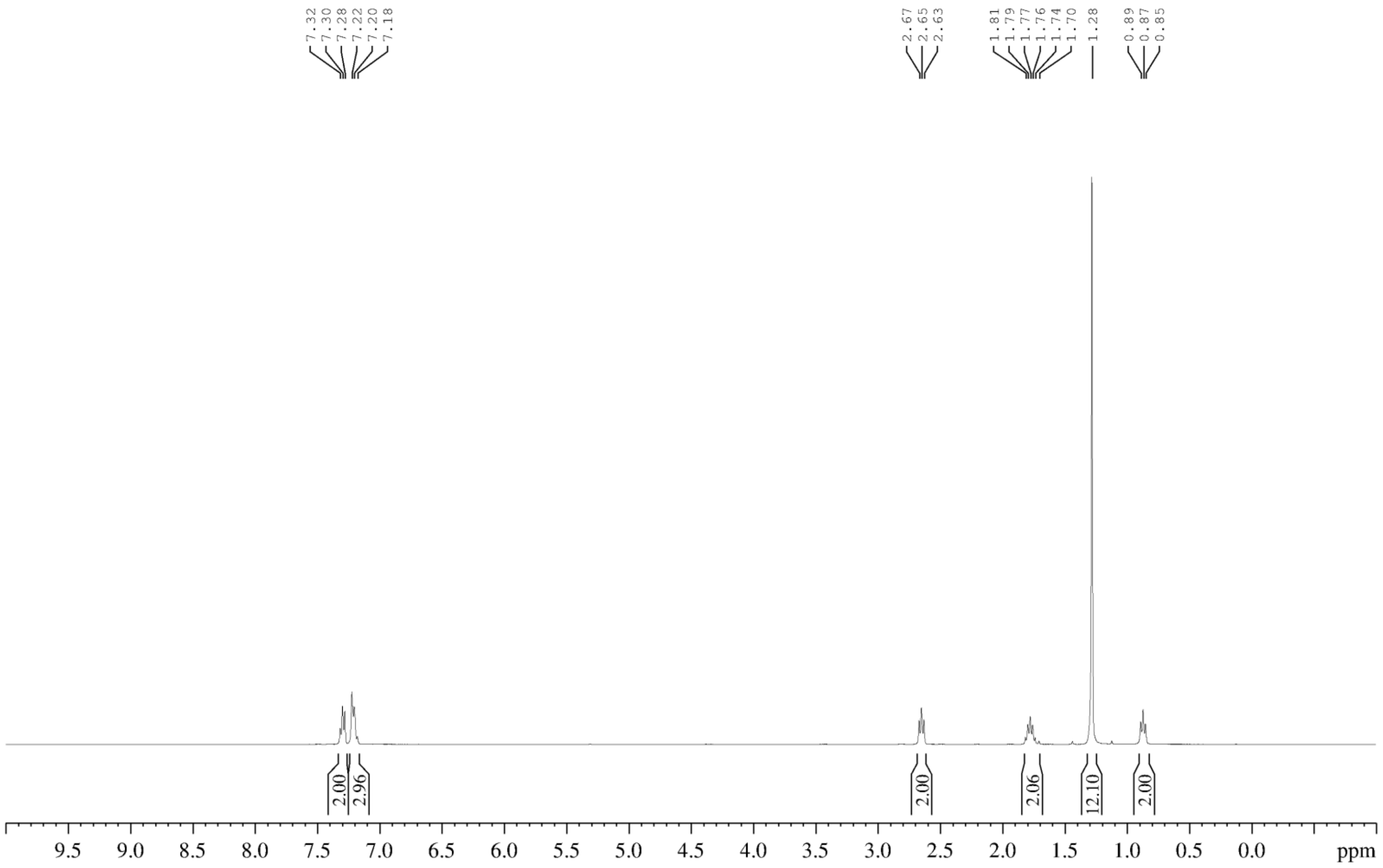

Figure S11. ^{13}C NMR (101 MHz, $CDCl_3$, 298 K) of **2a**.

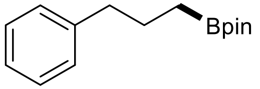

2a

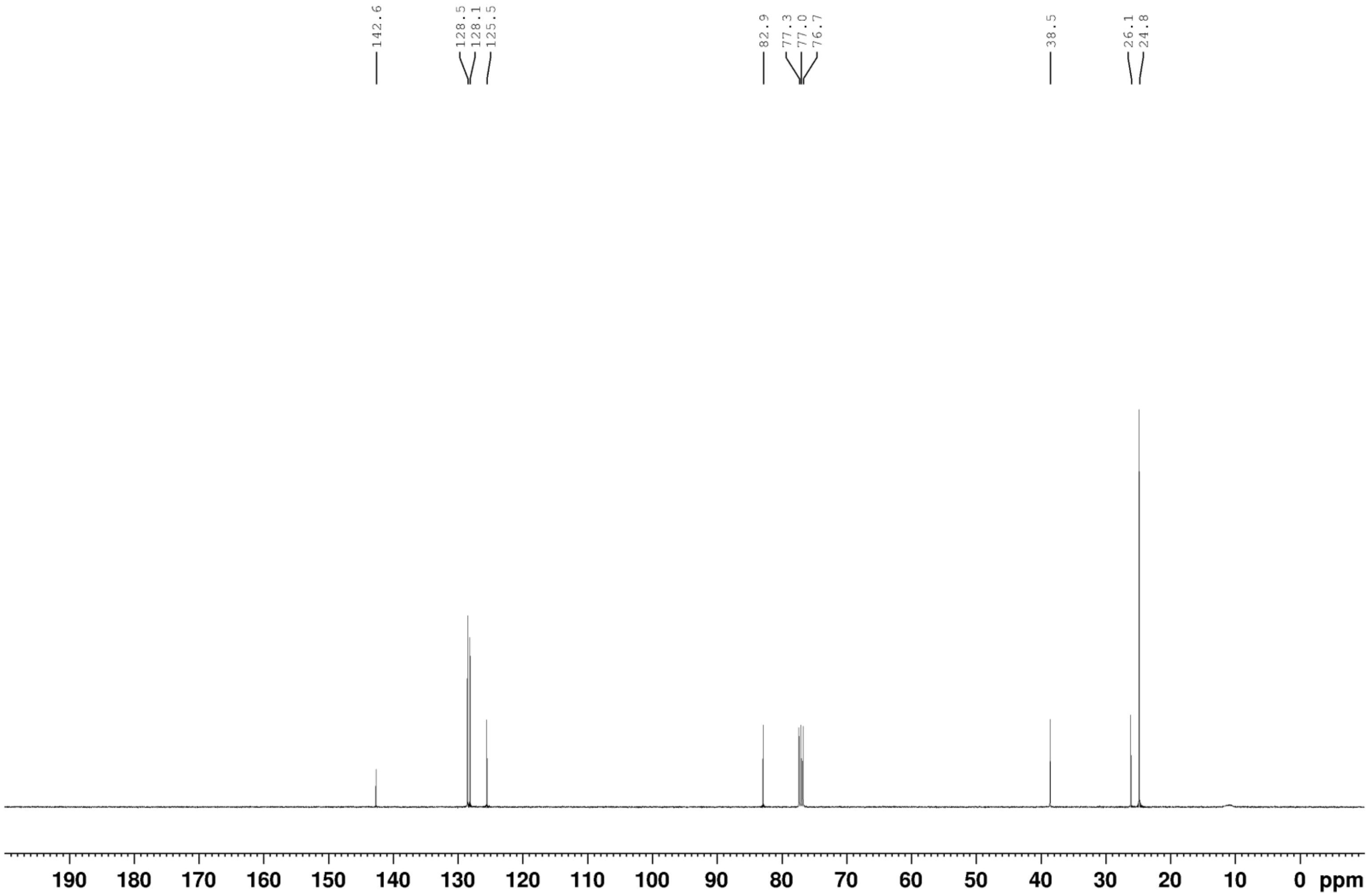

Figure S12. ^{11}B NMR (128 MHz, $CDCl_3$, 298 K) of **2a**.

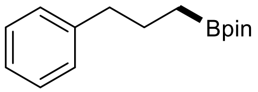

2a

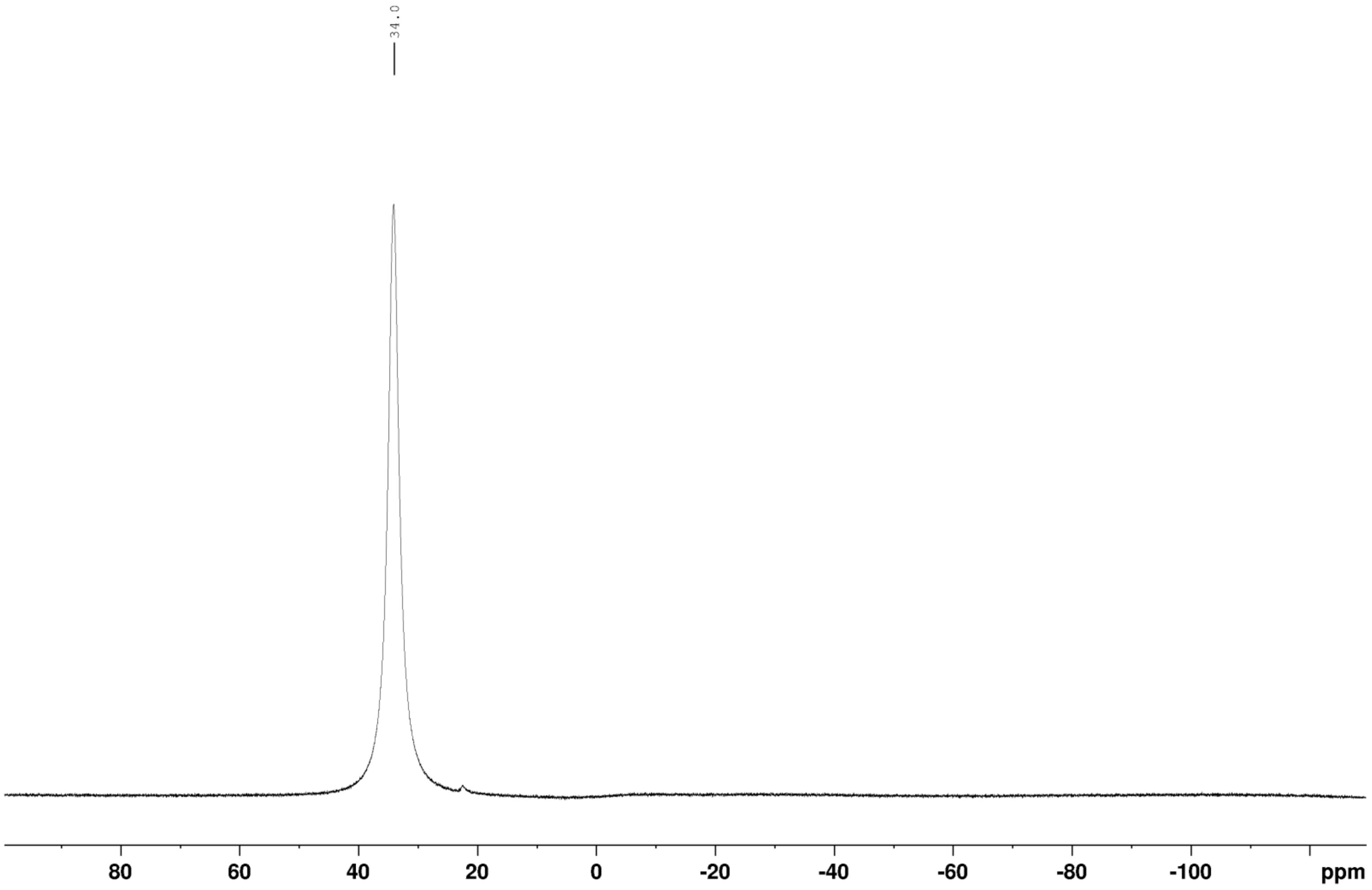

Figure S13. ^{1}H NMR (400 MHz, $CDCl_3$, 298 K) of **2b**.

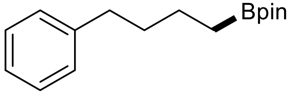

2b

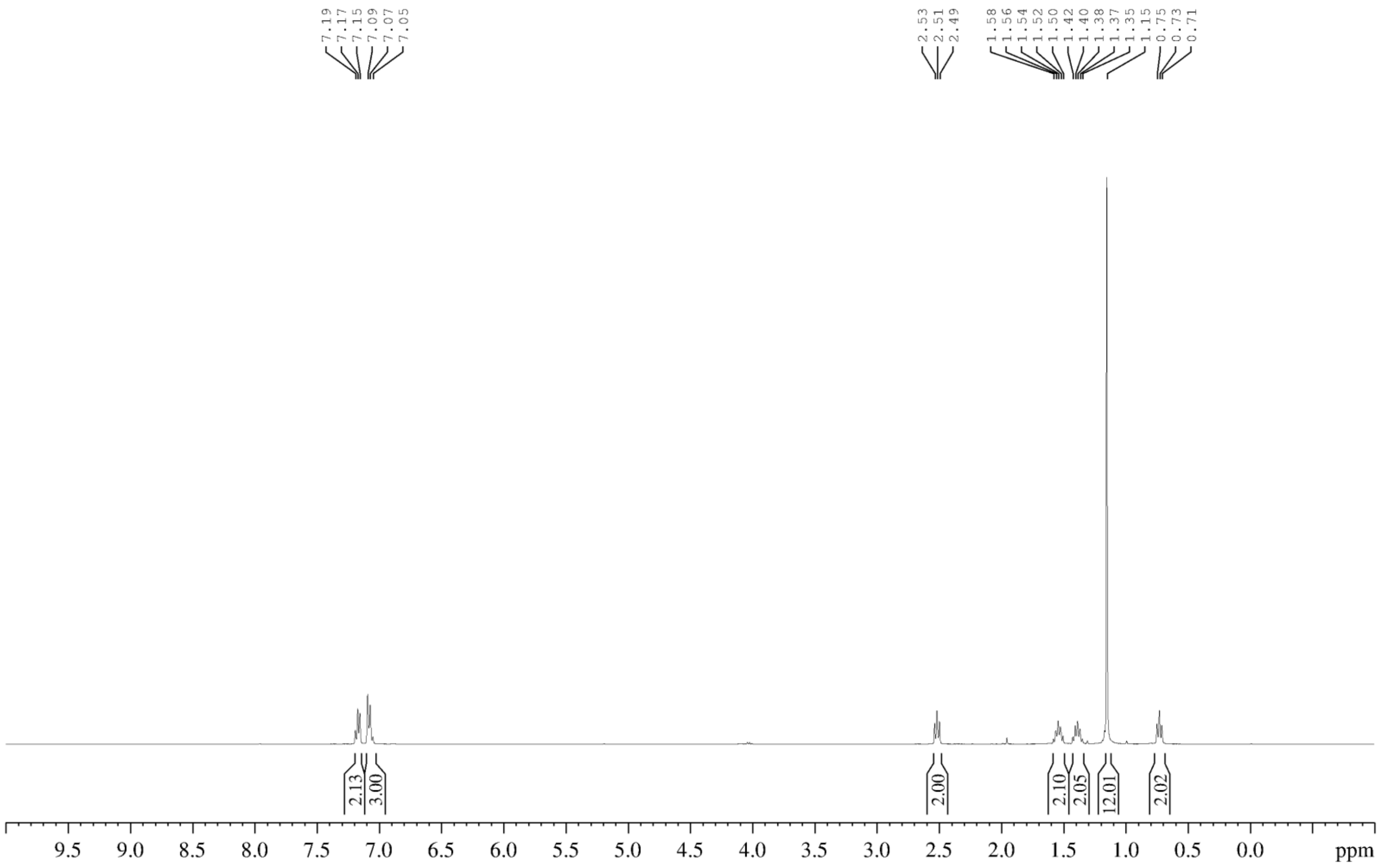

Figure S14. ^{13}C NMR (101 MHz, $CDCl_3$, 298 K) of **2b**.

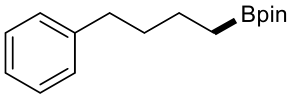

2b

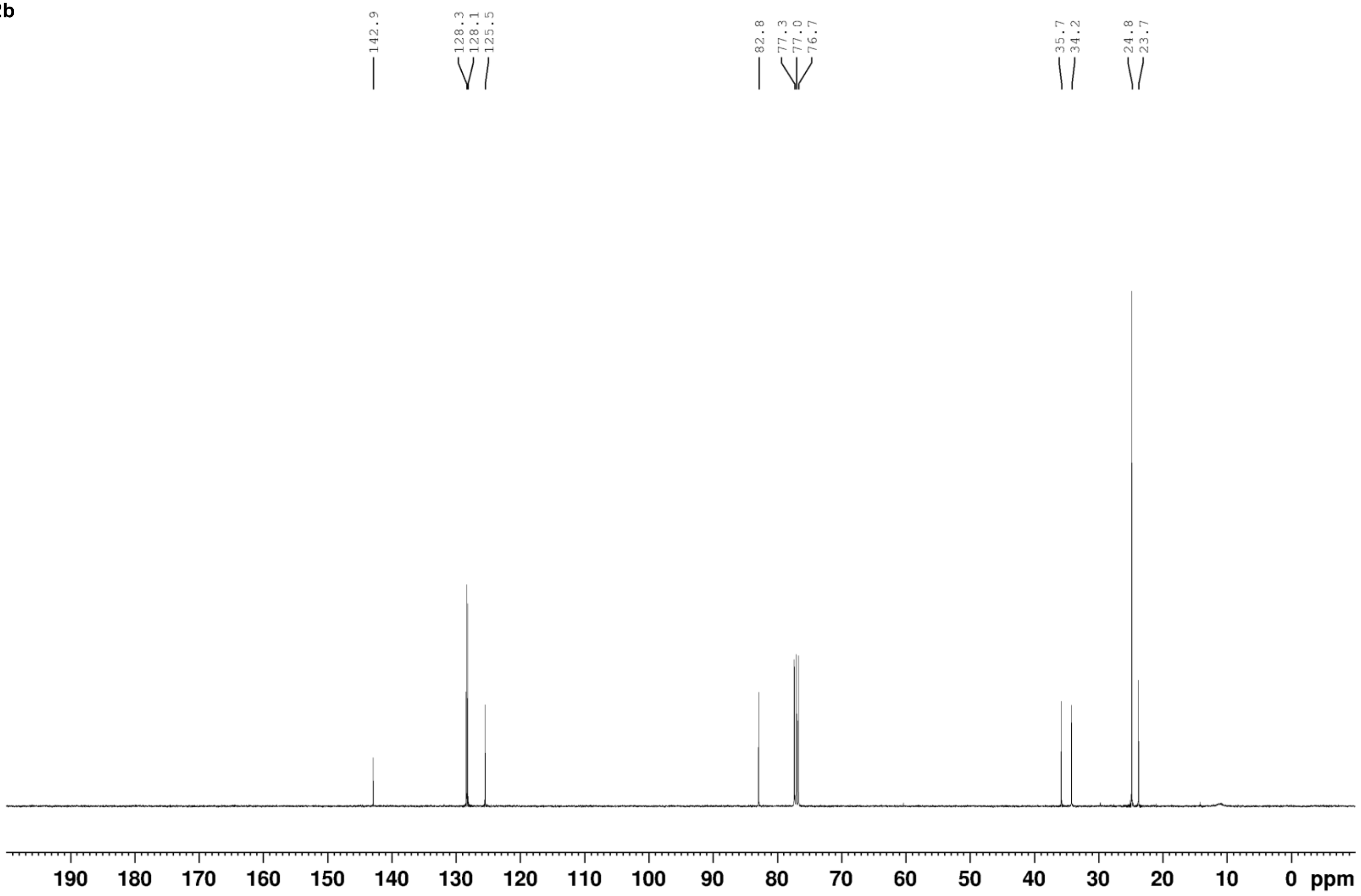

Figure S15. ^{11}B NMR (128 MHz, $CDCl_3$, 298 K) of **2b**.

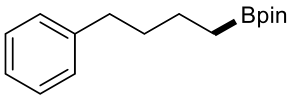

2b

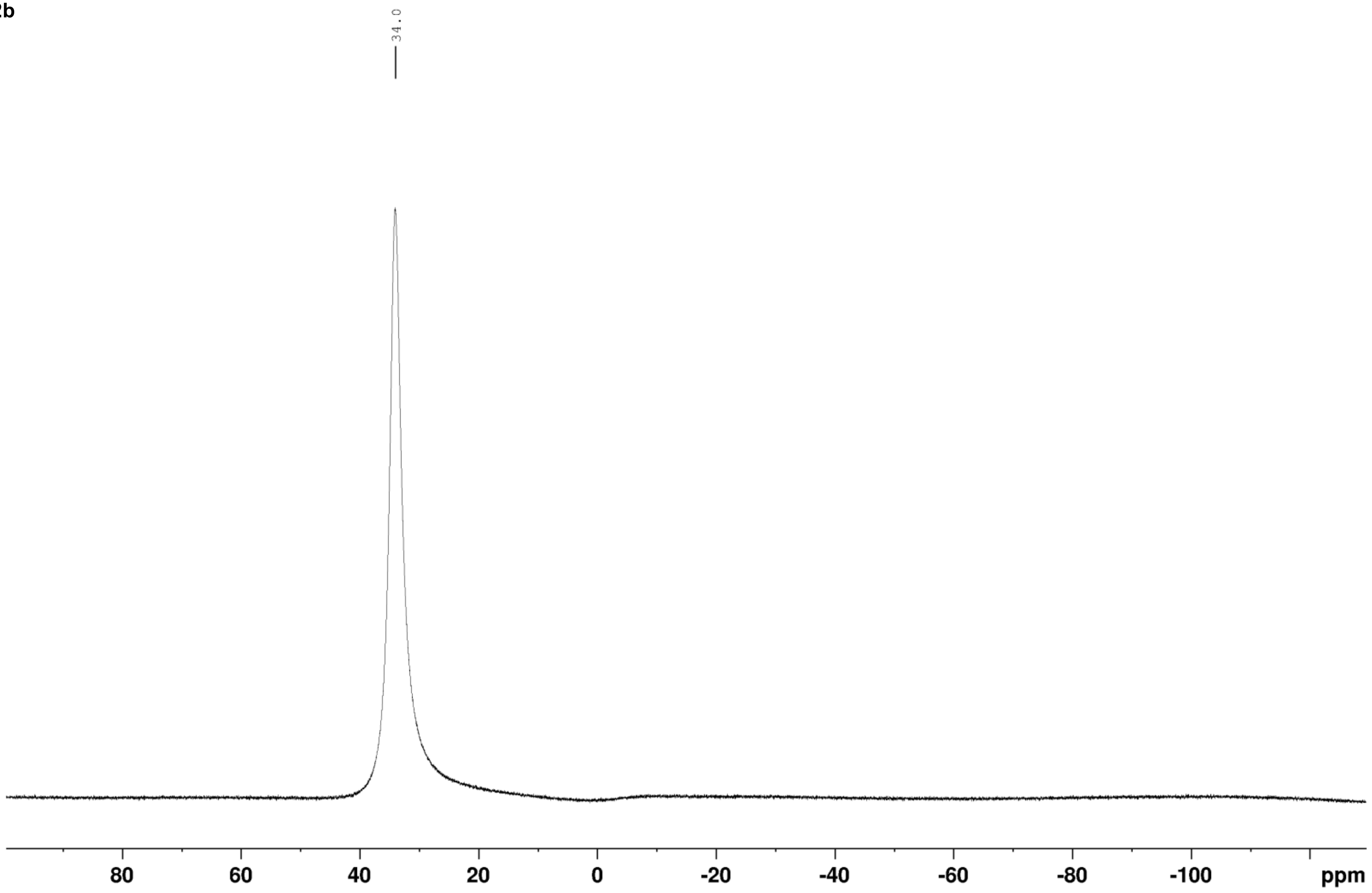

Figure S16. ^{1}H NMR (400 MHz, $CDCl_3$, 298 K) of **2c**.

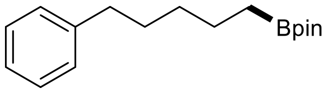

2c

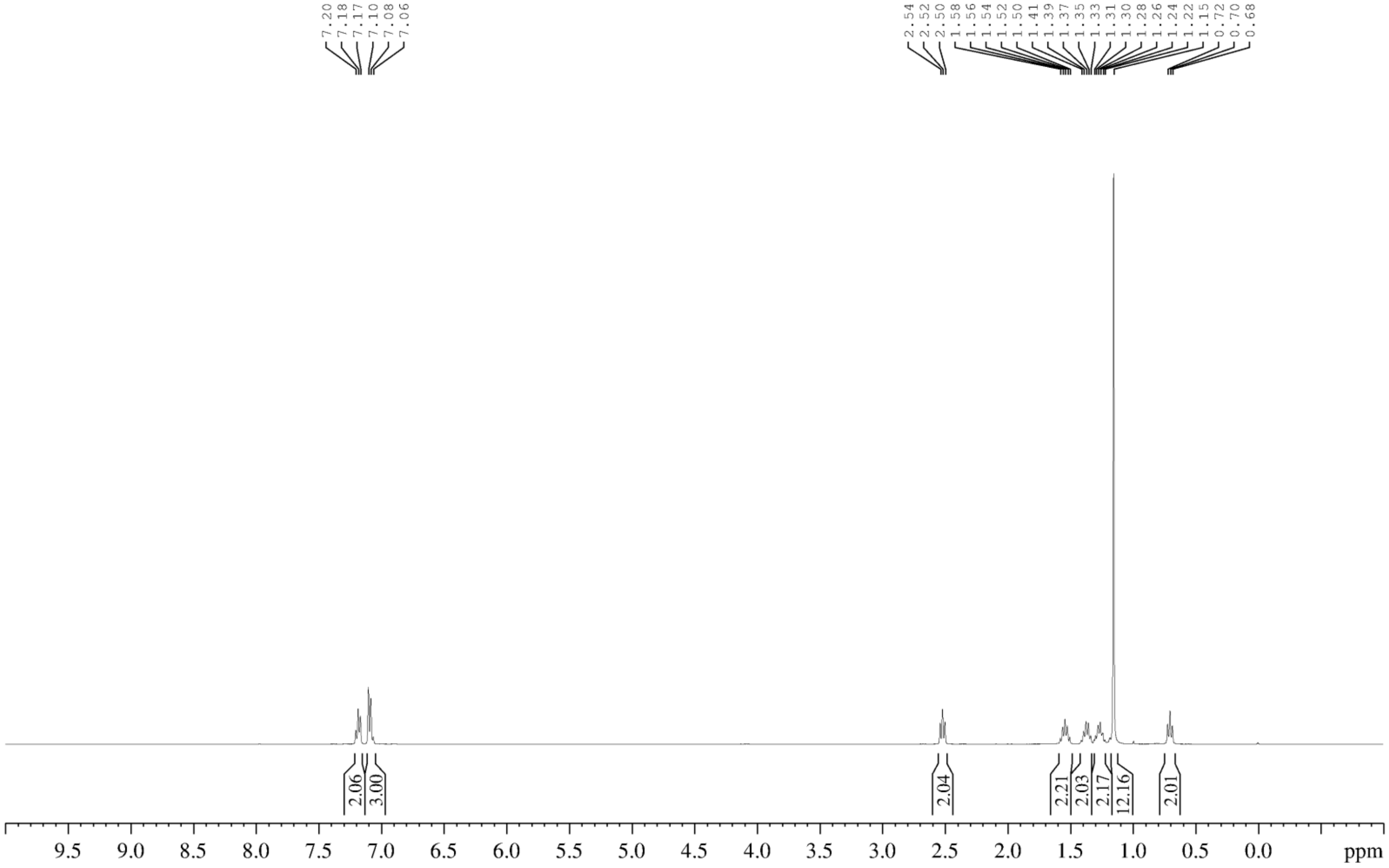

Figure S17. ^{13}C NMR (101 MHz, $CDCl_3$, 298 K) of **2c**.

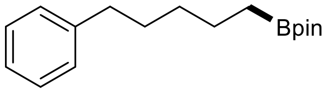

2c

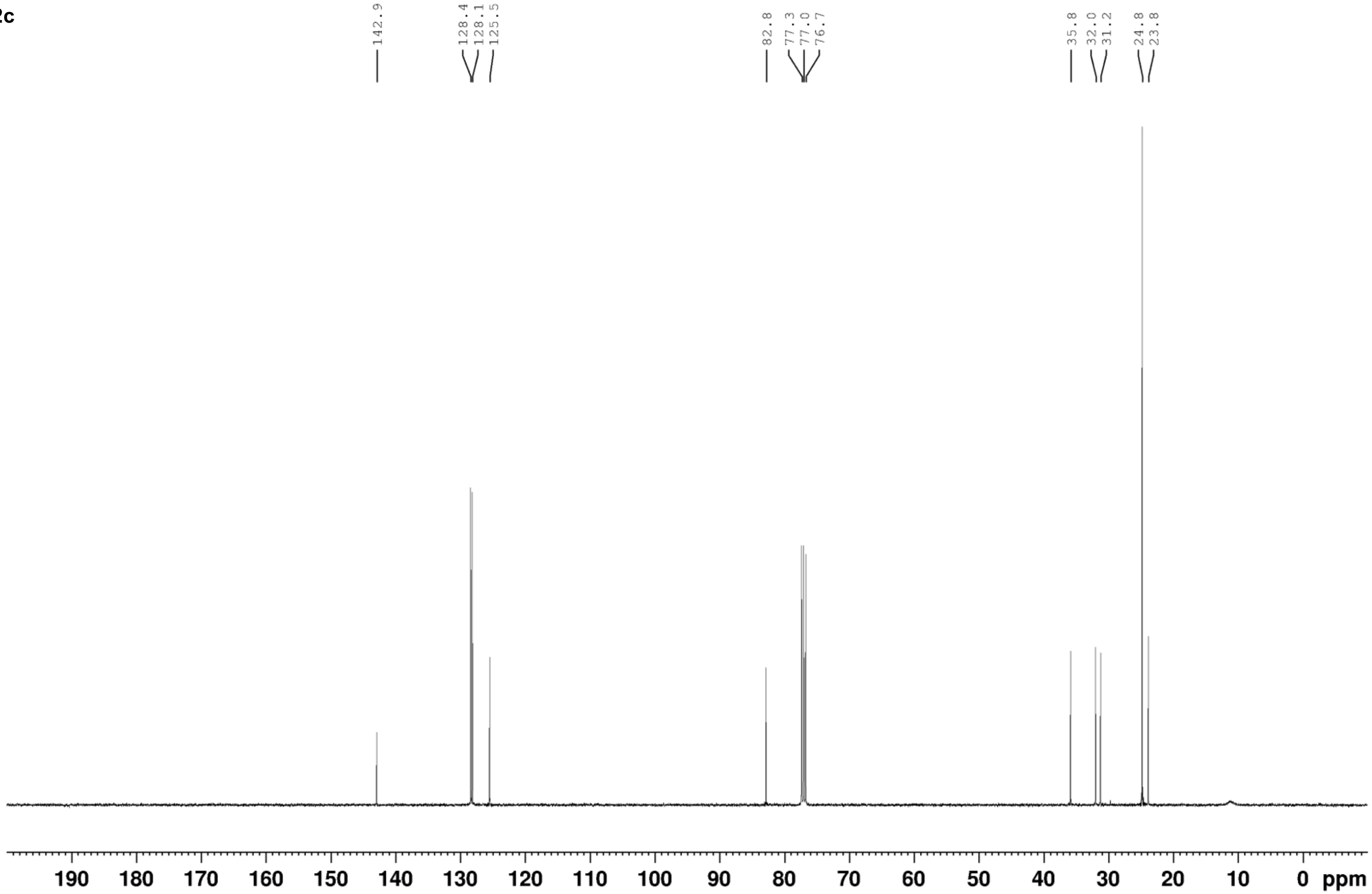

Figure S18. ^{11}B NMR (128 MHz, $CDCl_3$, 298 K) of **2c**.

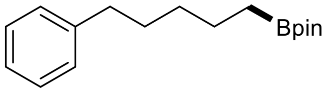

2c

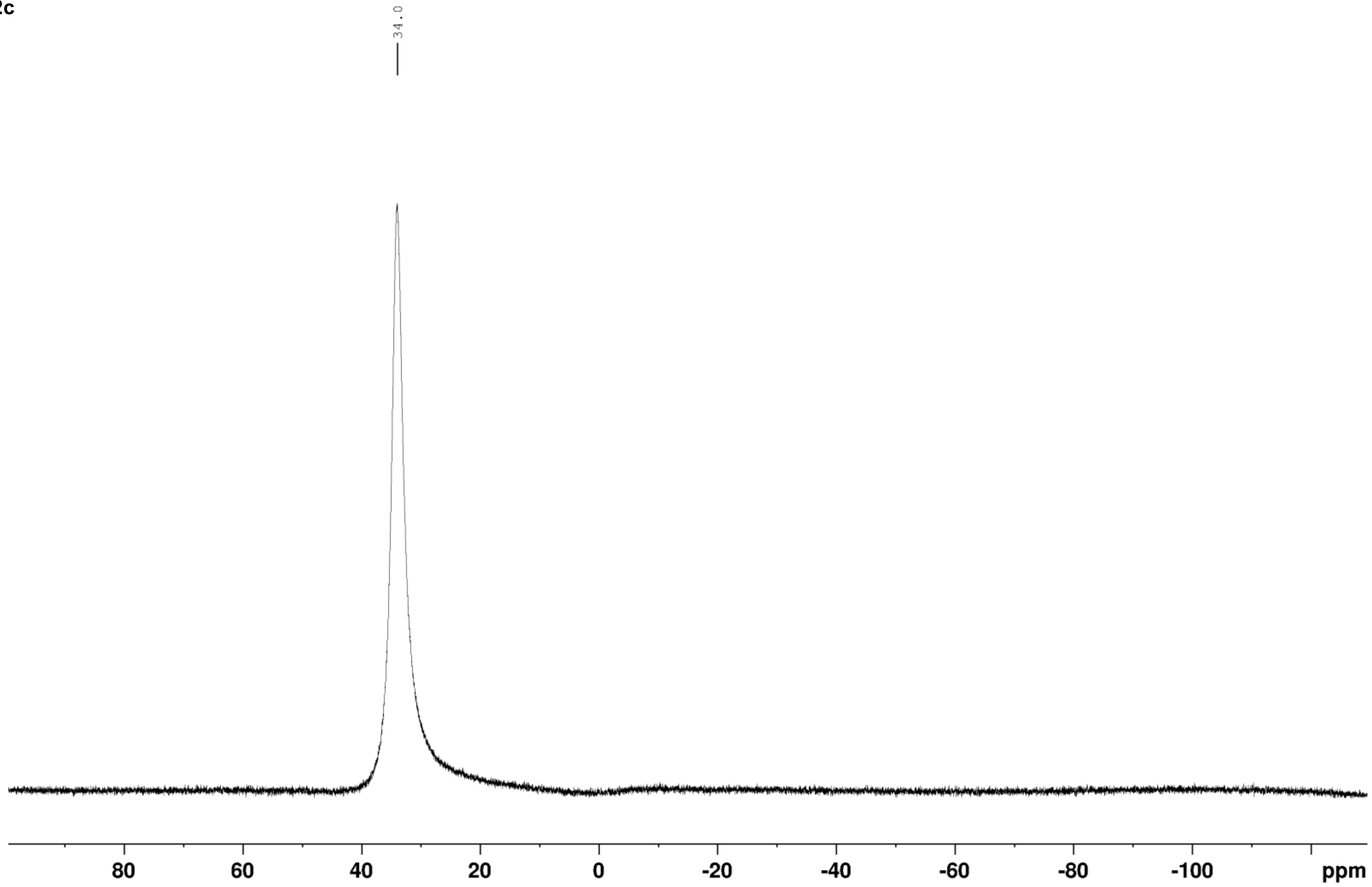

Figure S19. ¹H NMR (400 MHz, $CDCl_3$, 298 K) of **2d**.

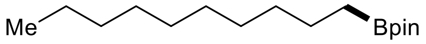

2d

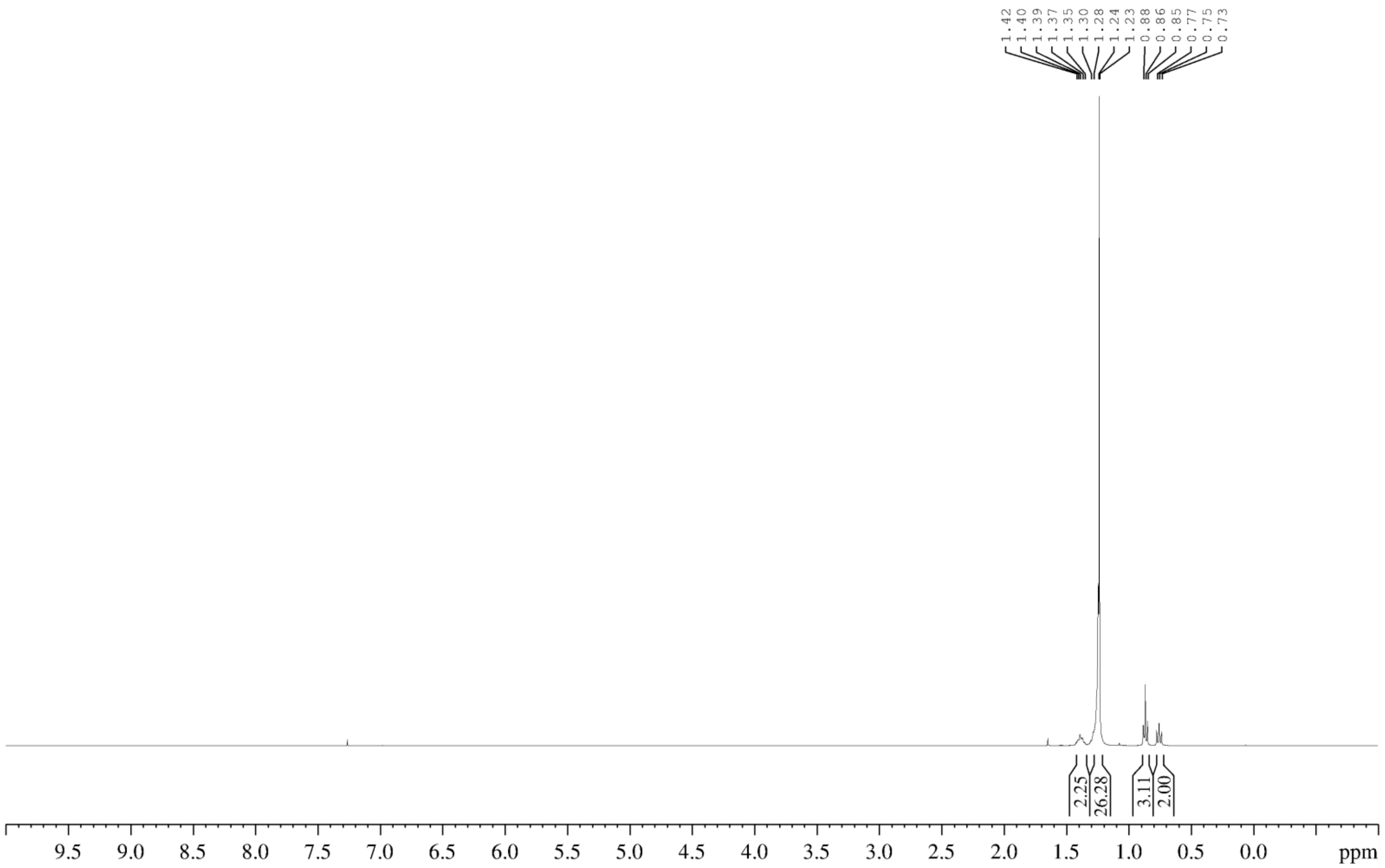

Figure S20. ^{13}C NMR (101 MHz, $CDCl_3$, 298 K) of **2d**.

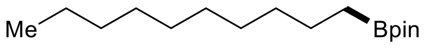

2d

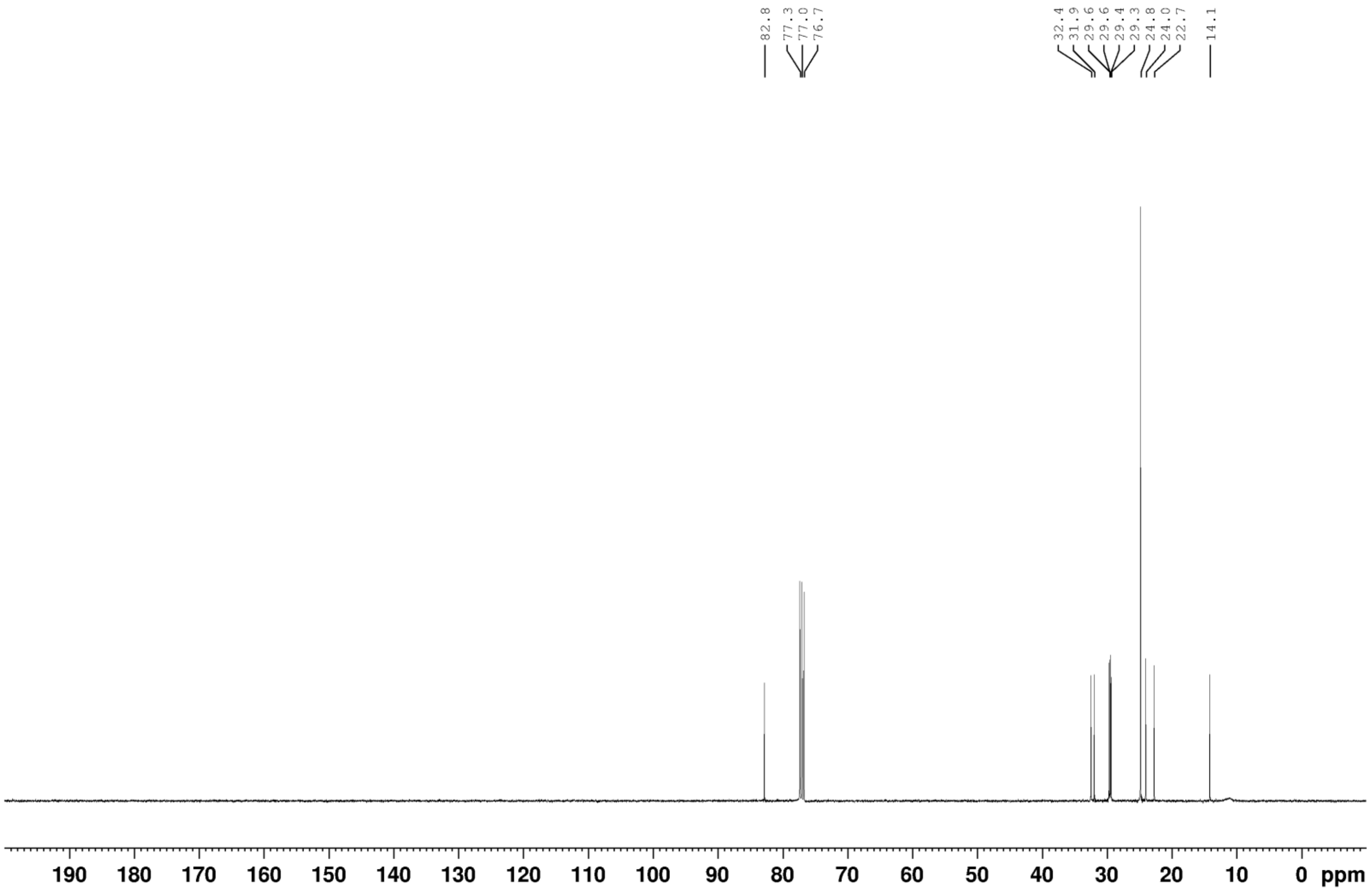

Figure S21. ^{11}B NMR (128 MHz, $CDCl_3$, 298 K) of **2d**.

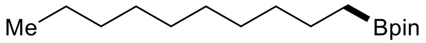

2d

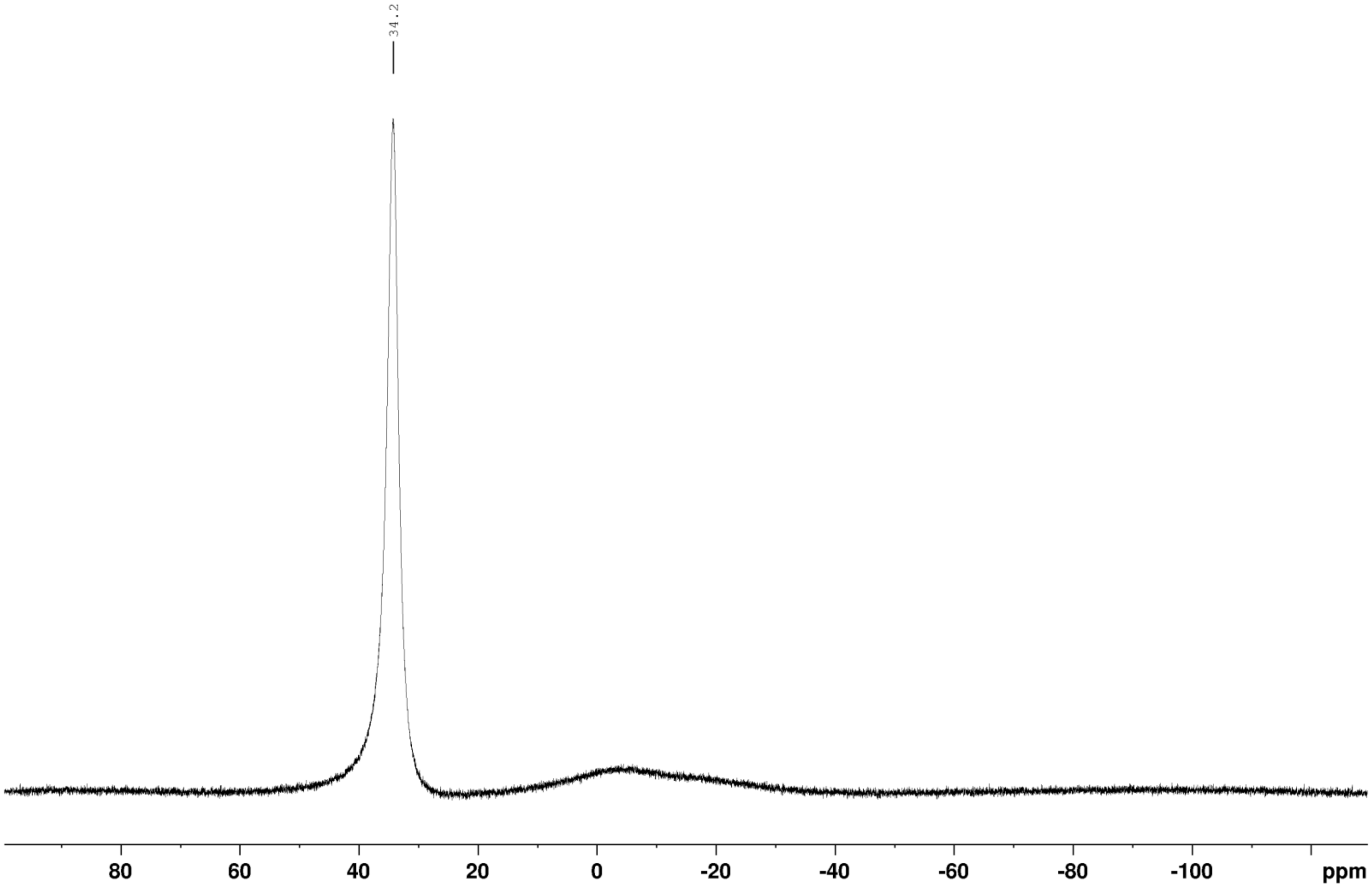

Figure S22. ^{1}H NMR (400 MHz, $CDCl_3$, 298 K) of **2e**.

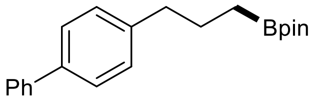

2e

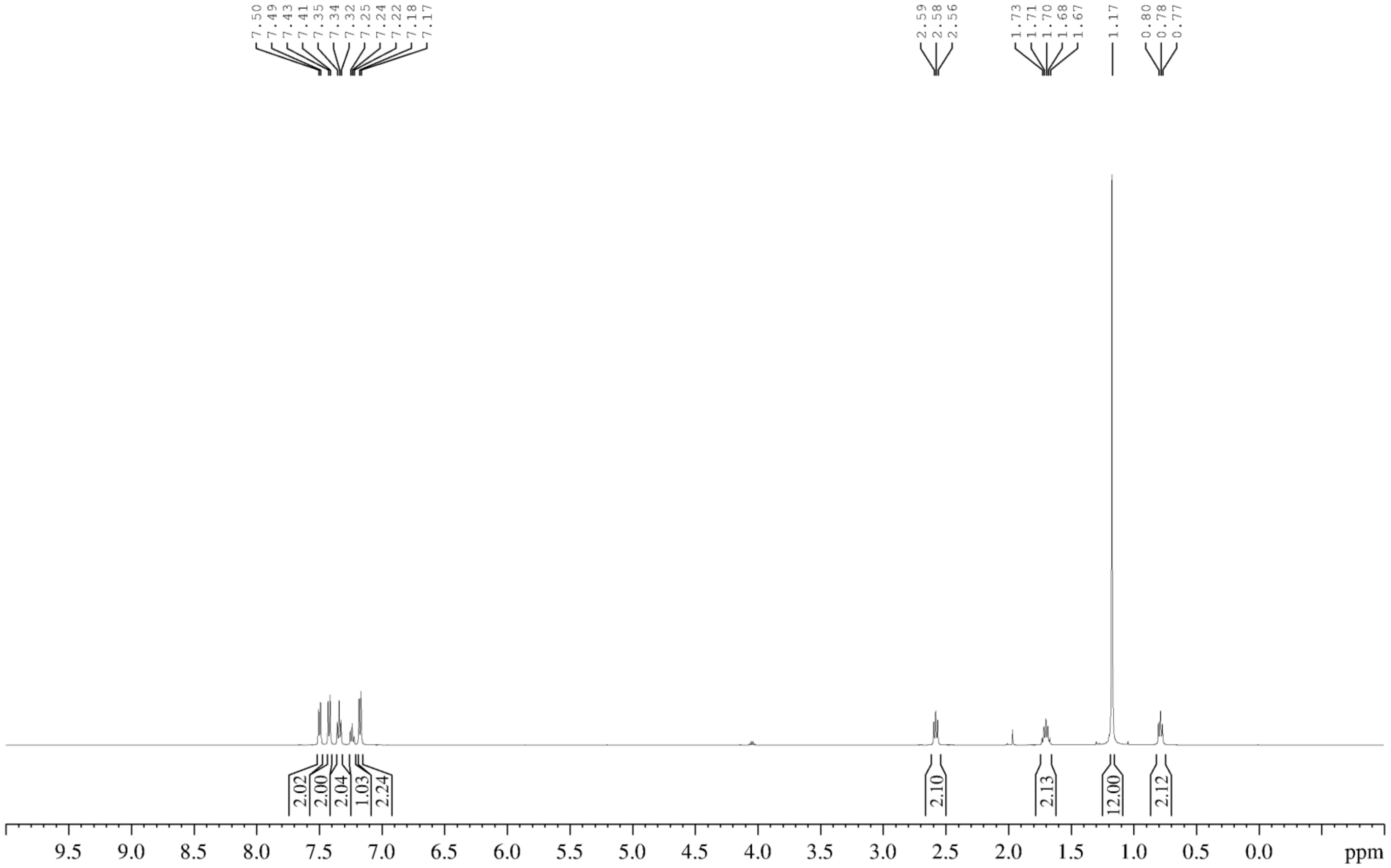

Figure S23. ^{13}C NMR (101 MHz, $CDCl_3$, 298 K) of **2e**.

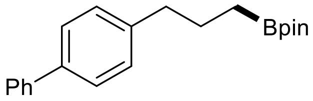

2e

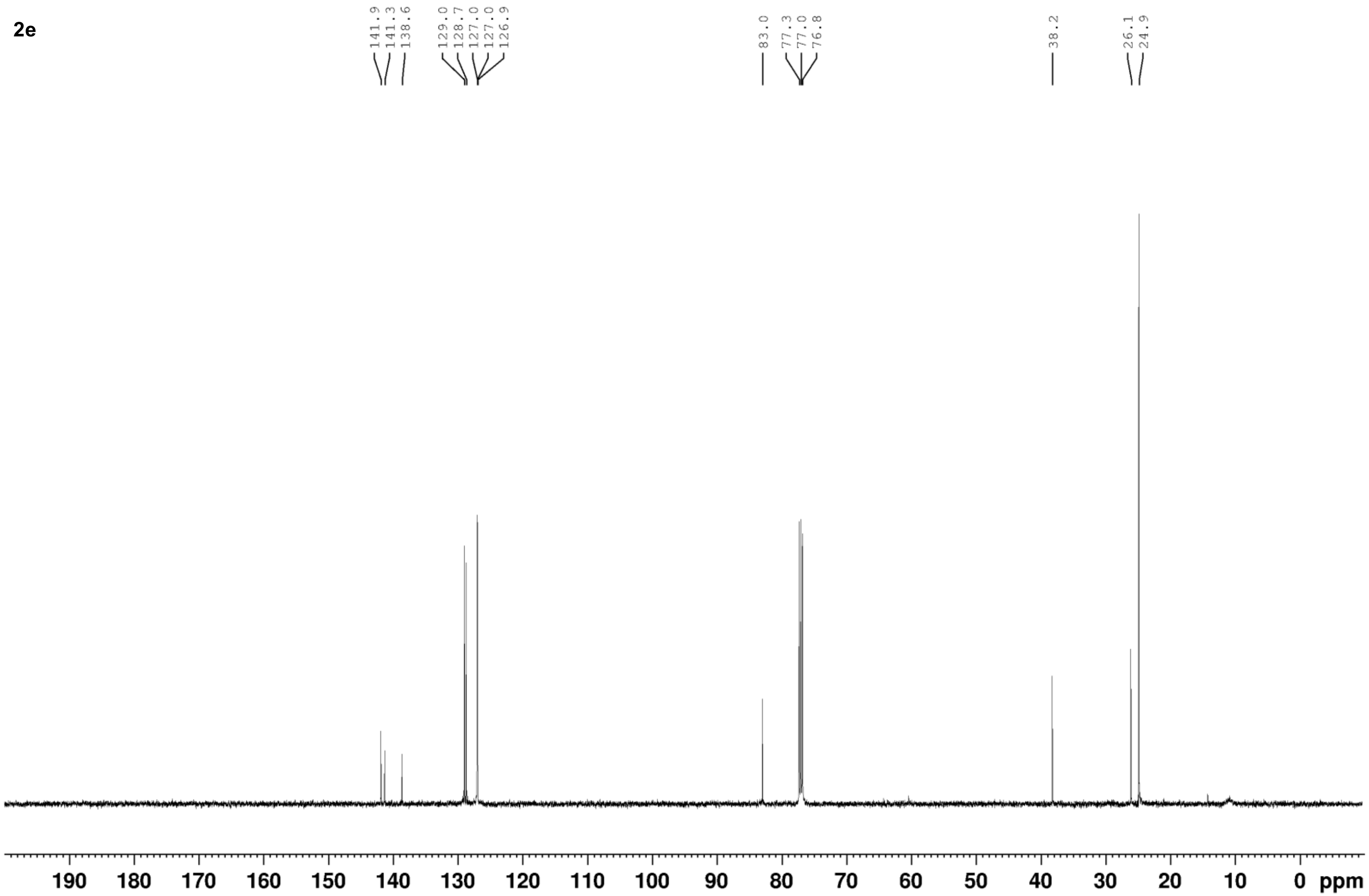

Figure S24. ^{11}B NMR (128 MHz, $CDCl_3$, 298 K) of **2e**.

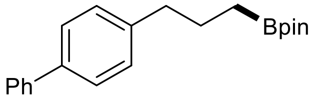

2e

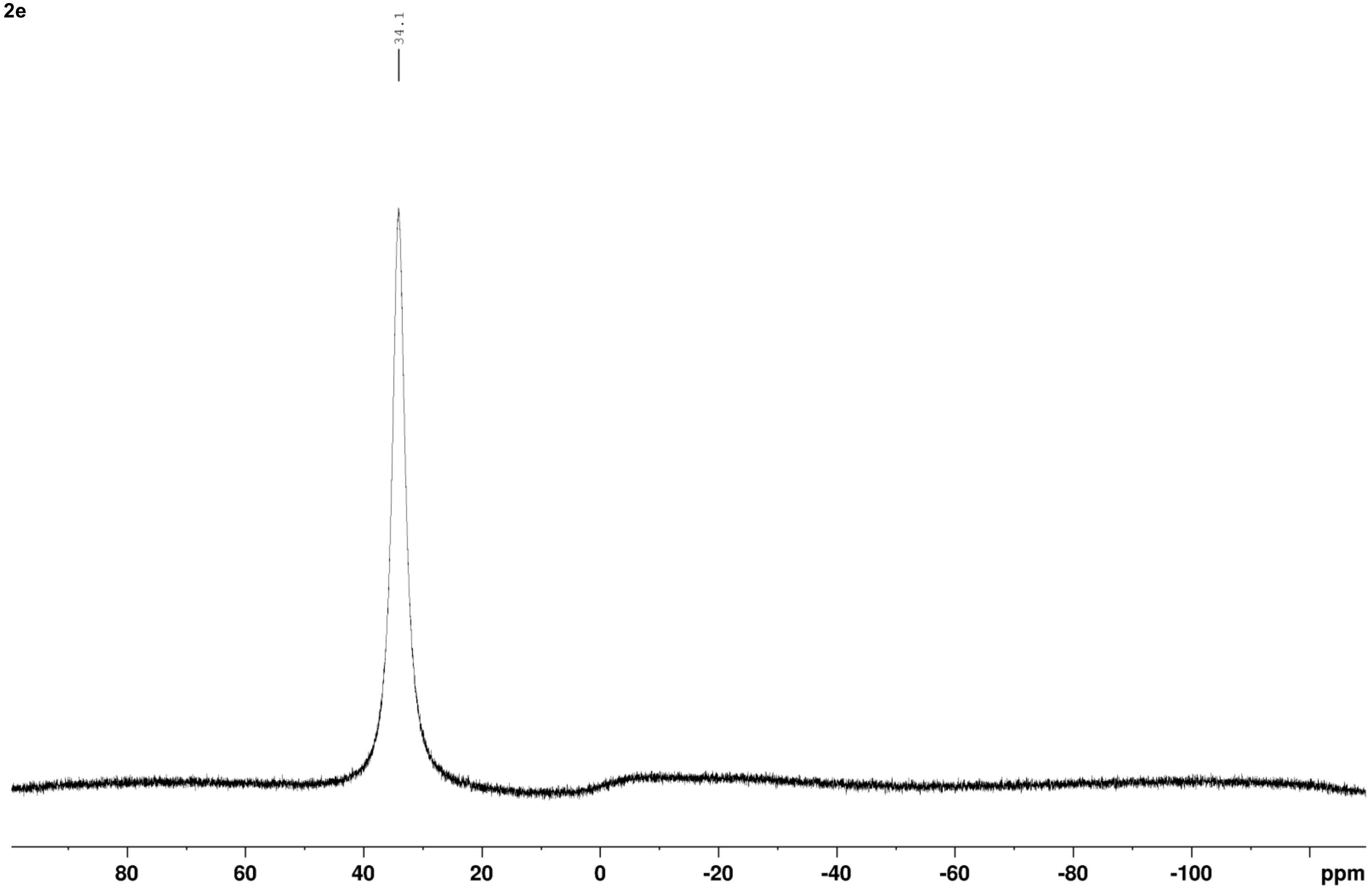

Figure S25. ^{1}H NMR (400 MHz, $CDCl_3$, 298 K) of **2f**.

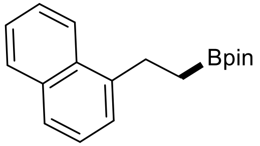

2f

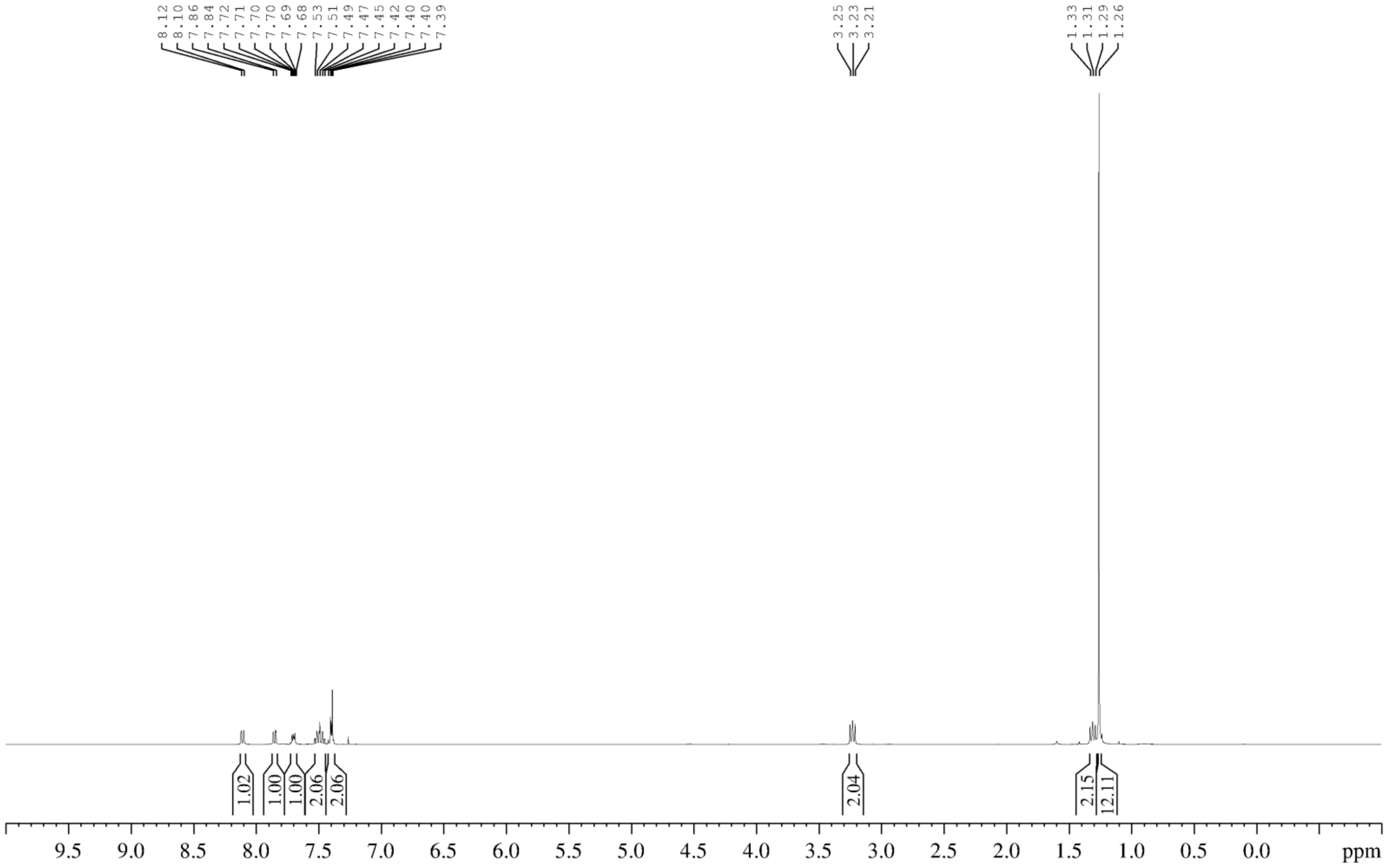

Figure S26. ^{13}C NMR (101 MHz, $CDCl_3$, 298 K) of **2f**.

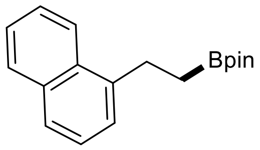

2f

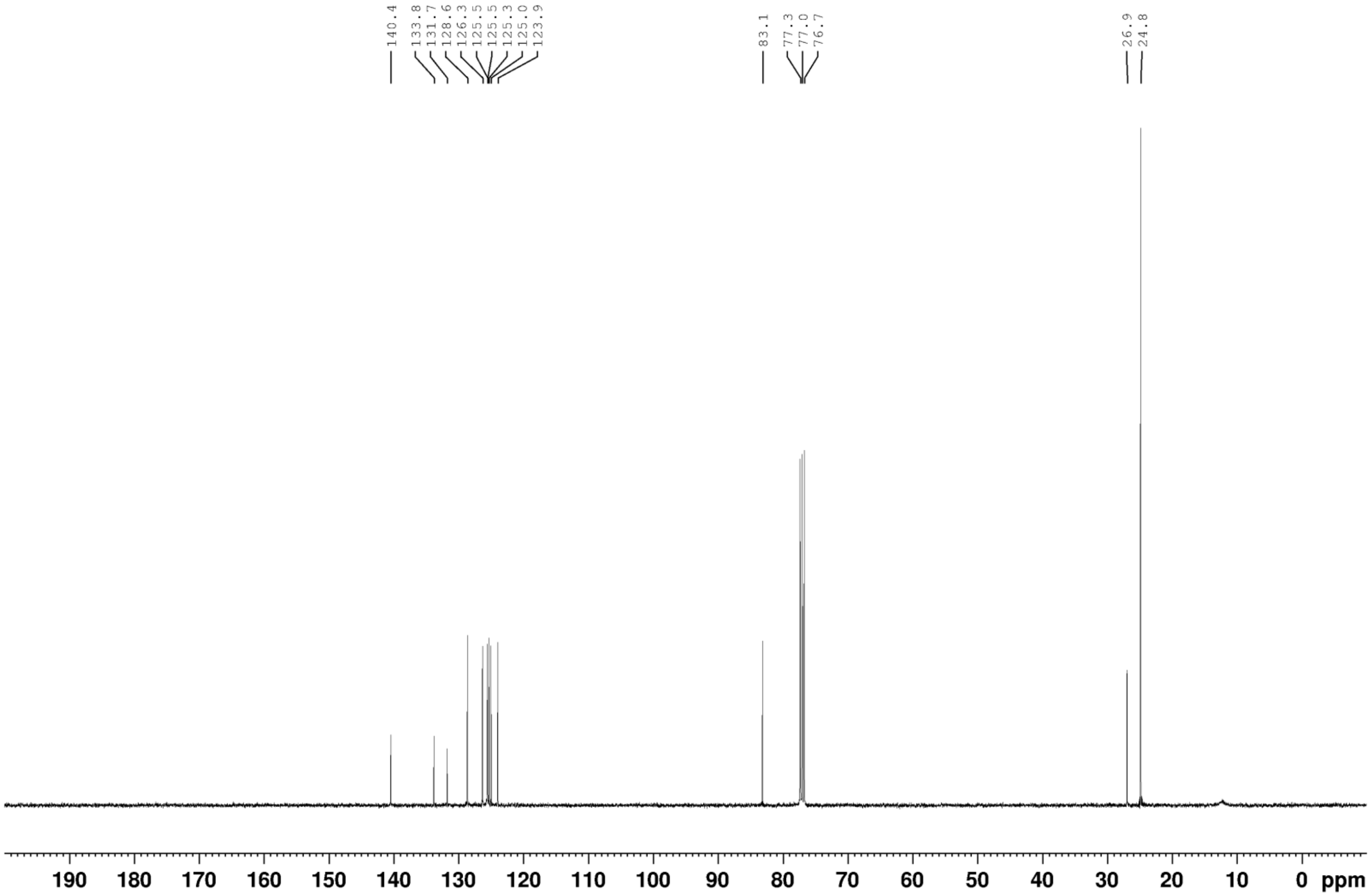

Figure S27. ^{11}B NMR (128 MHz, $CDCl_3$, 298 K) of **2f**.

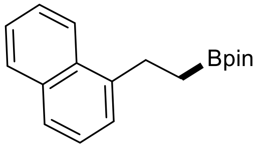

2f

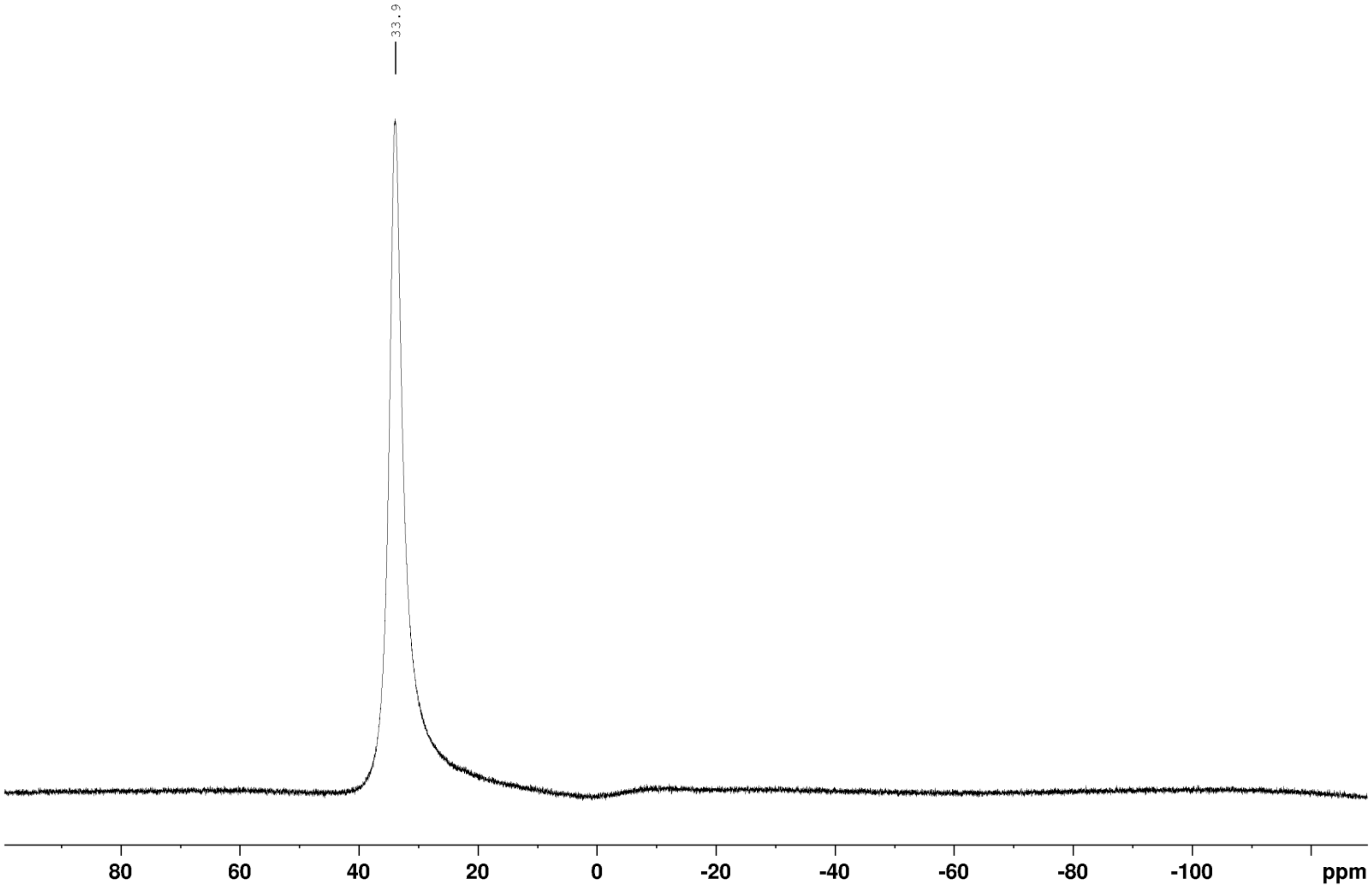

Figure S28. ^{1}H NMR (400 MHz, $CDCl_3$, 298 K) of **2g**.

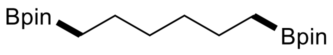

2g

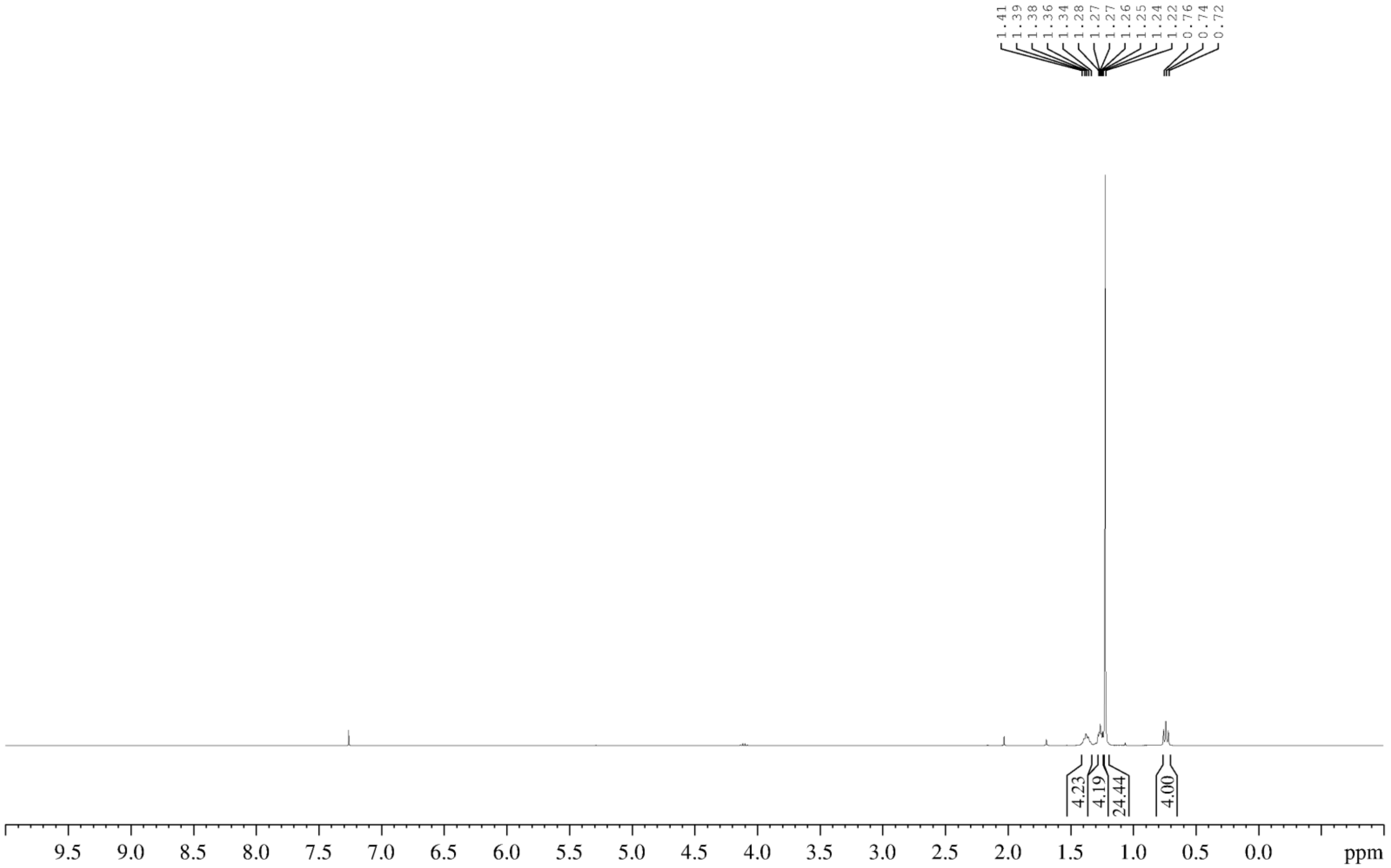

Figure S29. ^{13}C NMR (101 MHz, $CDCl_3$, 298 K) of **2g**.

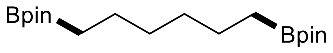

2g

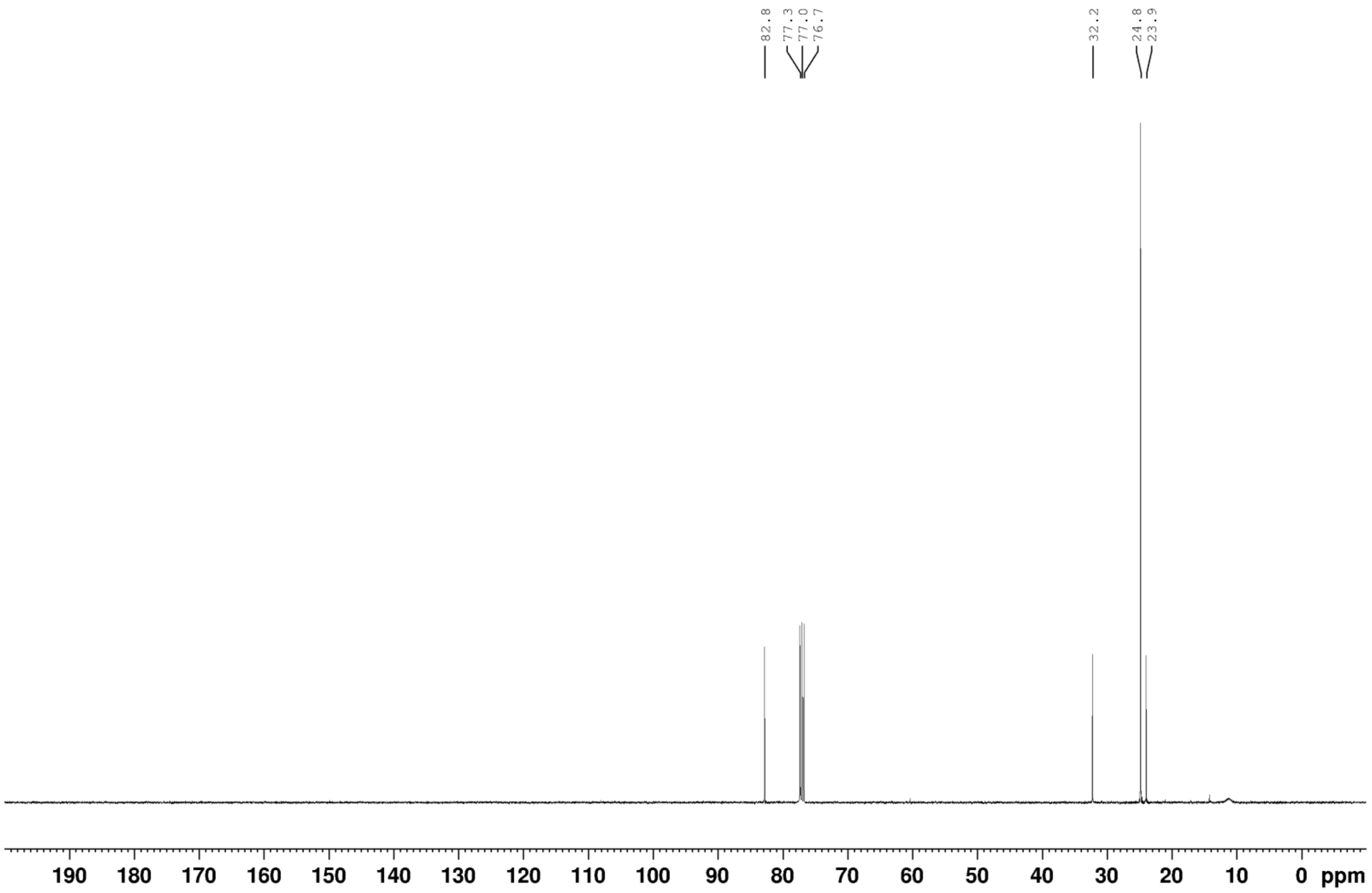

Figure S30. ^{11}B NMR (128 MHz, $CDCl_3$, 298 K) of **2g**.

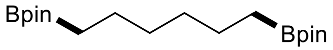

2g

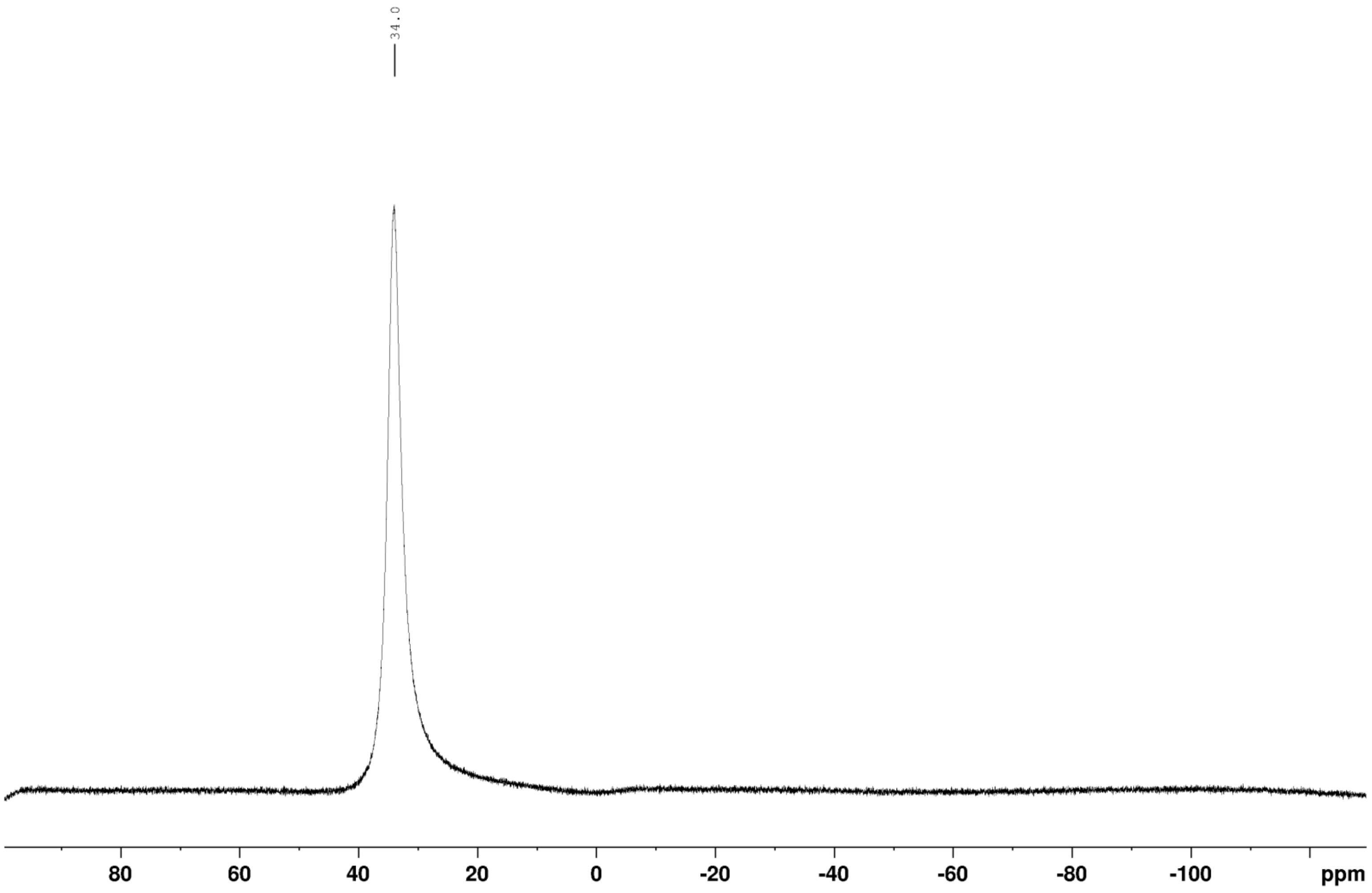

Figure S31. ^{1}H NMR (400 MHz, $CDCl_3$, 298 K) of **2h** (*Z*).

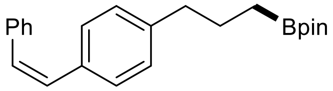

2h (*Z*)

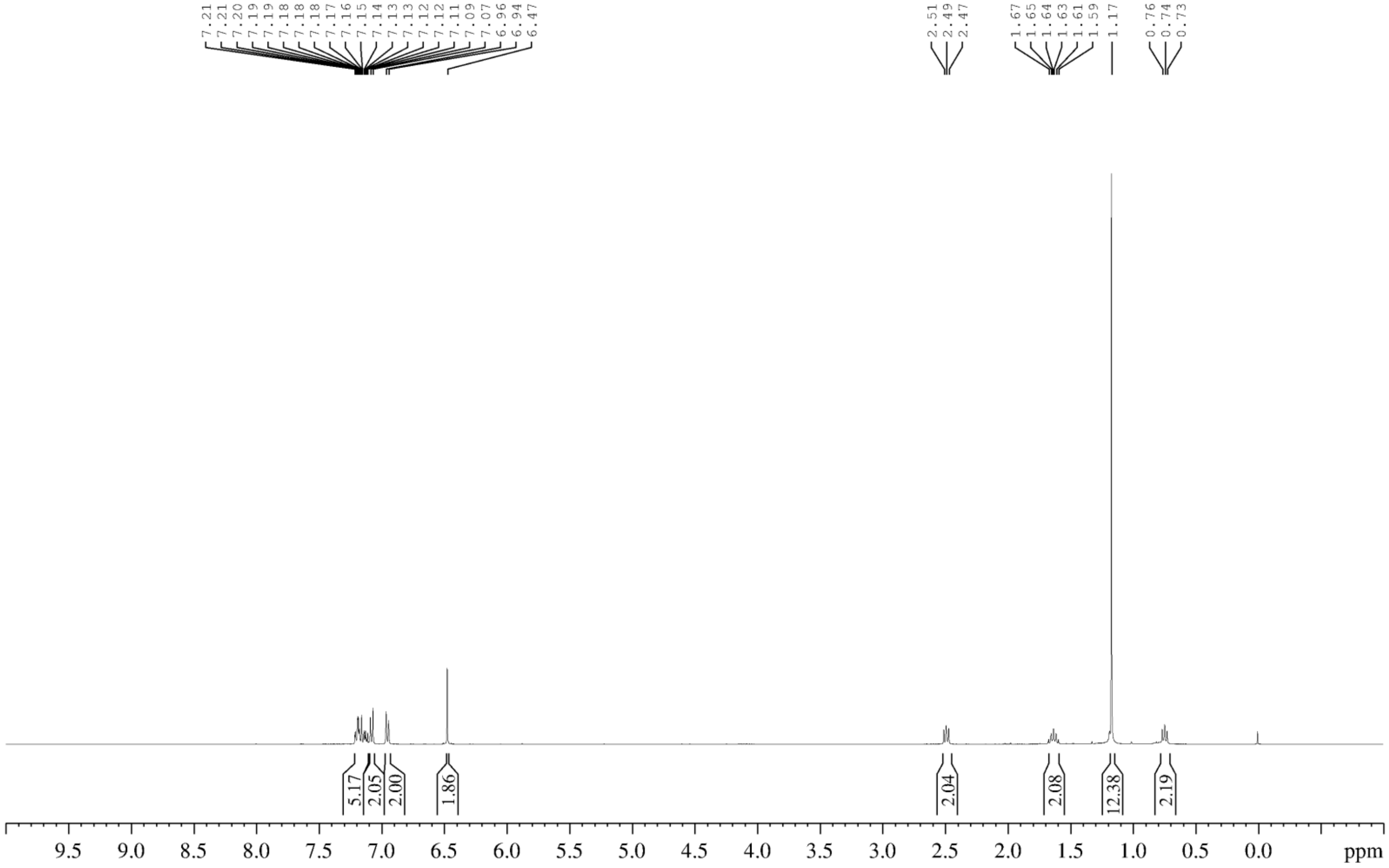

Figure S32. ^{13}C NMR (101 MHz, $CDCl_3$, 298 K) of **2h** (*Z*).

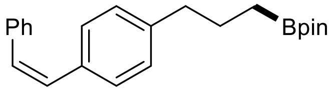

2h (*Z*)

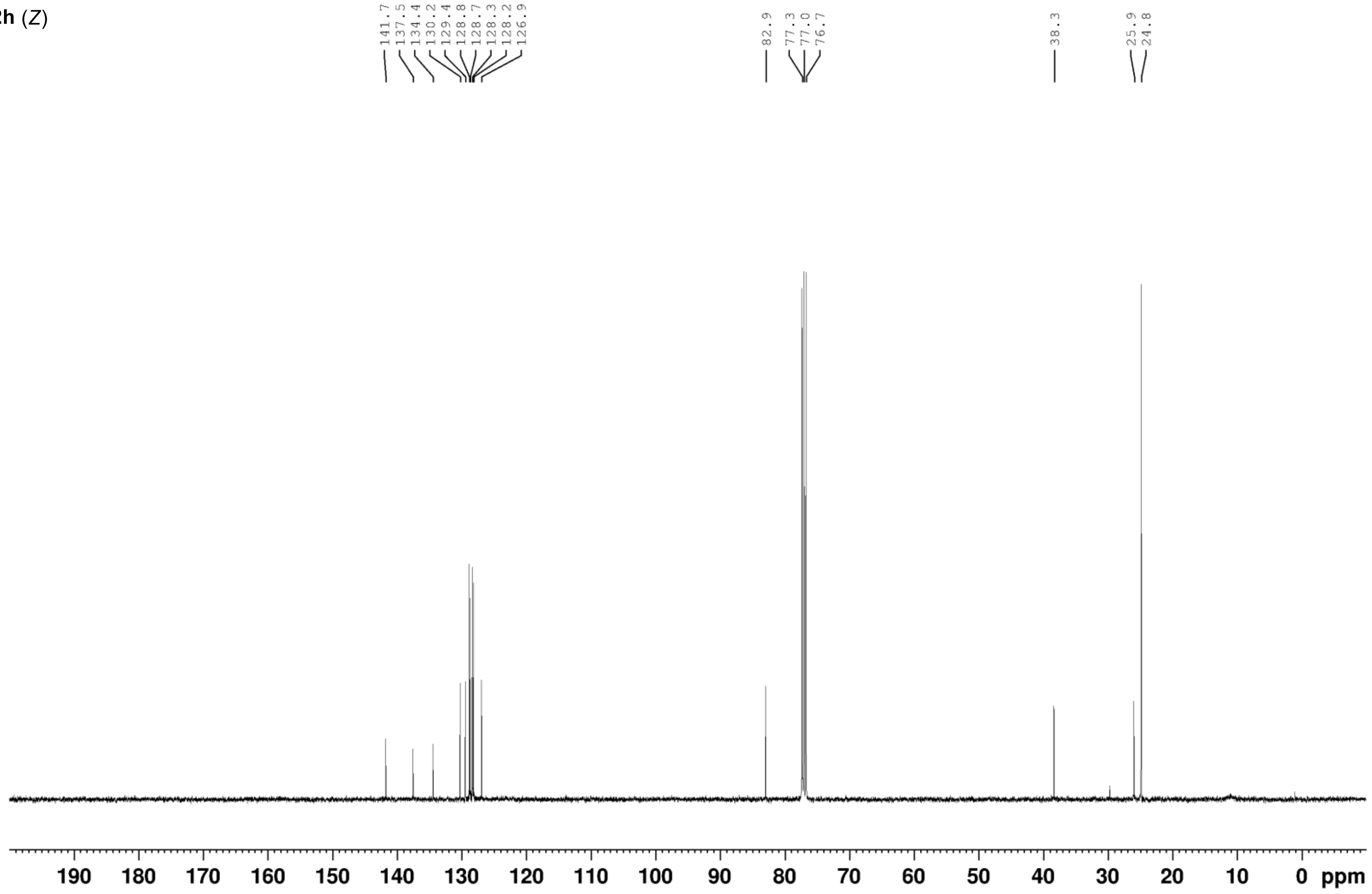

Figure S33. ^{11}B NMR (128 MHz, $CDCl_3$, 298 K) of **2h** (*Z*).

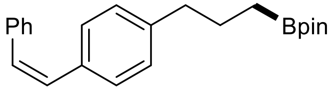

2h (*Z*)

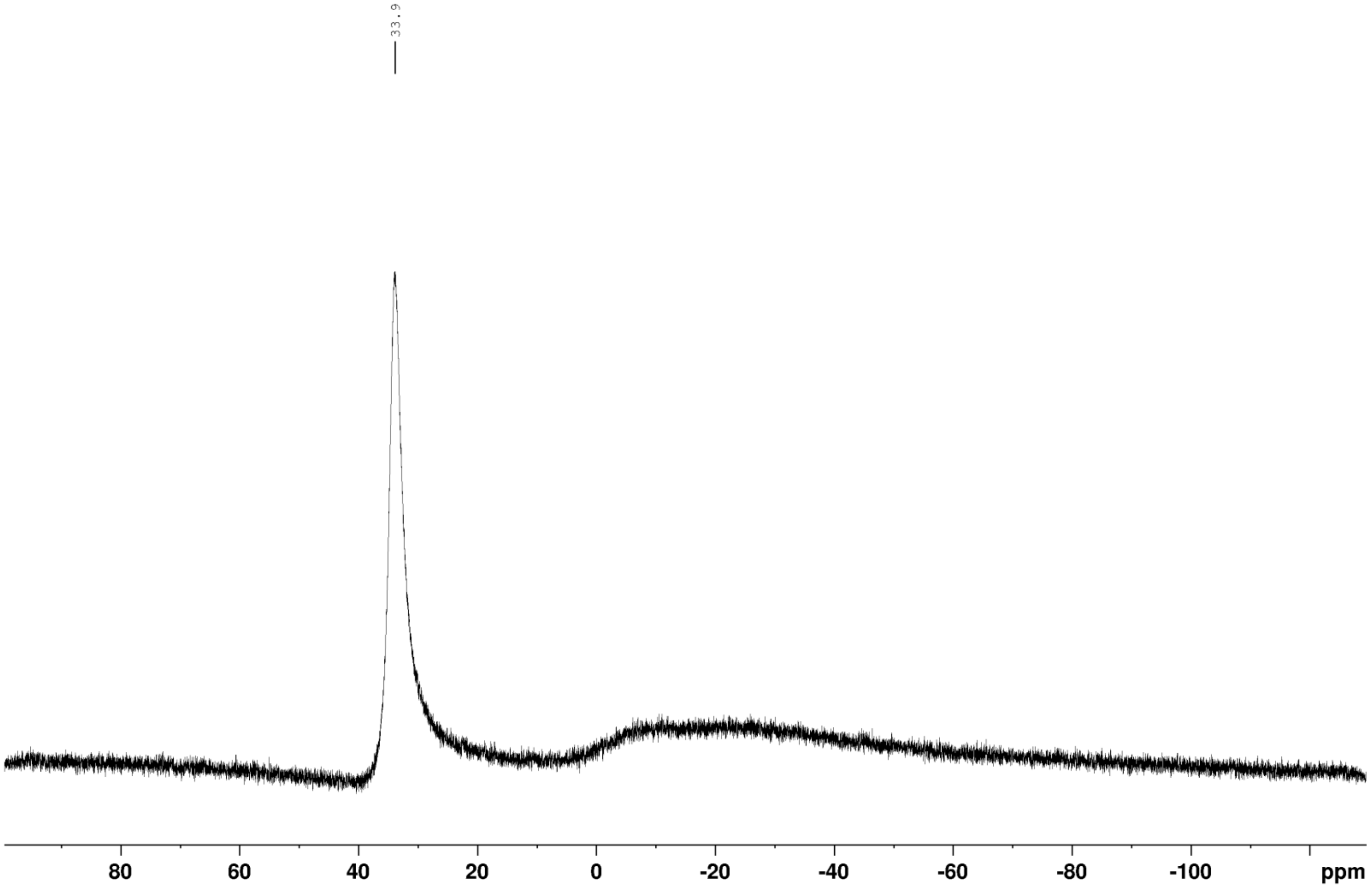

Figure S34. ^{1}H NMR (400 MHz, $CDCl_3$, 298 K) of **2h** (*E*).

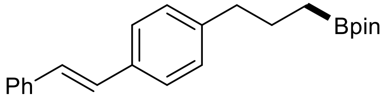

2h (*E*)

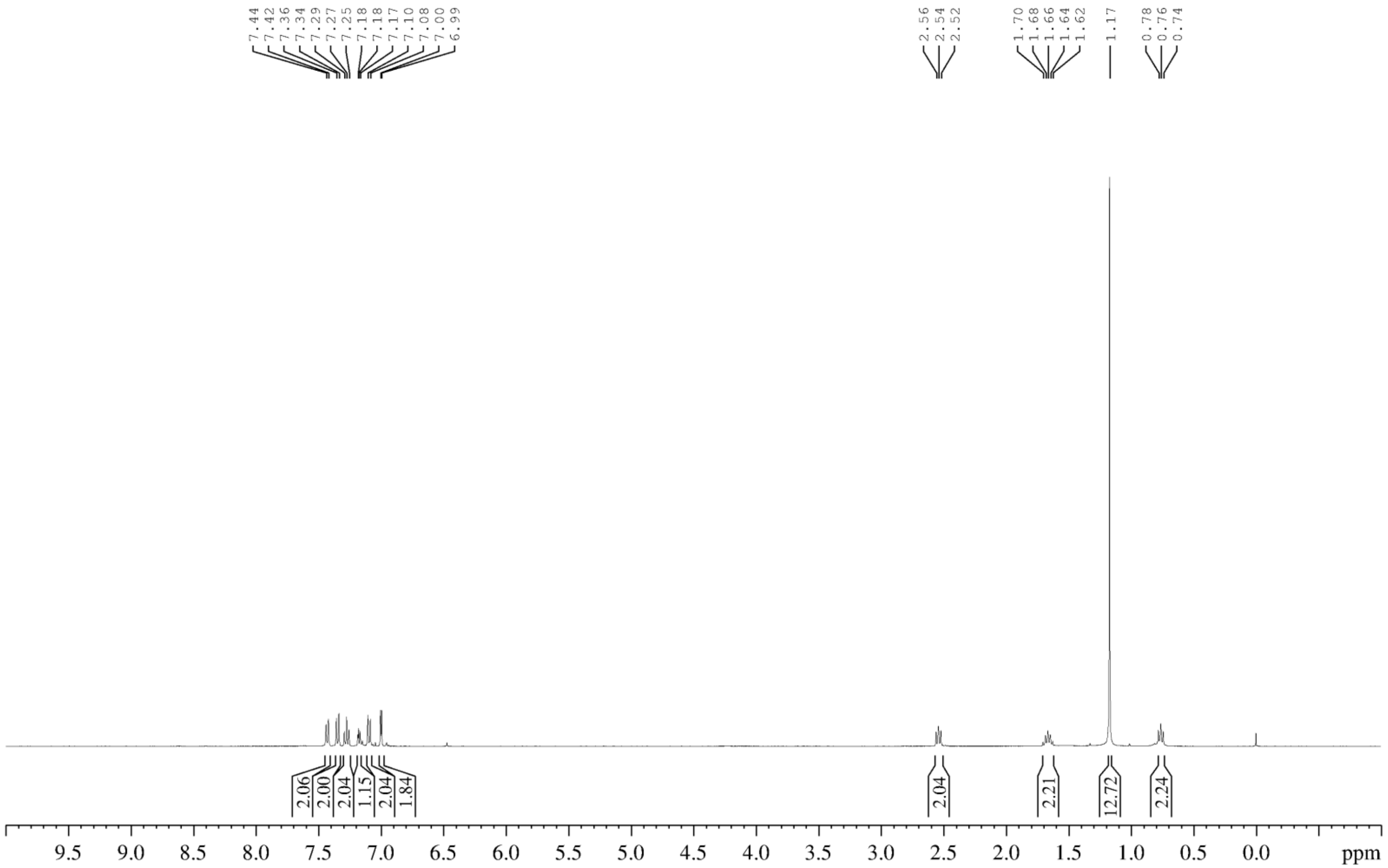

Figure S35. ^{13}C NMR (101 MHz, $CDCl_3$, 298 K) of **2h** (*E*).

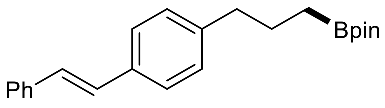

2h (*E*)

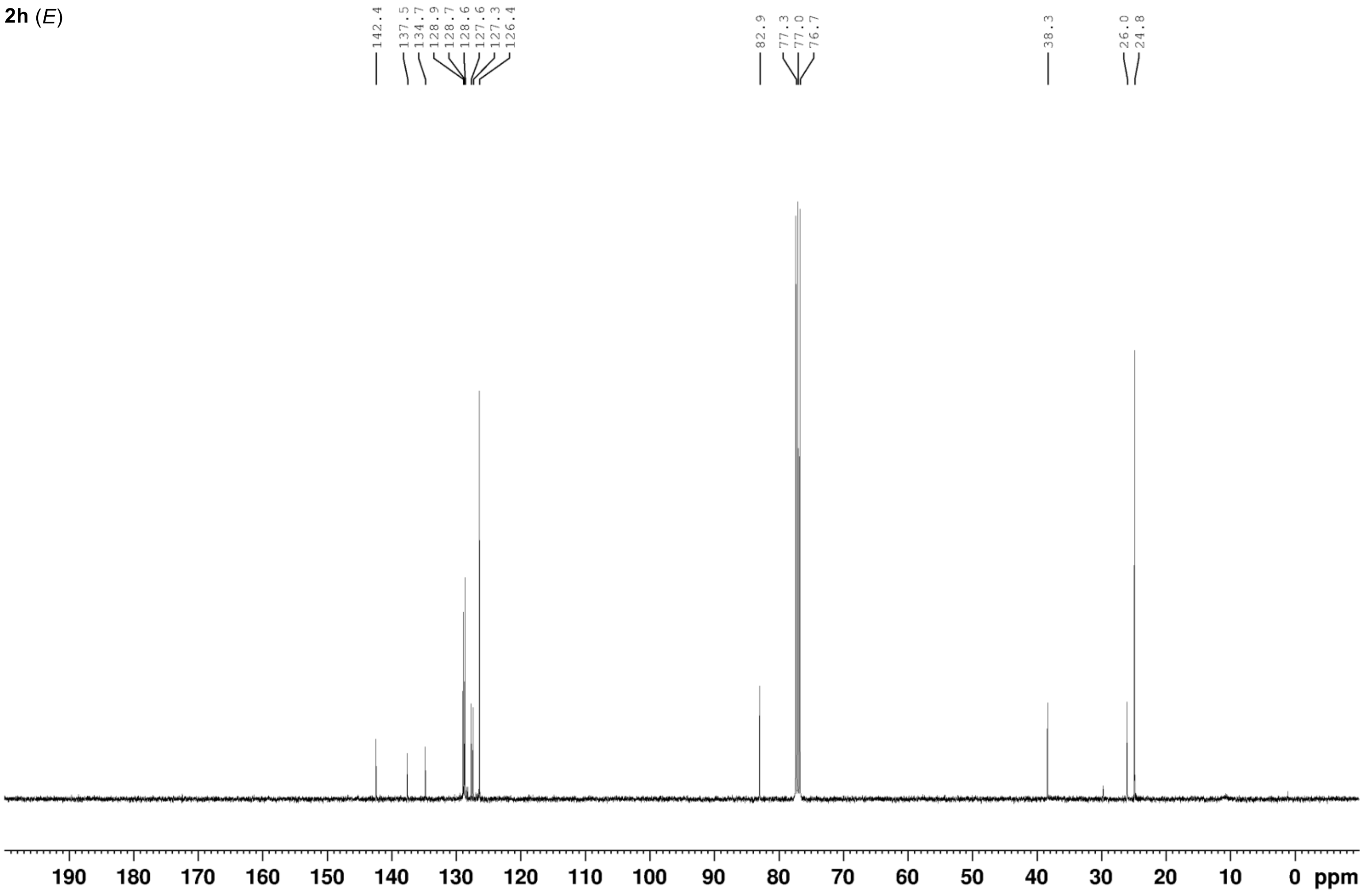

Figure S36. ^{11}B NMR (128 MHz, $CDCl_3$, 298 K) of **2h** (*E*).

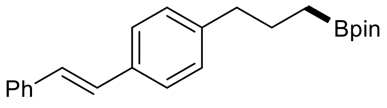

2h (*E*)

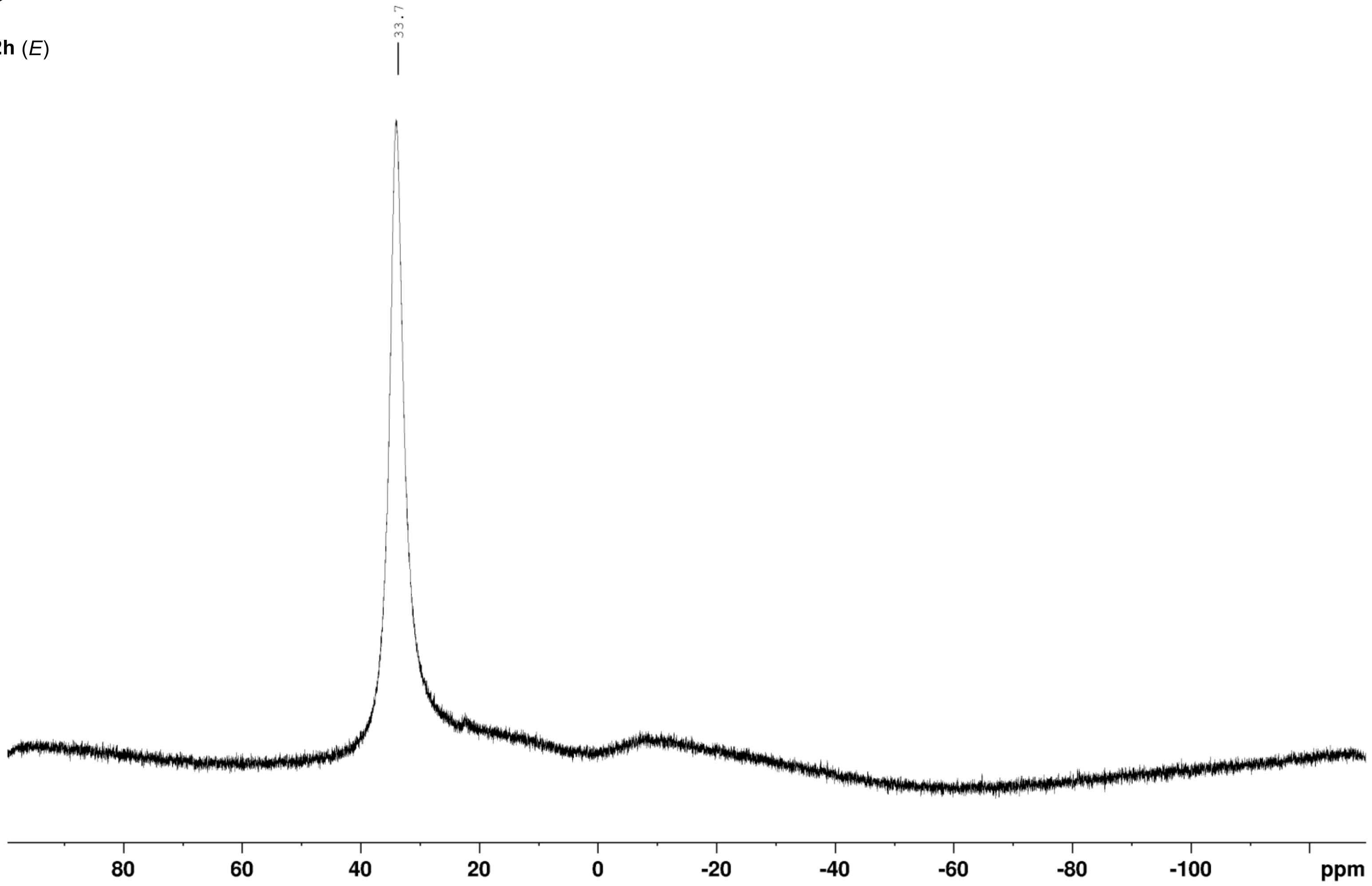

Figure S37. ^{1}H NMR (400 MHz, $CDCl_3$, 298 K) of **2i**.

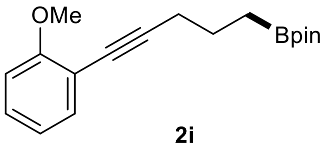

2i

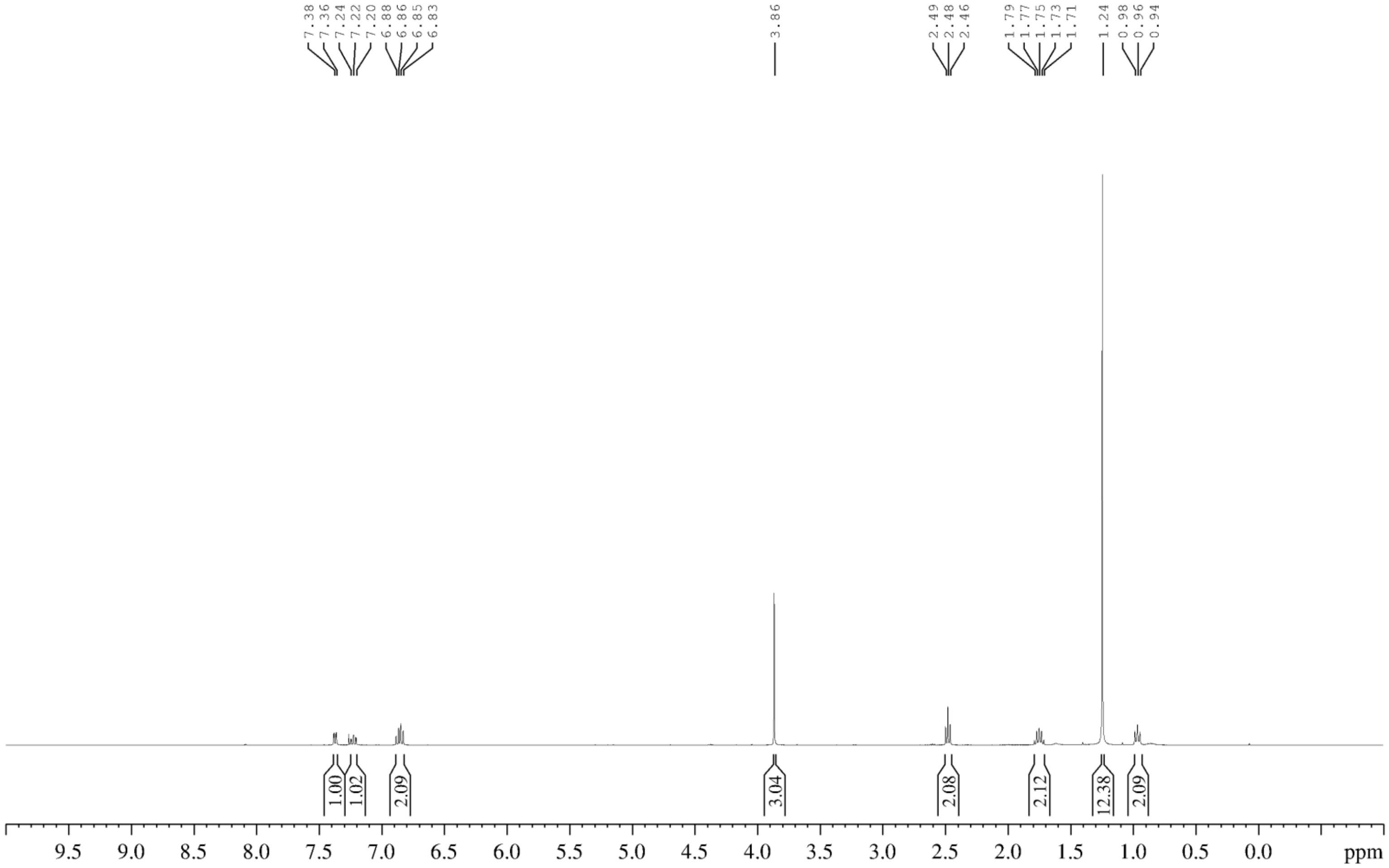

Figure S38. ^{13}C NMR (101 MHz, $CDCl_3$, 298 K) of **2i**.

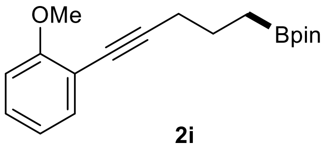

2i

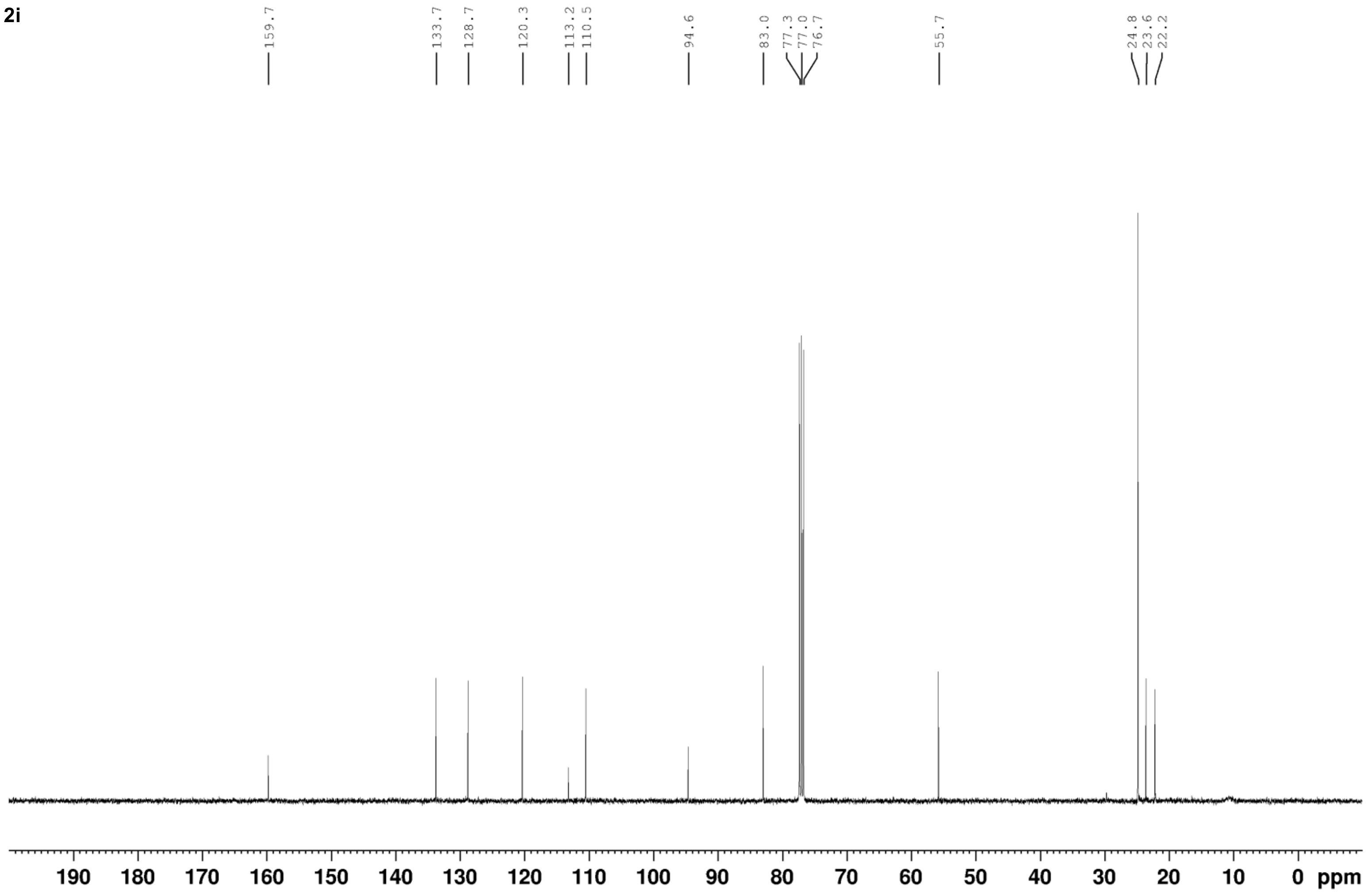

Figure S39. ^{11}B NMR (128 MHz, $CDCl_3$, 298 K) of **2i**.

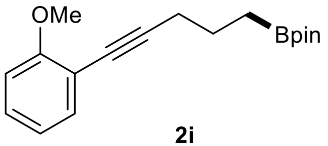

2i

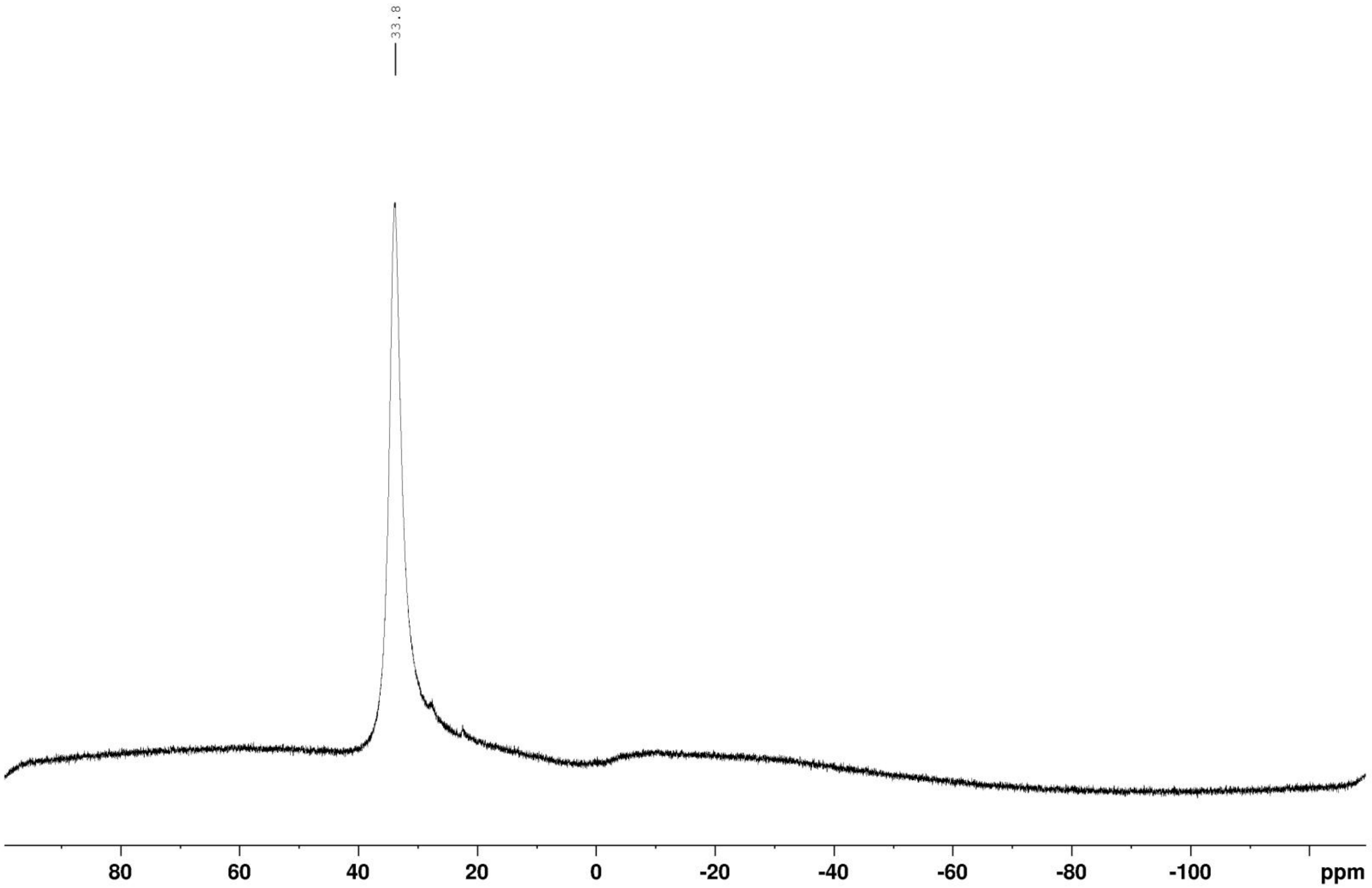

Figure S40. ^{1}H NMR (400 MHz, $CDCl_3$, 298 K) of **2j**.

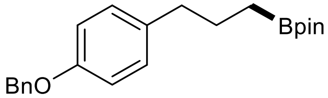

2j

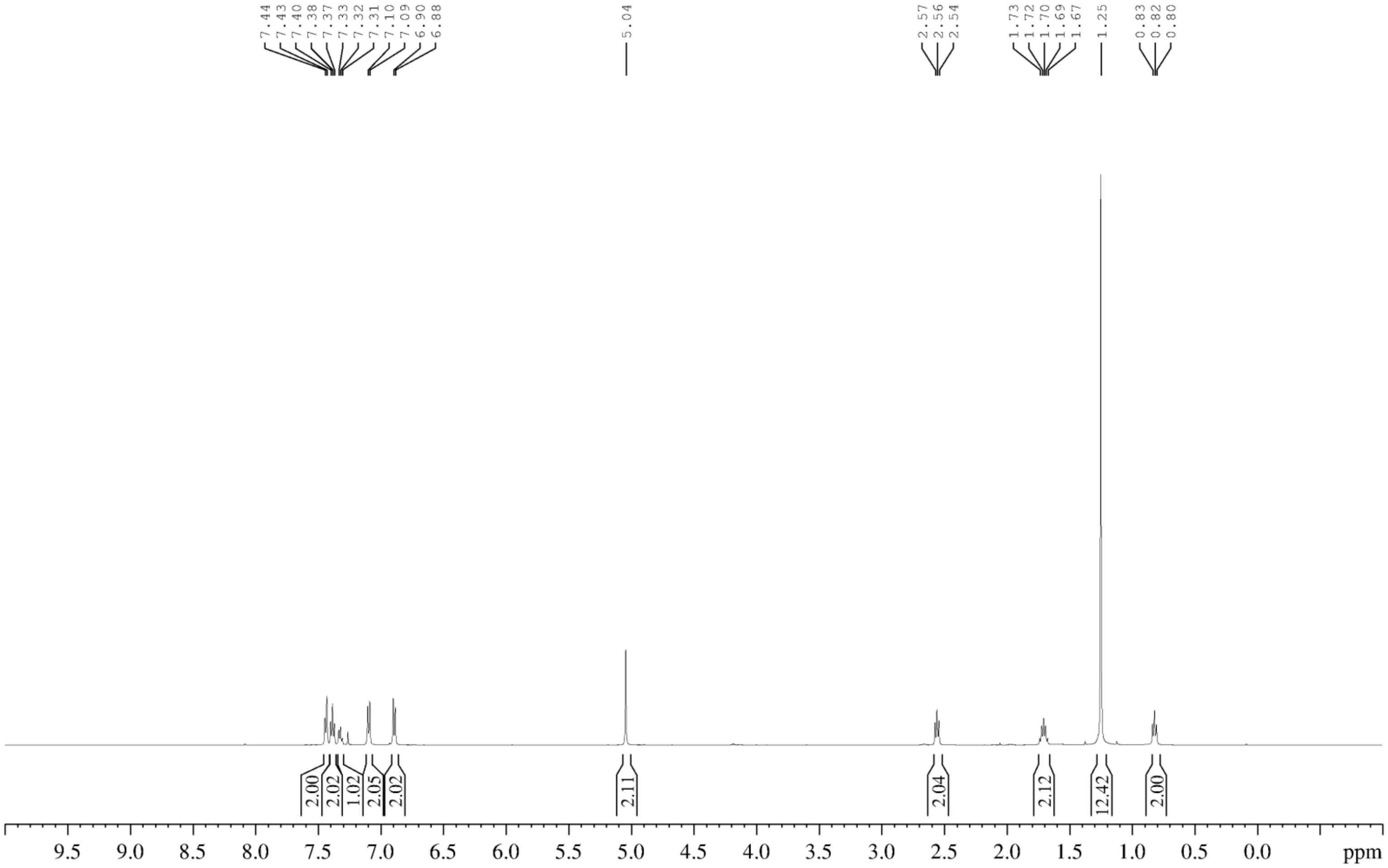

Figure S41. ^{13}C NMR (101 MHz, $CDCl_3$, 298 K) of **2j**.

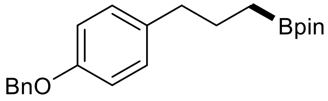

2j

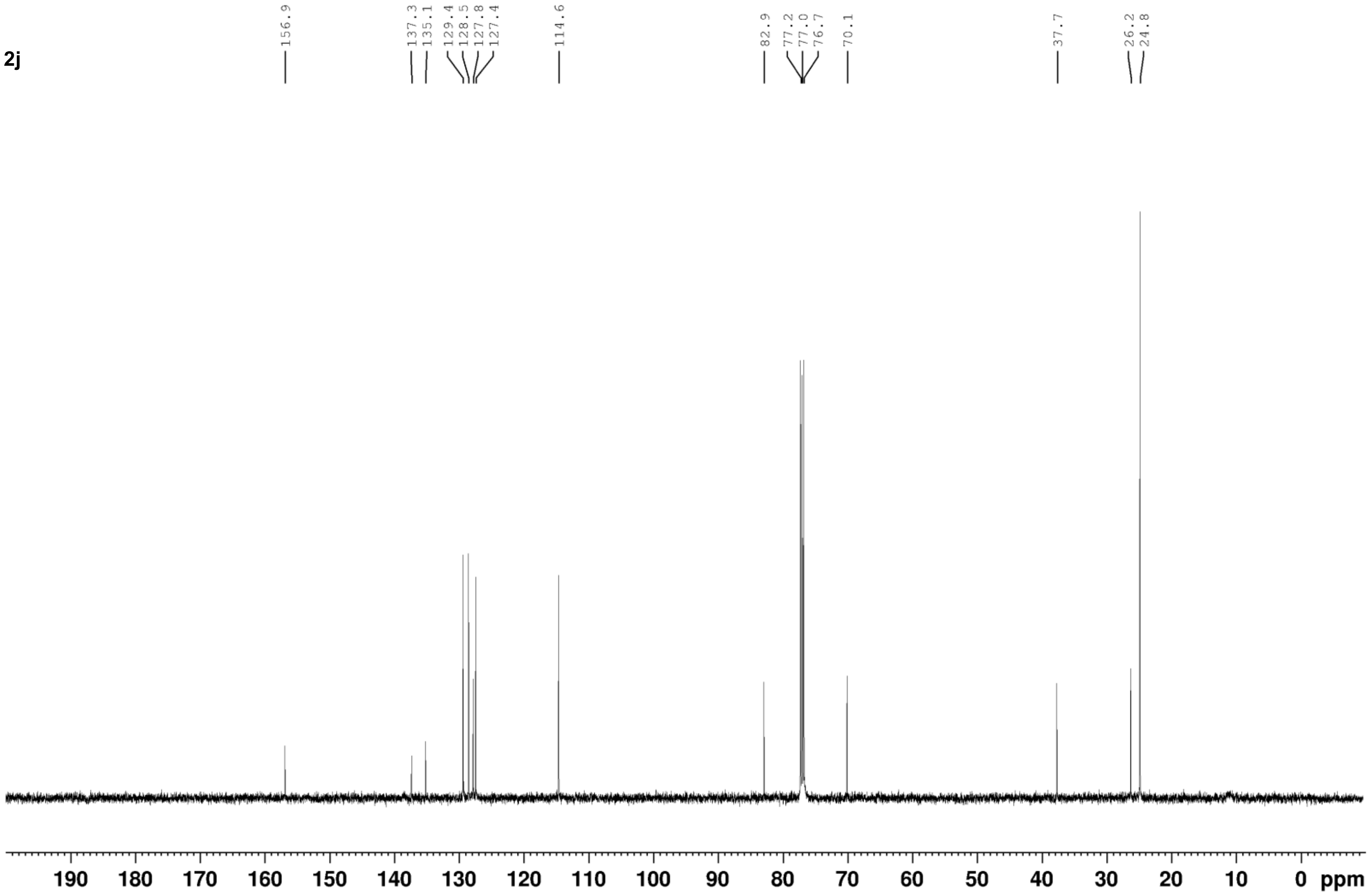

Figure S42. ^{11}B NMR (128 MHz, $CDCl_3$, 298 K) of **2j**.

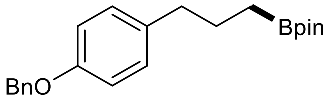

2j

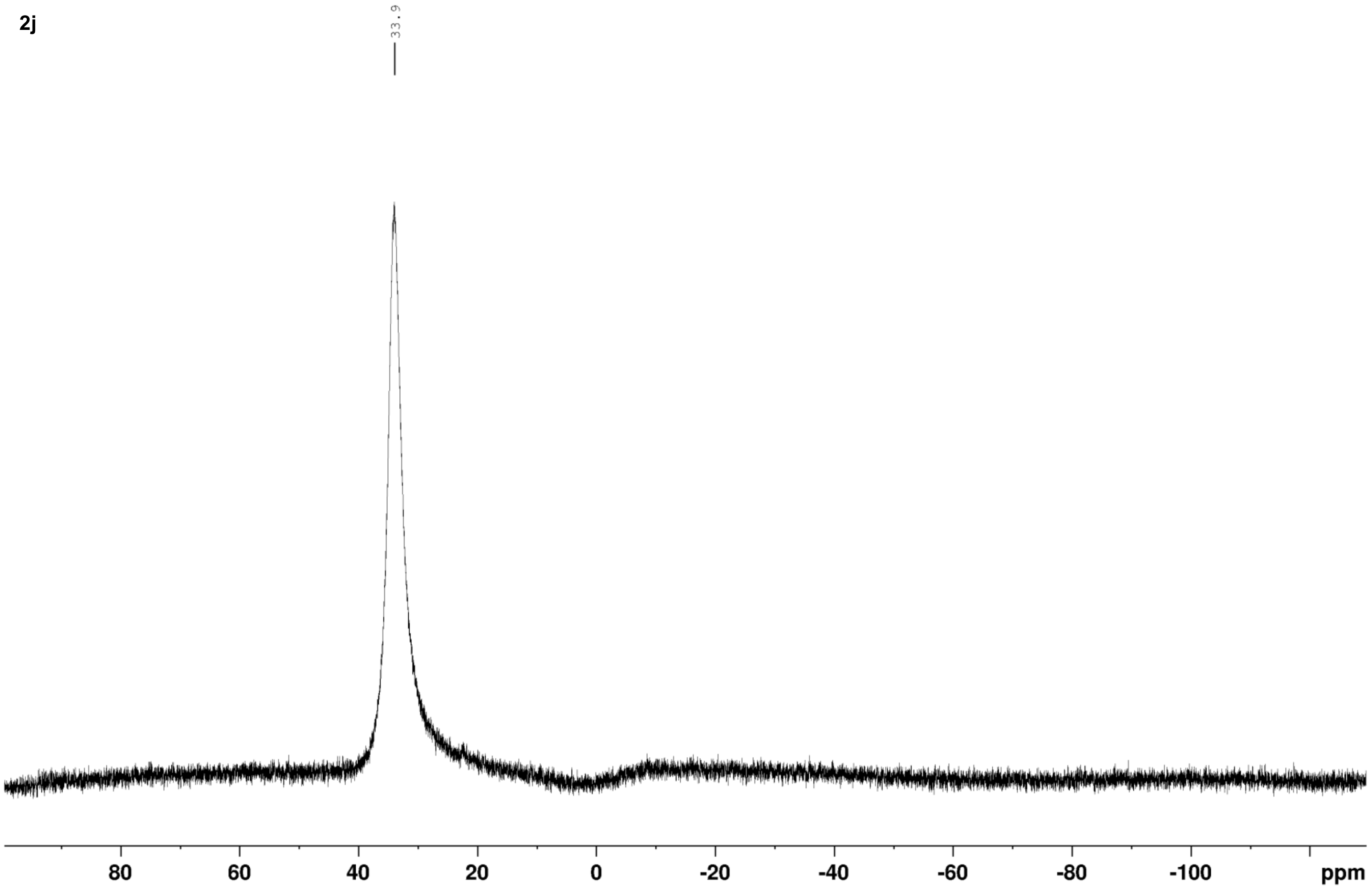

Figure S43. ^{1}H NMR (400 MHz, $CDCl_3$, 298 K) of **2k**.

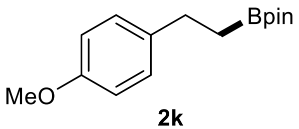

2k

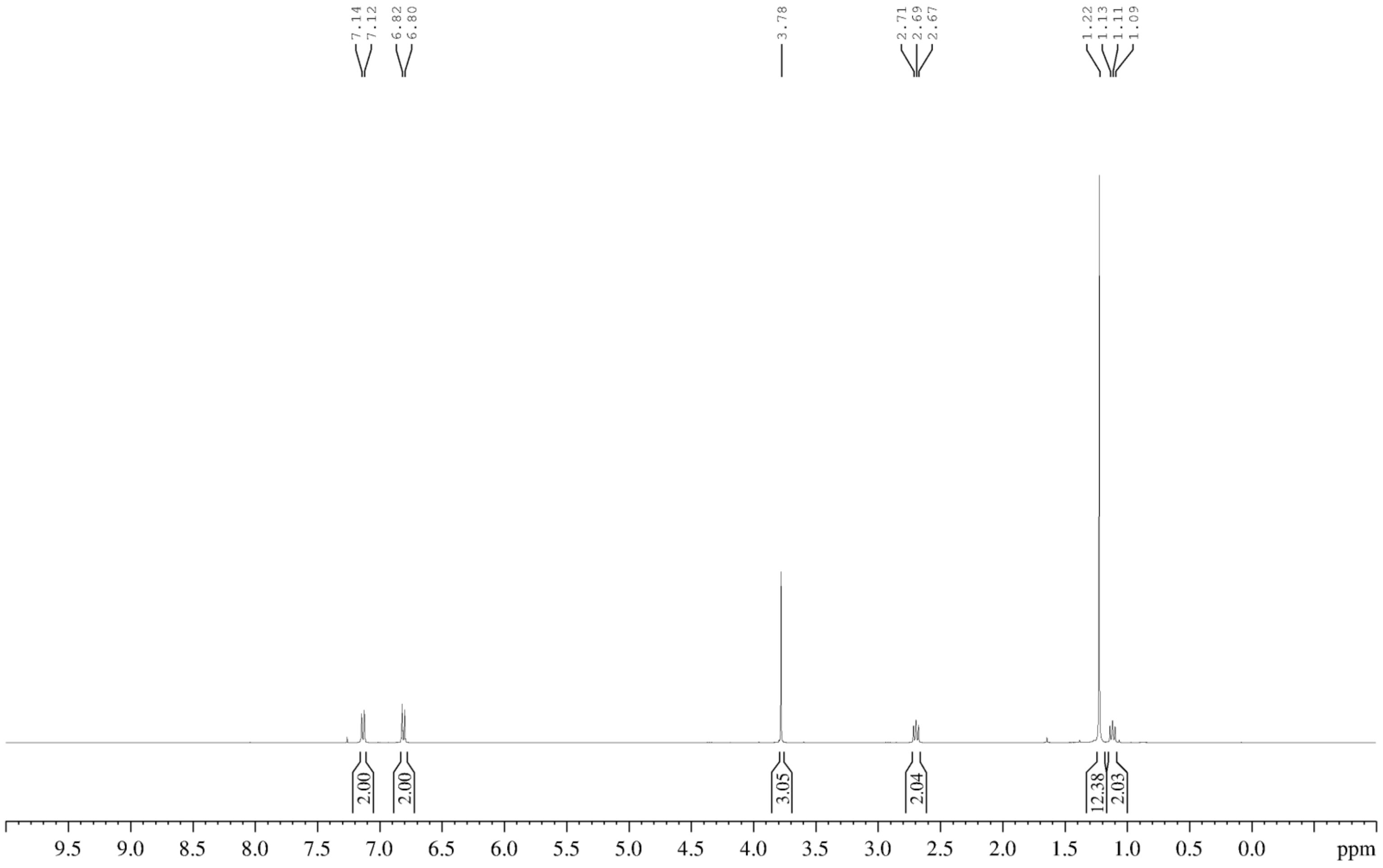

Figure S44. ^{13}C NMR (101 MHz, $CDCl_3$, 298 K) of **2k**.

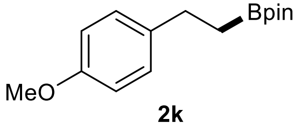

2k

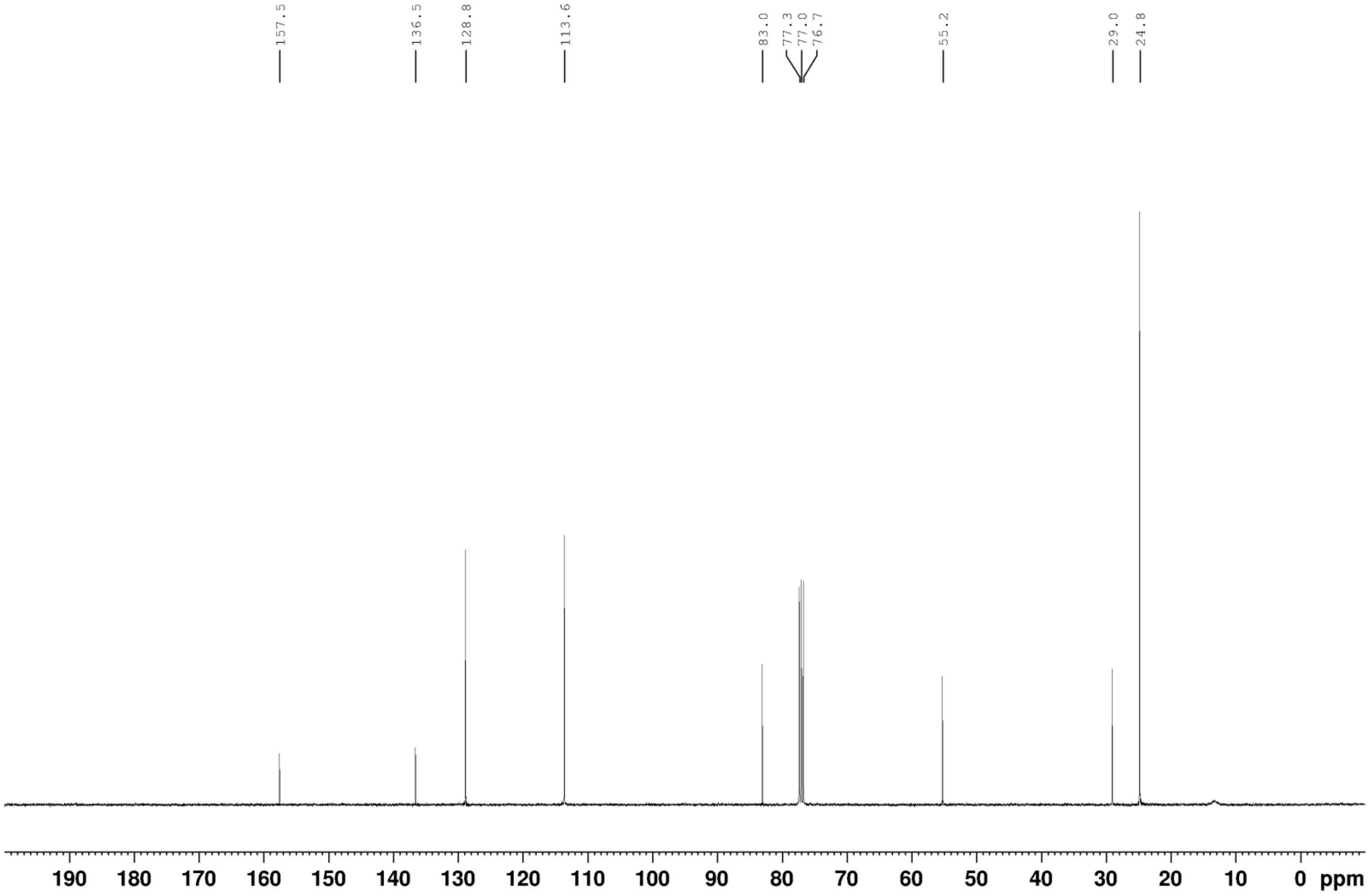

Figure S45. ^{11}B NMR (128 MHz, $CDCl_3$, 298 K) of **2k**.

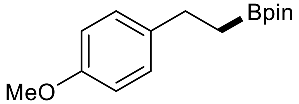

2k

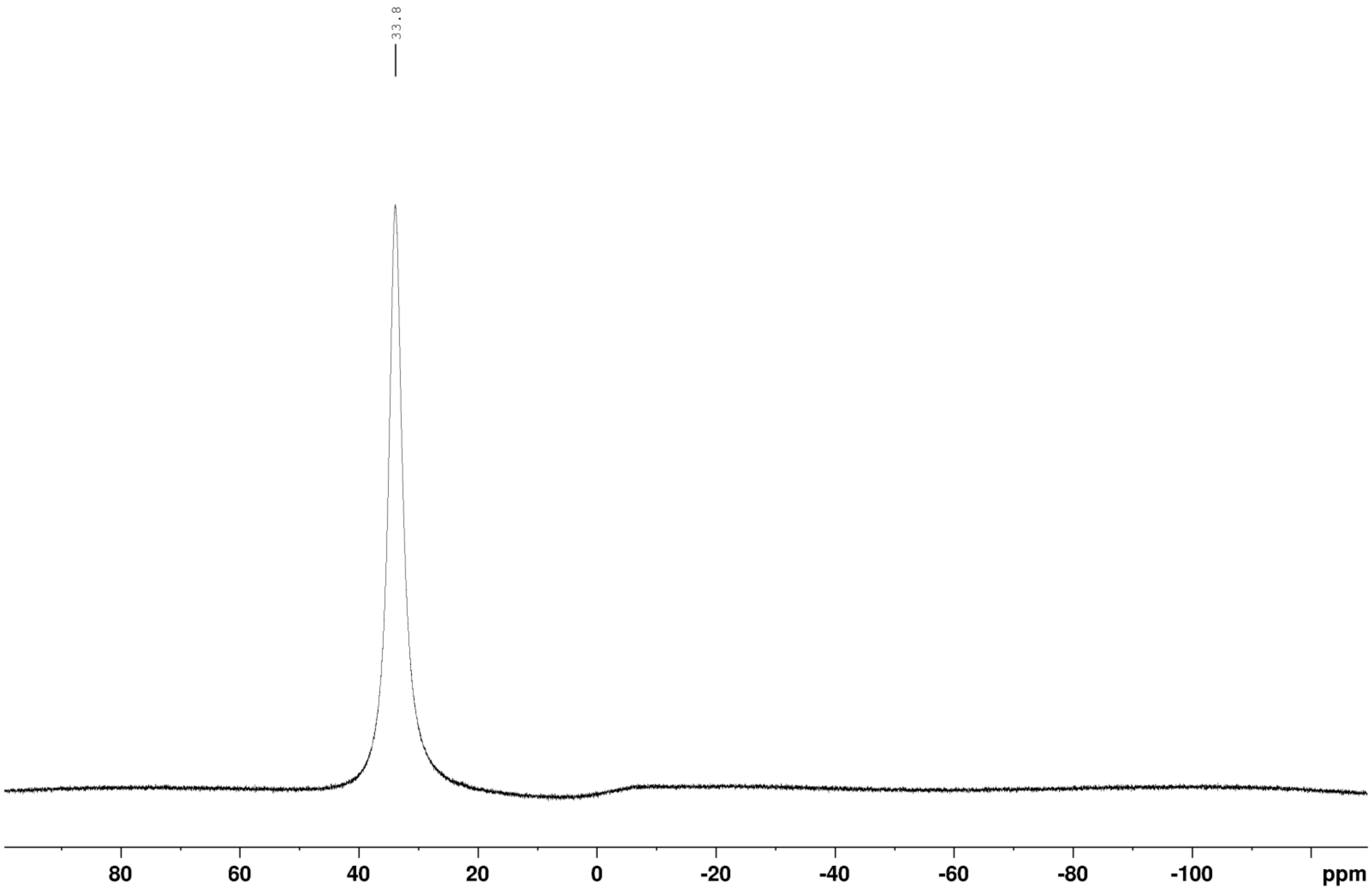

Figure S46. ^{1}H NMR (400 MHz, $CDCl_3$, 298 K) of **2l**.

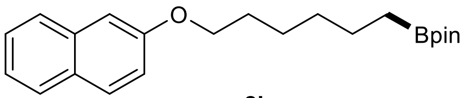

2l

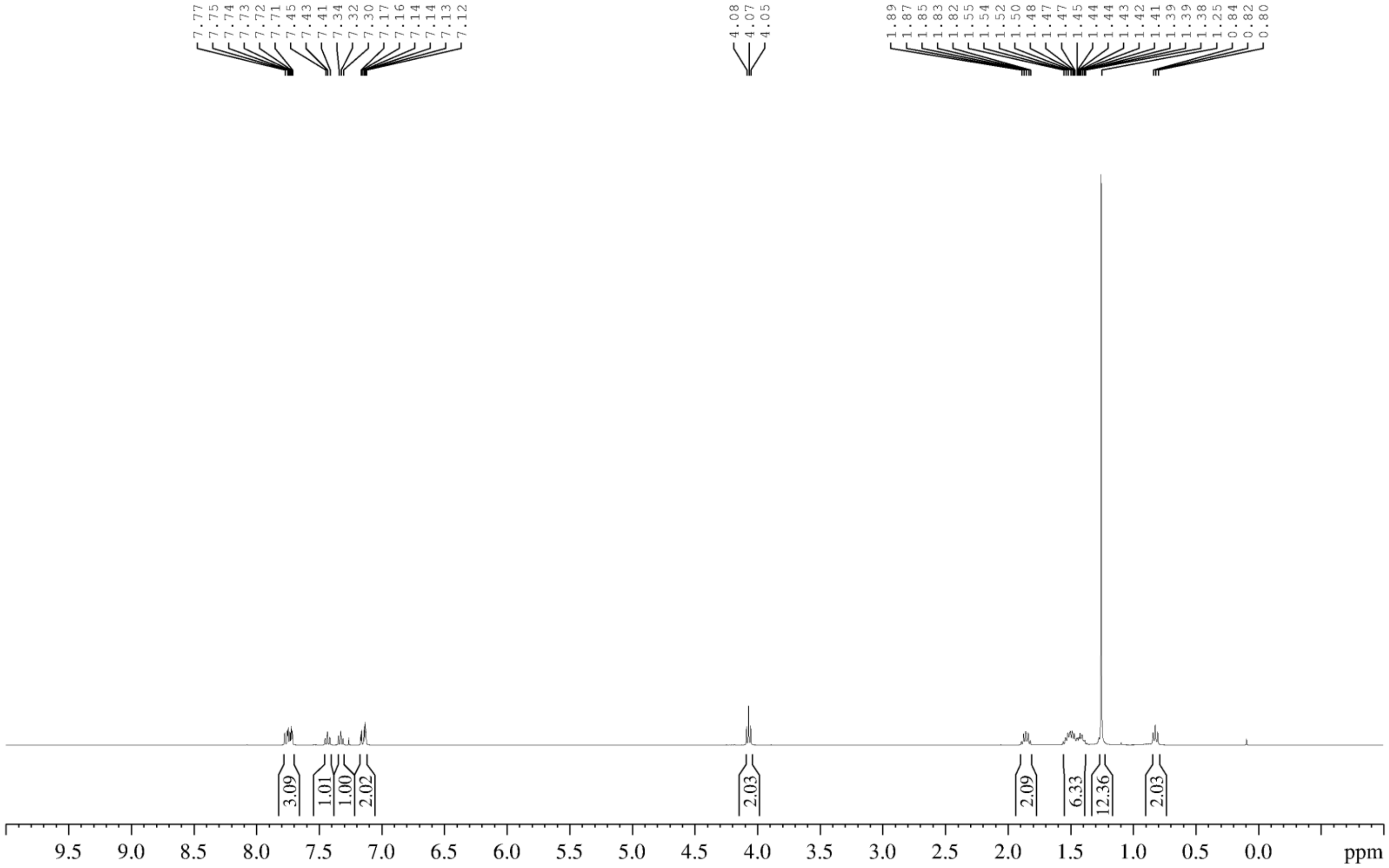

Figure S47. ^{13}C NMR (101 MHz, $CDCl_3$, 298 K) of **2l**.

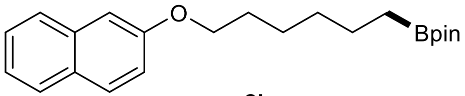

2l

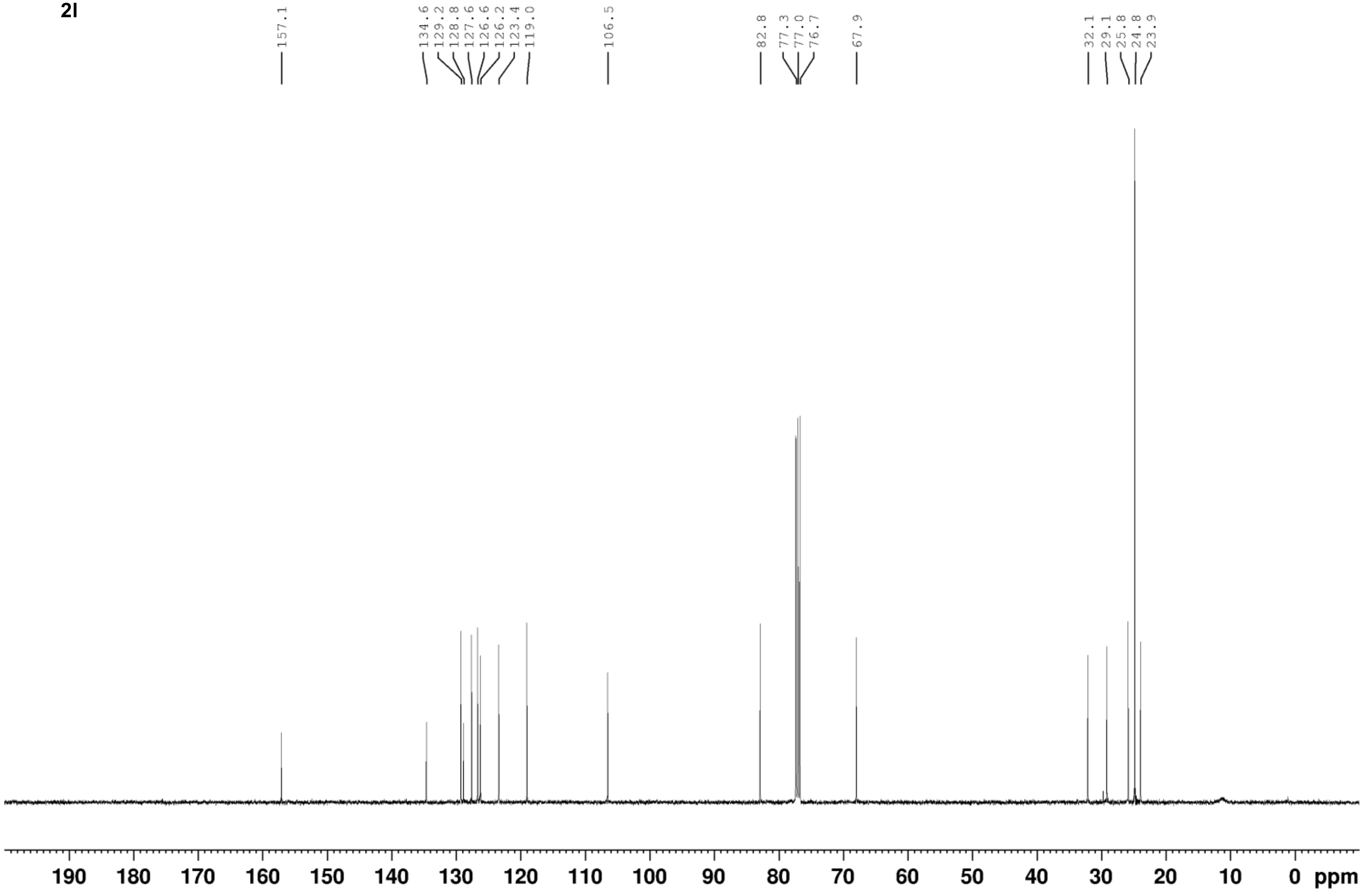

Figure S48. ^{11}B NMR (128 MHz, $CDCl_3$, 298 K) of **2l**.

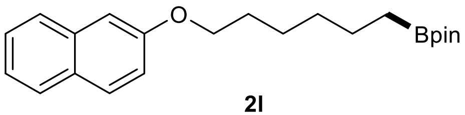

2l

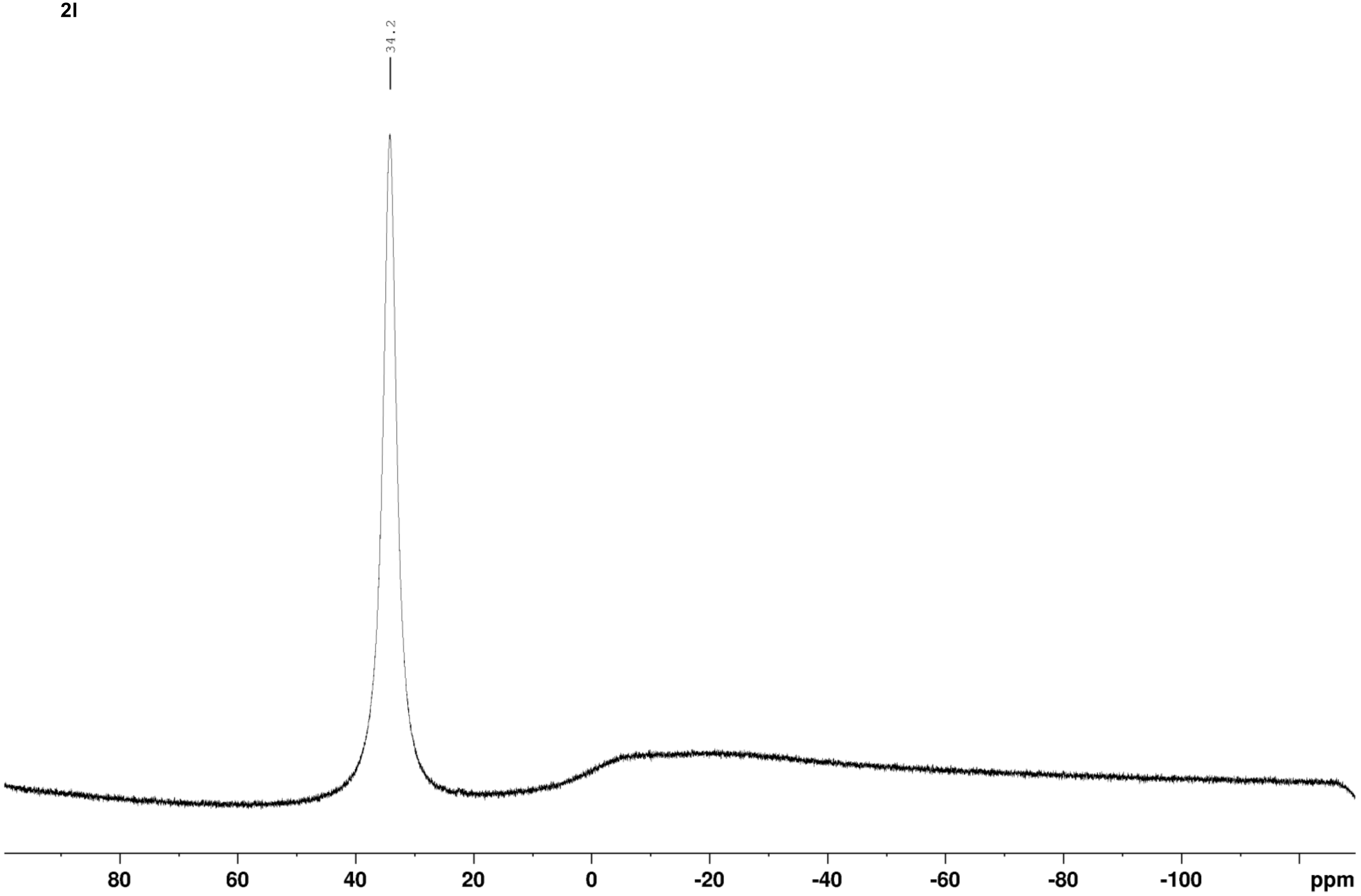

Figure S49. ^{1}H NMR (400MHz, $CDCl_3$, 298 K) of **2m**.

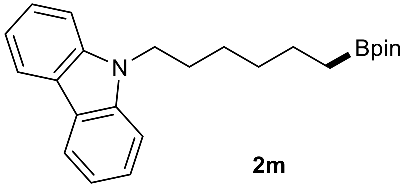

2m

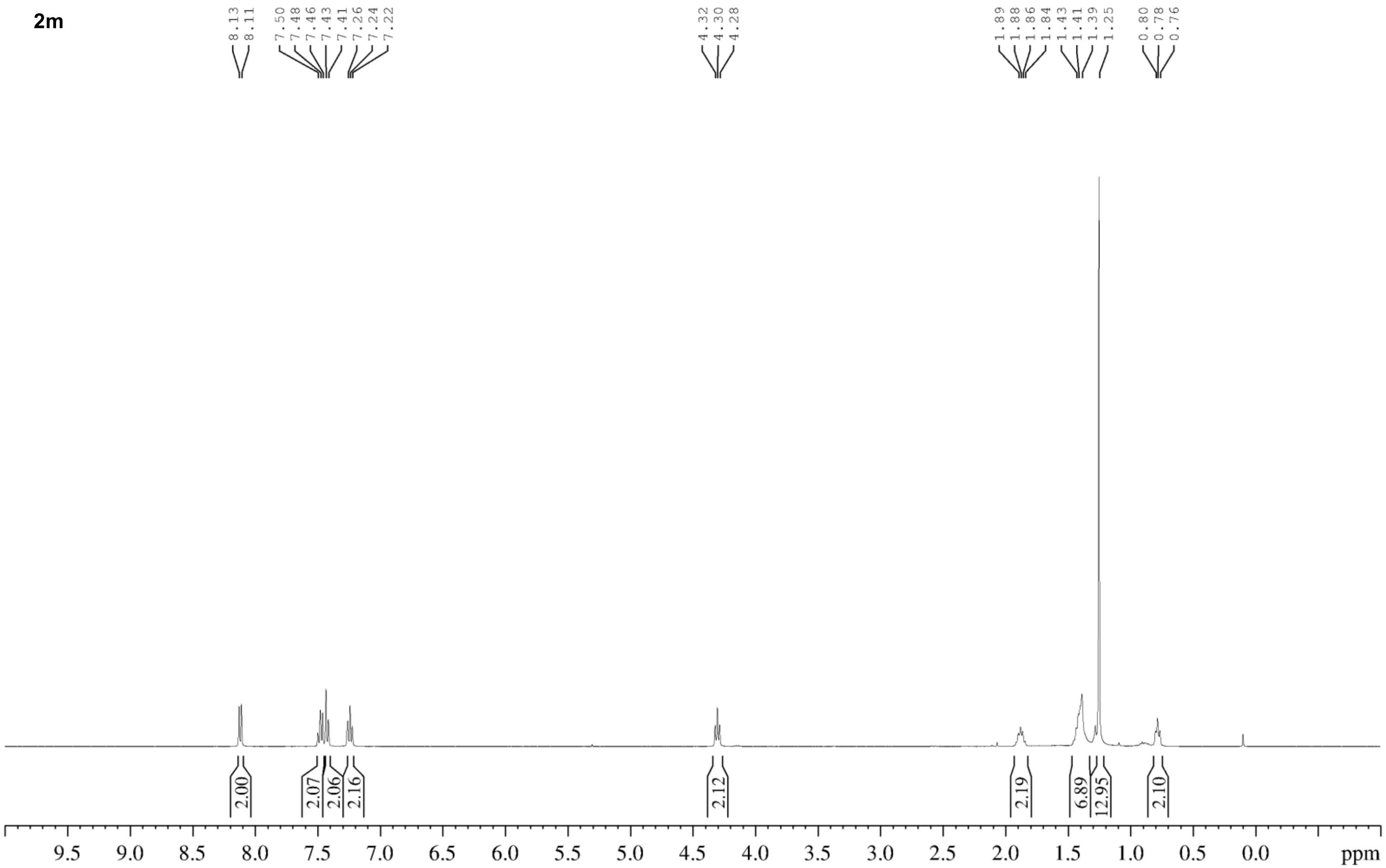

Figure S50. ^{13}C NMR (101 MHz, $CDCl_3$, 298 K) of **2m**.

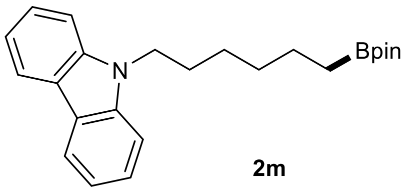

2m

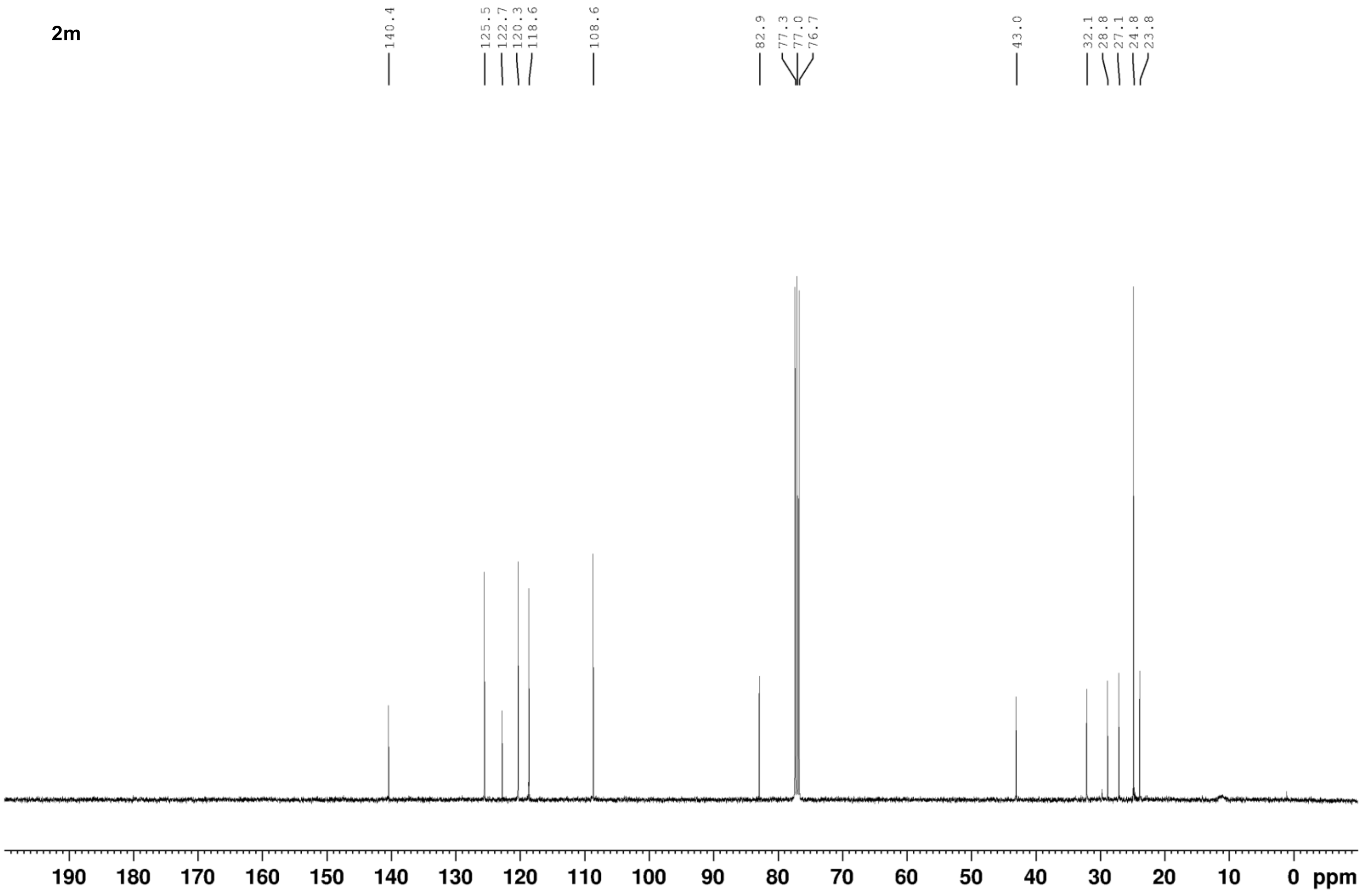

Figure S51. ^{11}B NMR (128 MHz, $CDCl_3$, 298 K) of **2m**.

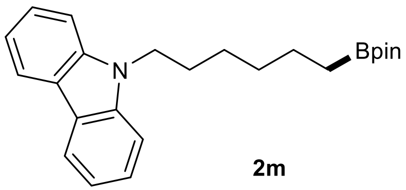

2m

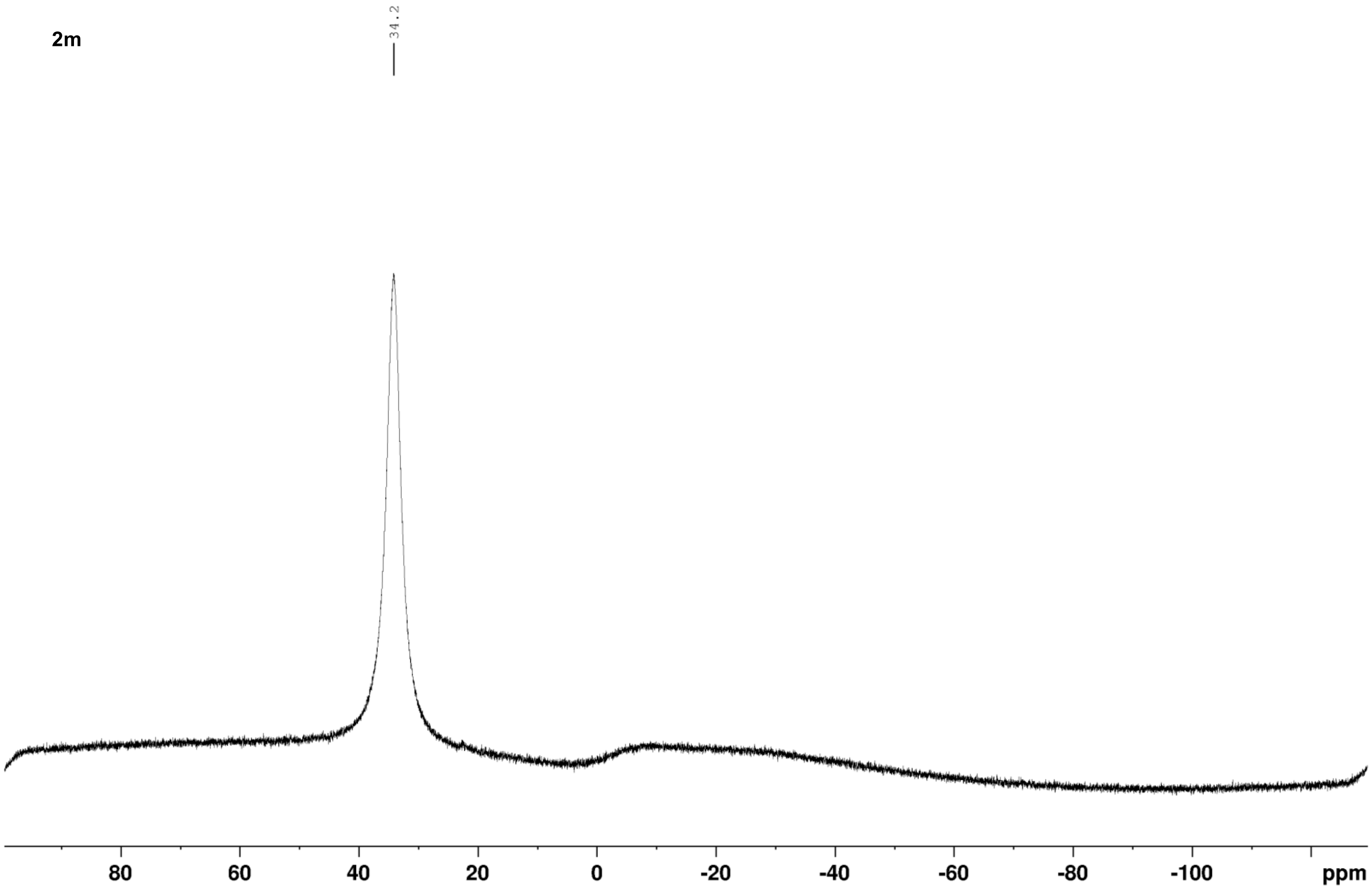

Figure S52. ^{1}H NMR (400 MHz, $CDCl_3$, 298 K) of **2n**.

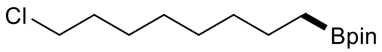

2n

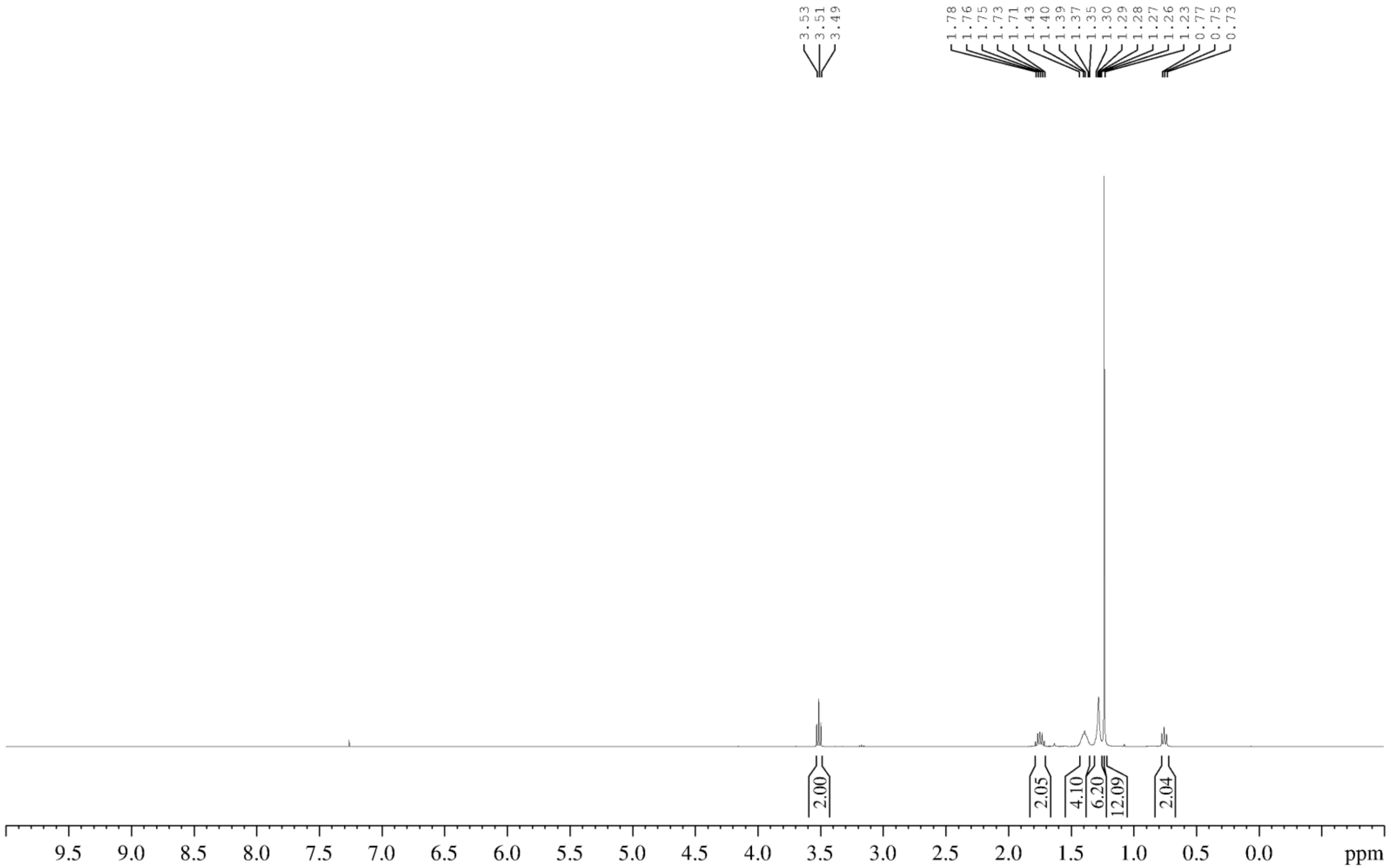

Figure S53. ^{13}C NMR (101 MHz, $CDCl_3$, 298 K) of **2n**.

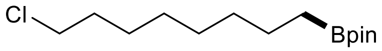

2n

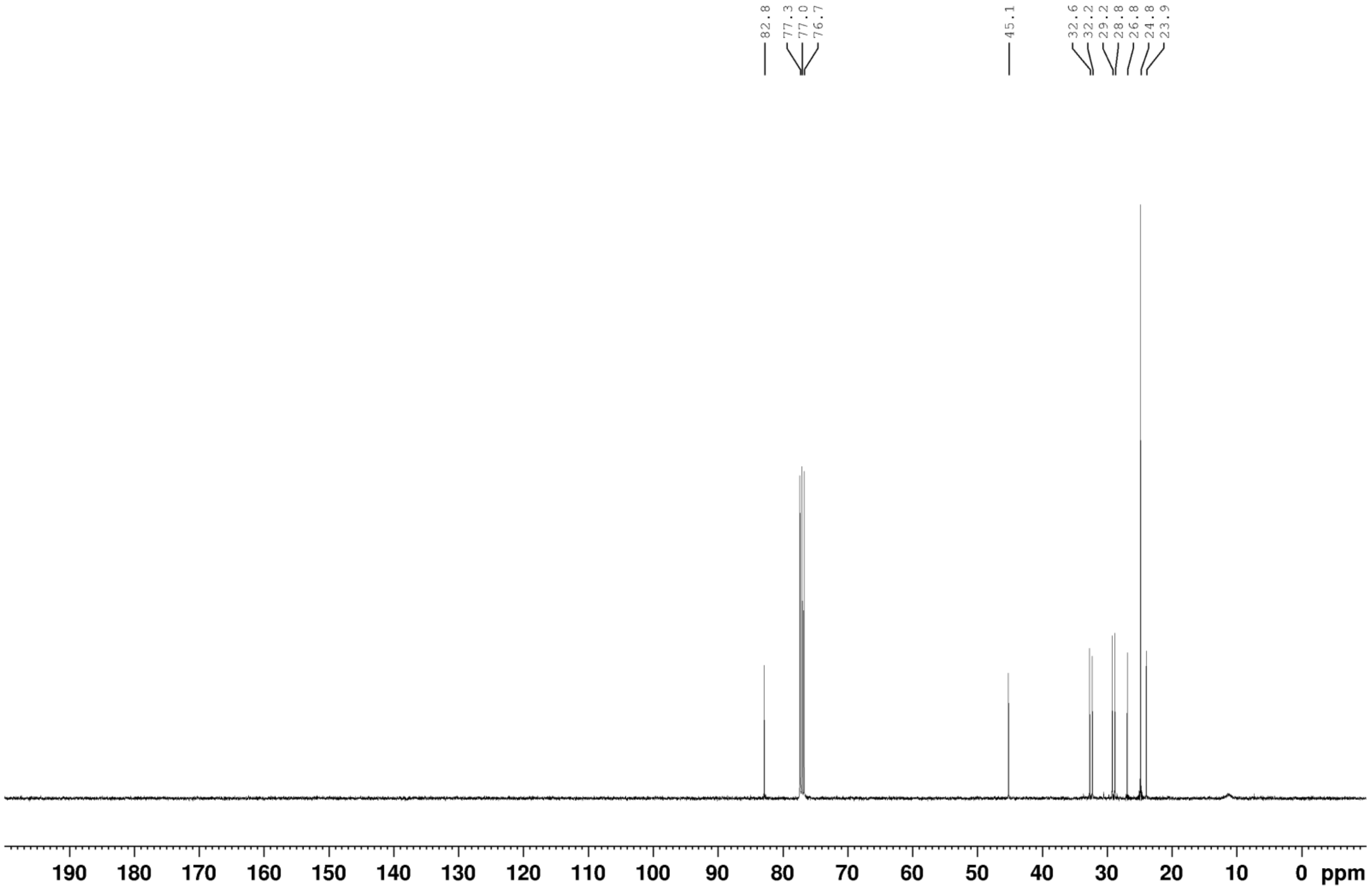

Figure S54. ^{11}B NMR (128 MHz, $CDCl_3$, 298 K) of **2n**.

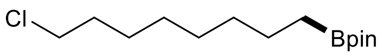

2n

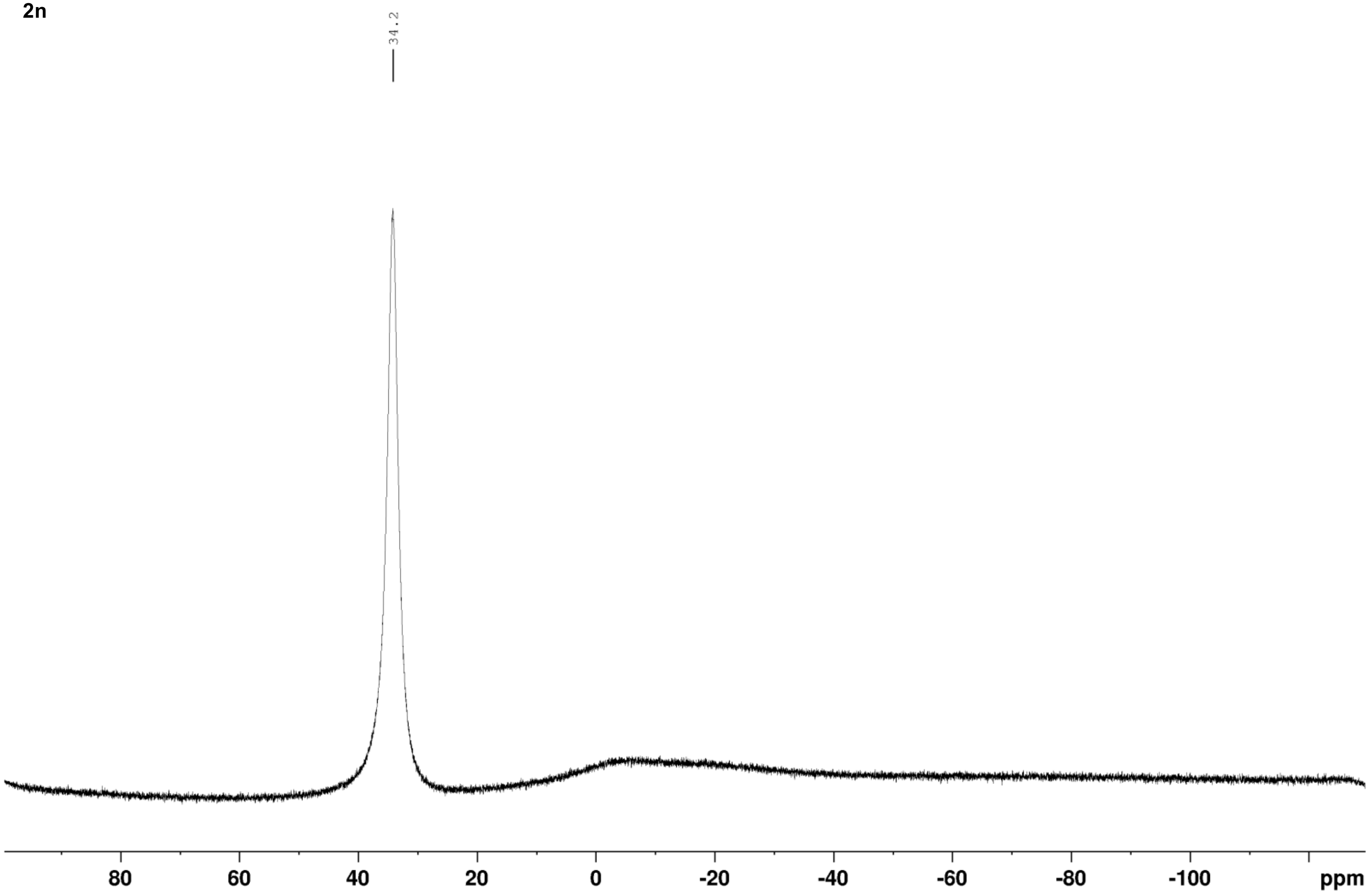

Figure S55. ^1H NMR (400 MHz, $CDCl_3$, 298 K) of **2o**.

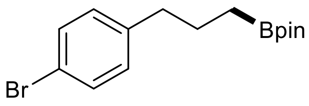

2o

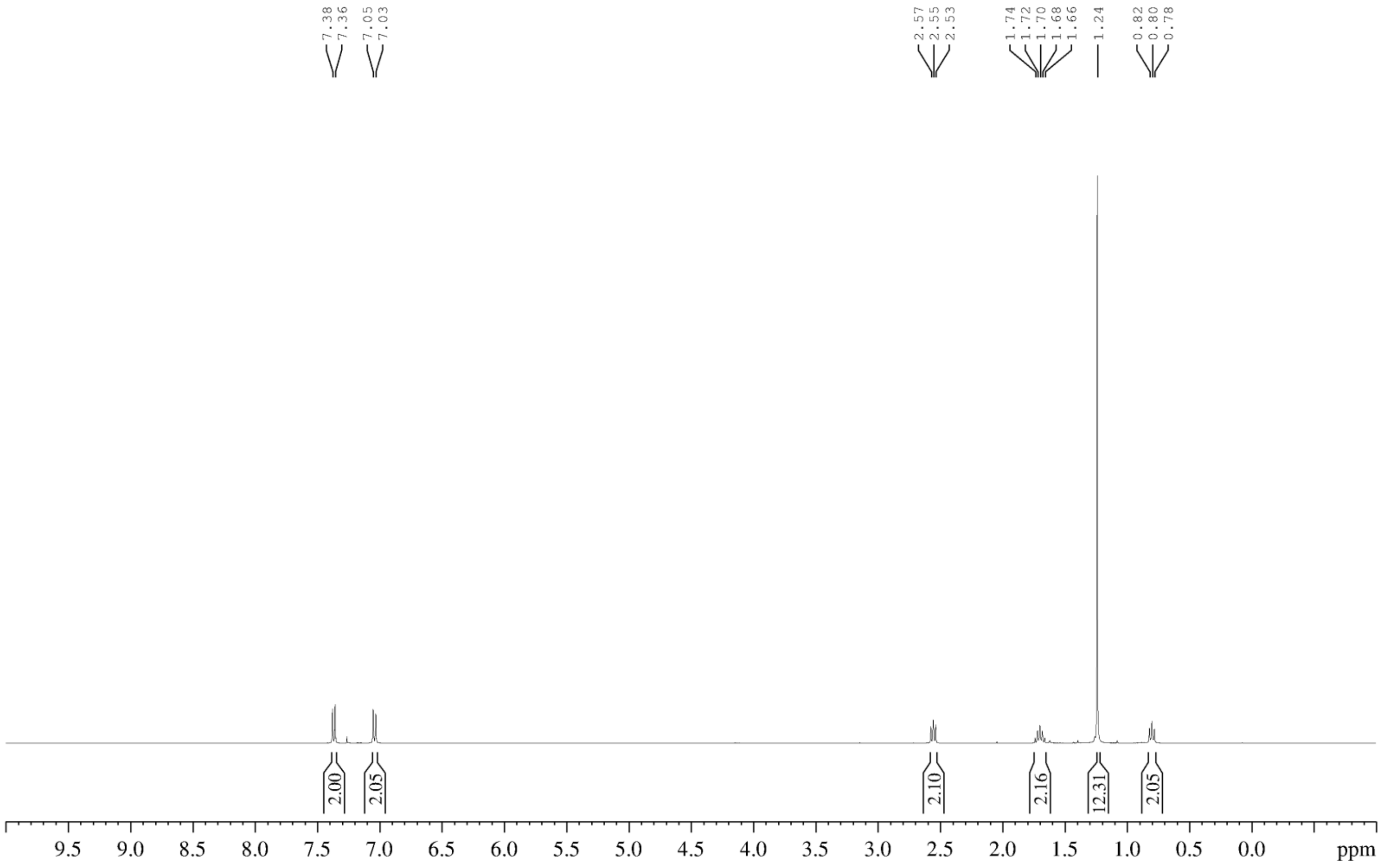

Figure S56. ^{13}C NMR (101 MHz, $CDCl_3$, 298 K) of **2o**.

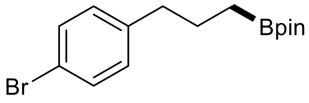

2o

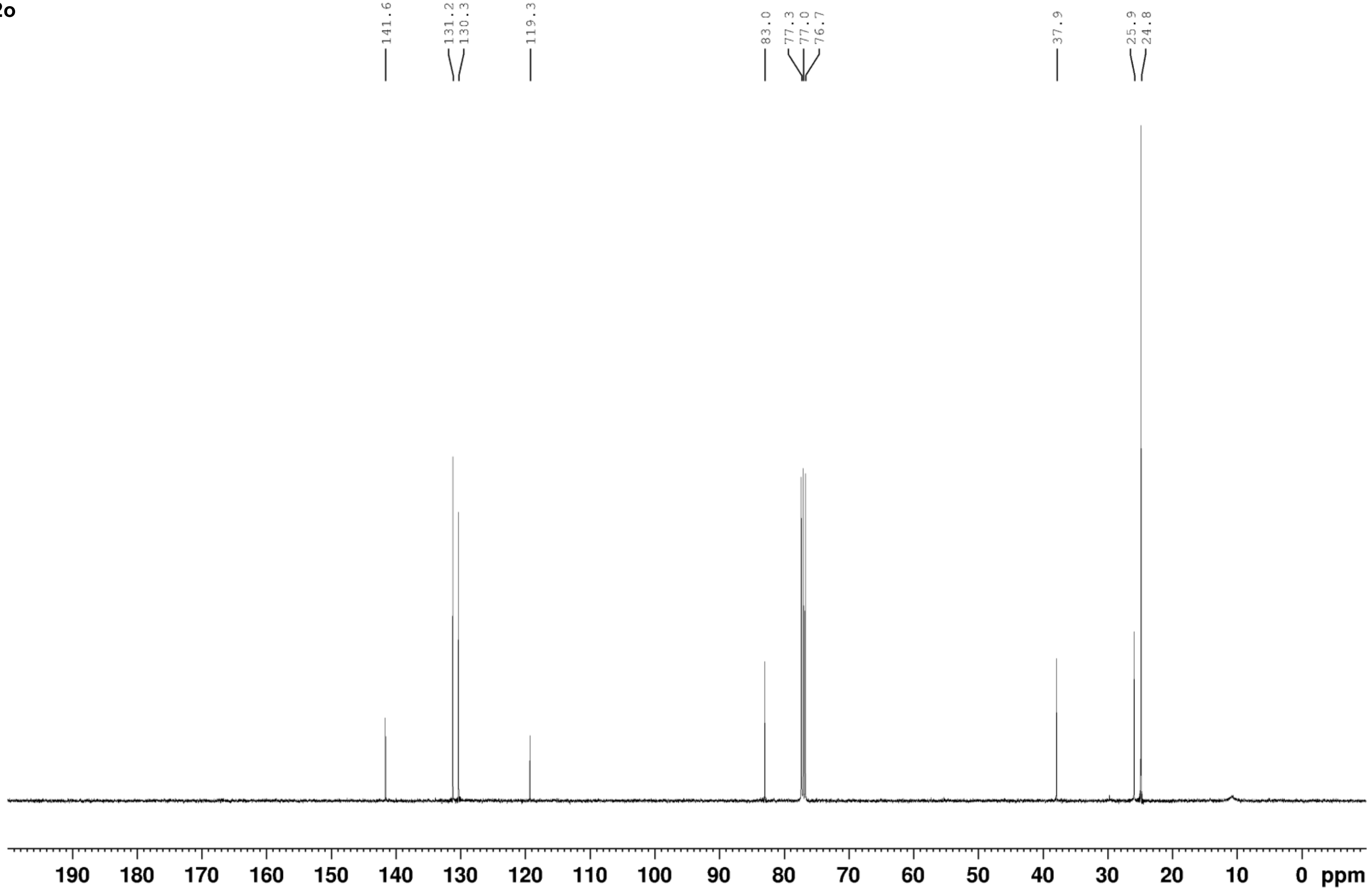

Figure S57. ^{11}B NMR (128 MHz, $CDCl_3$, 298 K) of **2o**.

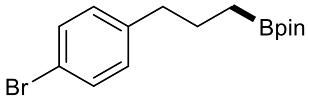

2o

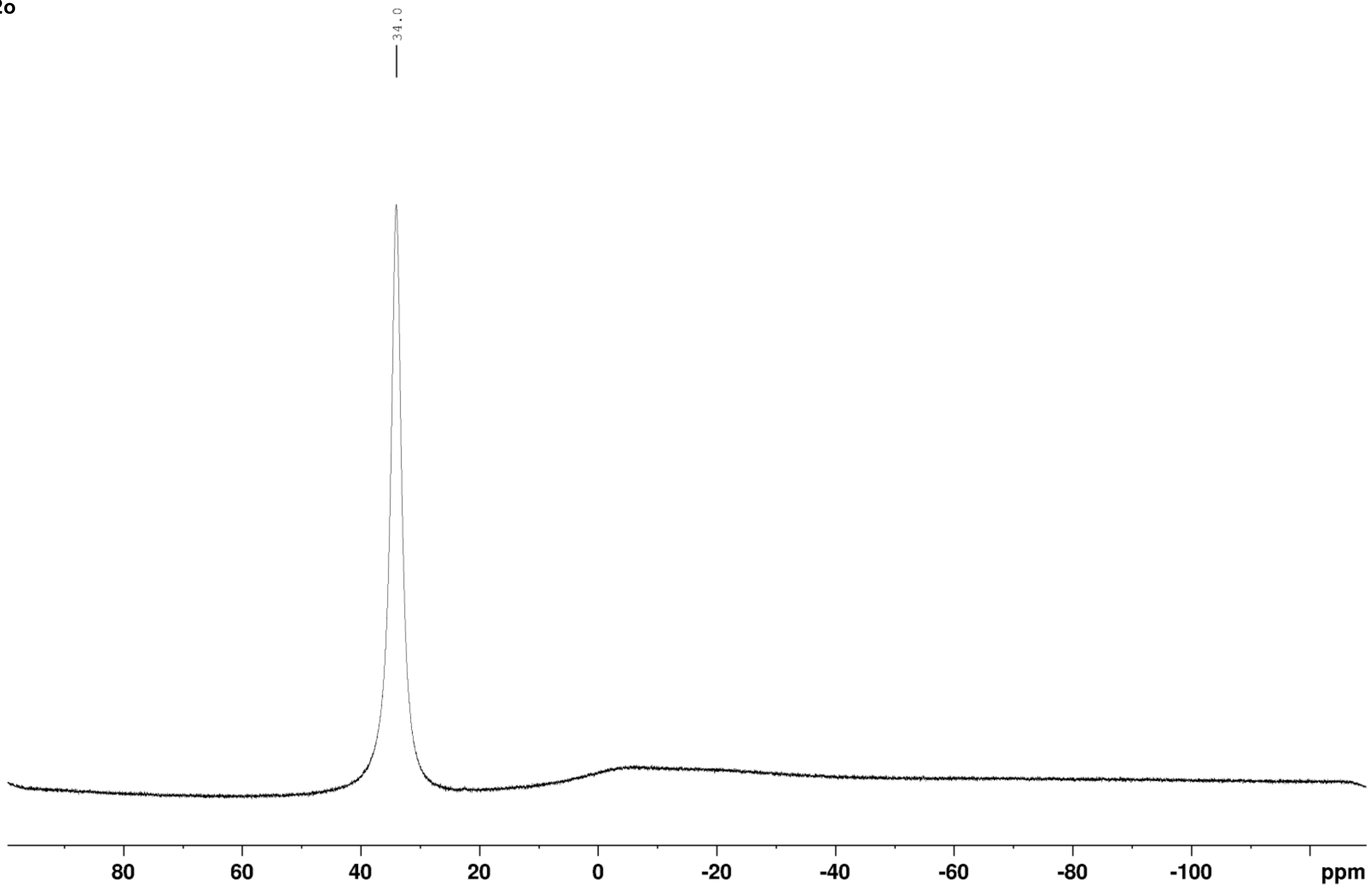

Figure S58. ^{1}H NMR (400 MHz, $CDCl_3$, 298 K) of **2p**.

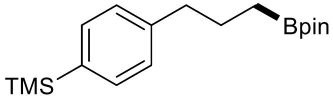

2p

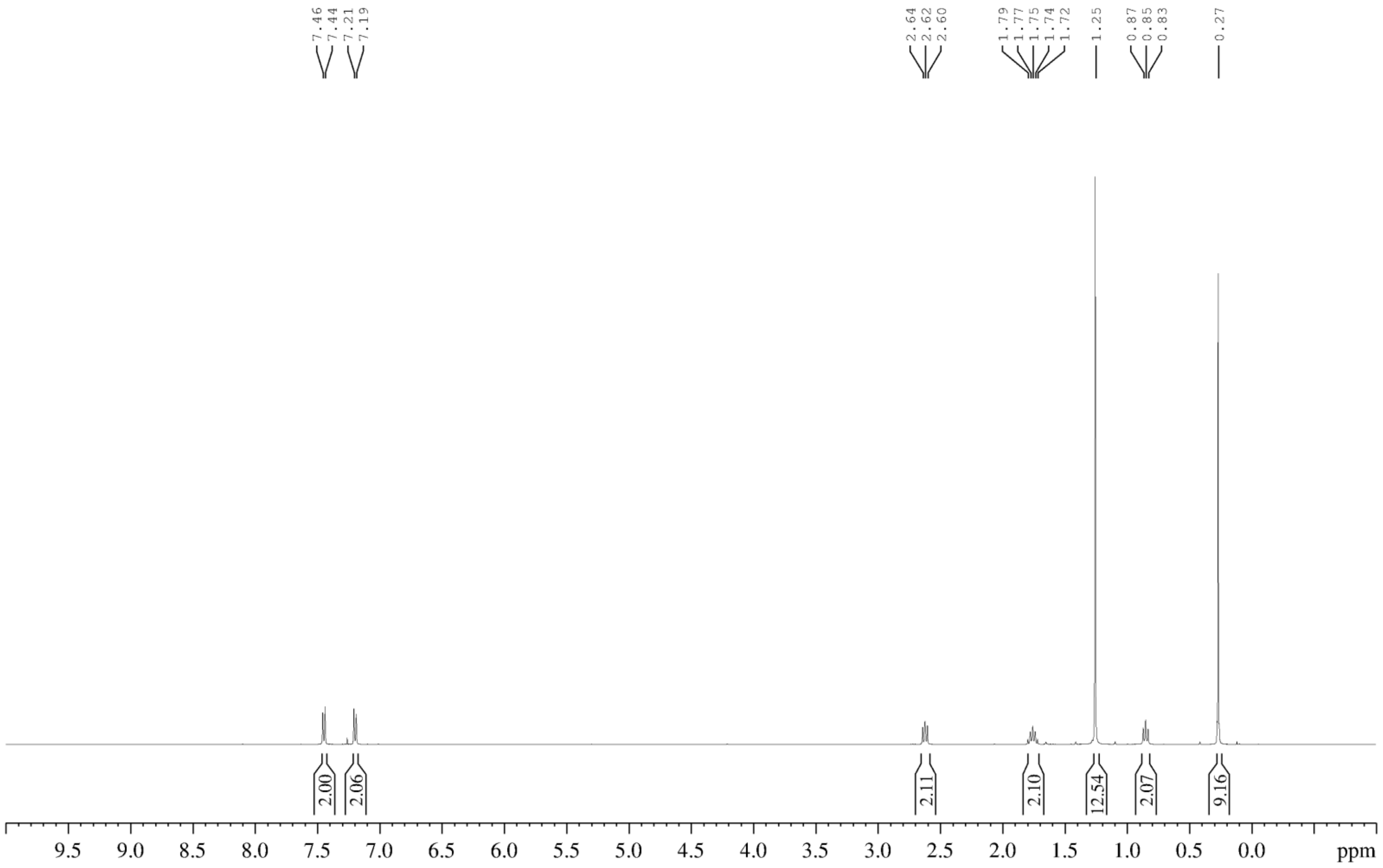

Figure S59. ^{13}C NMR (101 MHz, $CDCl_3$, 298 K) of **2p**.

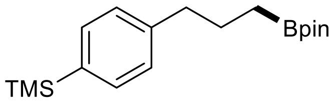

2p

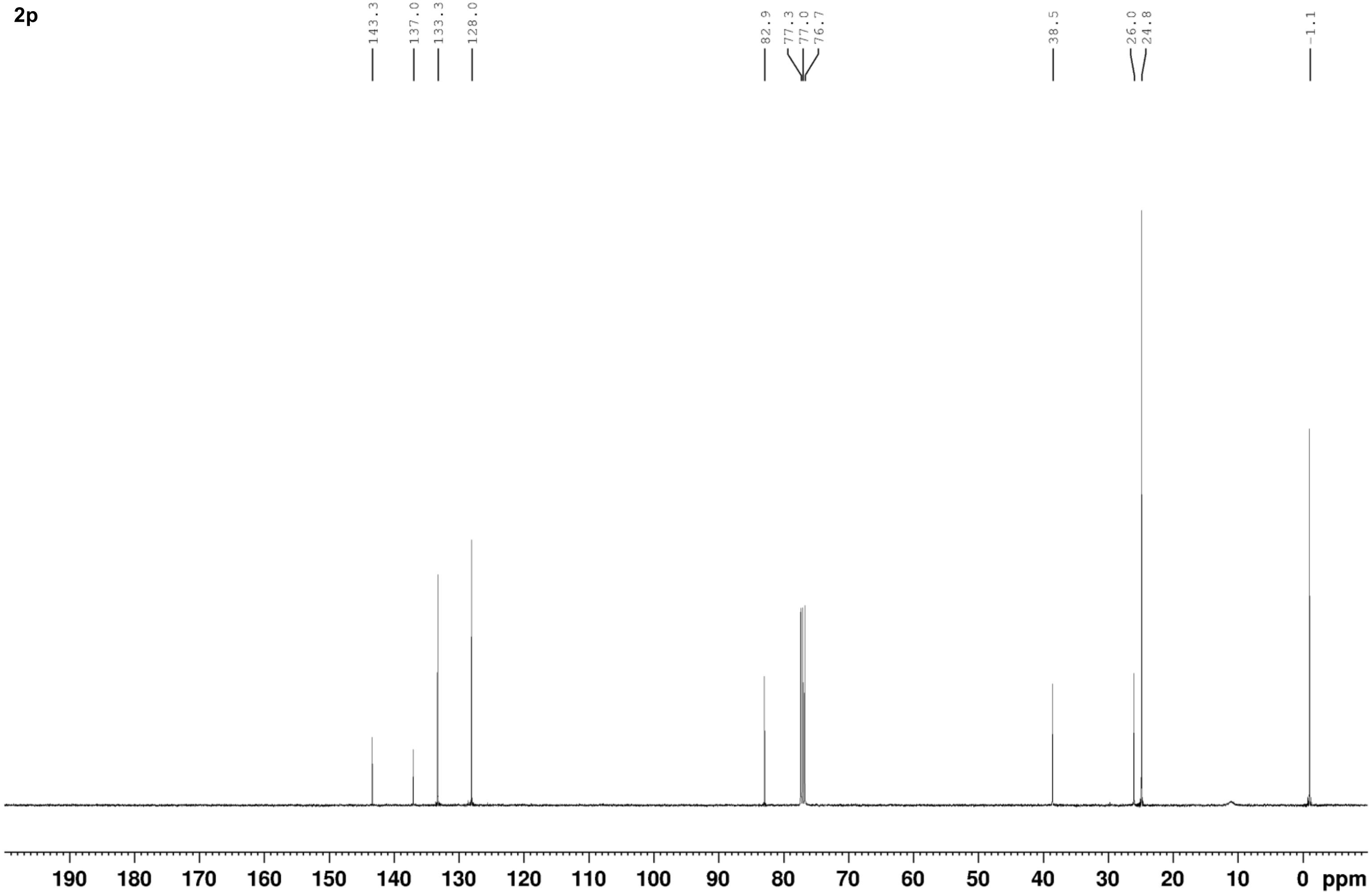

Figure S60. ^{11}B NMR (128 MHz, $CDCl_3$, 298 K) of **2p**.

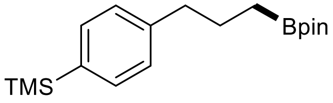

2p

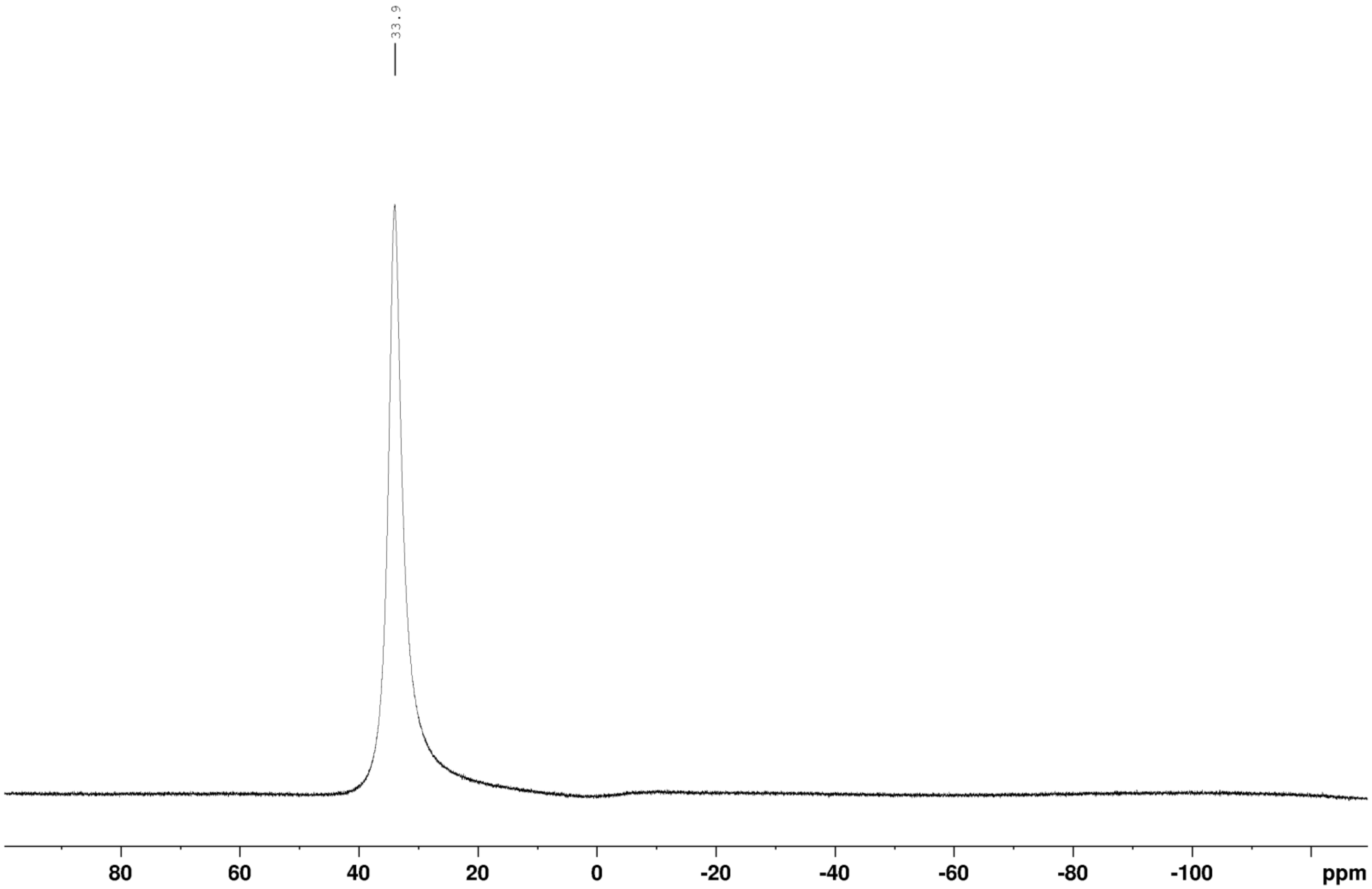

Figure S61. ^{29}Si NMR (79 MHz, $CDCl_3$, 298 K) of **2p**.

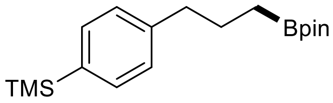

2p

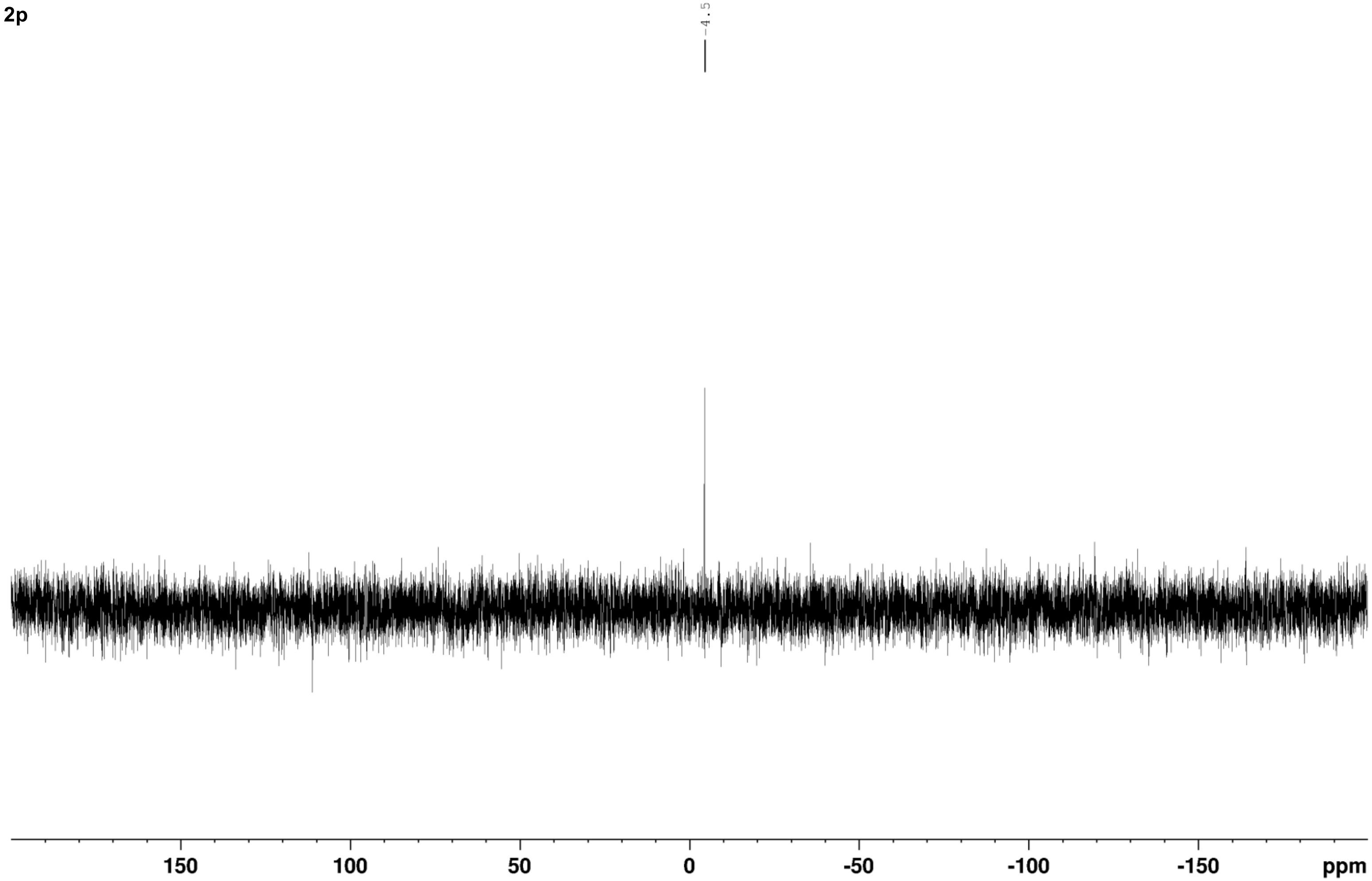

Figure S62. ^{1}H NMR (400 MHz, $CDCl_3$, 298 K) of **4a**.

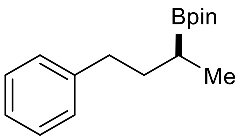

4a

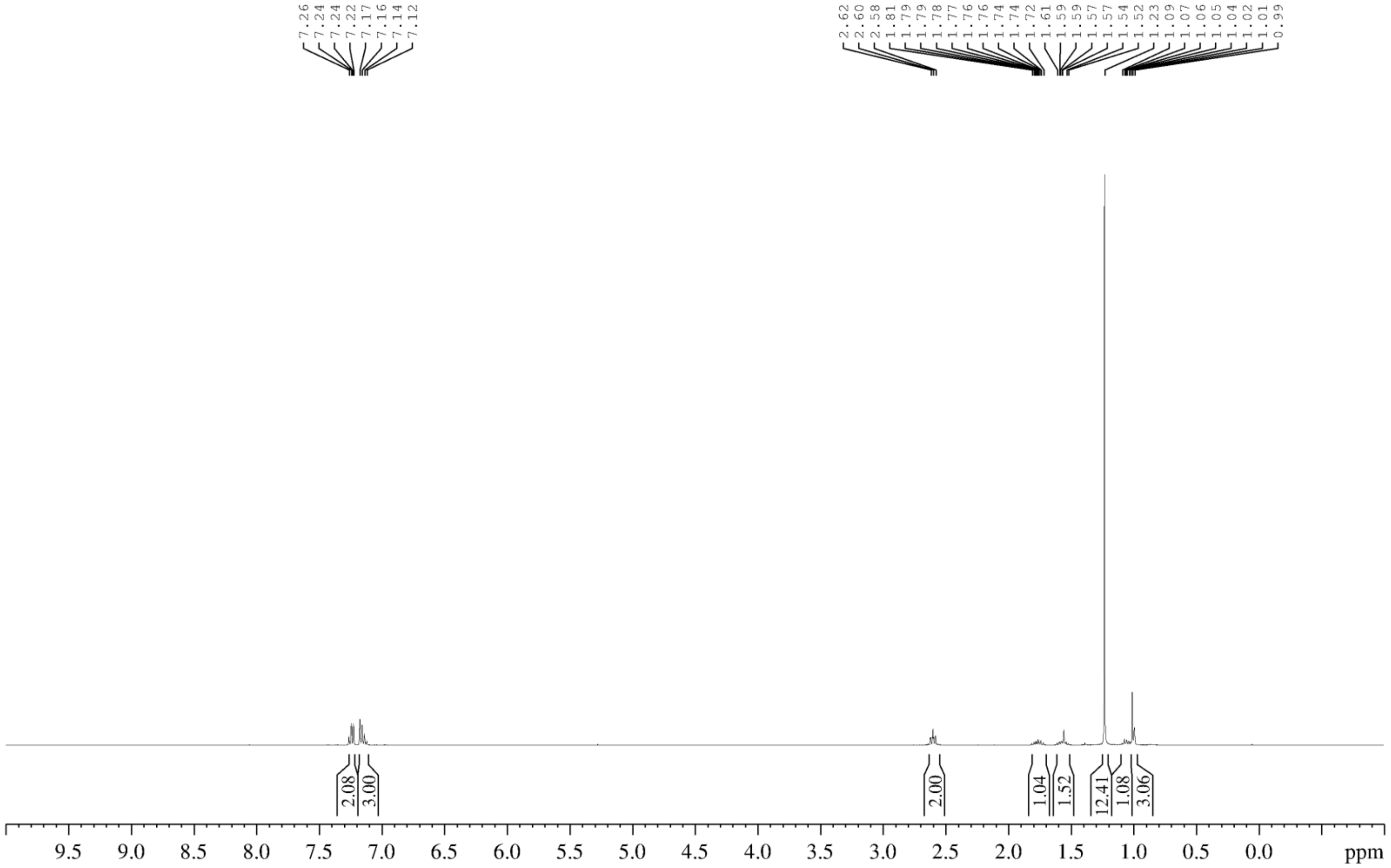

Figure S63. ^{13}C NMR (101 MHz, $CDCl_3$, 298 K) of **4a**.

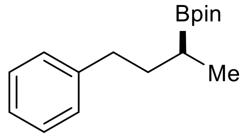

4a

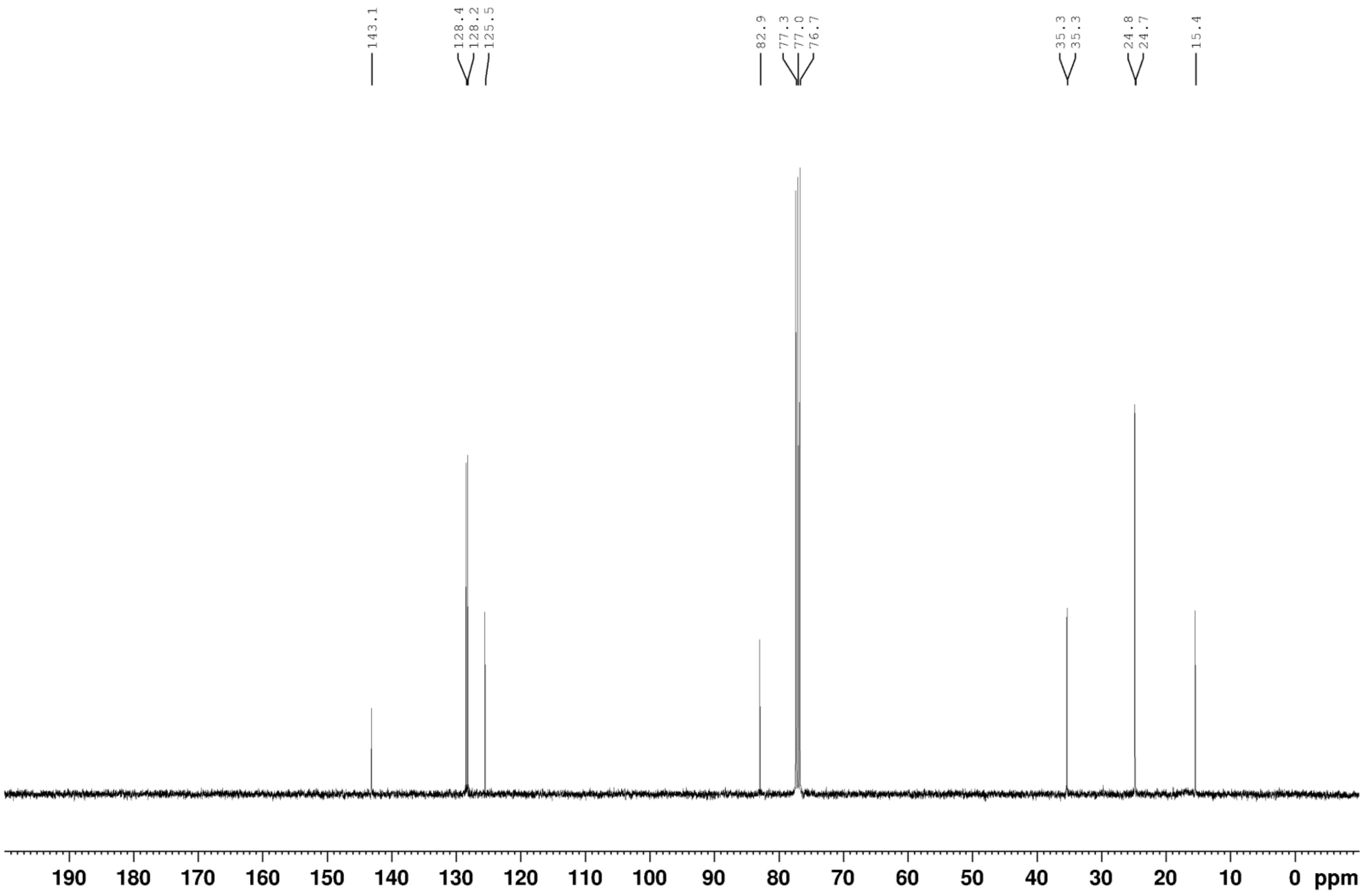

Figure S64. ^{11}B NMR (128 MHz, $CDCl_3$, 298 K) of **4a**.

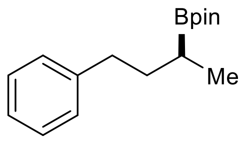

4a

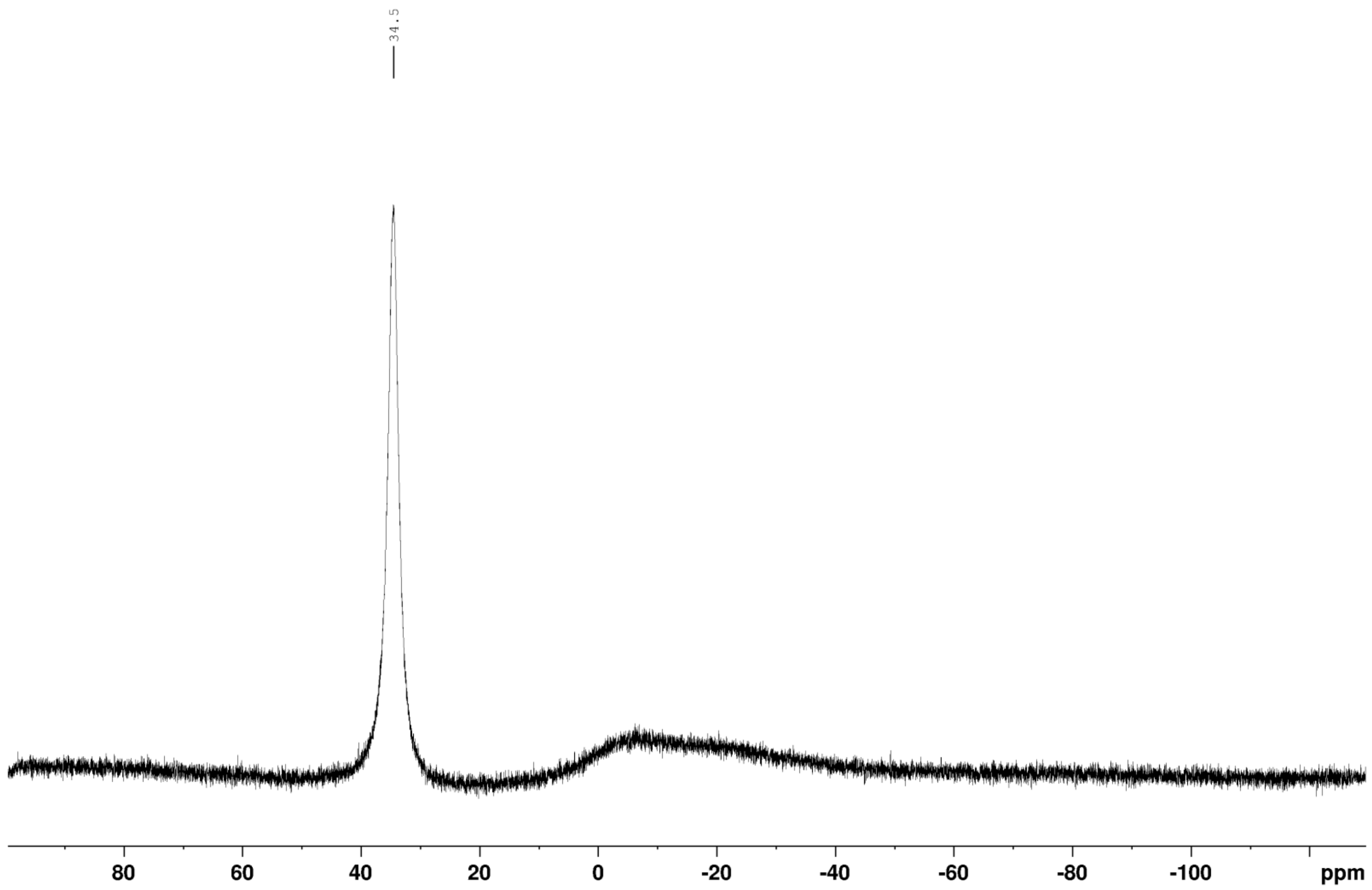

Figure S65. ^{1}H NMR (400 MHz, $CDCl_3$, 298 K) of **4b**.

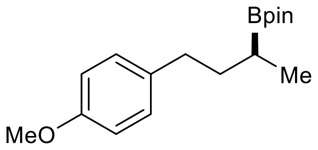

4b

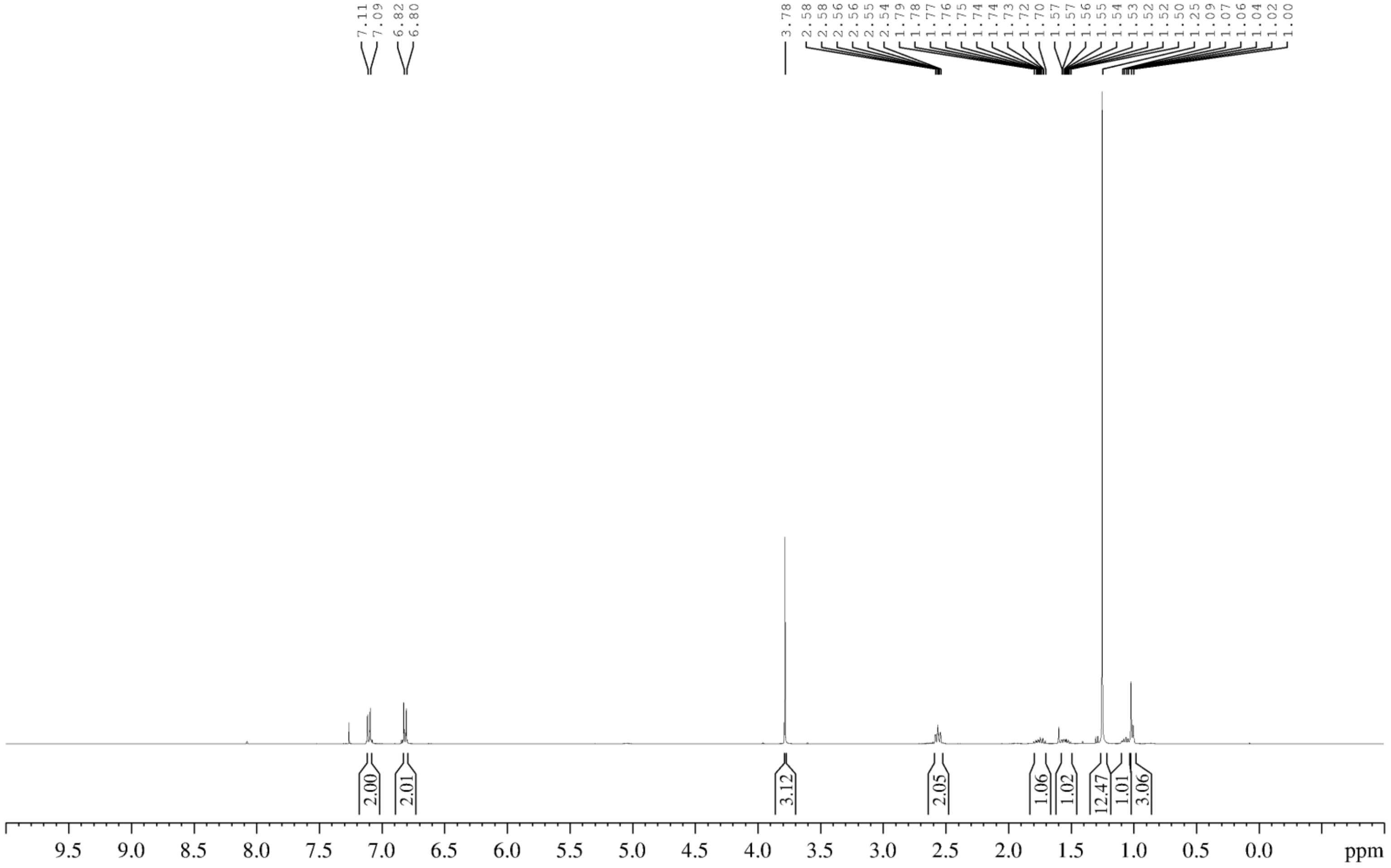

Figure S66. ^{13}C NMR (101 MHz, $CDCl_3$, 298 K) of **4b**.

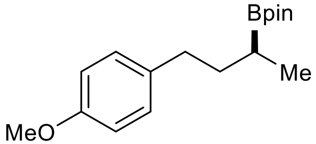

4b

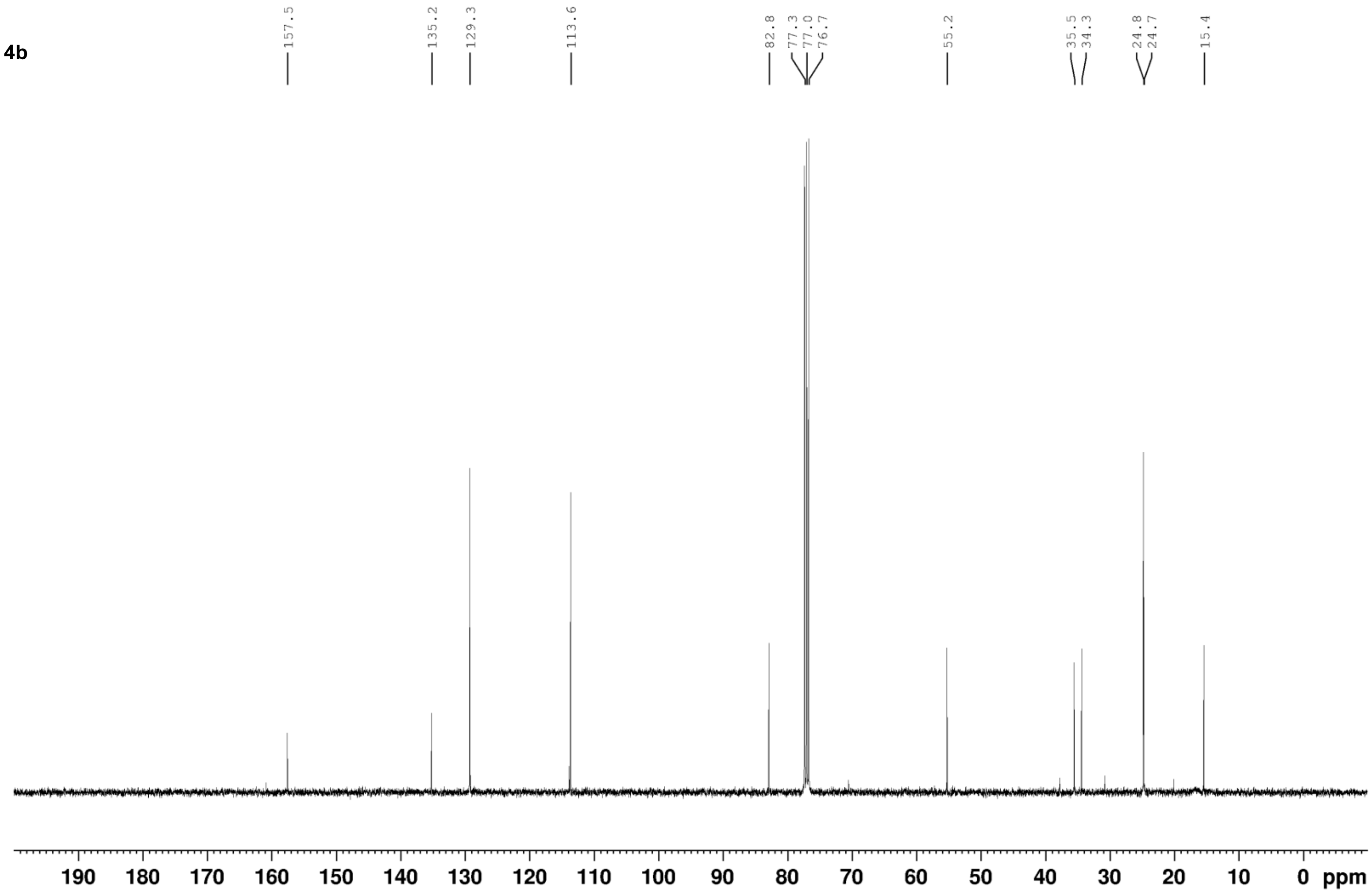

Figure S67. ^{11}B NMR (128 MHz, $CDCl_3$, 298 K) of **4b**.

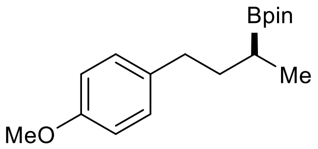

4b

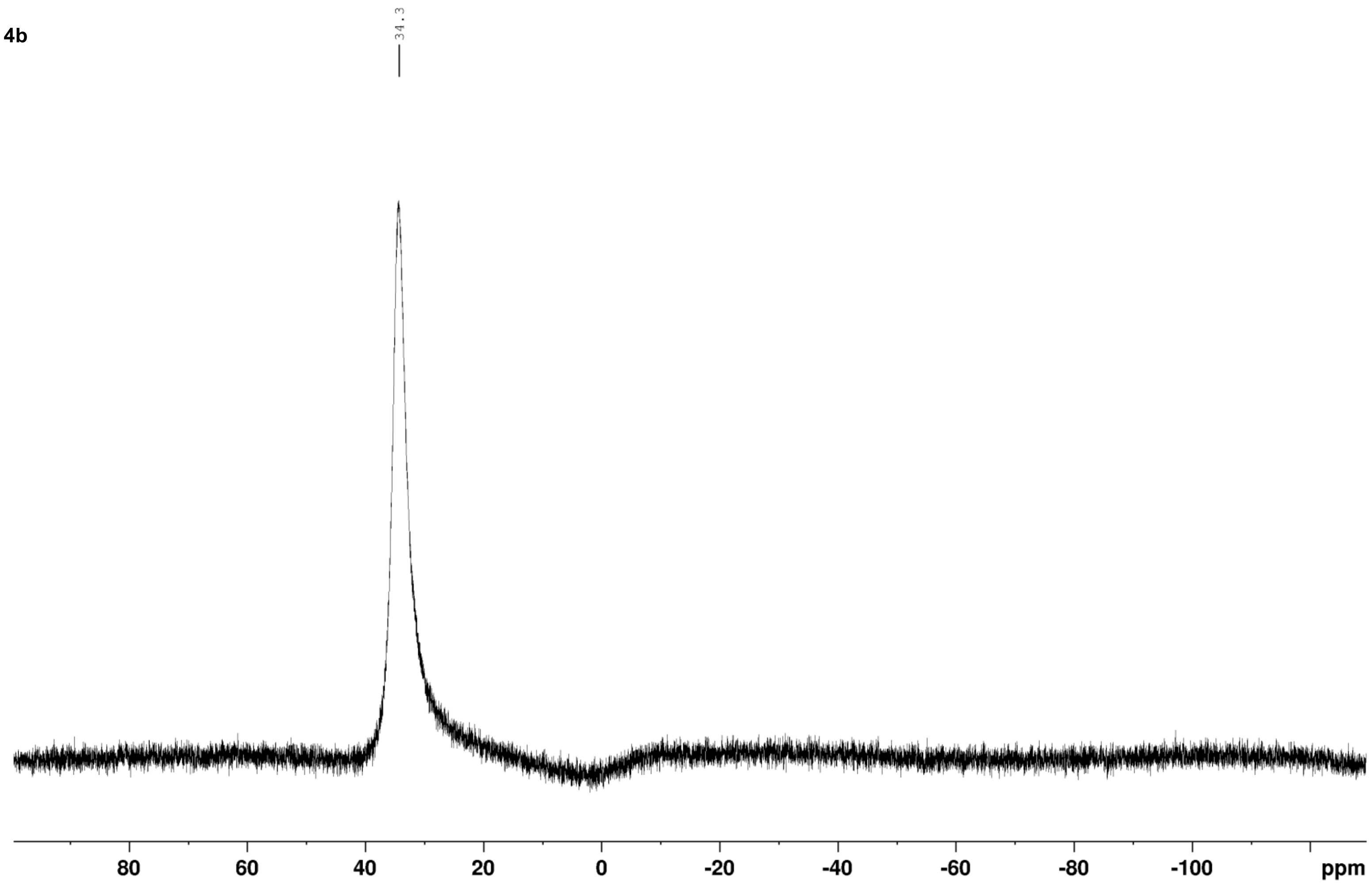

Figure S68. ^{1}H NMR (400 MHz, $CDCl_3$, 298 K) of **4c**.

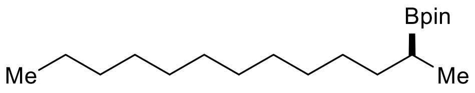

4c

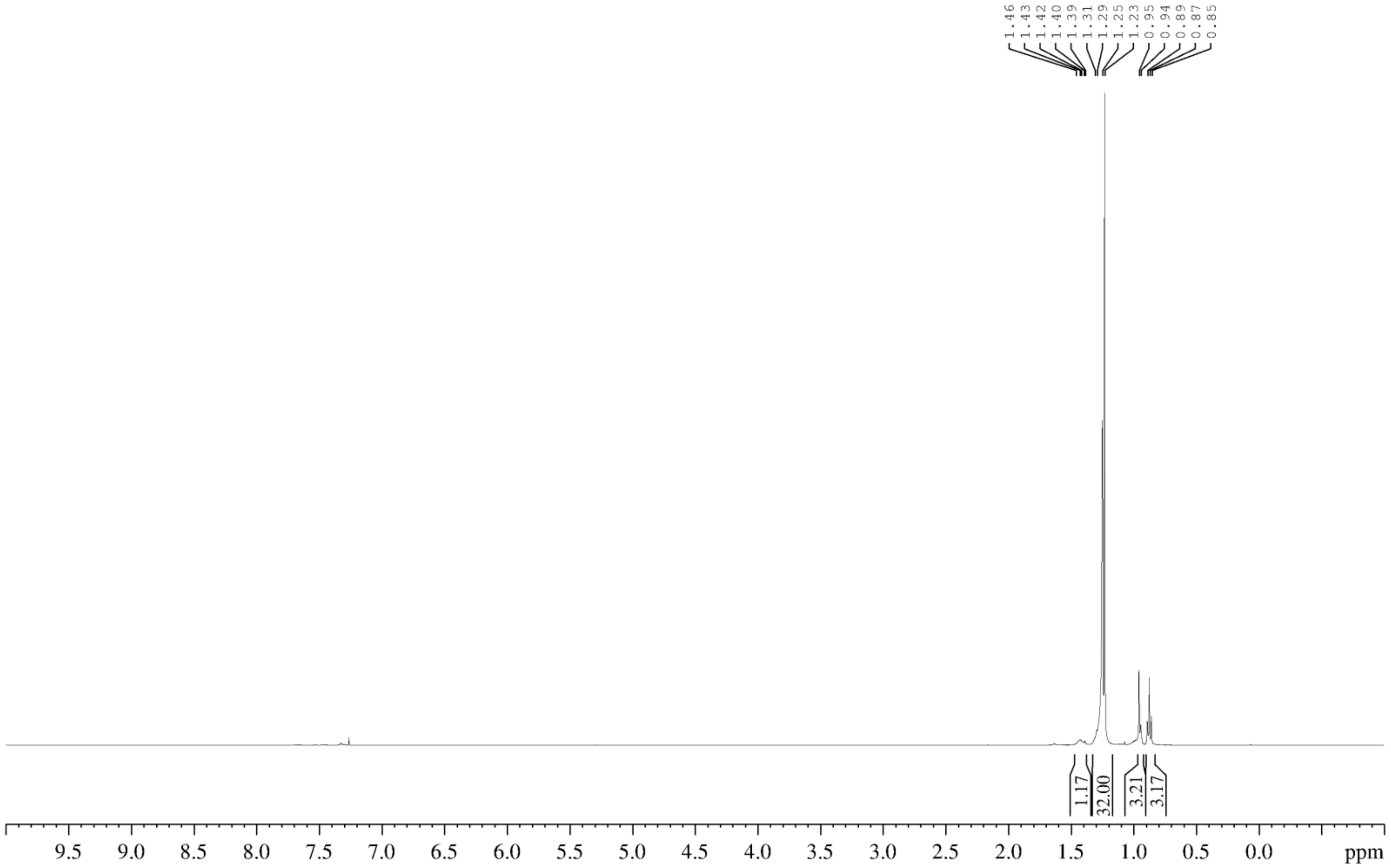

Figure S69. ^{13}C NMR (101 MHz, $CDCl_3$, 298 K) of **4c**.

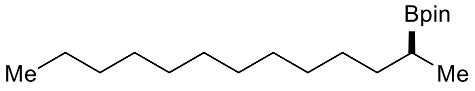

4c

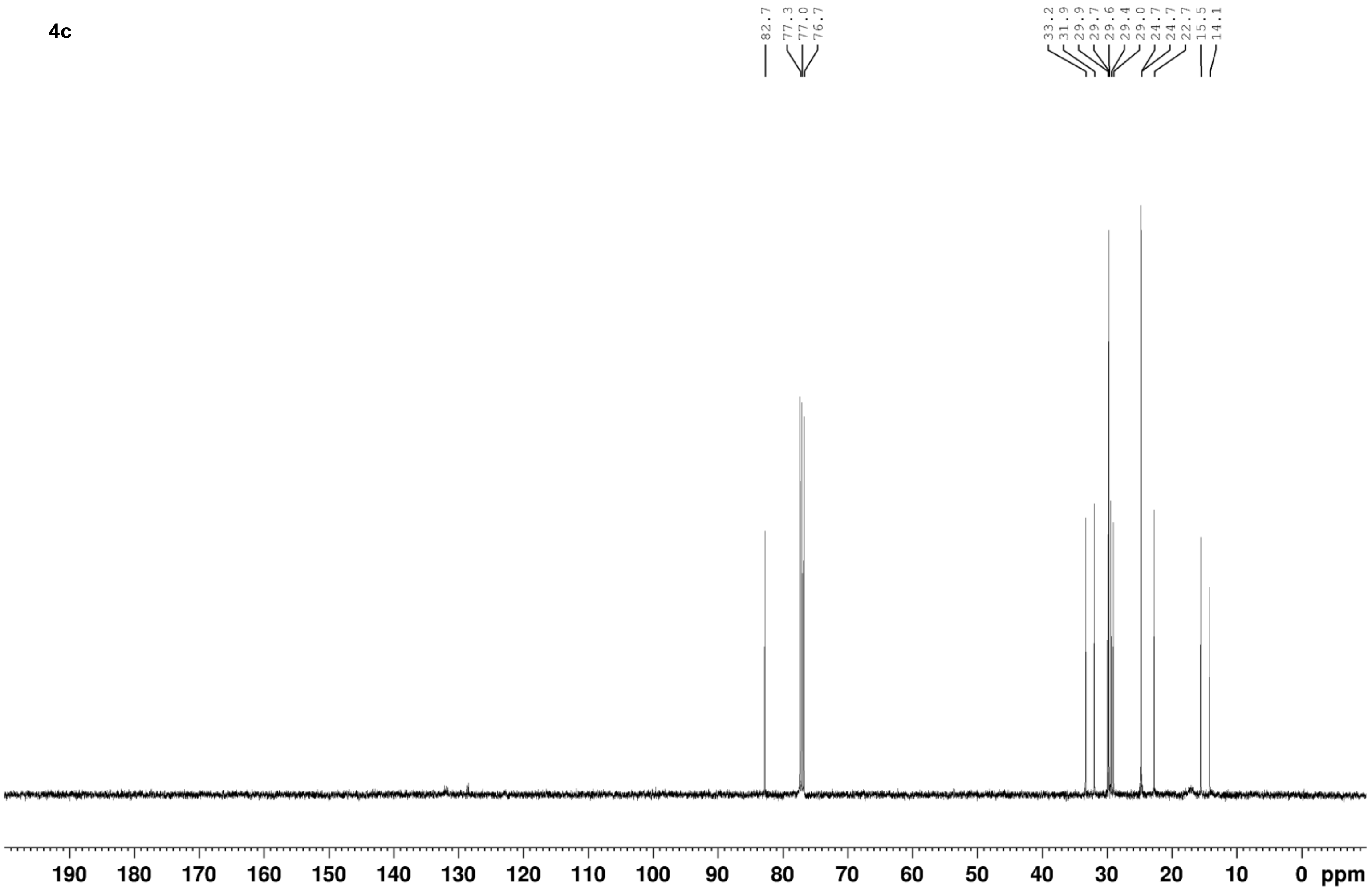

Figure S70. ^{11}B NMR (128 MHz, $CDCl_3$, 298 K) of **4c**.

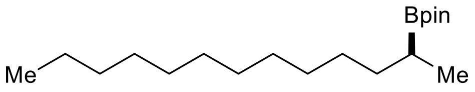

4c

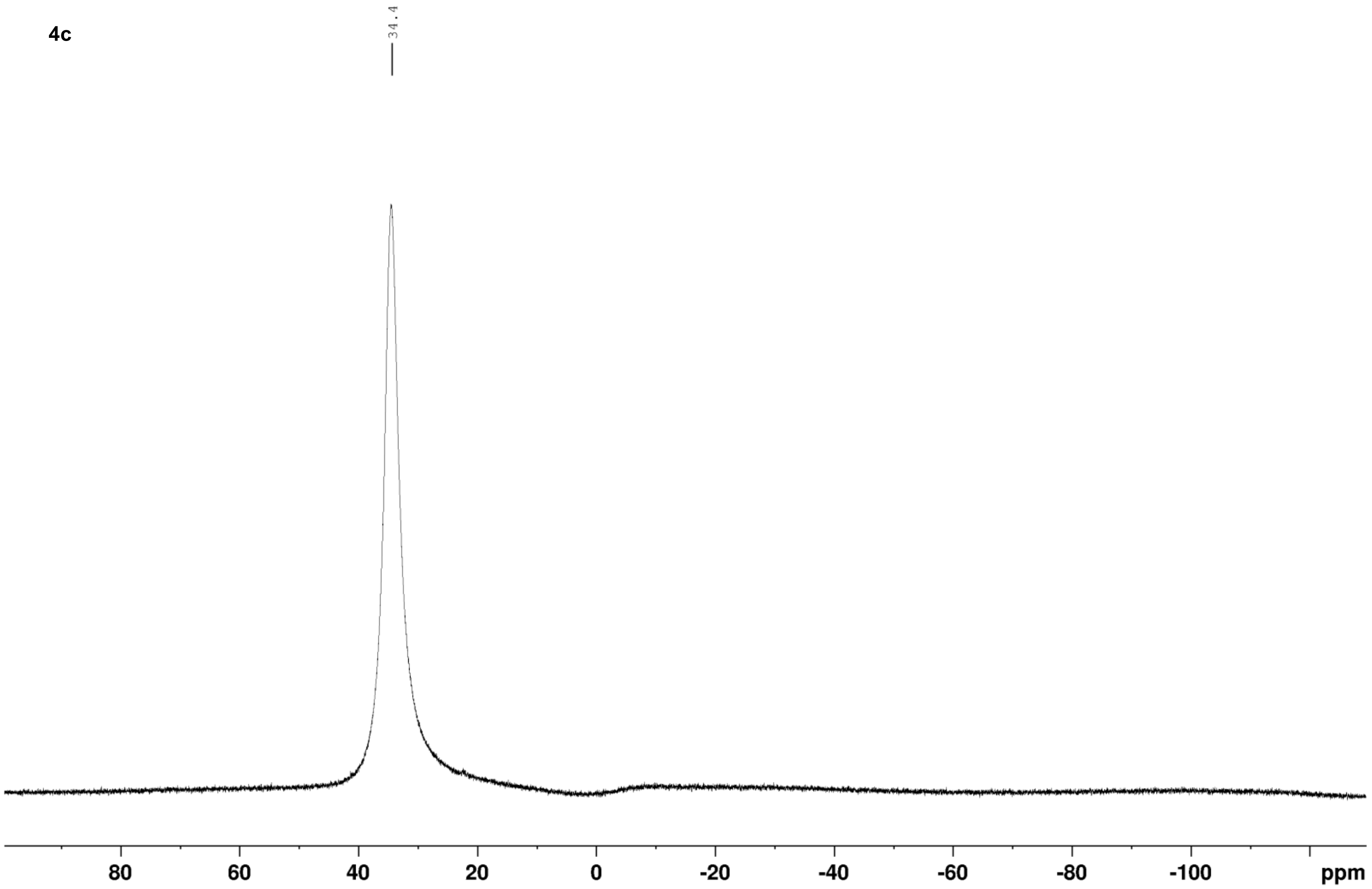

Figure S71. ^{1}H NMR (400 MHz, $CDCl_3$, 298 K) of **4d**.

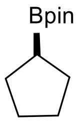

4d

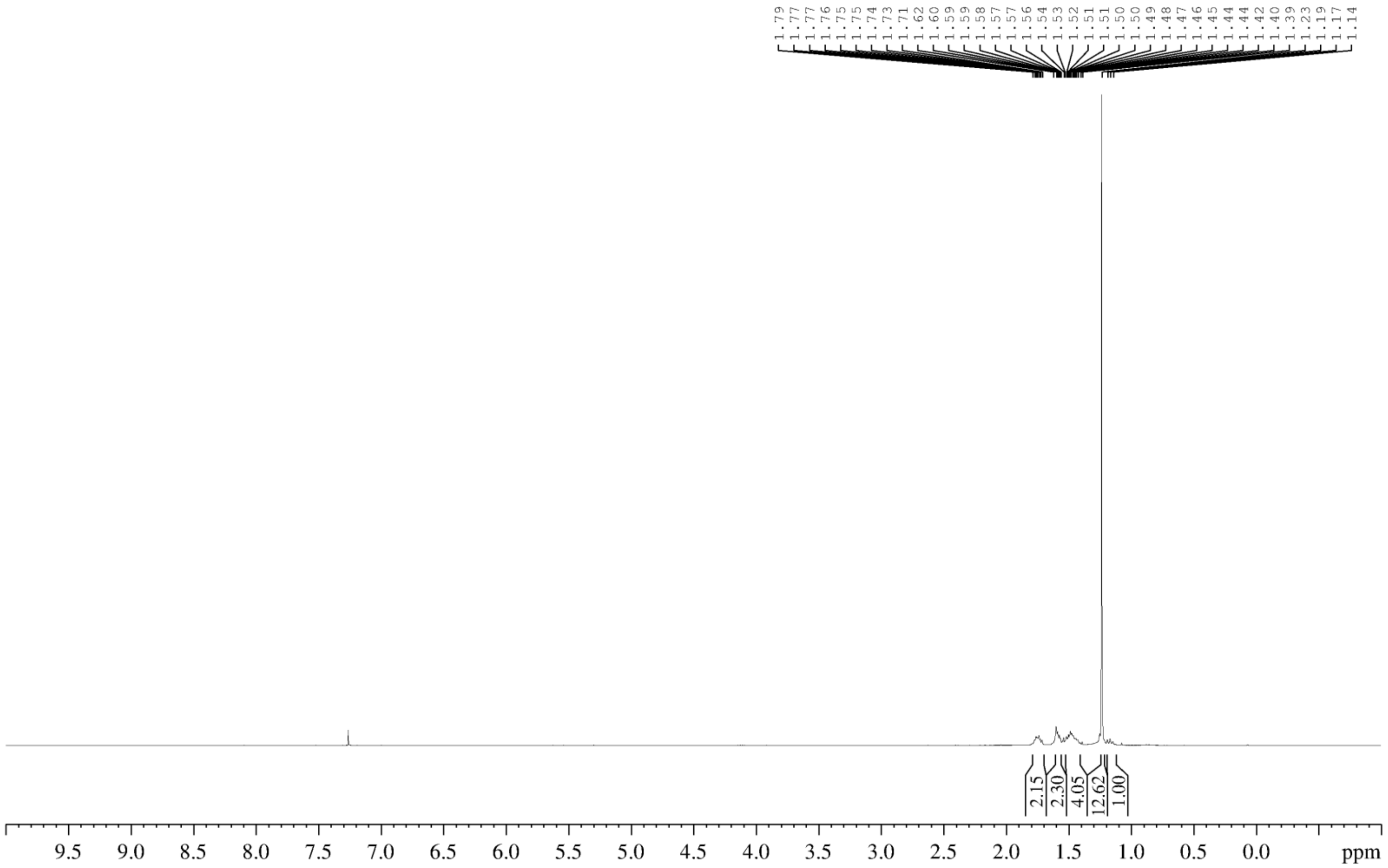

Figure S72. ^{13}C NMR (101 MHz, $CDCl_3$, 298 K) of **4d**.

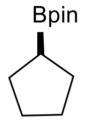

4d

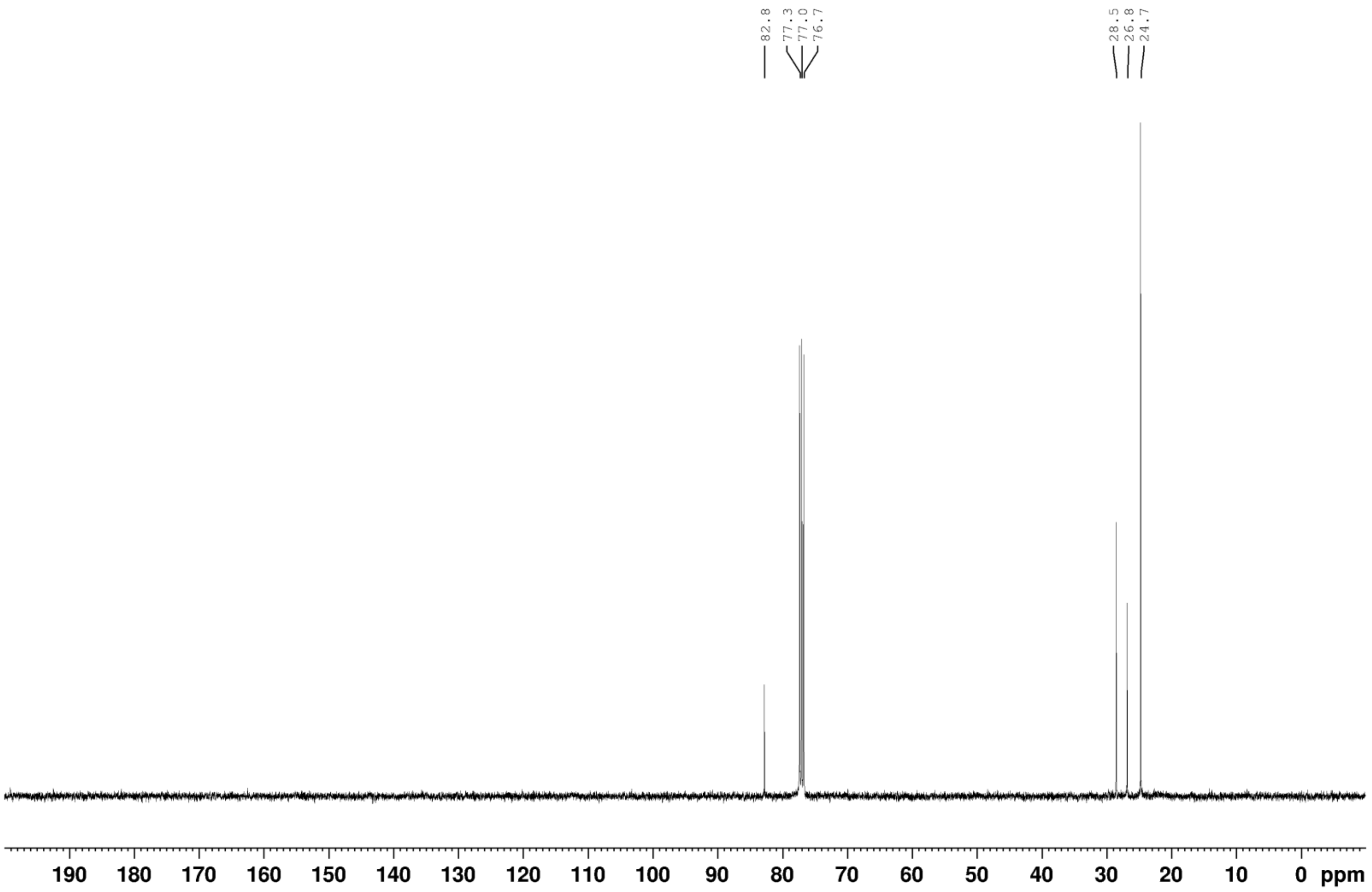

Figure S73. ^{11}B NMR (128 MHz, $CDCl_3$, 298 K) of **4d**.

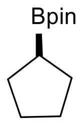

4d

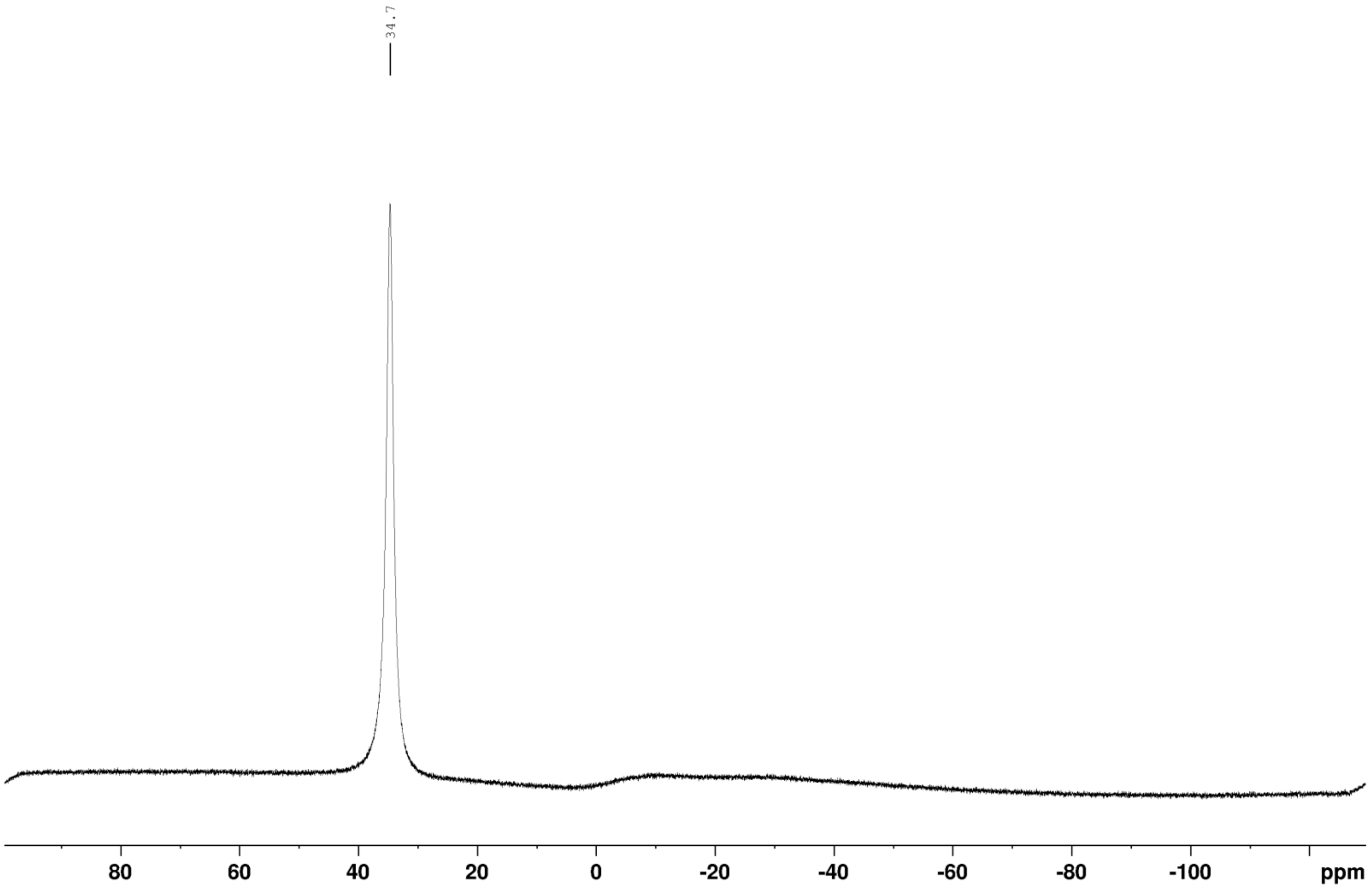

Figure S74. ^{1}H NMR (400 MHz, $CDCl_3$, 298 K) of **4e**.

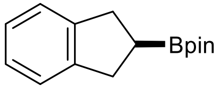

4e

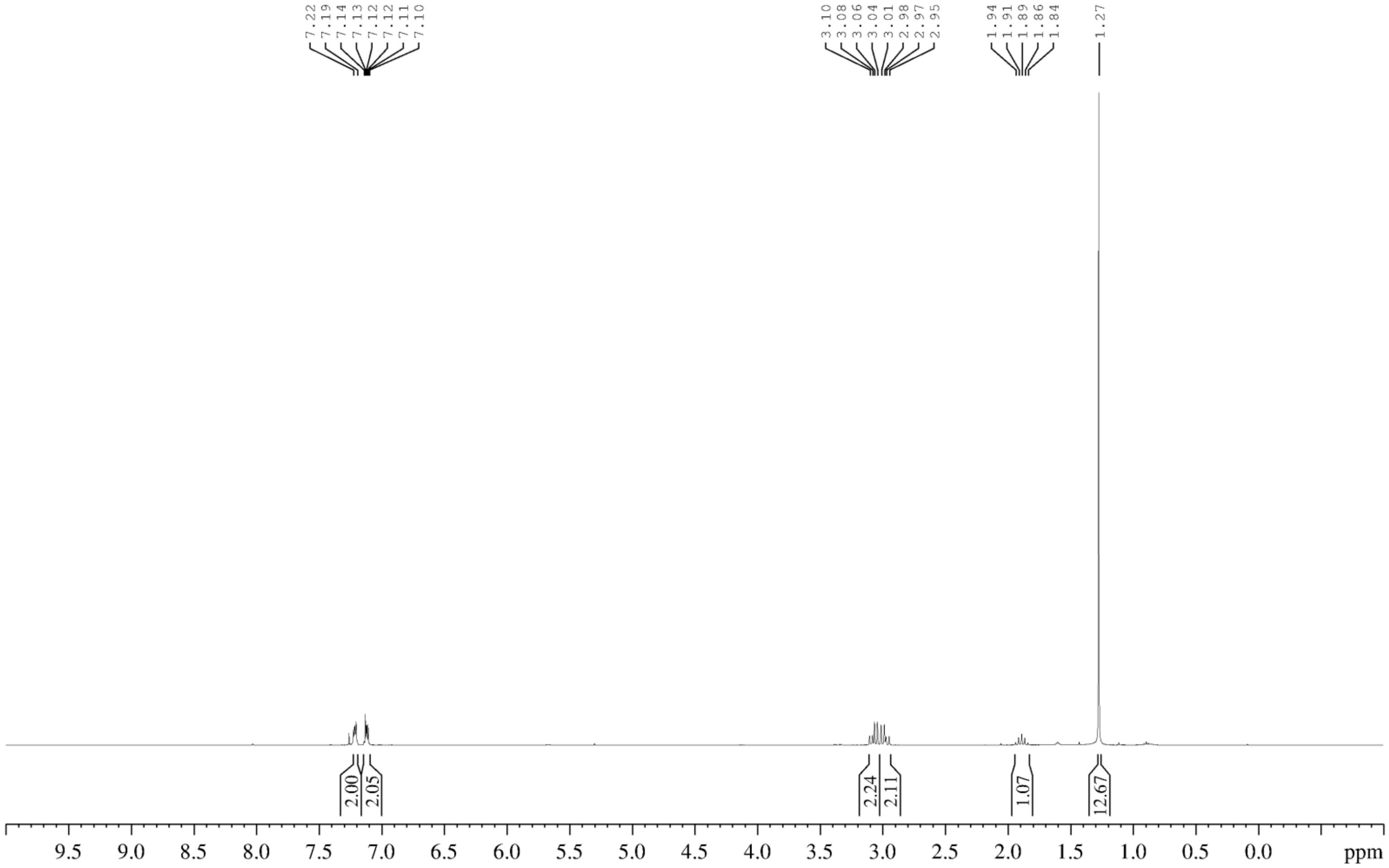

Figure S75. ^{13}C NMR (101 MHz, $CDCl_3$, 298 K) of **4e**.

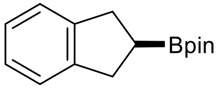

4e

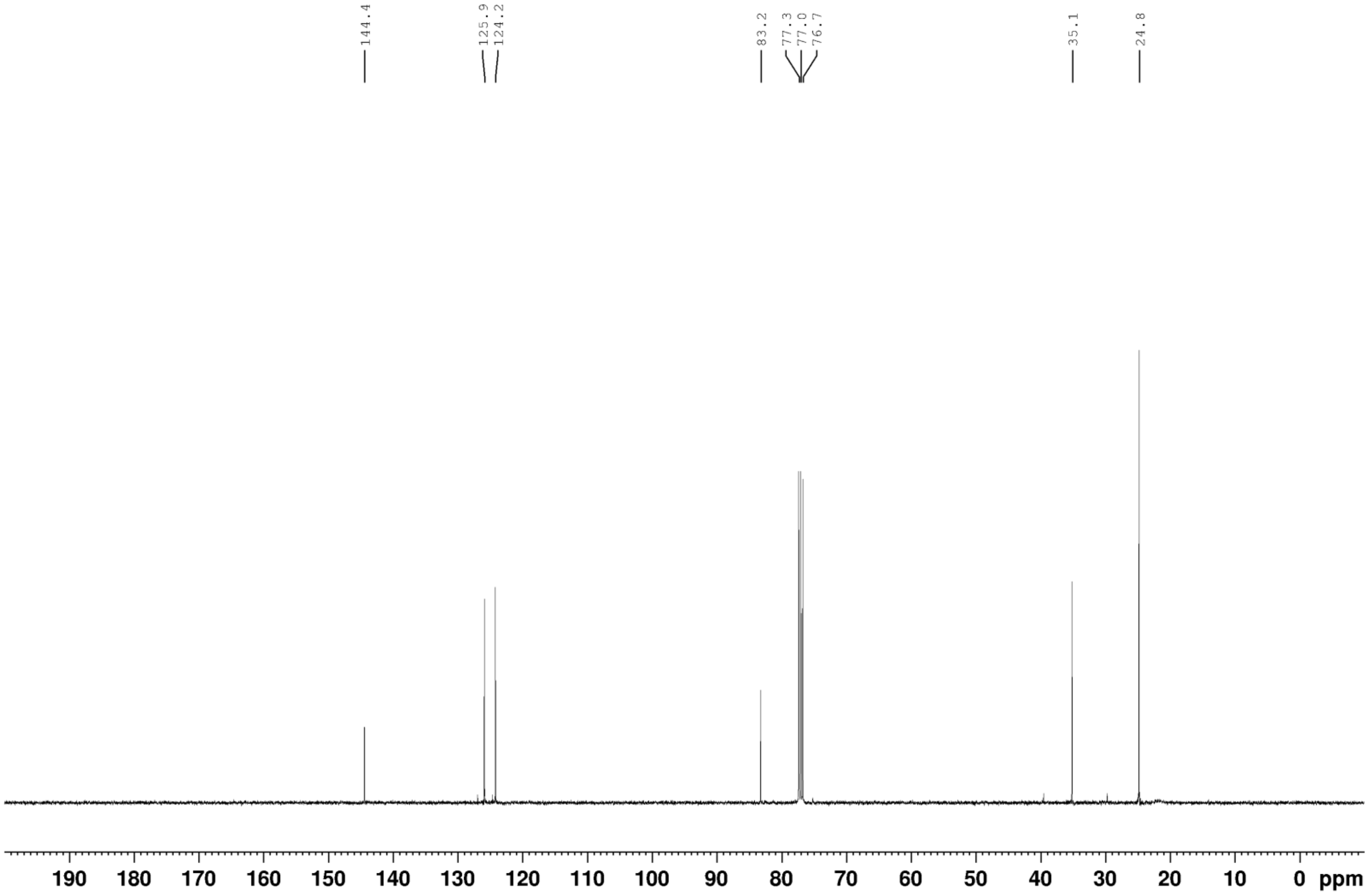

Figure S76. ^{11}B NMR (128 MHz, $CDCl_3$, 298 K) of **4e**.

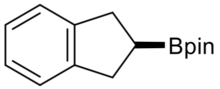

4e

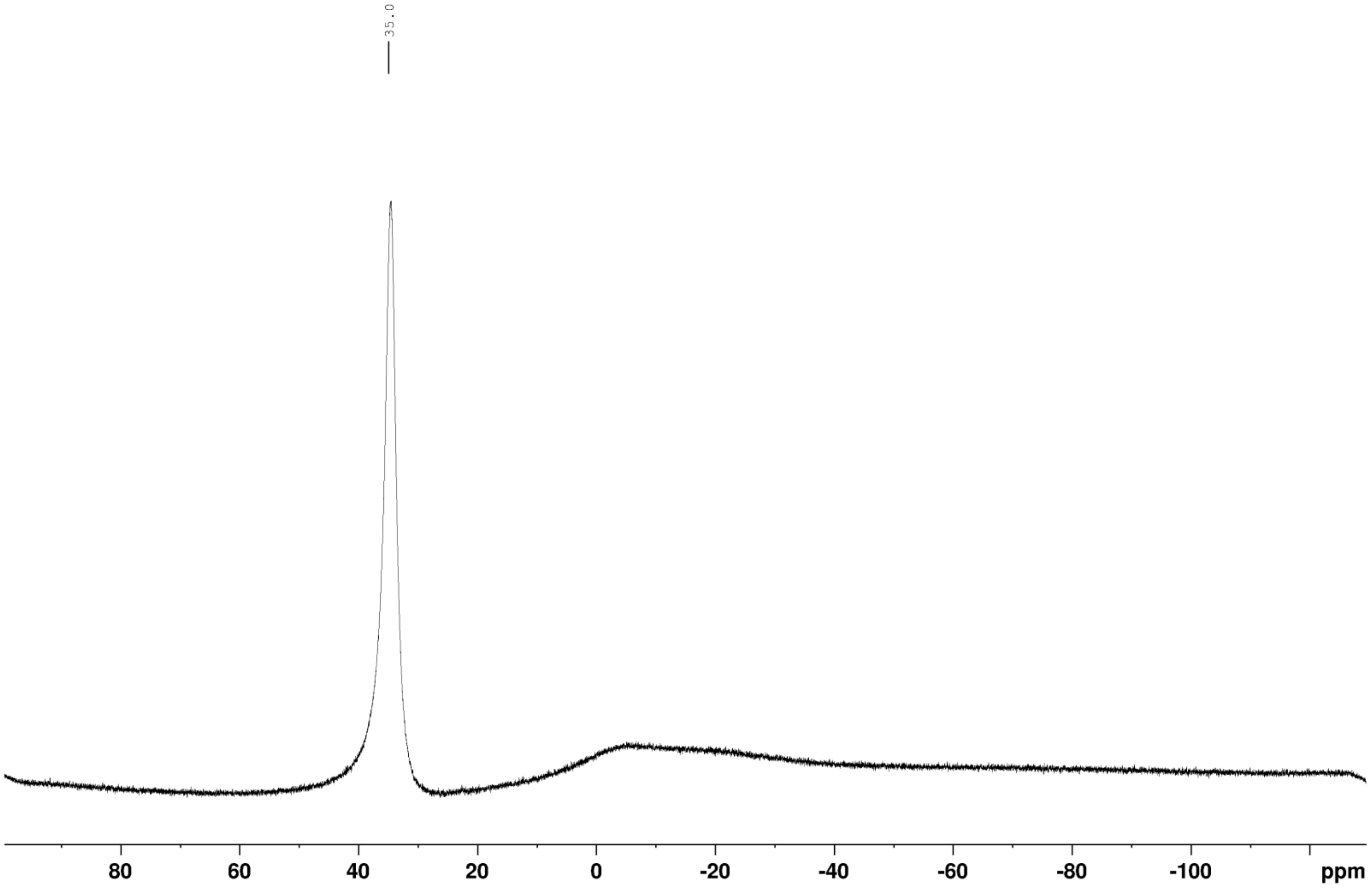

Figure S77. ^{1}H NMR (400 MHz, $CDCl_3$, 298 K) of **4f** as a mixture (*trans*:*cis* = 88:12).

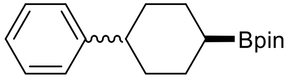

4f

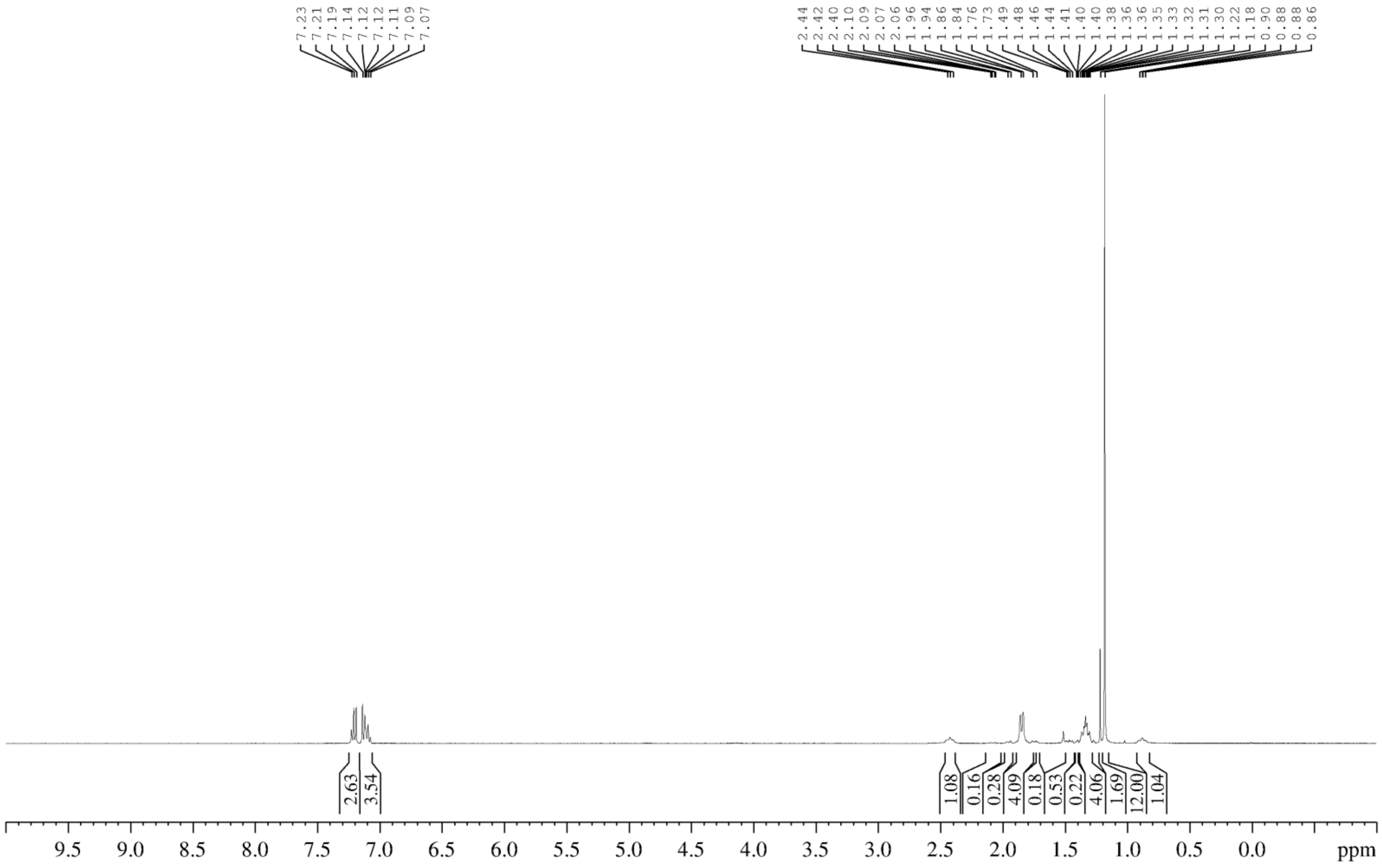

Figure S78. ^{13}C NMR (101 MHz, $CDCl_3$, 298 K) of **4f** as a mixture (*trans*:*cis* = 88:12).

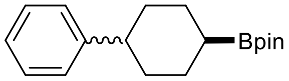

4f

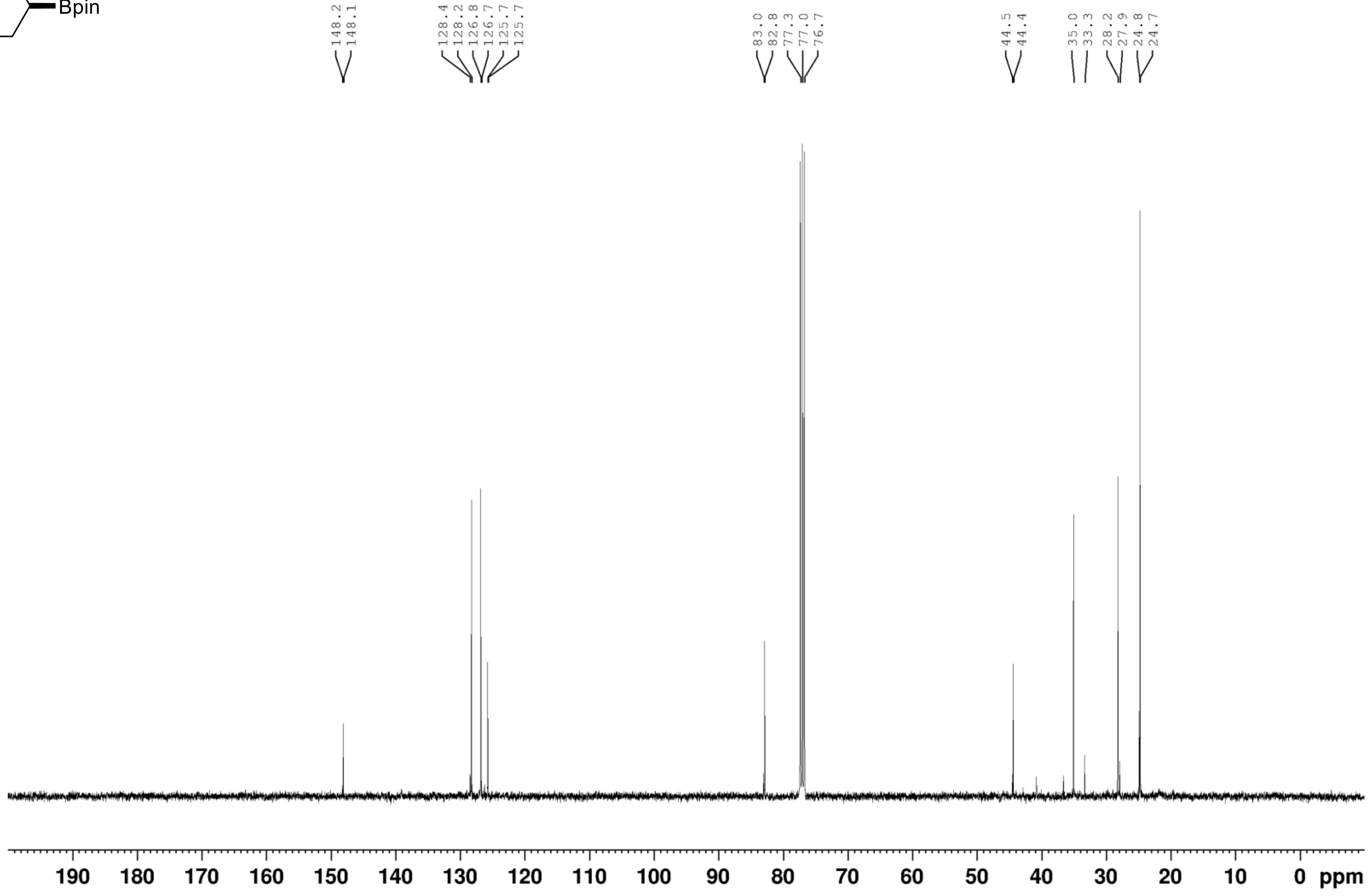

Figure S79. ^{11}B NMR (128 MHz, $CDCl_3$, 298 K) of **4f**.

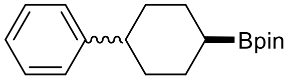

4f

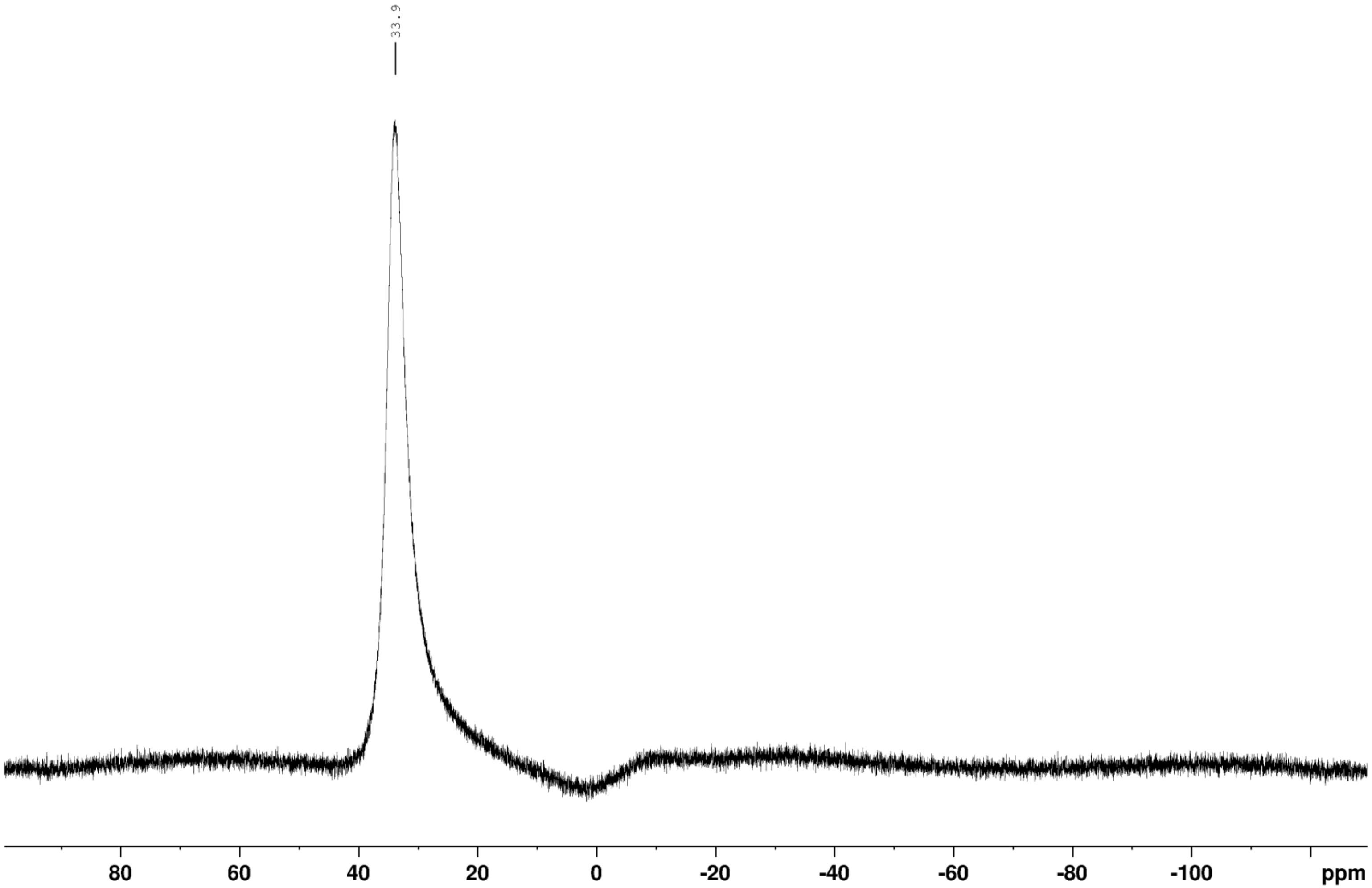

Figure S80. ^1H NMR (400 MHz, $CDCl_3$, 298 K) of **6**.

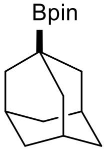

6

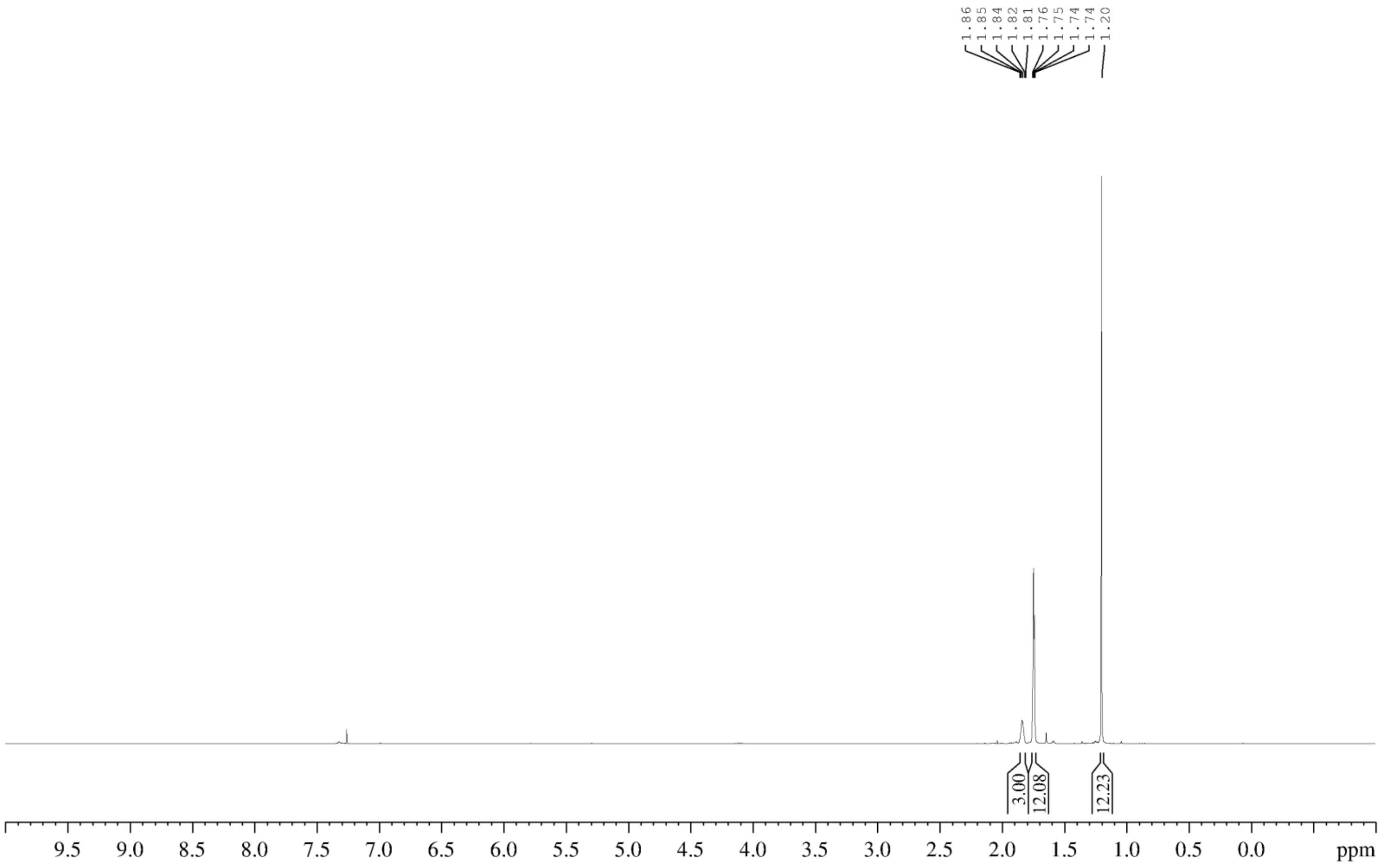

Figure S81. ^{13}C NMR (101 MHz, $CDCl_3$, 298 K) of **6**.

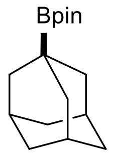

6

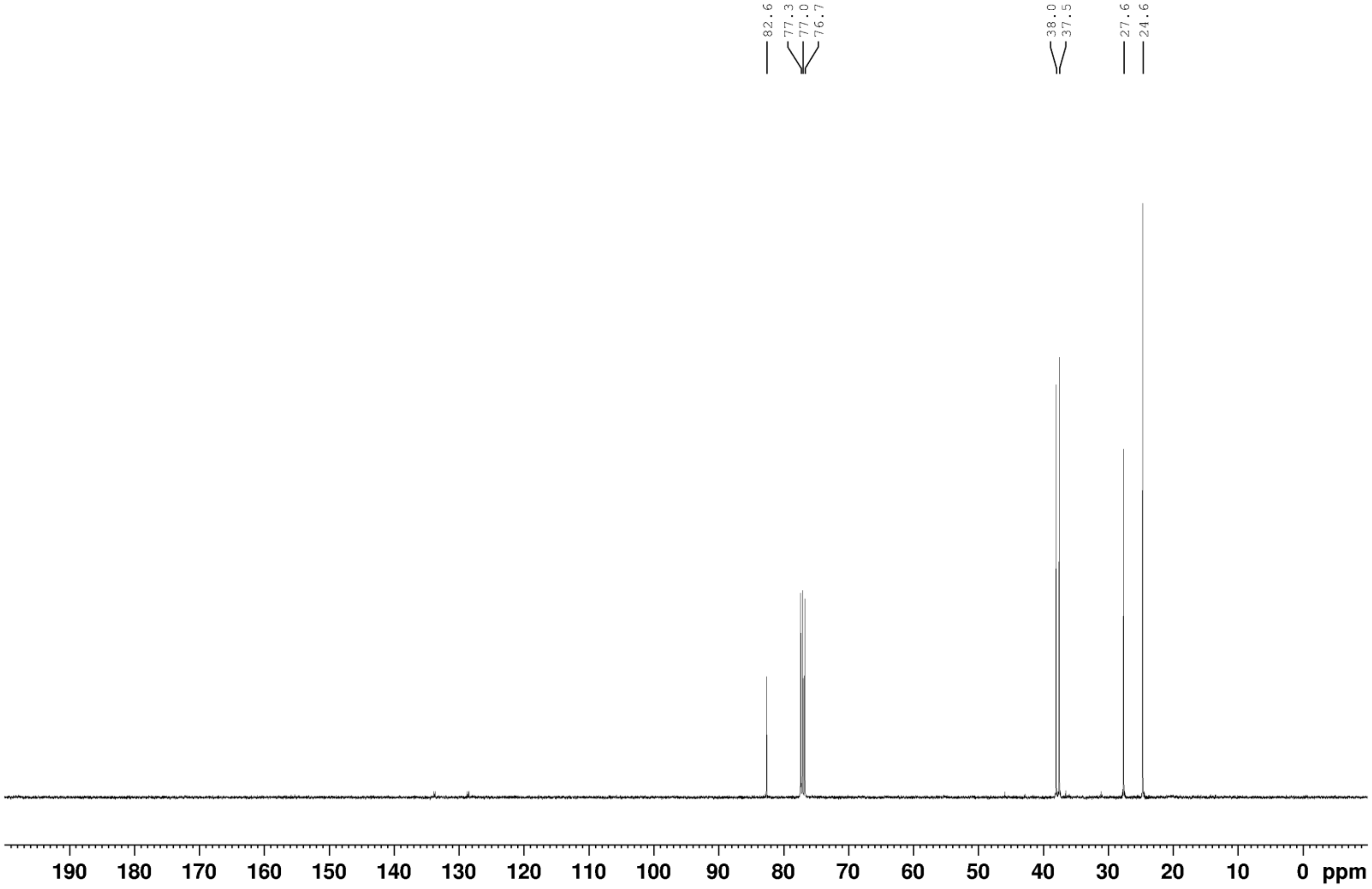

Figure S82. ^{11}B NMR (128 MHz, $CDCl_3$, 298 K) of **6**.

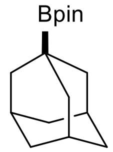

6

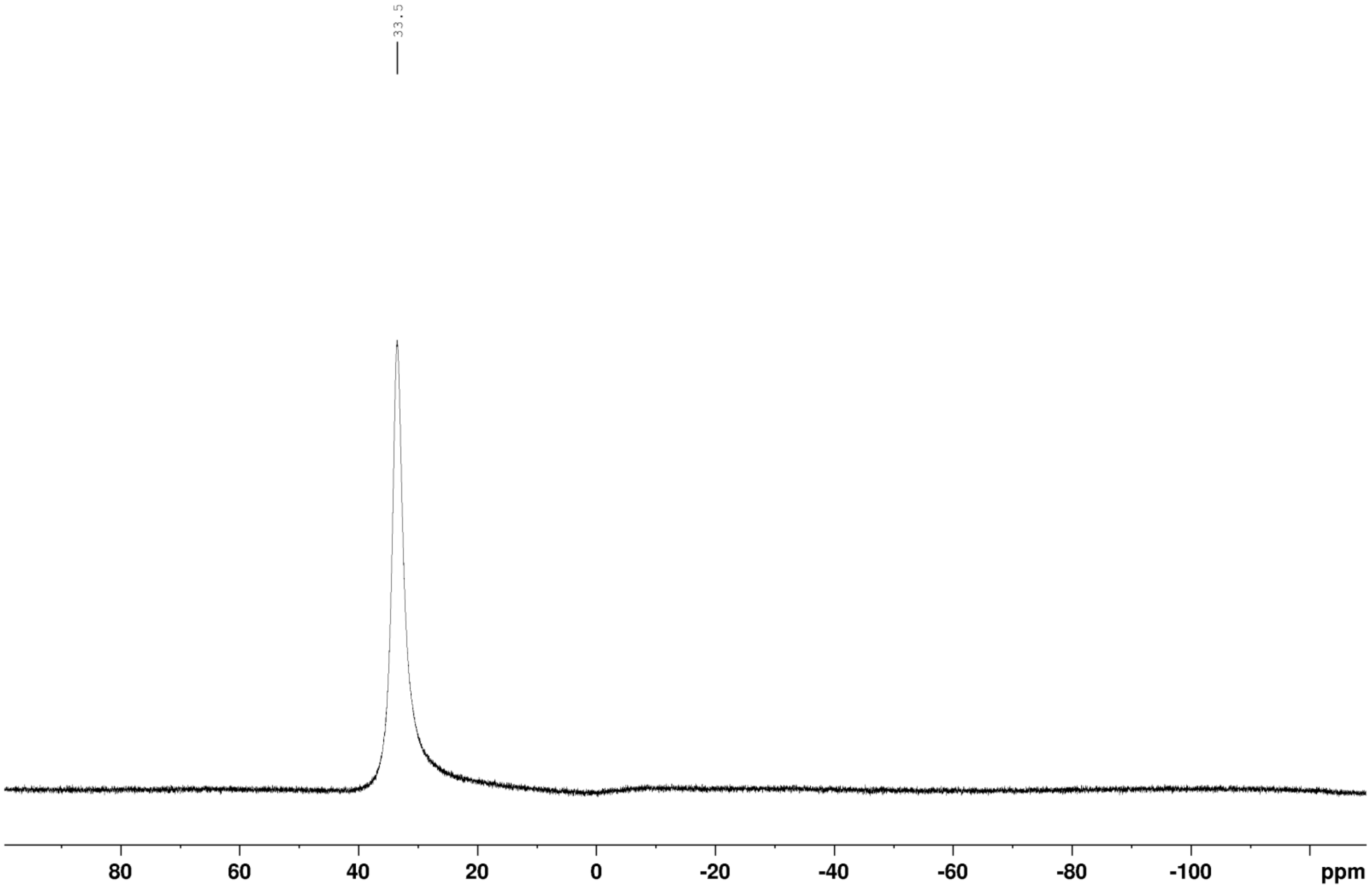

Figure S83. ^{1}H NMR (400 MHz, $CDCl_3$, 298 K) of **8a**.

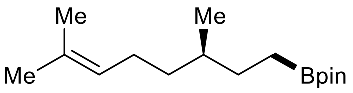

8a

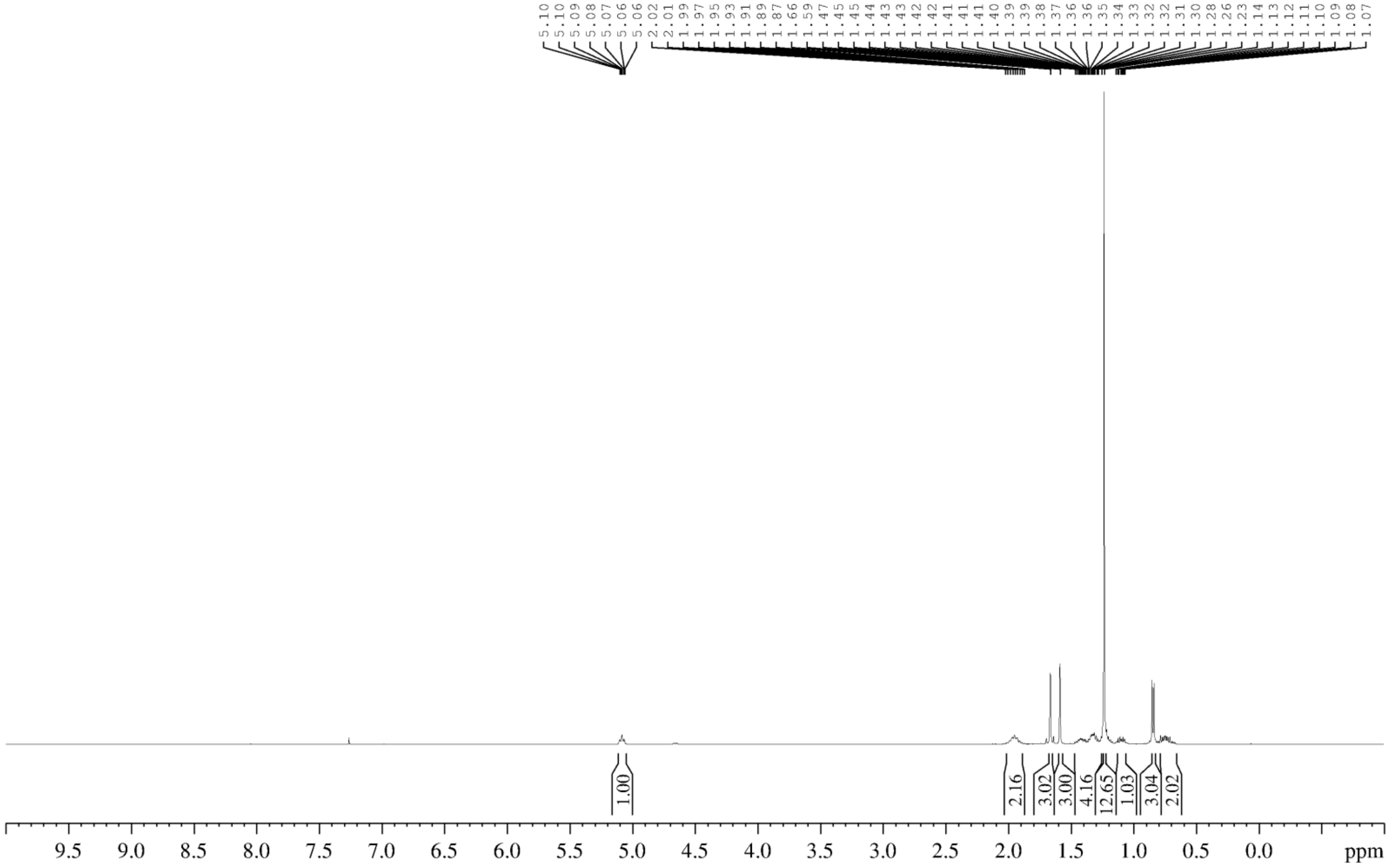

Figure S84. ^{13}C NMR (101 MHz, $CDCl_3$, 298 K) of **8a**.

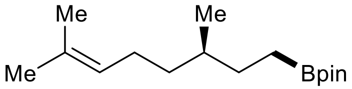

8a

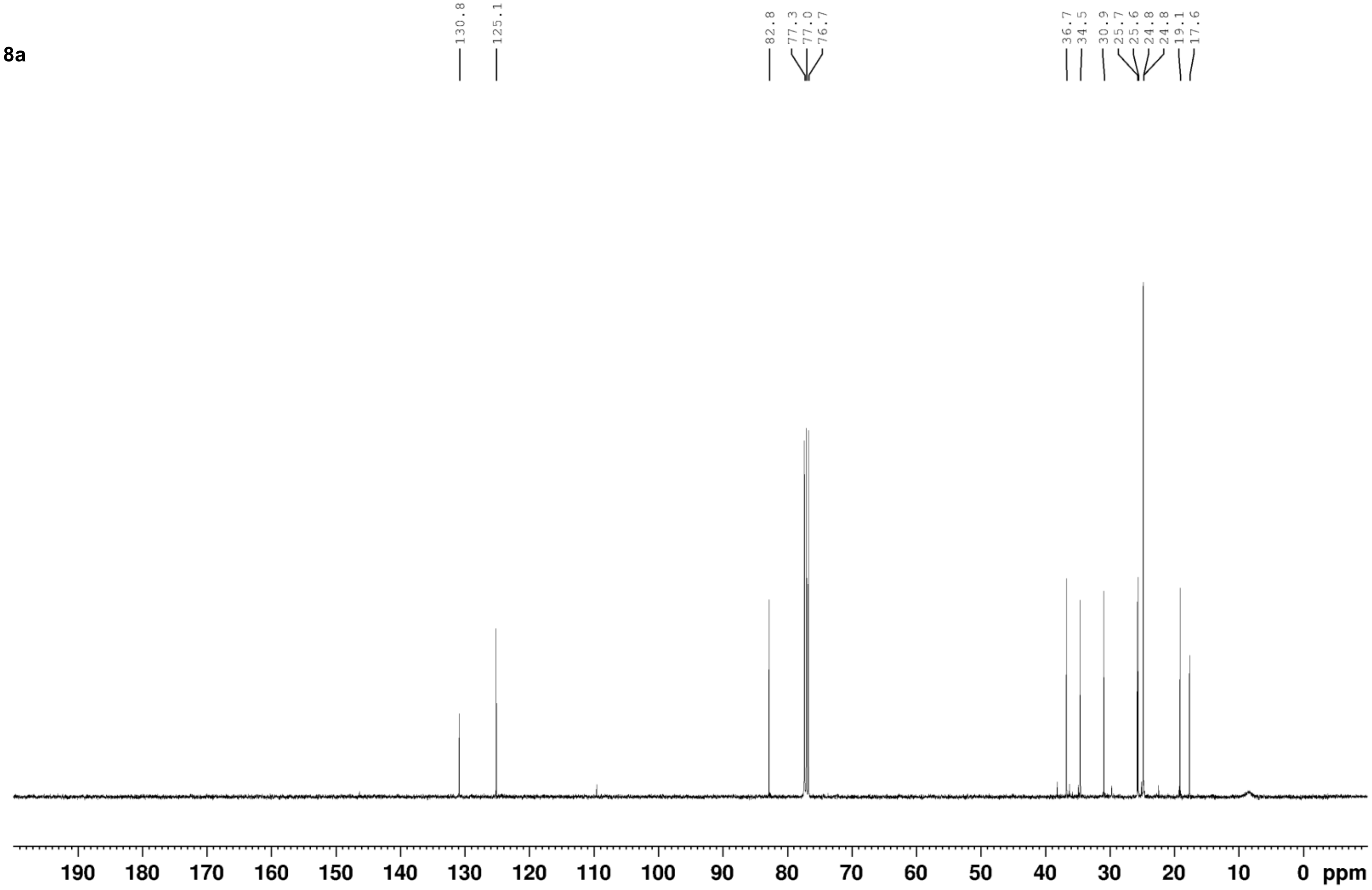

Figure S85. ^{13}B NMR (128 MHz, $CDCl_3$, 298 K) of **8a**.

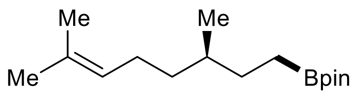

8a

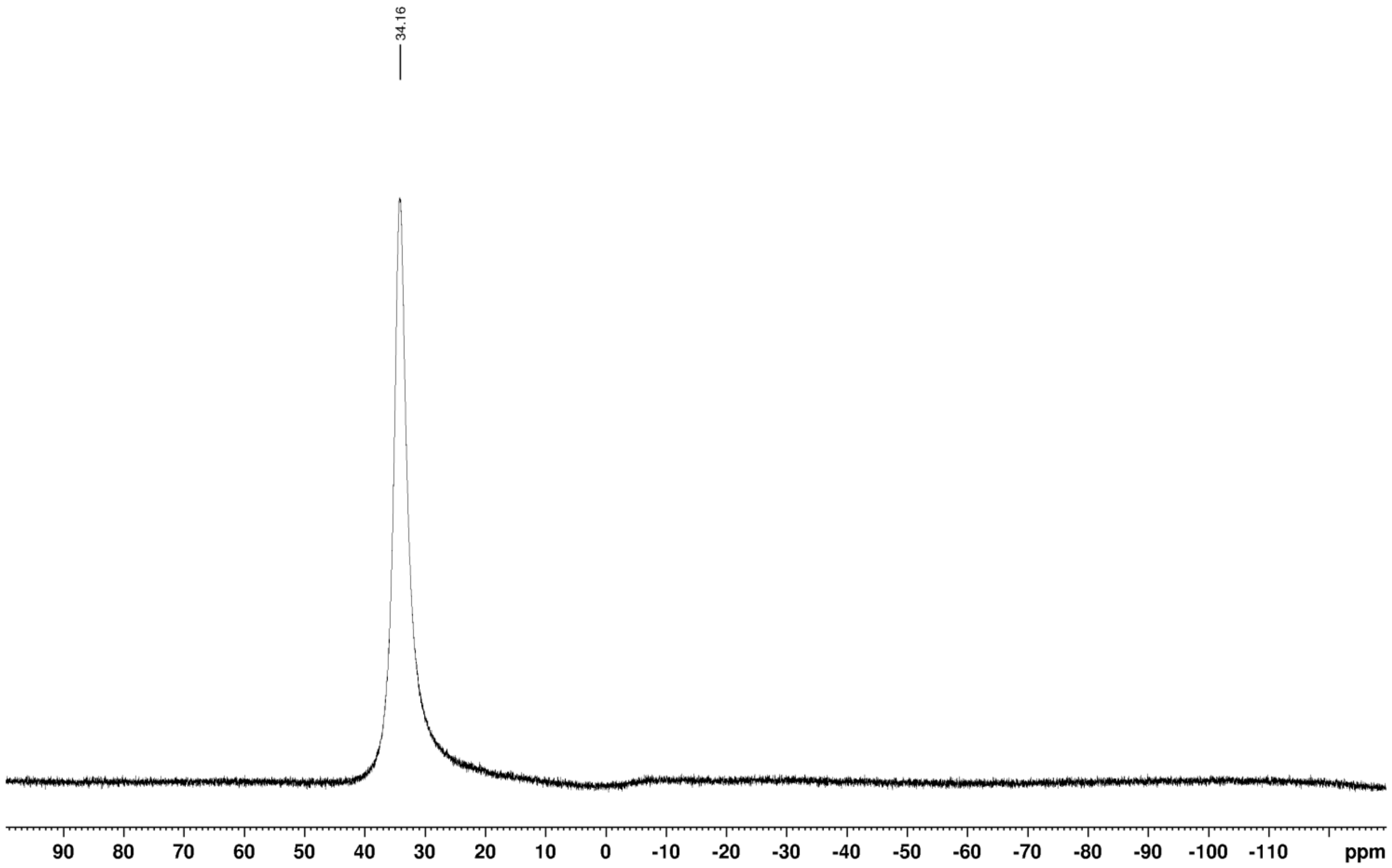

Figure S86. ^{1}H NMR (400 MHz, $CDCl_3$, 298 K) of **8b**.

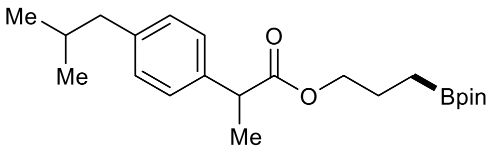

8b

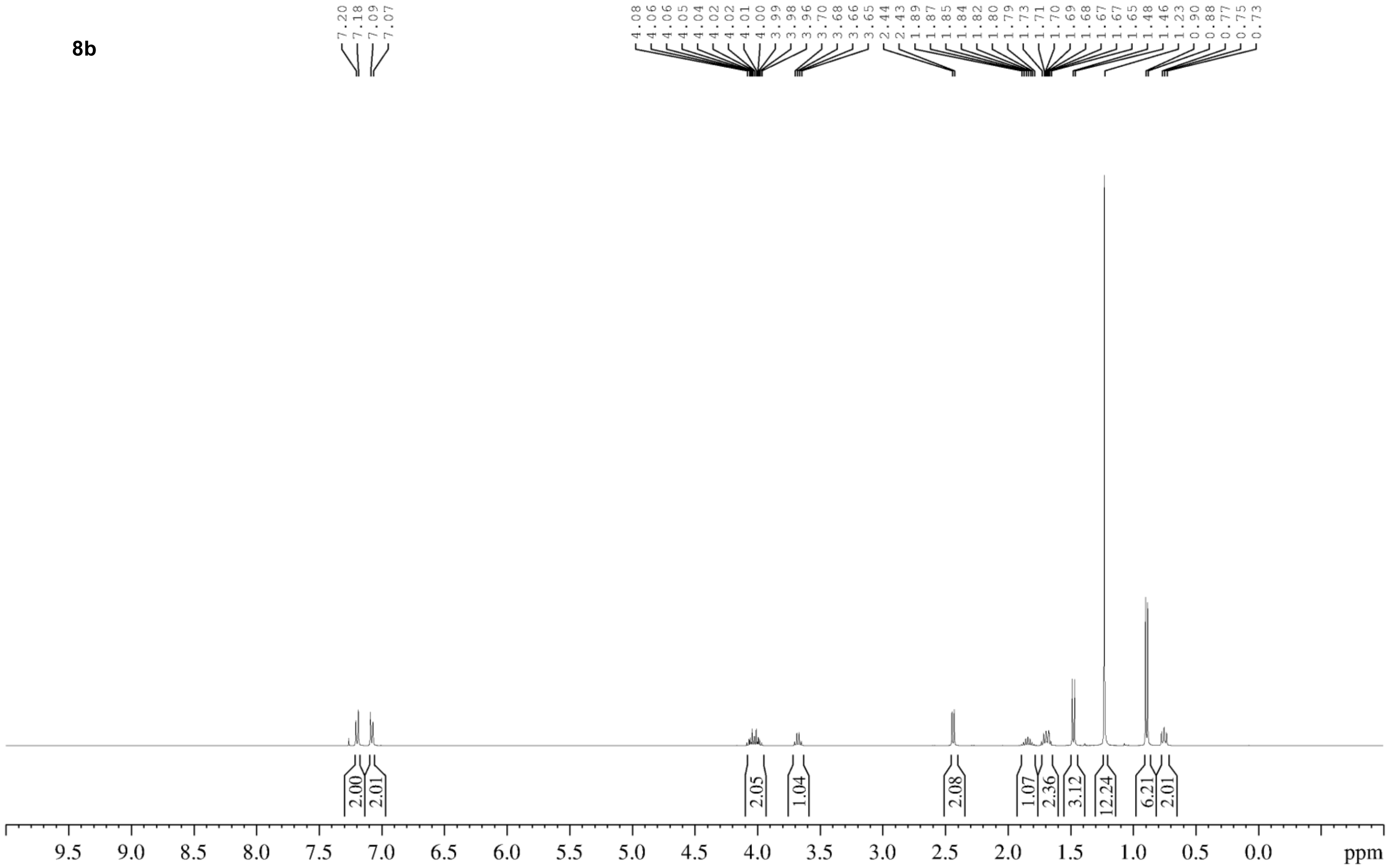

Figure S87. ^{13}C NMR (101 MHz, $CDCl_3$, 298 K) of **8b**.

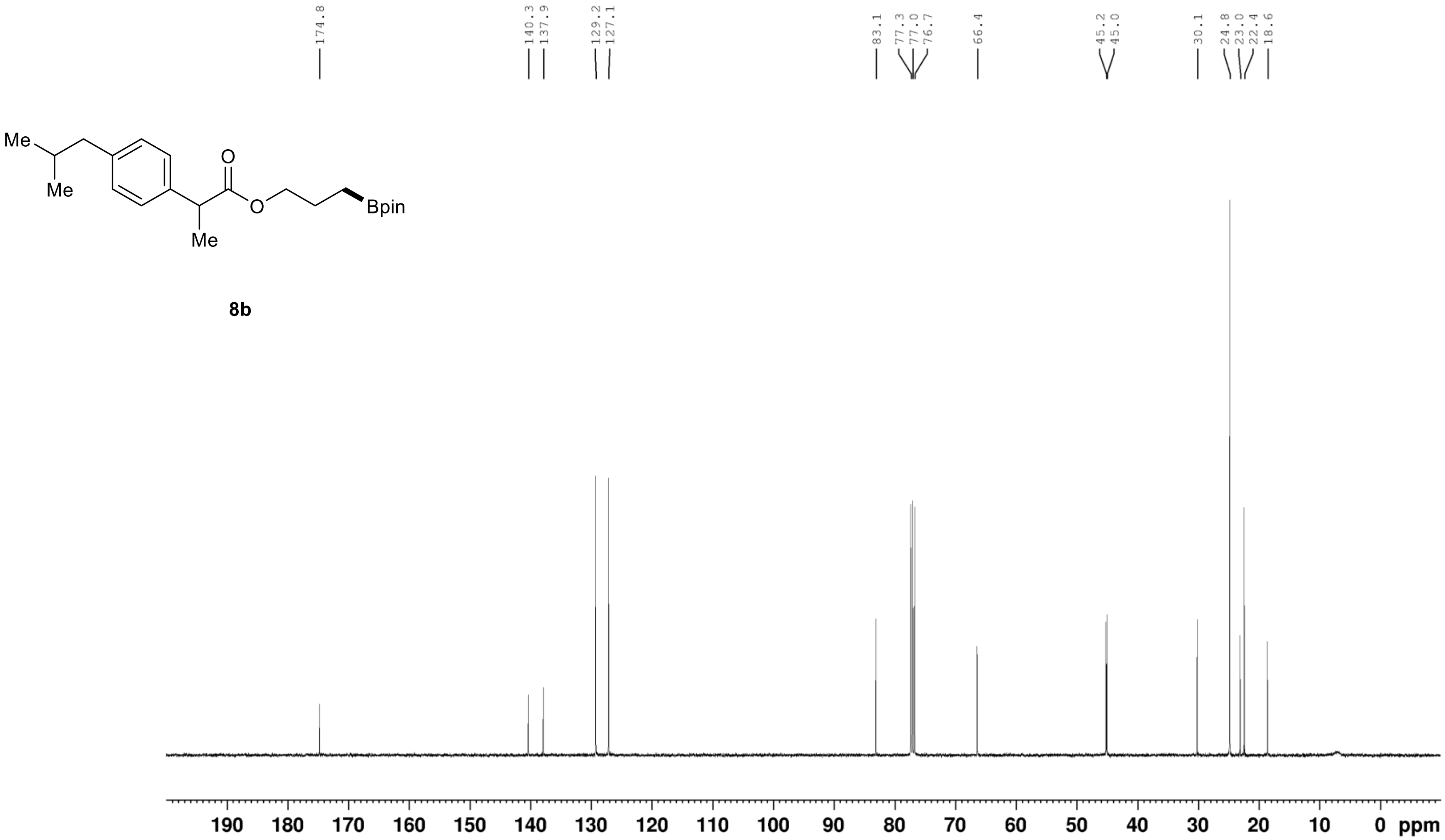

Figure S88. ^{11}B NMR (128 MHz, $CDCl_3$, 298 K) of **8b**.

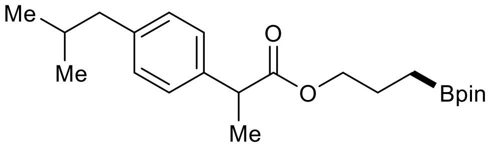

8b

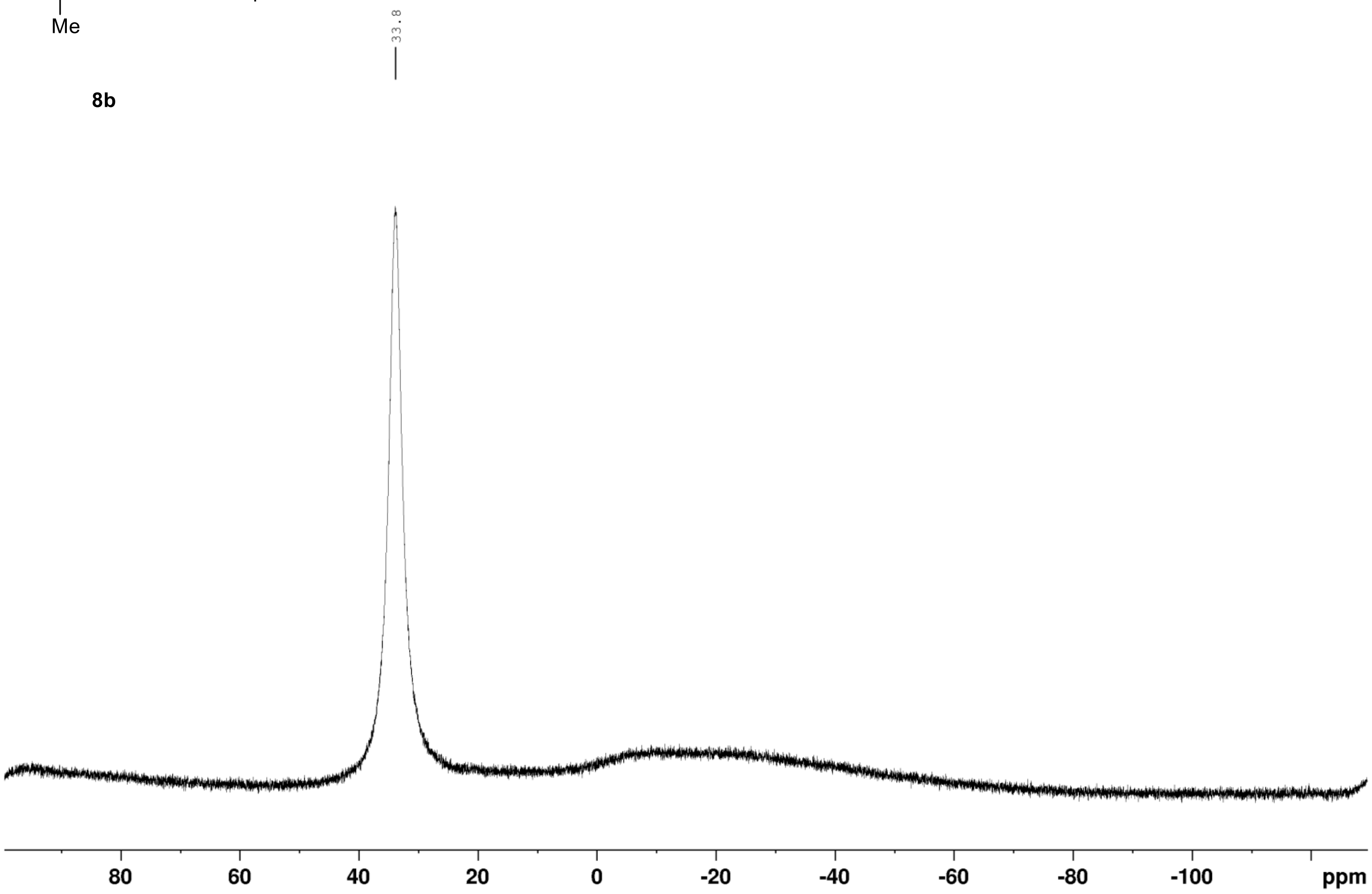

Figure S89. ^{1}H NMR (400 MHz, $CDCl_3$, 298 K) of **8c**.

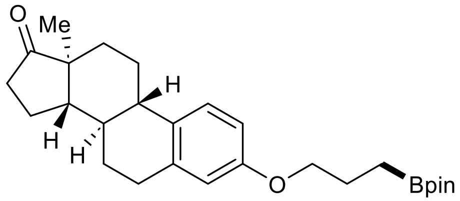

8c

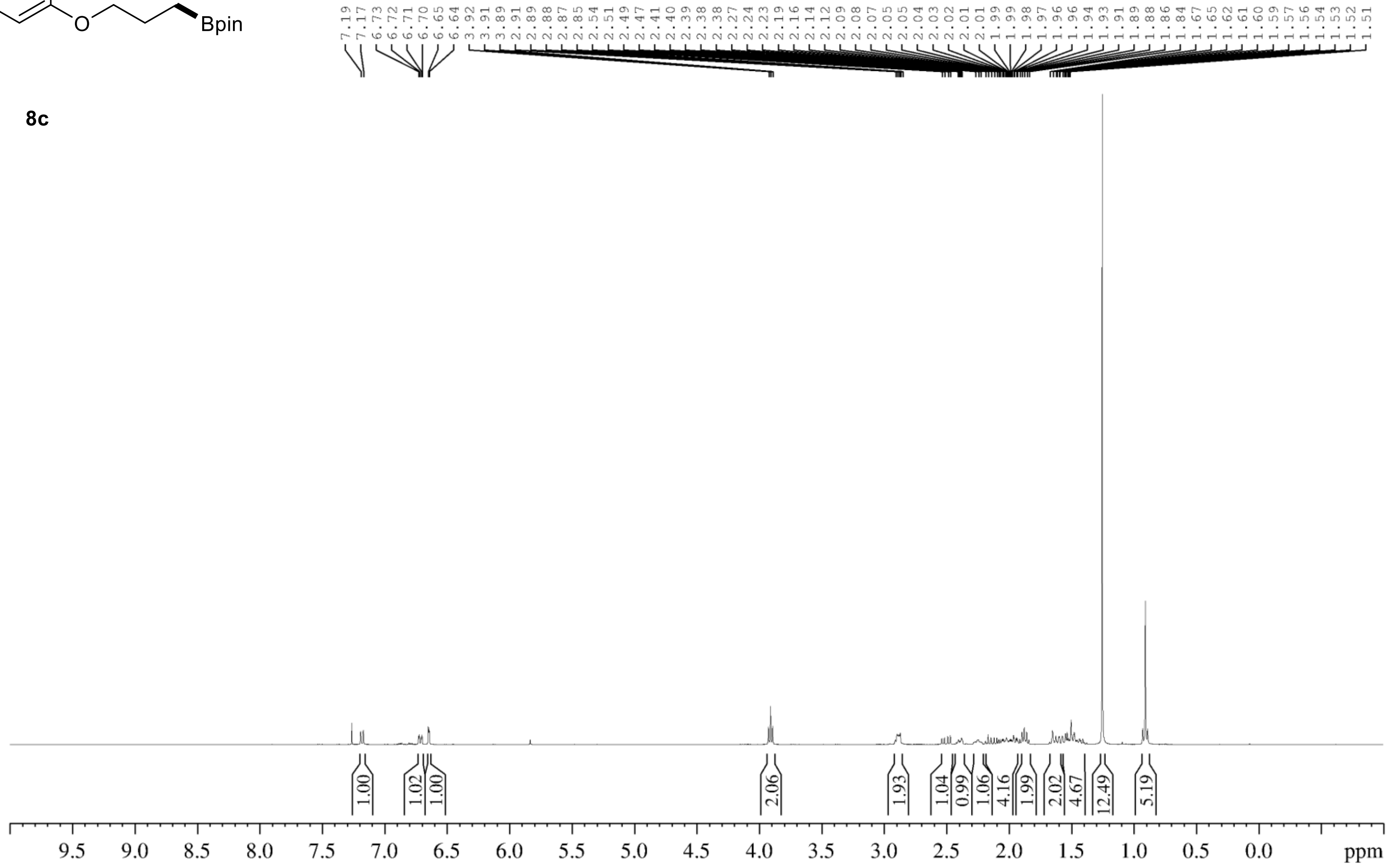

Figure S90. ^{13}C NMR (101 MHz, $CDCl_3$, 298 K) of **8c**.

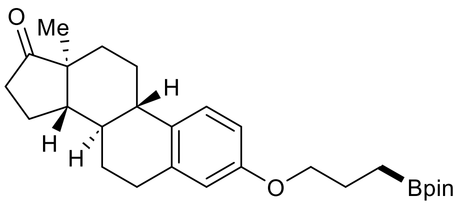

8c

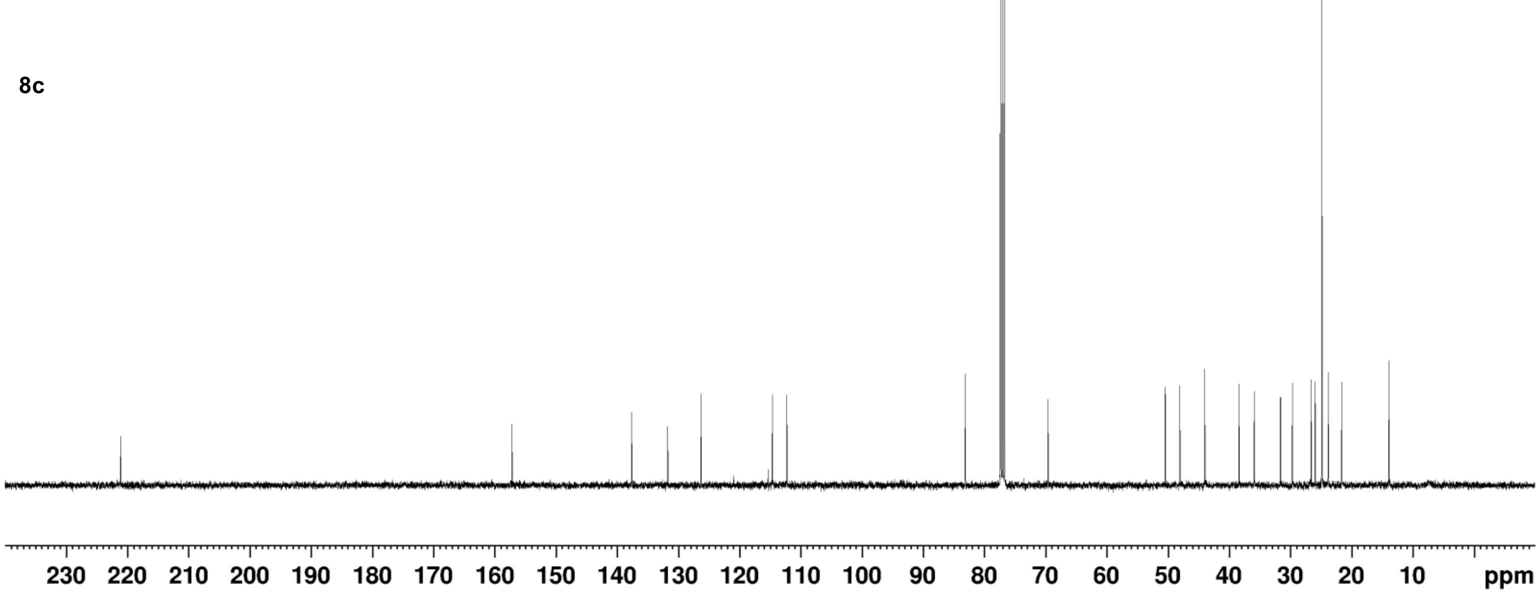

Figure S91. ^{11}B NMR (128 MHz, $CDCl_3$, 298 K) of **8c**.

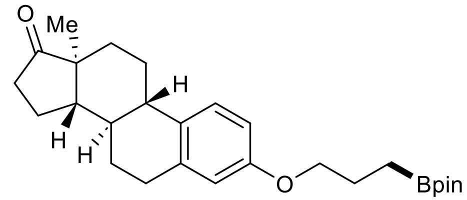

8c

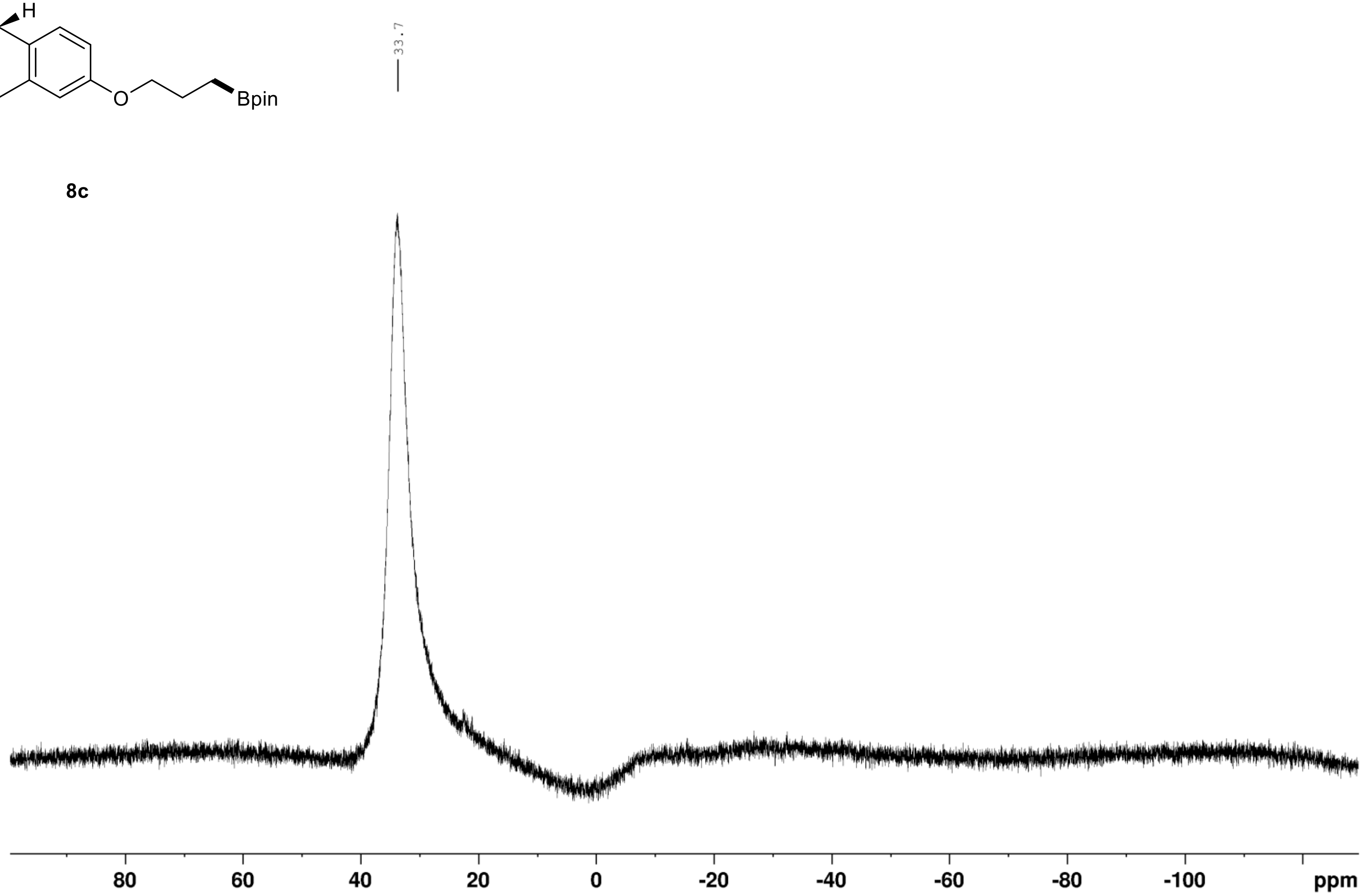

Figure S92. ^{1}H NMR (400 MHz, $CDCl_3$, 298 K) of **8d**.

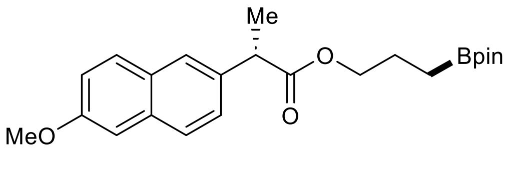

8d

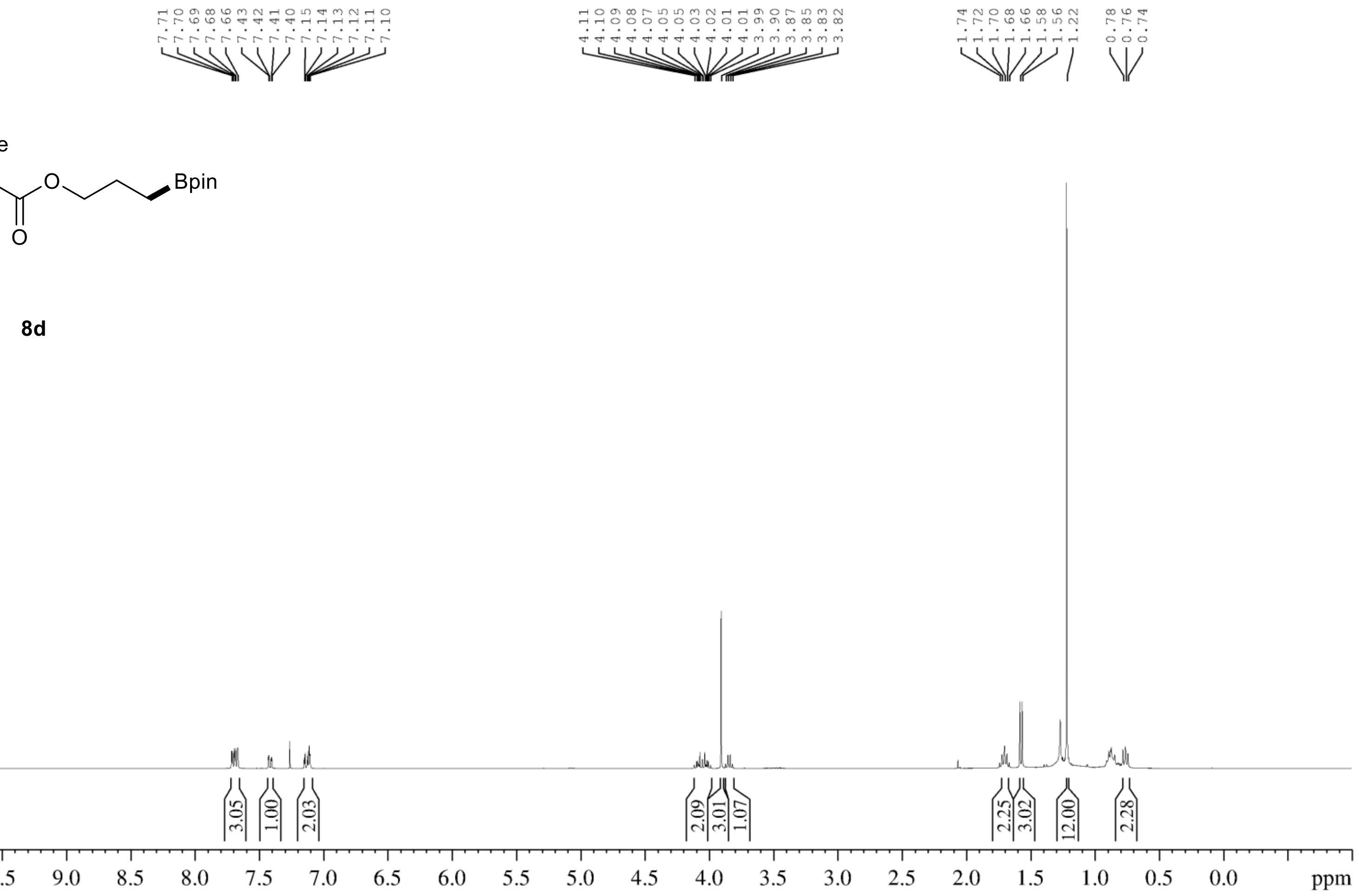

Figure S93. ^{13}C NMR (101 MHz, $CDCl_3$, 298 K) of **8d**.

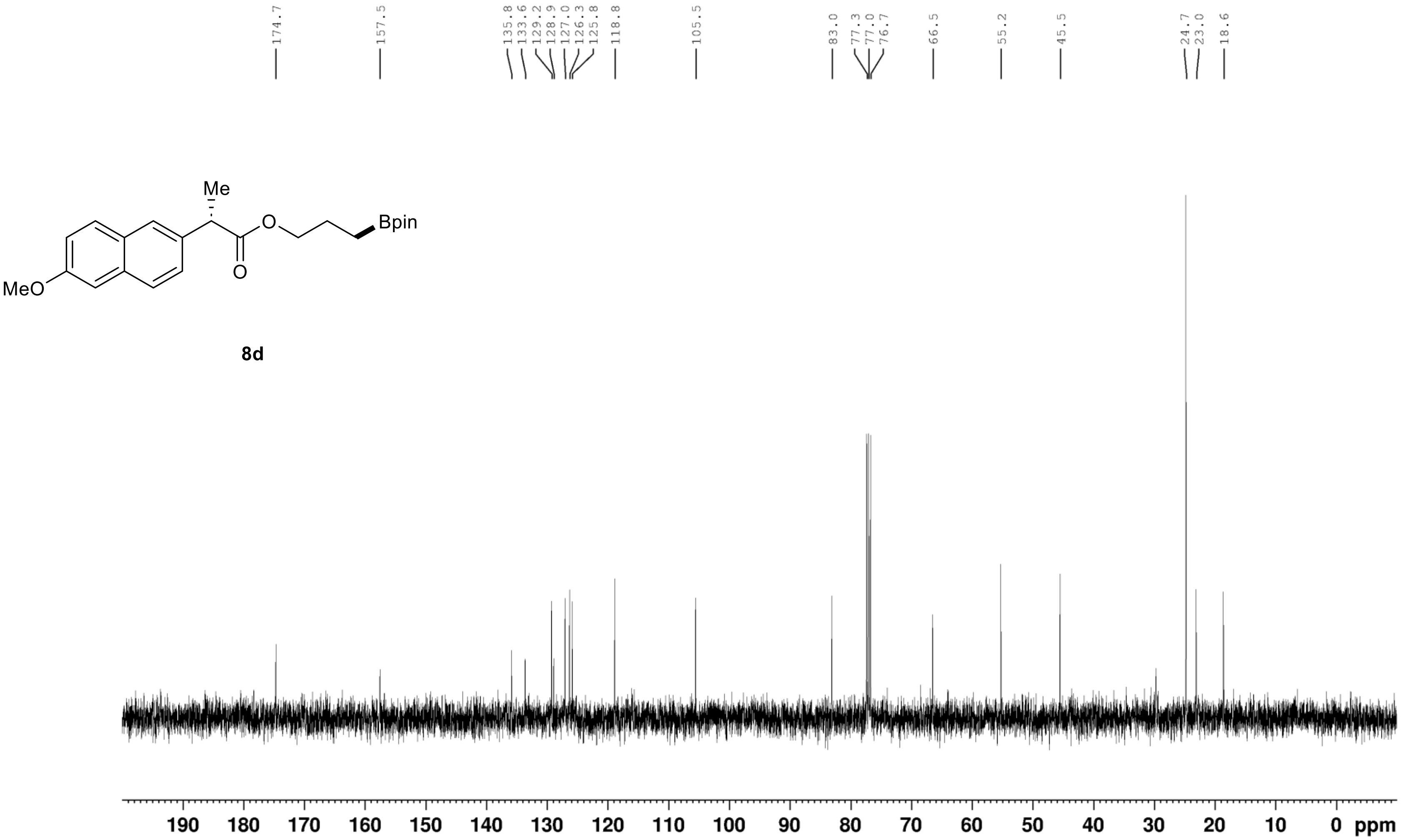

Figure S94. ^{11}B NMR (128 MHz, $CDCl_3$, 298 K) of **8d**.

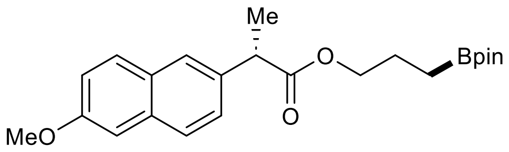

8d

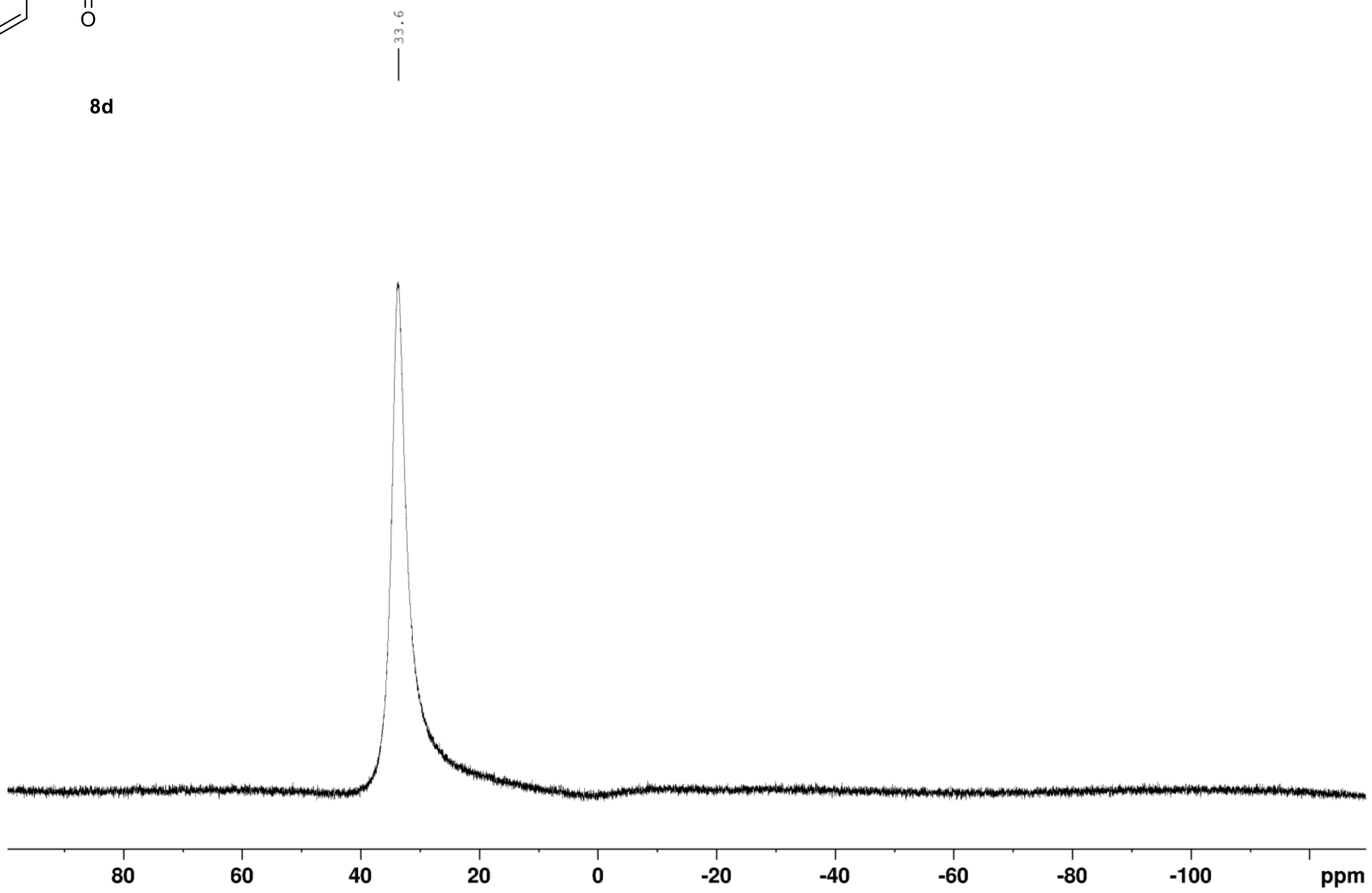

Figure S95. ^{1}H NMR (400 MHz, $CDCl_3$, 298 K) of **8e**.

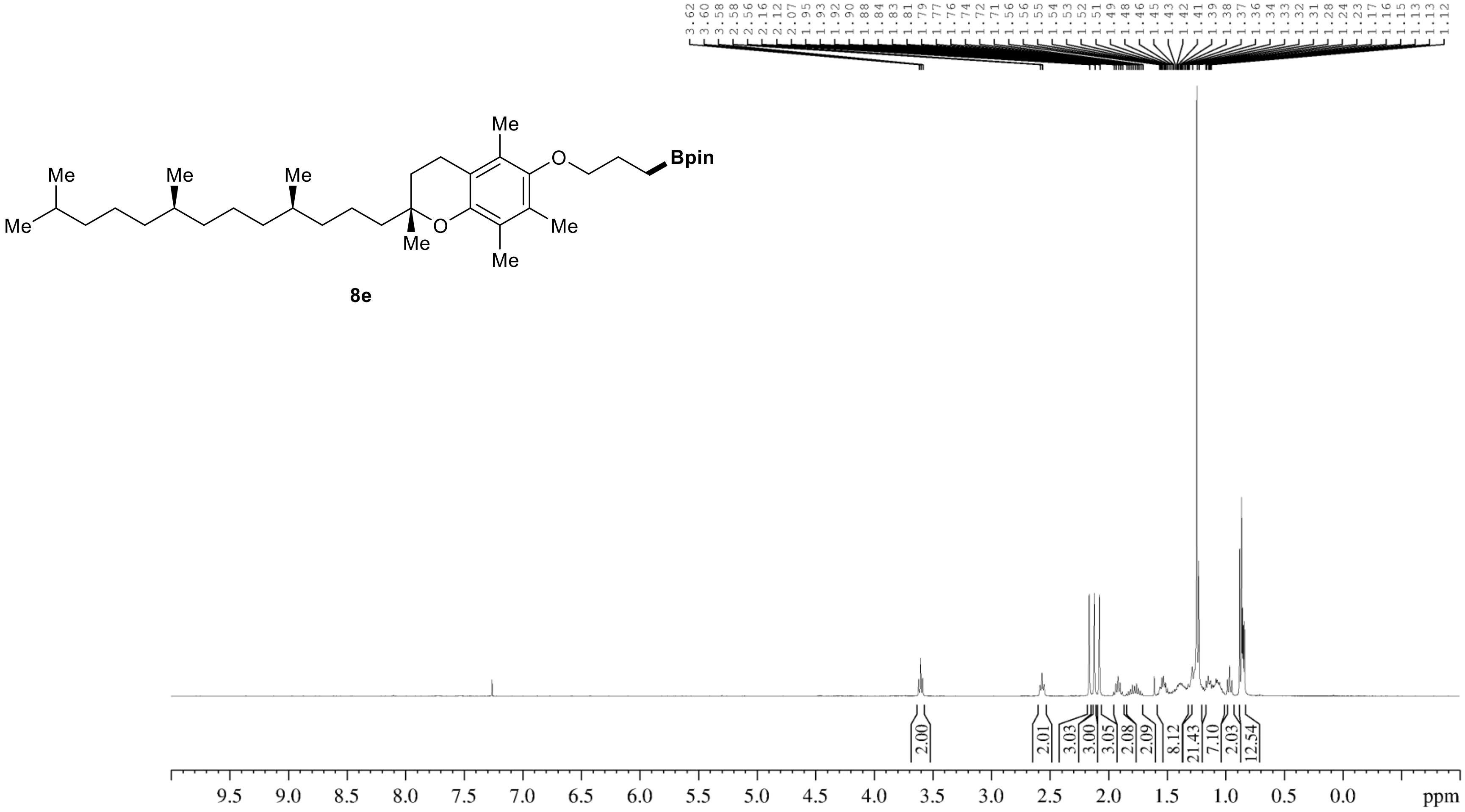

Figure S96. ^{13}C NMR (101 MHz, $CDCl_3$, 298 K) of **8e**.

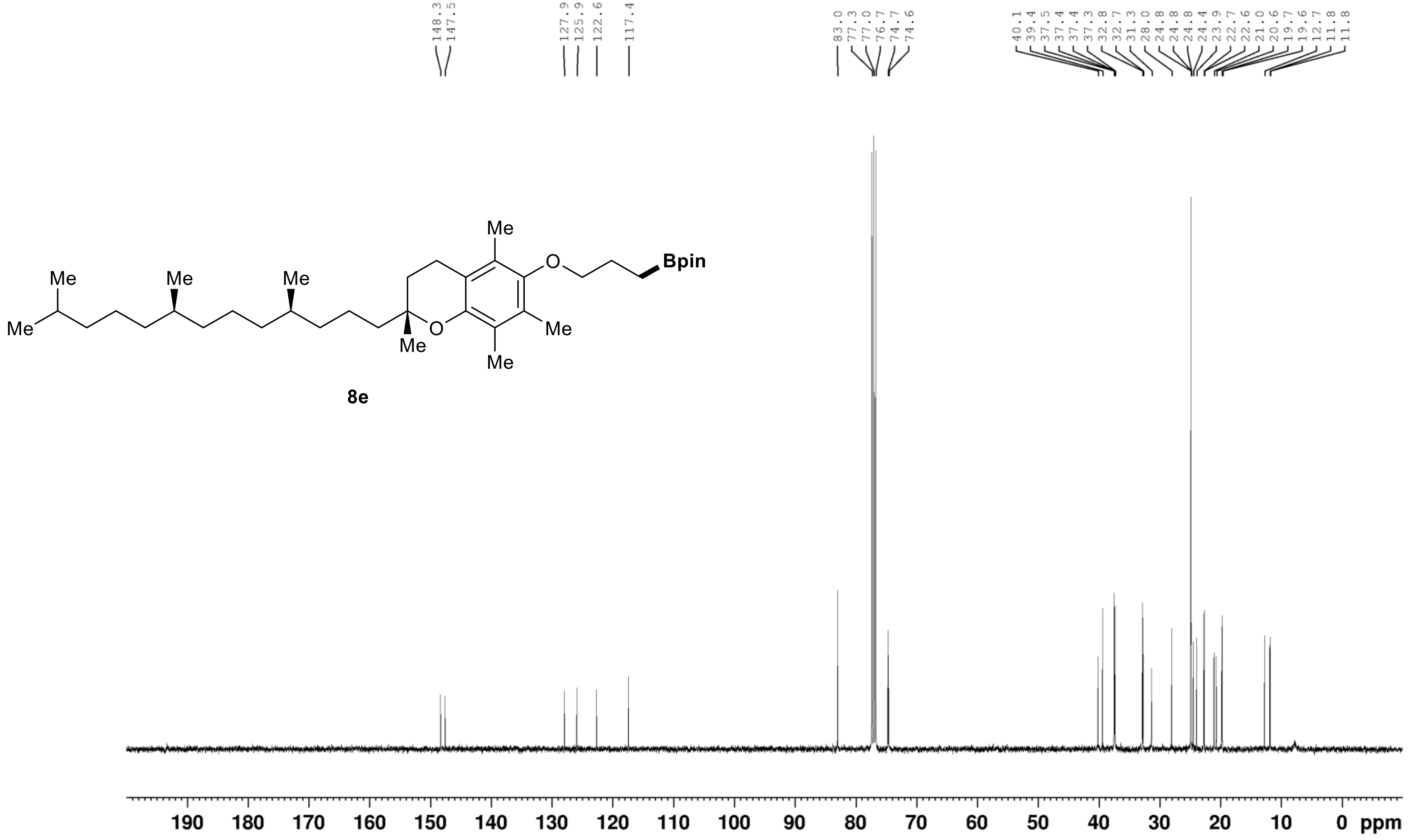

Figure S97. ^{11}B NMR (128 MHz, $CDCl_3$, 298 K) of **8e**.

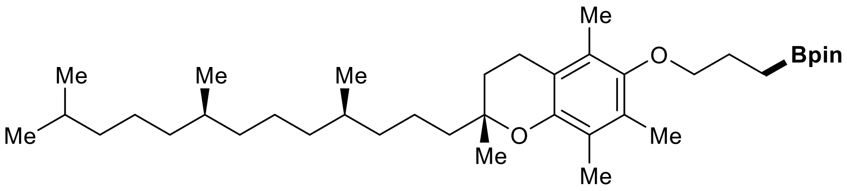

8e

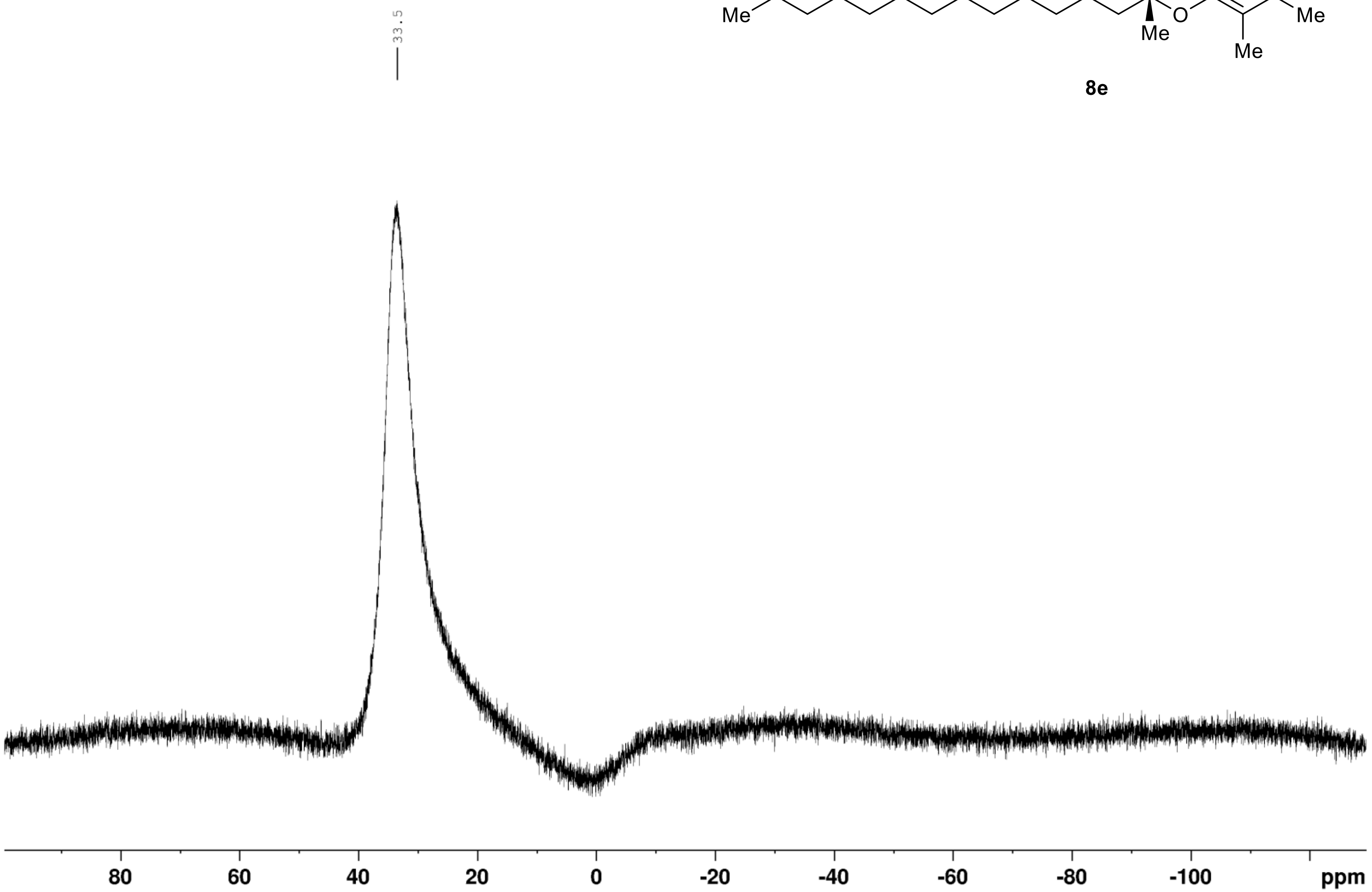

Figure S98. ^{1}H NMR (400 MHz, $CDCl_3$, 298 K) of **9a**.

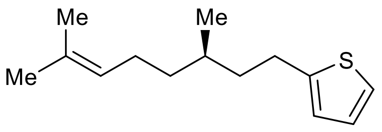

9a

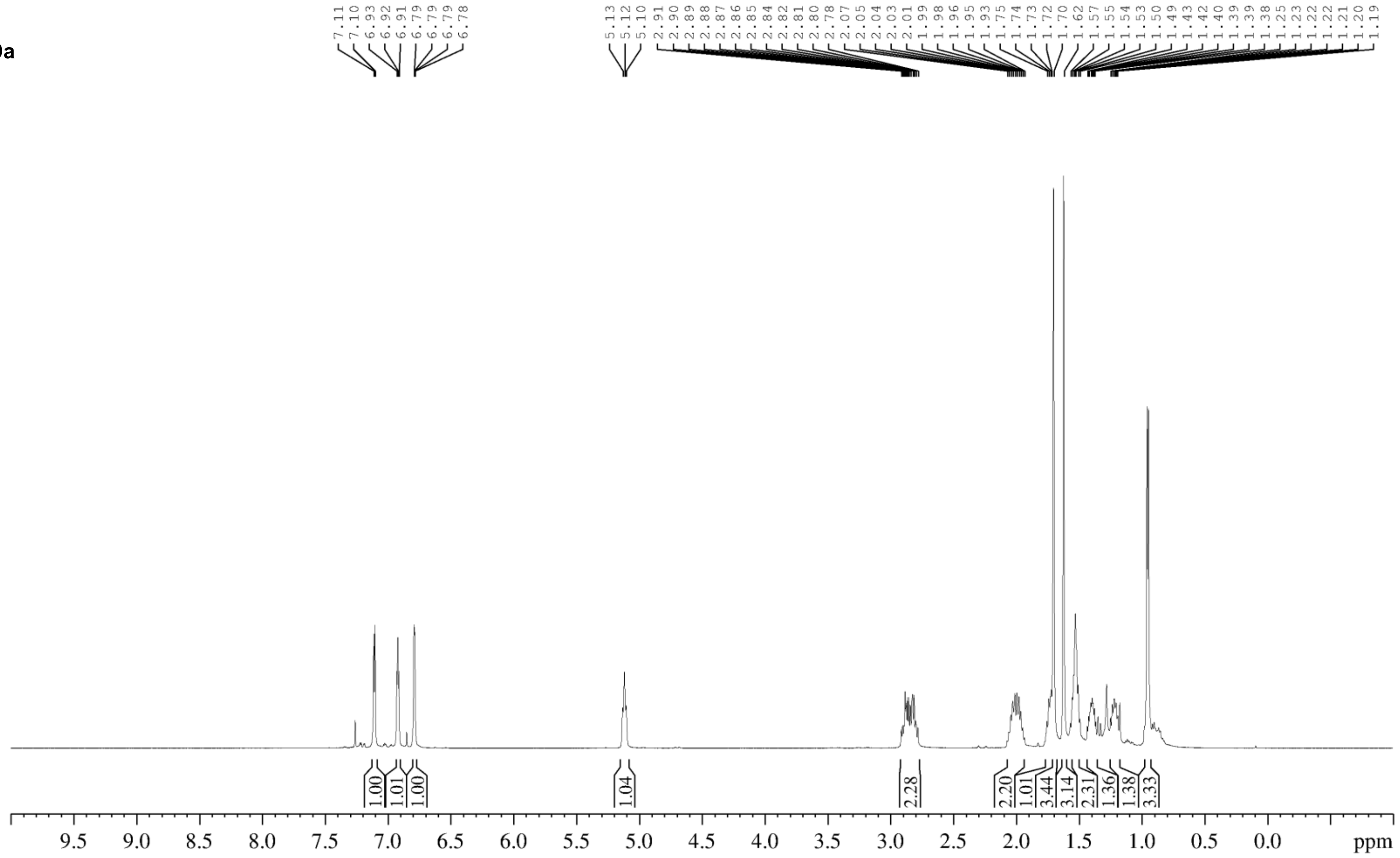

Figure S99. ^{13}C NMR (101 MHz, $CDCl_3$, 298 K) of **9a**.

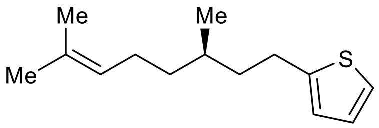

9a

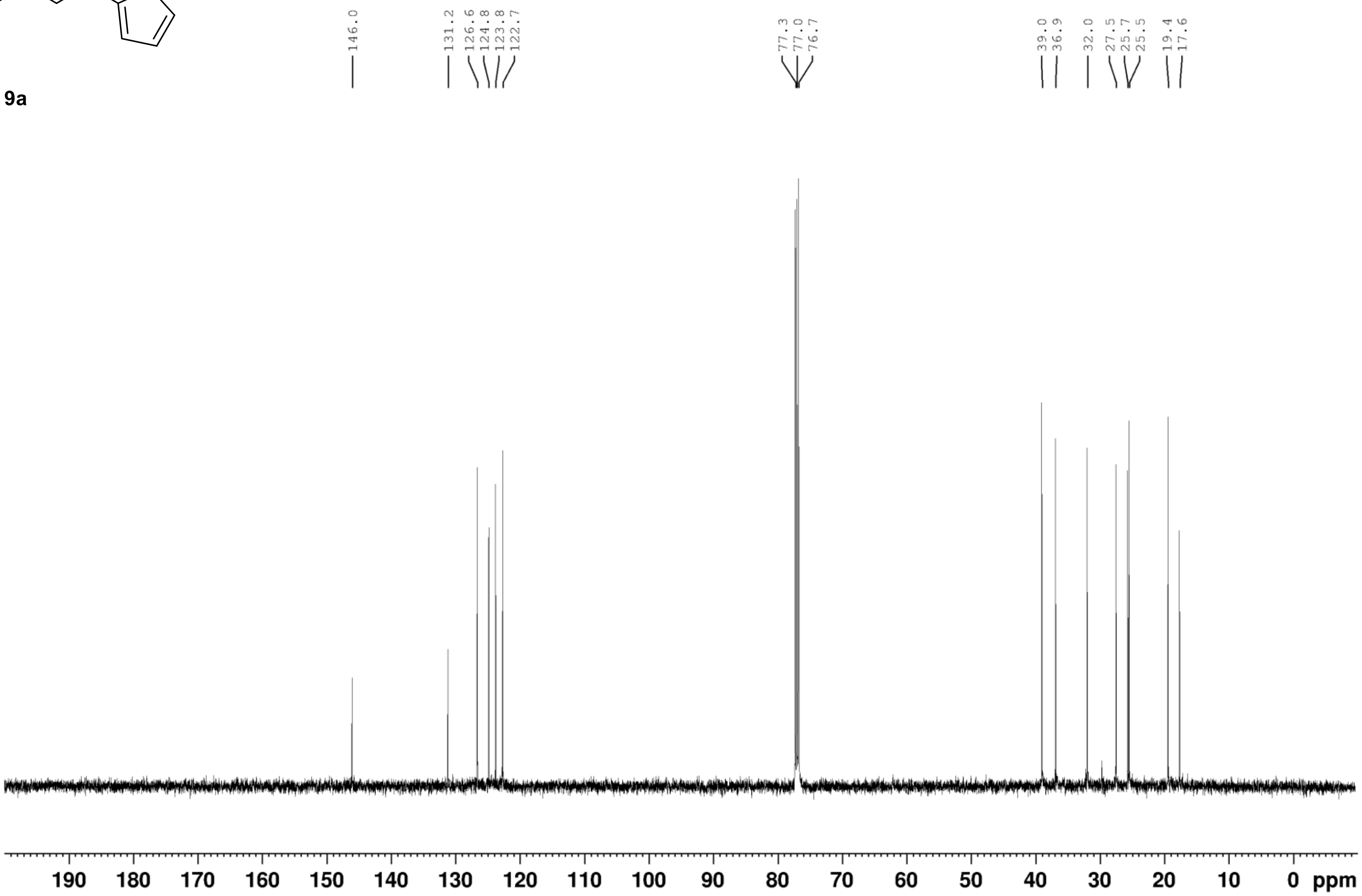

Figure S100. ^{1}H NMR (400 MHz, $CDCl_3$, 298 K) of **9b**.

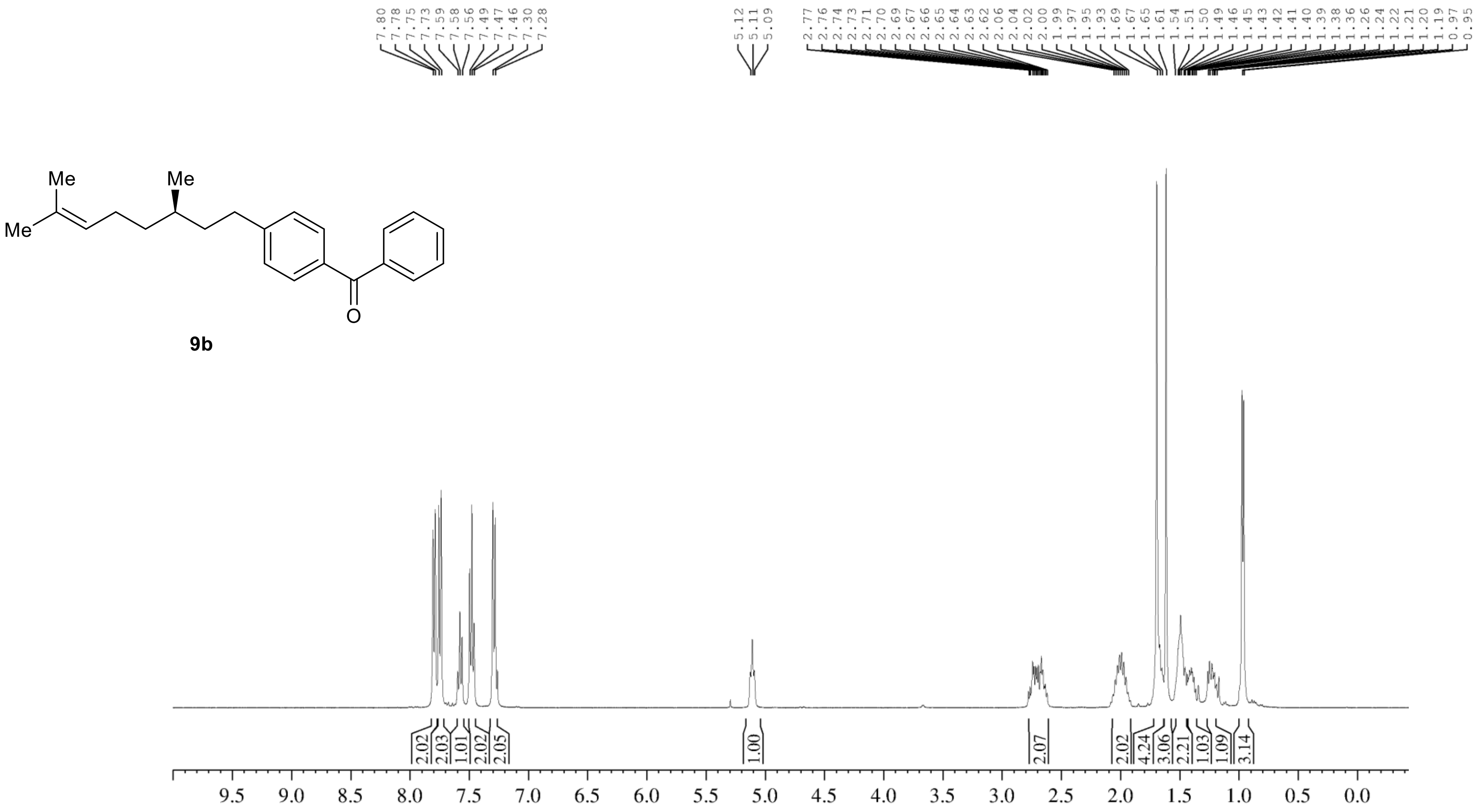

9b

Figure S101. ^{13}C NMR (101 MHz, $CDCl_3$, 298 K) of **9b**.

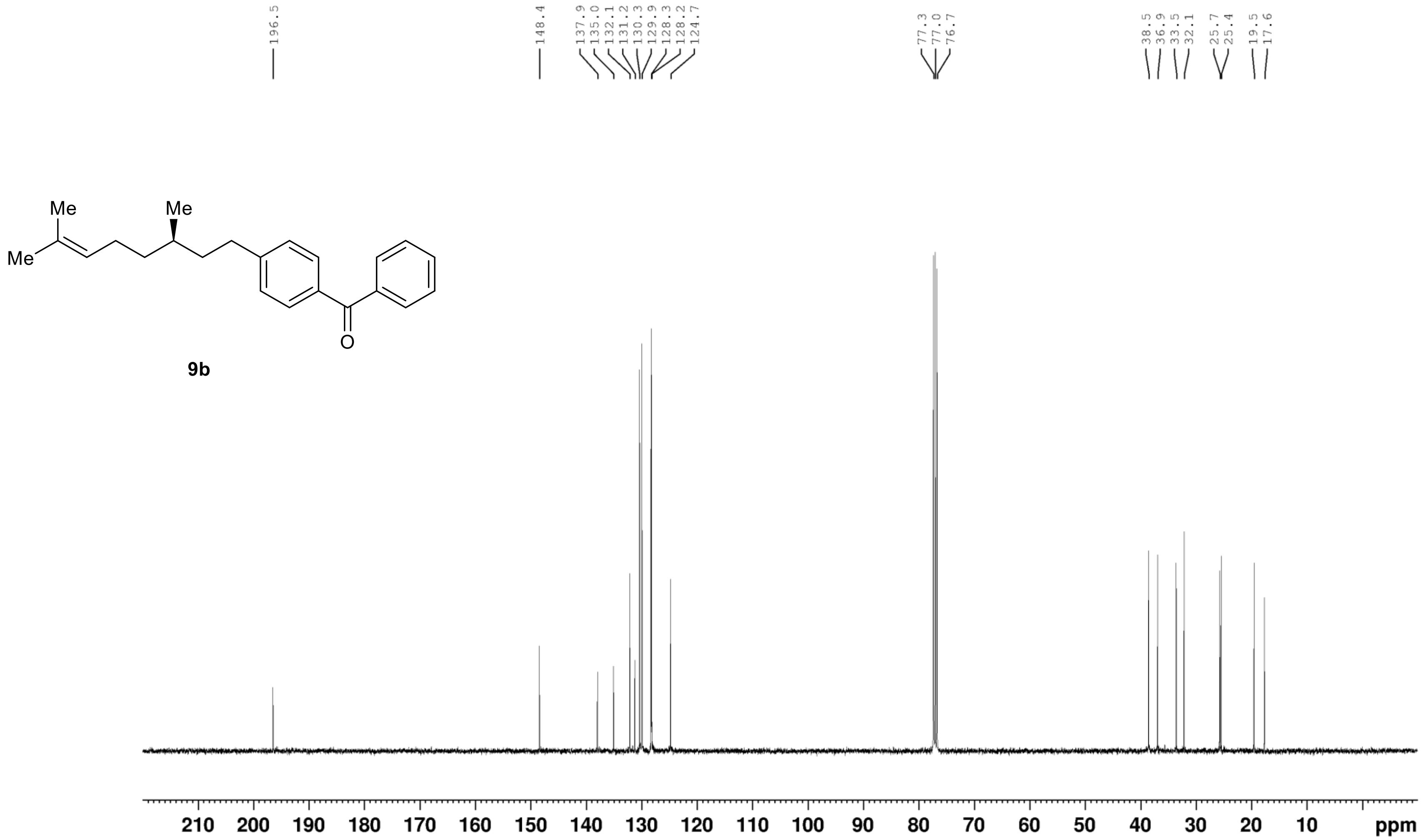

Figure S102. ^{1}H NMR (400 MHz, $CDCl_3$, 298 K) of **9c**.

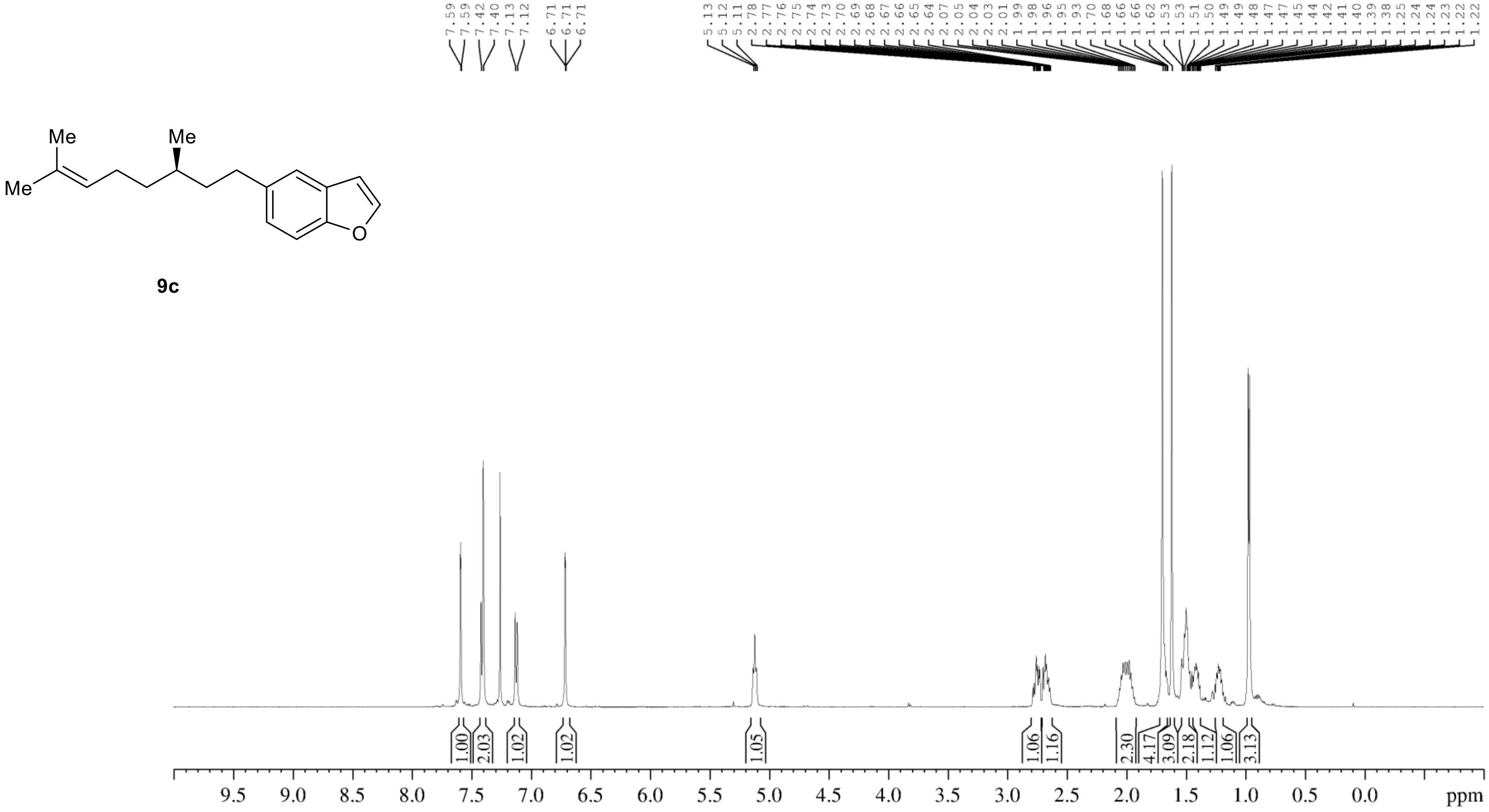

9c

Figure S103. ^{13}C NMR (101 MHz, $CDCl_3$, 298 K) of **9c**.

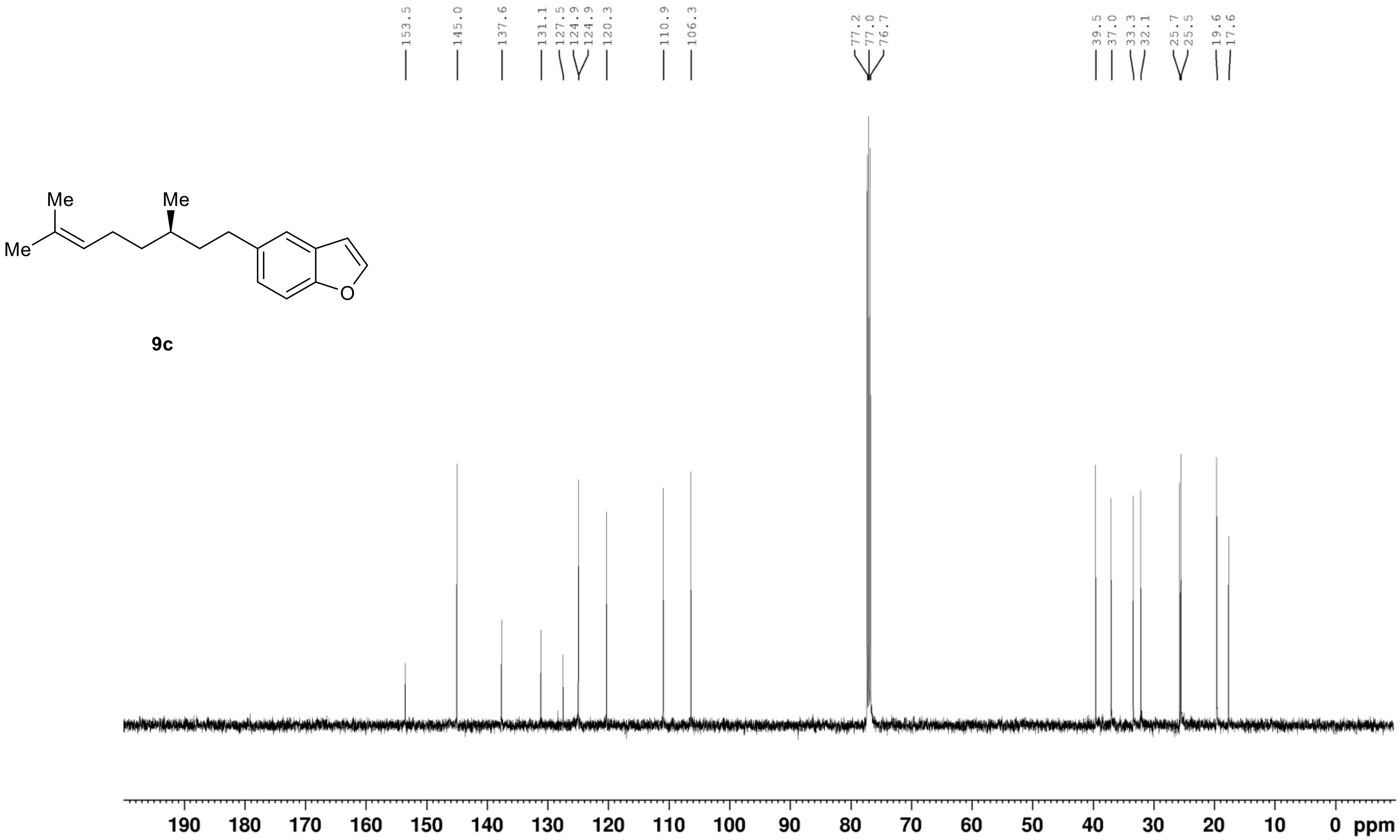

9c

Figure S104. ^{1}H NMR (400 MHz, $CDCl_3$, 298 K) of **9d**.

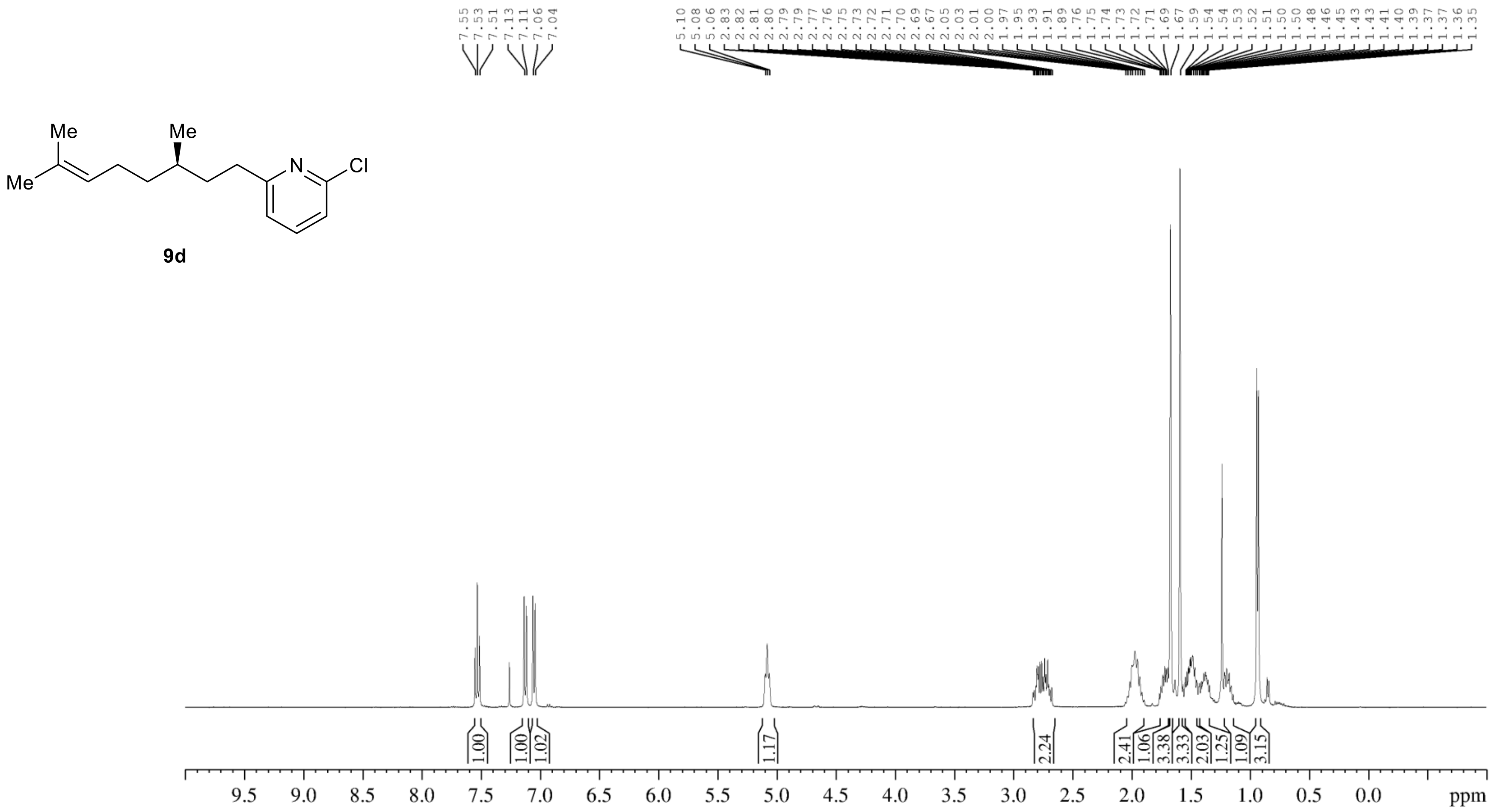

9d

Figure S105. ^{13}C NMR (101 MHz, $CDCl_3$, 298 K) of **9d**.

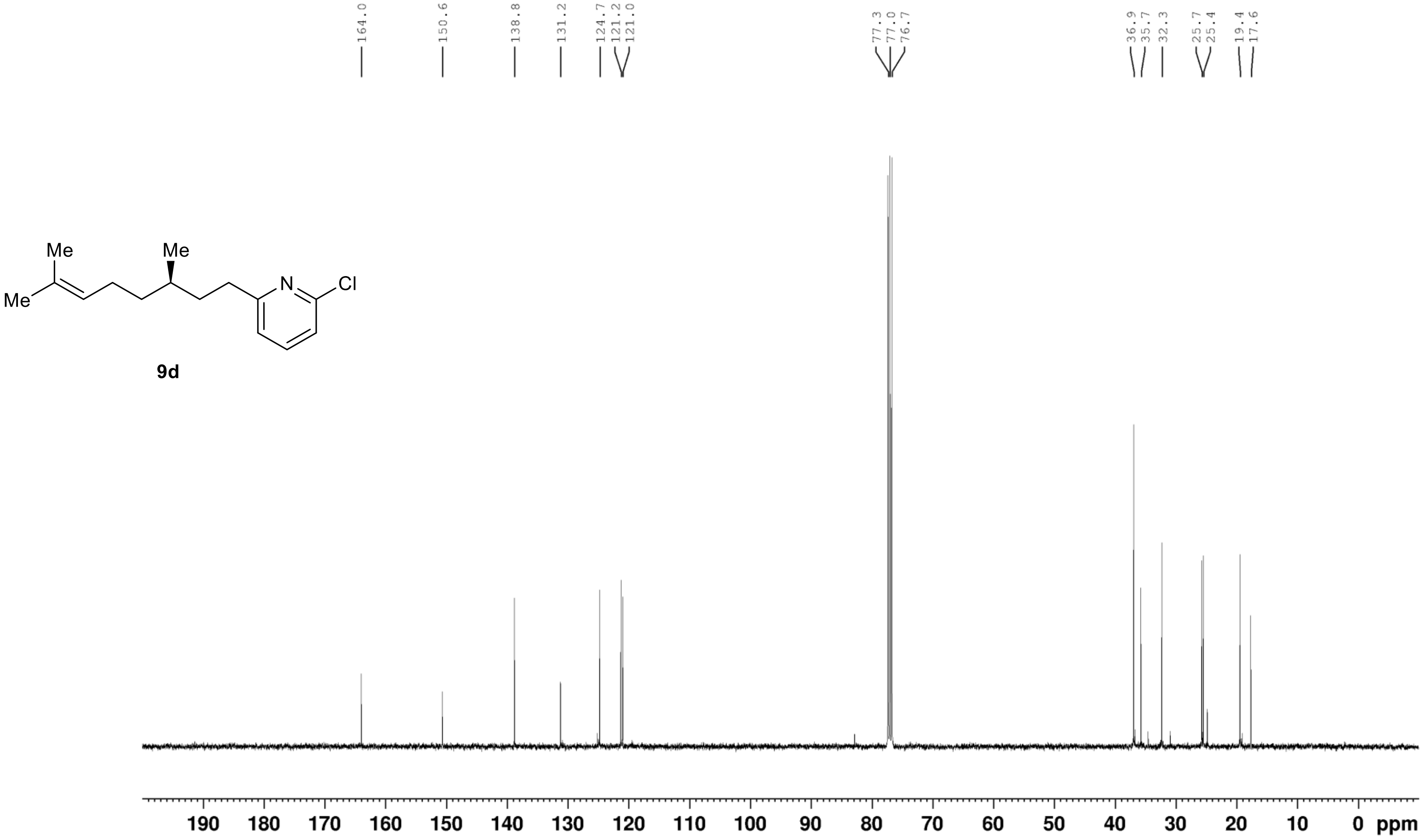

9d

6 References

[S1] Liu, X.; Xu, B.; Oestreich, M. One-Pot Sequential Alcohol Activation and Nickel-Catalyzed Cross-Electrophile Coupling with Chlorosilanes. *Org. Lett.* **2025**, *27*, 3686-3690.

[S2] Dudnik, A. S.; Fu, G. C. Nickel-catalyzed coupling reactions of alkyl electrophiles, including unactivated tertiary halides, to generate carbon-boron bonds. *J. Am. Chem. Soc.* **2012**, *134*, 10693–10697.

[S3] Bonet, A.; Odachowski, M.; Leonori, D.; Essafi, S.; Aggarwal, V. K. Enantiospecific sp^2-sp^3 coupling of secondary and tertiary boronic esters. *Nat. Chem.* **2014**, *6*, 584-589.

[S4] Han, J. T.; Lee, J. Y.; Yun, J. Asymmetric synthesis of gamma-chiral borylalkanes via sequential reduction/hydroboration using a single copper catalyst. *Chem. Sci.* **2020**, *11*, 8961-8965.

[S5] Laulhe, S.; Blackburn, J. M.; Roizen, J. L. Selective and Serial Suzuki-Miyaura Reactions of Polychlorinated Aromatics with Alkyl Pinacol Boronic Esters. *Org. Lett.* **2016**, *18*, 4440-4443.
